# Photocatalytic Umpolung of *N*- and *O*-substituted alkenes for the synthesis of 1,2-amino alcohols and diols†‡

**DOI:** 10.1039/d0sc03655b

**Published:** 2020-09-22

**Authors:** Stephanie G. E. Amos, Stefano Nicolai, Jerome Waser

**Affiliations:** Laboratory of Catalysis and Organic Synthesis, Institut des Sciences et Ingénierie Chimique, Ecole Polytechnique Fédérale de Lausanne Ch-1015 Lausanne Switzerland jerome.waser@epfl.ch

## Abstract

We report an organophotocatalytic 1,2-oxyalkynylation of ene-carbamates and enol ethers using Ethynyl BenziodoXolones (EBXs). 1-Alkynyl-1,2-amino alcohols and diols were obtained in up to 89% yield. Photocatalytic formation of radical cations led to Umpolung of the innate reactivity of the alkenes, enabling addition of a nucleophilic benzoate followed by radical alkynylation.

## Abstract 2

Photocatalytic Umpolung with organic dyes overcoming the innate nucleophilicity of enecarbamates and enol ethers for oxyalkynylation with EBX reagents to access 1-alkynyl-1,2-amino alcohols and diols.

## Graphical abstract

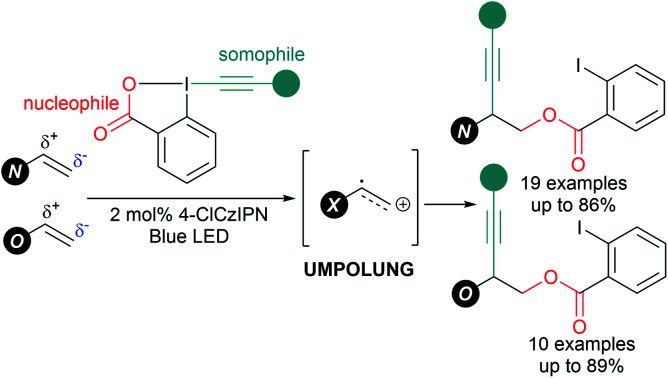

## 1. Introduction

Accessing 1,2-amino-alcohols and 1,2-diols has been a longstanding target in synthetic methodology. These scaffolds have found multiple applications in pharmaceutical, material and agrochemical sciences.^1,2^ Alkynes are highly useful building blocks for synthesis, as starting points for product diversification.^3^ Combining both functionalities, 1-alkynyl-1,2-amino alcohols can be found as intermediates in the synthesis of insecticidal 4-alkynyloxazolines^4^ and β-erythroidine,^5,6^ as well as essential structural elements in bioactive antitumoral enediynes.^7,8^ 1-Alkynyl-1,2-diols can be found, for example, in the Petrosiol family of neurotrophic diyne tetraols.^9,10^

**Scheme 1:**
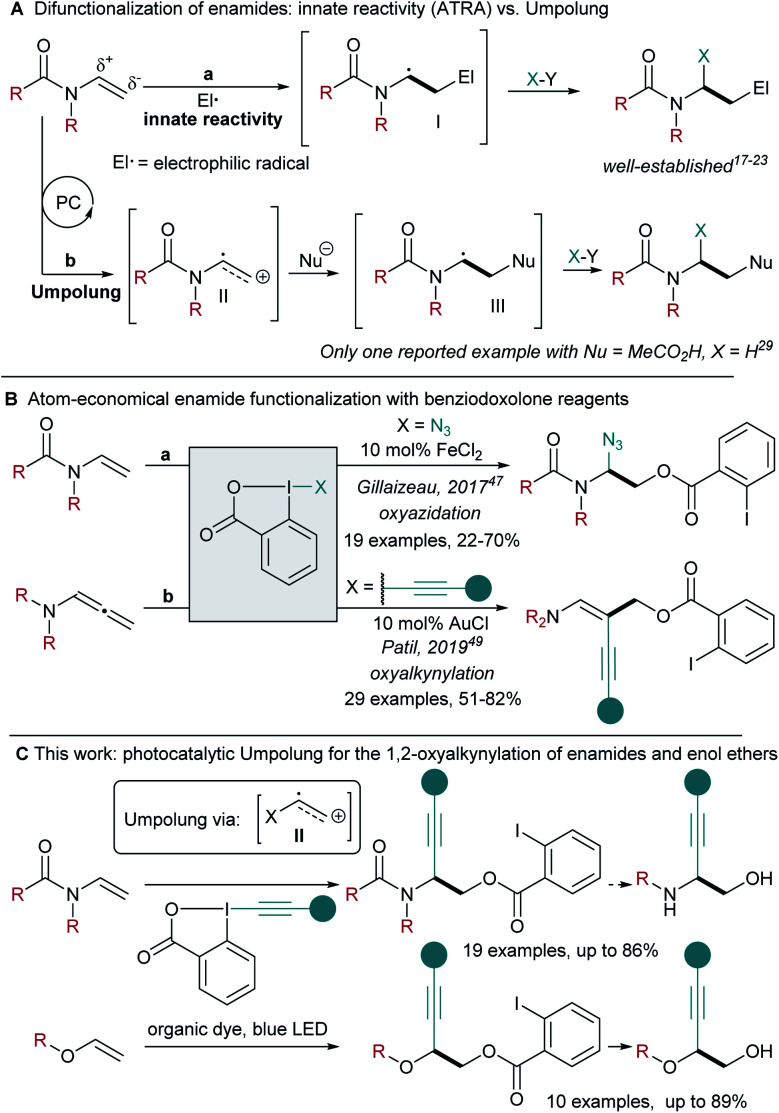
(A) Difunctionalization of enamides: innate reactivity *vs.* Umpolung. (B) Atom-economical enamide difunctionalization with benziodoxolone reagents. (C) This work: photocatalytic Umpolung enabling the synthesis of 1-alkynyl-1,2-amino alcohols and diols.

Enamides and ene-carbamates are versatile starting materials for the generation of complex aminated building blocks.^11–16^ In particular, they have been used extensively in atom transfer radical addition (ATRA) reactions.^17^ Due to their innate nucleophilicity, they are excellent traps for electrophilic radicals, leading to the formation of a nucleophilic α-amino radical **I** (Scheme 1A, a). The latter can then react with a radical trap,^18^ undergo oxidation to the α-amino cation,^19–21^ reduction to the α-amino anion,^22^ or addition to an organometallic species followed by reductive elimination.^23^ Despite the efficiency associated to such transformations, all enamide difunctionalizations reported so far are based on the initial addition of a highly reactive electrophilic radical, limiting functional group tolerance and the structural diversity of the obtained products.

Nicewicz and co-workers developed a different approach towards alkene difunctionalization based on oxidation under photoredox conditions for the generation of radical cations.^24–26^ This highly electrophilic species can then react with various nucleophiles, enabling new types of hydrofunctionalizations.^27–32^

In the case of enamides or ene-carbamates, such a strategy would result in a neat Umpolung of the reactivity (Scheme 1A, b). It is important to stress that such an approach would completely change the type of transformations accessible, as the first step would involve reaction with a nucleophile, in opposition to the electrophilic radical already intensively investigated.^17–23^ Although this strategy appears highly attractive to answer current limitations in enamide functionalization, only one example of ene-carbamate hydroacetoxylation has been reported by Nicewicz and co-workers.^29^ When considering the importance of nitrogen-containing compounds, a difunctionalization of enamides *via* photocatalytic Umpolung would be highly desirable.

In order to develop such a process, we turned to Ethynyl BenziodoXolone (EBX) hypervalent iodine reagents, which have been identified as efficient traps for radicals.^33–37^ Their application in radical-mediated olefin alkynylation has also been explored.^38–42^ Recently, our group has exploited the nucleophilicity of the carboxylate group of EBX reagents in atom-economical reactions such as the 1,1-oxyalkynylation of diazo compounds and the ring-opening/oxyalkynylation of thiiranes.^43–46^ Therefore, EBX reagents appear ideally suited for the functionalization of radical cations due to their dual nucleophilic/somophilic nature. For what concerns atom economical enamide 1,2-difunctionalization with benziodoxole reagents, the Gillaizeau group has reported an iron-catalyzed enamide oxyazidation (Scheme 1B, a: X = N_3_) with Zhdankin's reagent.^47,48^ This reaction was proposed to occur *via* a classical ATRA mechanism. The Patil group reported a gold catalysed 1,2-oxyalkynylation of allenenamides with EBXs (Scheme 1B, b, X = alkynyl), involving both redox and π-activation by the gold catalyst.^49^ Consequently, 1,2-oxyalkynylation remains limited to allenenamides as substrates and photocatalytically generated radical cations have never been intercepted with EBX reagents.^50^

Herein, we show that ene-carbamate radical cations can be generated under oxidative photoredox conditions using 4-CzIPN-derived organic dyes.^51–53^ The formed intermediates react with Umpolung of the reactivity in an atom-economical fashion with EBX reagents acting as both *O*-nucleophile and alkynylating radical trap sources (Scheme 1C). This methodology could then be extended to commercially available enamides and enol ethers. The mild oxidative conditions allowed selective reaction of electron-rich alkenes in presence of non-activated ones. This procedure provides easy access to orthogonally protected 1-alkynyl-1,2-amino alcohols and diols, setting the foundations for the development of further difunctionalizations of electron-rich olefins *via* radical cation intermediates.

## 2. Results and discussions

Based on previous reports for enamide difunctionalization and α-amino radical alkynylation,^18^ we started our investigations with *N*-vinyloxazolidinone (**1a**)^54^ and Ph-EBX (**2**)^55^ (Table 1). The oxidation potential of **1a** was determined to be +1.30 V *vs.* SCE by cyclic voltammetry. Based on this result, we selected three organic photocatalysts (PC) for their oxidative properties in the excited state: 4-CzIPN (4a*/**4a˙−**: +1.35 V *vs.* SCE), 4-ClCzIPN (4b*/**4b˙−**: +1.58 V) and Mes-Acr^+^ (5+*/**5˙**: +2.06 V).^27,29^ Using DCE as a solvent and 1.5 equivalents of alkene both **4a** and **4b** enabled product formation (entries 1 and 2). **4b** gave a promising 42% yield of the desired compound **3a** (entry 2). Highly oxidizing **5** resulted in a 5% yield (entry 3). The yield obtained was dependent on the batch of benziodoxolone **2** when using photocatalyst **4b** (entry 4). With a recrystallized batch (as opposed to one purified by trituration only)^55,56^ the yields were reproducible yet low (entry 5). We speculated that an impurity from the triturated batch was affecting the reactivity. As the most probable impurities were iodine(iii) precursors, hydroxy and acetoxy benziodoxolones were examined as additives (BIOH, **6** and BIOAc, **7**):^57^ adding **6** (1.5 equiv., entry 6) improved slightly the yield. With **7** (1.5 equiv., entry 7), the yield increased to 70%. With 0.5 equivalent of **7**, the yield remained in the same range (75%, entry 8).^58^ Both DMSO and DCM could also be used as solvents (entries 9 and 10).^59^

**Table 1:** Optimization of the oxyalkynylation of ene-carbamate **1a**^a^

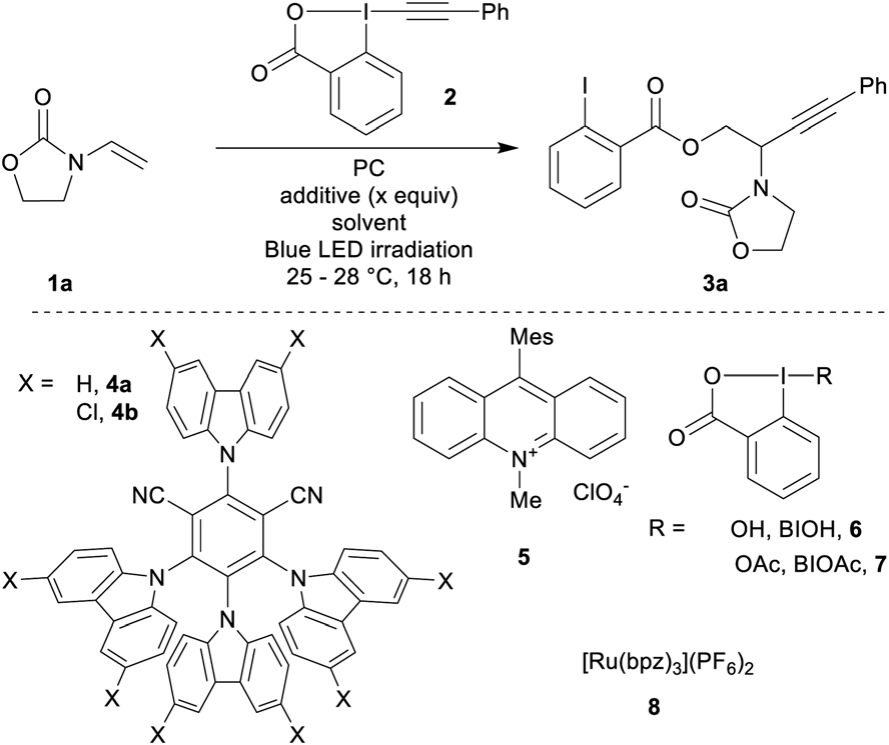				
Entry	PC	Additive (*x* equiv.)	Solvent	Yield^b^ (%)
1	**4a**	None	DCE	30
2	**4b**	None	DCE	42
3	**5**	None	DCE	5
4	**4b**	None	DCE	36–65
5^c^	**4b**	None	DCE	34
6^c^	**4b**	BIOH (**6**, 1.5)	DCE	46
7^c^	**4b**	BIOAc (**7**, 1.5)	DCE	70
8^c^	**4b**	BIOAc (**7**, 0.5)	DCE	75
9^c^	**4b**	BIOAc (**7**, 0.5)	DMSO	75
10^c^	**4b**	BIOAc (**7**, 0.5)	DCM	80
11^c^	**4b** d	BIOAc (**7**, 0.5)	DCM^e^	80
12^c^	**8** d	BIOAc (**7**, 0.5)	DCM^e^	21

a Reactions conditions: 0.05 mmol **2** (1 equiv.), **1a** (1.5 equiv.), additive (*x* equiv.) and PC (5 mol%) in solvent (0.1 M) unless specified otherwise. Blue led irradiation for 18 h at rt.

b ^1^H NMR yield determined by addition of 0.05 mmol of CH_2_Br_2_ as an internal standard after the reaction.

c Recrystallized **2**.

d 2 mol%.

e Concentration based on **2**: 0.25 M, at 0.2 mmol scale.

Final adjustments were made on scope scale (0.2 mmol): DCM was used as a solvent with a lower catalyst loading of 2 mol% and an increase of the concentration to 0.25 M. This gave product **3a** in 80% yield (entry 11). Finally, we tested ruthenium based photocatalyst **8**, which has a comparable oxidation potential (Ru^2+^*/Ru^+^: +1.40 V): **3a** was only obtained in 21% yield (entry 12). This result may have its origin from the weaker reduction potential of **8** (Ru^2+^/Ru^+^: −0.80 V), compared to **4b** (**4b**/**4b˙−**: −1.10 V).

**Scheme 2:**
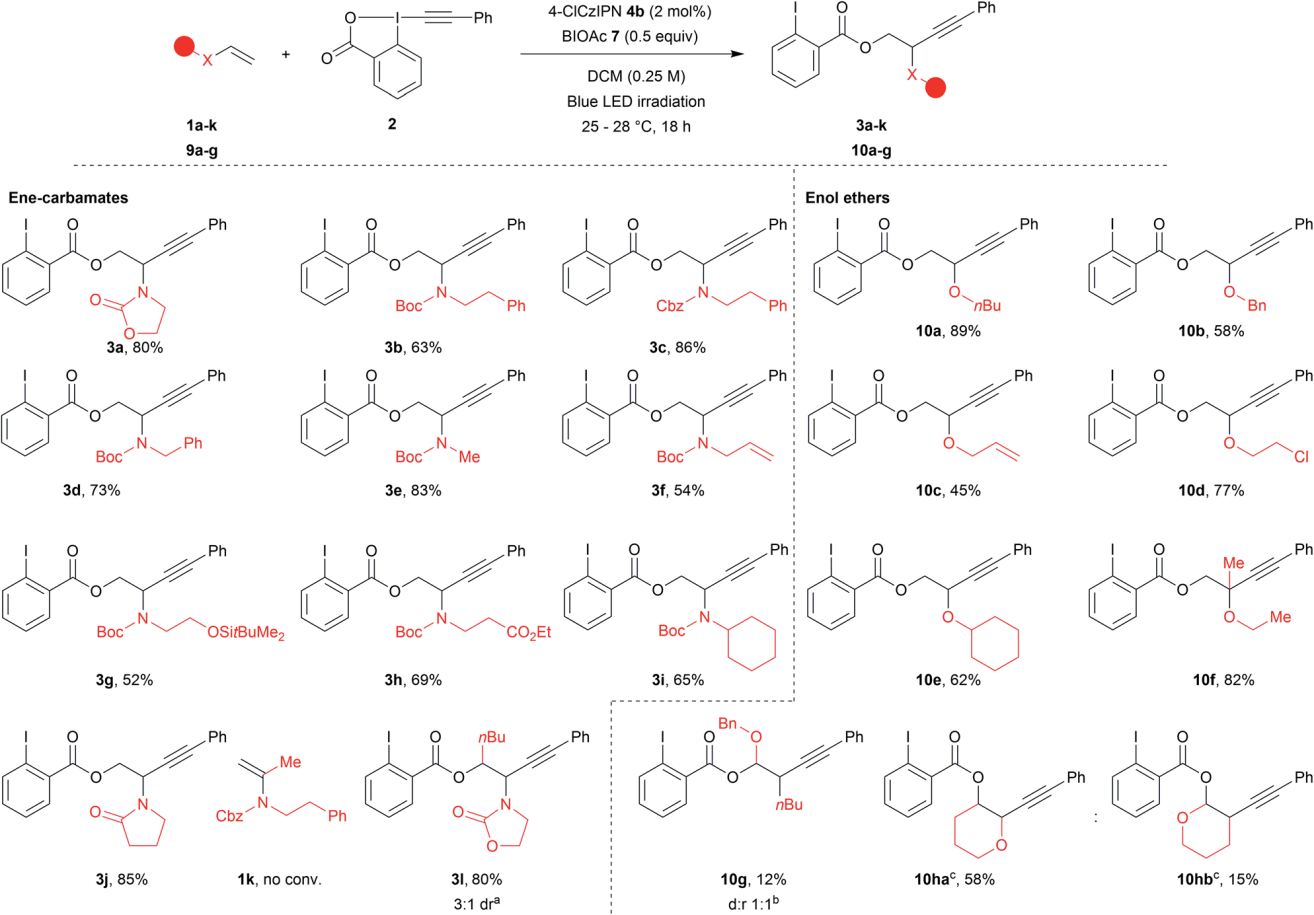
Scope of ene-carbamates and enol ethers. Reactions conditions: 0.20–0.25 mmol **2** (1 equiv.), alkene (1.2–1.5 equiv.), BIOAc (**7**, 0.5 equiv.) and **4b** (2 mol%) in DCM 0.25 M. Blue led irradiation for 18 h at rt. ^*a*^ Isolated ratio. ^*b* 1^H NMR ratio. ^*c*^**10ha** and **10hb** were obtained as an inseparable mixture of regioisomers from **9i**.

With the optimized reaction conditions in hand, we explored the scope of the reaction (Scheme 2). Acyclic ene-carbamates were tolerated affording Boc and Cbz protected amines **3b** and **3c** in 63% and 86% yield. Although N–H vinyl carbamates degraded under the reaction conditions, the orthogonally diprotected ene-carbamate **1d** was converted to **3d** in 73% yield. A methyl amine worked well under our reaction conditions (**3e**, 83% yield). An allyl amine was also tolerated affording **3f** in 54% yield,^60^ demonstrating that selective functionalization of ene-carbamates over alkenes was possible. Substrates bearing a silylated alcohol and an ethyl ester yielded the desired compounds **3g** and **3h** in 52% and 69% yield. The procedure also worked with secondary amines (**3i**, 65% yield). Commercial *N*-vinylpyrrolidinone gave compound **3j** in 85% yield. α-Substitution of the alkene was not tolerated (**1k** no conversion), but β-substituted (*E*)-ene-carbamate afforded **3l** as a mixture of diastereoisomers in 80% yield (3 : 1 dr.).

Finally, we examined enolethers, which have comparable oxidation potentials (*e.g.* dihydropyran (DHP, **9i**), 1.51 V *vs.* SCE). Aliphatic (**10a**), benzylic (**10b**) and allylic (**10c**) ethers were obtained in 89%, 58% and 45% yield. Chlorinated product **10d** was obtained in 77% yield. A secondary enol ether afforded **10e** in 62% yield. Although α-substituted ene-carbamates were not tolerated (**1k**, no conv.), tertiary ether **10f** was obtained from propen-2-yl enol ether in 82% yield. β-Substitution afforded a mixture of compounds, from which acetal **10g** corresponding to Markovnikov addition could be isolated in 13% yield.^61^ Finally, DHP **9h**^62^ afforded two regioisomers: anti-Markovnikov product **10ha** (58% yield) and Markovnikov product **10hb** (15% yield).^63^

**Scheme 3:**
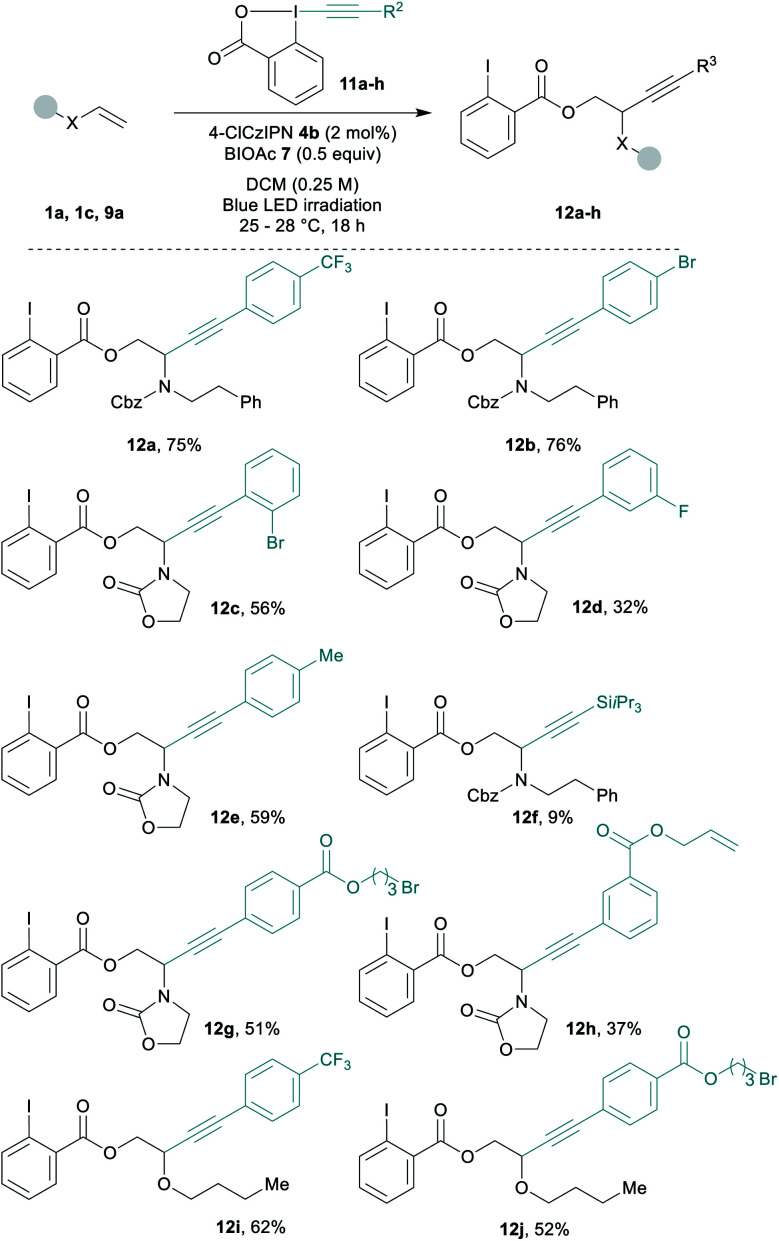
Scope of EBX reagents. Reactions conditions: 0.25 mmol **11a–h** (1 equiv.), **1a** or **3a** (1.5 equiv.), BIOAc (**7**, 0.5 equiv.) and **4b** (2 mol%) in DCM 0.25 M. Blue led irradiation for 18 h at rt.

Diverse EBX reagents were then examined (Scheme 3). Both electron-poor and electron-rich arenes on the alkyne provided the desired compounds **12a–12e** in up to 76% yield. The transfer of a silyl protected alkyne was less efficient and product **12f** was obtained in 9% yield only.^64^ EBXs bearing sensitive functionalities such as an alkyl bromide or a terminal alkene gave the corresponding products **12g** and **12h** in 51% and 37% yield. Functionalized EBXs could also be used with enol ether **9a** affording **12i** and **12j** in 62% and 52% yield.

**Scheme 4:**
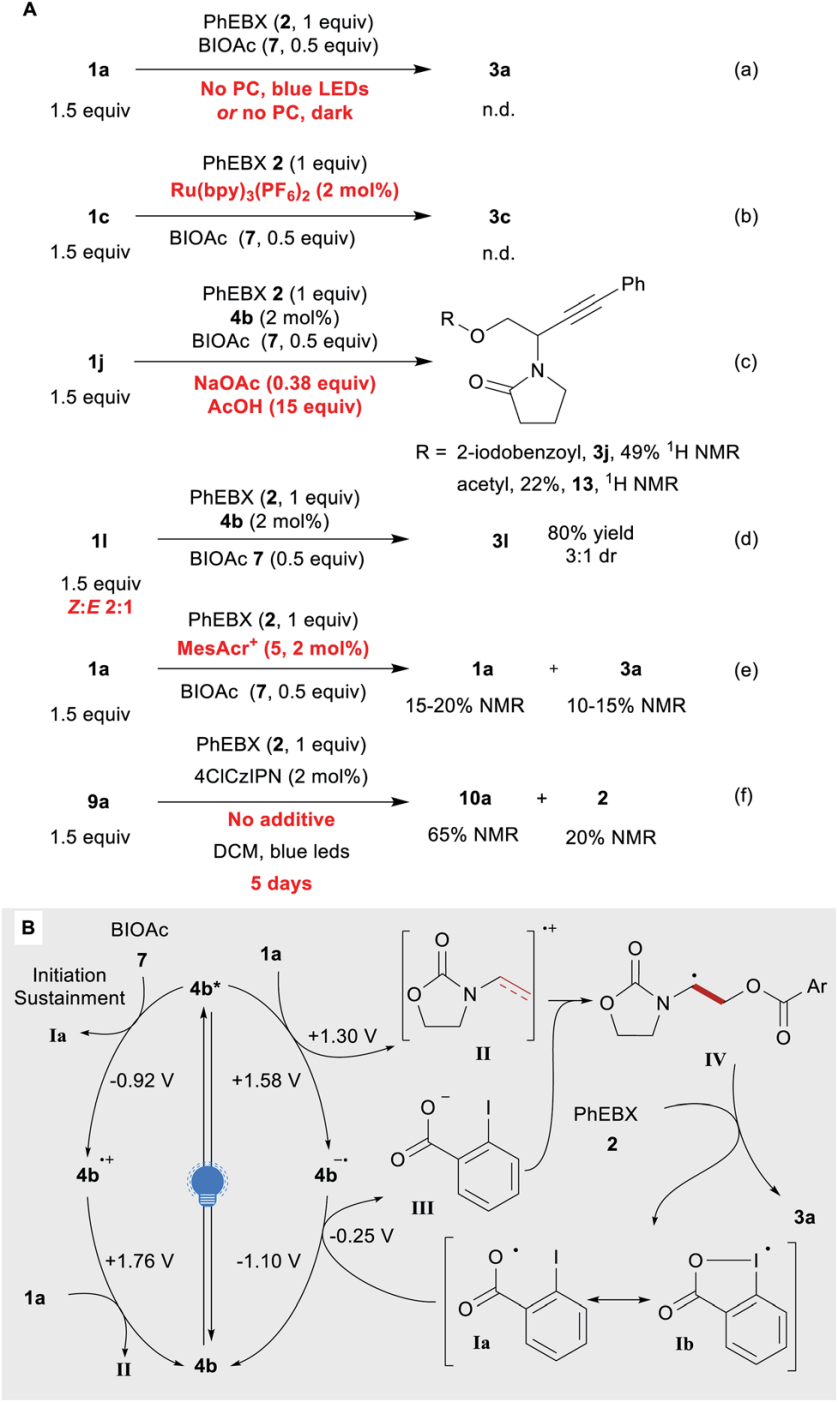
Mechanistic studies. (A) Control experiments Reaction conditions: 0.05–0.25 mmol **2** (1 equiv.), **1** (1.5 equiv.), additive (*x* equiv.), PC (2 mol%), DCE or DCM (0.25 M). (B) Proposed mechanism.

We then investigated the mechanism of the reaction. Without the photocatalyst and/or a light source, no product was detected (Scheme 4A, eqn (a)). We then considered the possibility of acyloxyl radical **Ia** (Scheme 4B) adding to the electron-rich olefin **1a**. Previously, **Ia** (a resonance structure of the iodanyl radical **Ib**) has been reported predominantly as a H-atom abstractor.^65–67^ To the best of our knowledge, the only proposed report of **Ia** adding to an alkene is that of the Gillaizeau group.^47^

Chen and co-workers reported the generation of **Ia** through the reduction of BIOAc (**7**) by excited Ru(bpy)_3_^2+^.^57^ With this photocatalyst, we observed no conversion or product formation (eqn (b)). This suggests that the generation of **Ia** alone does not lead to product formation. In addition, no conversion was observed with substrate **1k**, which is tougher to oxidize (**1k˙+**/**1k** ≈ 1.86 V) (Scheme 2). Some conversion would have been expected if the reaction proceeded through the addition of oxygen-centred radical **Ia**. β-Substituted alkene **1l** gave product **3l** in 80% yield with the same efficiency as for terminal enamide **1a**. An ATRA process would have been more significantly impaired by the substituent. The observed anti-Markovnikov selectivity is in agreement with the reactivity reported for radical cations.^26^ In order to test our hypothesis, we performed the reaction under the standard conditions in presence of sodium acetate and acetic acid (eqn (c)). The acetate could indeed be introduced (compound **13**), but EBX-addition product **3j** remains the main product (2 : 1 ratio). When the reaction was performed with a 2 : 1 ratio of *Z* and *E* isomers of **1l** instead of pure *E* compound, no change in yield and diastereoselectivity was observed, supporting the presence of a radical intermediate (eqn (d)). With the strongly oxidizing catalyst **5**, low yields were observed even in presence of BIOAc (**7**), despite almost full conversion of **1a** (eqn (e)). Based on these results, we propose a tentative mechanism for the oxyalkynylation (Scheme 4B). First, the excited photocatalyst 4b* oxidizes **1a** generating radical cation **II** and reduced catalyst **4b˙−**. As support for this step, quenching of fluorescence of catalyst **4b** by **1a** was observed in a Stern–Volmer experiment (see ESI‡ for details).^68^ Then **II** is trapped by carboxylate **III**. This results in the formation of radical **IV**, which can add to **2** affording the product and iodanyl radical **Ib**. The latter can close the catalytic cycle by oxidizing **4b˙−** to regenerate catalyst **4b**.^69^ We suspect that BIOAc (**7**) serves as initiator for the reaction by generating **Ia***via* reduction of BIOAc (**7**) with 4b*. The resulting oxidized catalyst **4b˙+** would be also competent to oxidize **1a**. This pathway would also help sustaining the catalytic cycle by ensuring a sufficient concentration of **Ia**. A final control experiment corroborates this hypothesis: the reaction was performed with no additive (eqn (f)). The desired compound was obtained in 65% ^1^H NMR yield after 5 days of reaction time with 20% residual PhEBX (**2**).

**Scheme 5:**
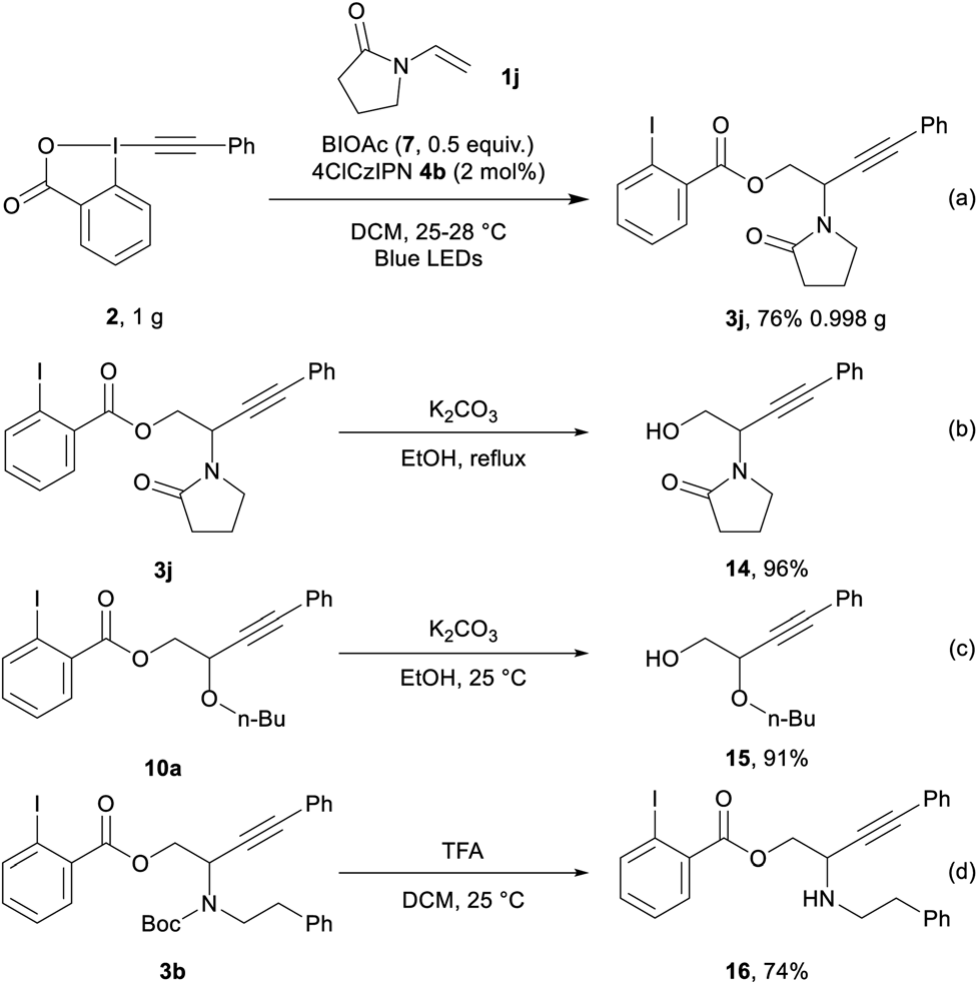
Gram-scale reaction and post-functionalization.

We then performed the transformation on gram scale (Scheme 5, eqn (a)), affording **3j** in 76% yield (0.998 g). Selective hydrolysis of the ester group from **3j** gave **14** in 96% yield (eqn (b)). Hydrolysis of **10a** provided **15** in 91% yield (eqn (c)). Finally, **3b** underwent Boc deprotection to give amino ester **16** in 74% yield (eqn (d)).

## 3. Conclusions

In conclusion, we have developed a photocatalytic 1,2-oxy-alkynylation of ene-carbamates based on Umpolung of the reactivity. The transformation proceeds in an atom-economical fashion with EBXs acting both as alkynylating and carboxylating reagents. The reaction occurs at room temperature under blue LED irradiation using 4-ClCzIPN (**4b**) as an organic photocatalyst and does not require the use of highly reactive electrophilic radicals. The methodology could be extended to enamides and enolethers. The method shows good chemoselectivity for nitrogen or oxygen-substituted olefins over aliphatic alkenes. Based on preliminary mechanistic studies, we propose that an ene-carbamate radical cation is the key intermediate that ensures the anti-Markovnikov regioselectivity initiated by nucleophile addition, contrasting with the classical ATRA mechanism usually invoked for the functionalization of alkenes with hypervalent iodine reagents. This reaction allows quick access to protected 1-alkynyl-1,2-amino alcohols and 1-alkynyl-1,2-diols, which are important building blocks in agrochemical, pharmaceutical and material sciences.

## Conflicts of interest

There are no conflicts to declare.

## Supplementary Material

SC-011-D0SC03655B-s001
