# Supplementary material for: Photocatalytic Umpolung of N- and O-substituted alkenes for the synthesis of 1,2-amino alcohols and diols
Source: Chem Sci. 2020 Sep 22;11(41):11274–9. doi: 10.1039/d0sc03655b (PMC8162387; doi:10.1039/d0sc03655b)

Electronic Supplementary Material (ESI) for Chemical Science.
This journal is © The Royal Society of Chemistry 2020

Supporting Information

Photocatalytic Umpolung of *N*- and *O*- Substituted Alkenes for the Synthesis of 1,2-Amino alcohols and Diols

Stephanie G. E. Amos, Stefano Nicolai, Jerome Waser*

Laboratory of Catalysis and Organic Synthesis, Institut des Sciences et Ingénierie Chimique, Ecole Polytechnique Fédérale de Lausanne, CH-1015, Lausanne, Switzerland

Raw data for NMR, MS and IR is available at: https://doi.org/10.5281/zenodo.4043189

Contents

1. General methods 3

2. Synthesis of the hypervalent iodine reagents 5

3. General procedure A: Synthesis of the photocatalysts 13

4. Synthesis of electron rich alkenes (**1a-k** and **9g**) 14

5. Ene-carbamate and enol-ether oxyalkynylation 28

Optimisation of the reaction conditions and control reactions: 28

Control expermients and mechanistic studies 29

Electrochemical experiments 32

Stern Volmer quenching expermients 34

Experimental set-up 36

General procedure for scope scale reactions: 37

Characterisation data 37

6. Gram scale synthesis and product modification 51

7. NMR spectra for synthesised alkenes and new compounds 54

Starting materials 54

Products 69

Product modification 106

1. General methods

All reactions that were carried out in oven dried glassware and under an atmosphere of nitrogen is stated at the start of the reaction conditions. For flash chromatography, distilled technical grade solvents were used. THF, CH_3CN, toluene, Et_2O and CH_2Cl_2 were dried by passage over activated alumina under nitrogen atmosphere (H_2O content < 10 ppm, Karl-Fischer titration). The solvents were degassed by Freeze-Pump-Thaw method when mentioned. All chemicals were purchased from Acros, Aldrich, Fluka, VWR, TCI, Merck and used as such unless stated otherwise. Chromatographic purification was performed as flash chromatography using Macherey-Nagel silica 40-63, 60 Å, using the solvents indicated as eluent with 0.1-0.5 bar pressure. TLC was performed on Merck silica gel 60 F254 TLC glass plates and visualized with UV light and *p*-anisaldehyde stain (EtOH:H_2SO_4:AcOH:*p*-anisaldehyde 135:5:1.5:3.7 V:V:V:V).

^{1}H-NMR spectra were recorded on a Brucker DPX-400 400 MHz spectrometer in chloroform-*d*, acetonitrile-d_3, DMSO-d_6 or acetone-d_6, all signals Are reported in ppm with the internal chloroform signal at 7.26 ppm, the internal acetonitrile signal at 1.94 ppm, the internal methanol signal at 3.30 ppm, the internal DMSO signal at 2.50 ppm or the internal acetone signal at 2.05 ppm as standard. The data is reported as (s = singlet, d = doublet, t= triplet, q = quadruplet, qi = quintet, m = multiplet or unresolved, br = broad signal, app = apparent, coupling constant(s) in Hz, integration, interpretation). ^{13}C-NMR spectra were recorded with ^{1}H-*d*ecoupling on a Brucker DPX-400 100 MHz spectrometer in chloroform-*d*, acetonitrile-d^3 CD_3OD, DMSO-d^6 or acetone-d^6, all signals Are reported in ppm with the internal chloroform signal at 77.0 ppm, the internal acetonitrile signal at 1.3 ppm the internal methanol signal at 49.0 ppm, the internal DMSO signal at 39.5 ppm or the internal acetone signals at 29.84 and 206.26 ppm as standard. Rotameric mixtures have been described at room temperature as a mixture of rotamers, only the split signals have been assigned to the major or minor rotamer. Regiomeric mixtures have been assigned based on the shift of the characteristic proton signals. Diastereoiomers have been separated when possible if not assigned based on ^{1}H NMR analysis.

Infrared spectra were recorded on a JASCO FT-IR B4100 spectrophotometer with an ATR PRO410-S and a ZnSe prisma and is reported in cm^{-1} (w = weak, m = medium, s = strong, br = broad).

High resolution mass spectrometric measurements were performed by the mass spectrometry service of ISIC at the EPFL on a MICROMASS (ESI) Q-TOF Ultima API.

All photoredox catalyzed reactions were carried out in oven dried glassware and under inert atmosphere (freeze pump thaw solvent stored on molecular sieves and under argon for maximum one week) unless specified otherwise. They were performed in test tubes (5 and 10 mL) which were held using a rack for test tubes placed at the center of a crystallization dish or screw cap vials (0.5 – 10 mL) which were stuck to the base of the crystallization dish. In order to keep the temperature as constant as possible all reactions were ventilated by use of an over-head ventilator (desk fan). To the crystallization dish (a straight sided 15 cm diameter pyrex dish) were attached the blue LEDs (RUBAN LED 5MÈTRES - 60LED/M - 3528 BLEU - IP65

with Transformateur pour Ruban LED 24W/2A/12V, bought directly on RubanLED.com). The distance between the LEDs and the test tubes was approximatively 3 cm for all vials and test tubes. Long irradiation resulted in temperature increasing up to 27 °C during overnight reactions. Photos have been provided.

UV/Vis spectroscopy was performed on an Agilent Cary 60 UV-Vis and steady-state luminescence spectroscopy was recorded on a Varian Cary Eclipse spectrophotometer. Cyclic voltammetry experiments were performed on a Biologic SP-150 Potentiostat, with a three-electrode cell configuration: a glassy carbon electrode as the working electrode, Pt wire as a counter electrode and an Ag/AgCl (KCl, 3M) electrode as the reference electrode. Bu_4NPF_6 was employed as the electrolyte (0.1 M).

Synthesis of the hypervalent iodine reagents

The synthesis of reagents **2**, and **11a-11f** had already been described before by our group. The procedures are taken from the indicated publications to facilitate reproduction of the results by having all data in the same file.

1-Hydroxy-1,2-benziodoxol-3-(1*H*)-one (**6**)

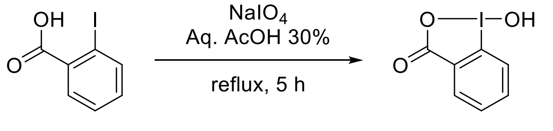

Following a reported procedure,[1] $NaIO_4$ (40.5 g, 189 mmol, 1.05 equiv) and 2-iodobenzoic acid (**17**) (44.8 g, 180 mmol, 1.0 equiv) were suspended in 30% (v:v) aq. AcOH (350 mL). The mixture was vigorously stirred and refluxed for 5 h. The reaction mixture was then diluted with cold water (250 mL) and allowed to cool to rt, protecting it from light. After 1 h, the crude product was collected by filtration, washed on the filter with ice water (3 x 150 mL) and acetone (3 x 150 mL), and air-*d*ried in the dark overnight to afford 1-Hydroxy-1,2-benziodoxol-3-(1*H*)-one (**6**) (44.3 g, 168 mmol, 93% yield) as a white solid.

^{1}H NMR (400 MHz, DMSO-d_6) δ 8.02 (dd, J = 7.7, 1.4 Hz, 1H, Ar*H*), 7.97 (m, 1H, Ar*H*), 7.85 (dd, J = 8.2, 0.7 Hz, 1H, Ar*H*), 7.71 (td, J = 7.6, 1.2 Hz, 1H, Ar*H*). **^{13}C NMR** (100 MHz, DMSO-d_6) δ 167.7, 134.5, 131.5, 131.1, 130.4, 126.3, 120.4. Consistent with reported data.[1]

1-Acetoxy-1,2-benziodoxol-3-(1H)-one (**7**)

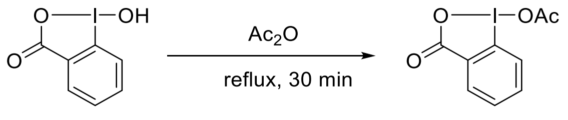

Following a reported procedure,[2] compound **6** (3.00 g, 11.3 mmol, 1.00 equiv) was heated in Ac_2O (10 mL) to reflux until the solution turned clear (without suspension, ca. 30 min). The mixture was then left to cool down and white crystals started to form. The crystallization was continued at -18 °C. The crystals were then collected and dried overnight under high vacuum to give compound **7** (3.06 g, 10.0 mmol, 86% yield).

^{1}H NMR (400 MHz, Chloroform-d_3) δ 8.25 (dd, 1 H, J = 7.6, 1.4 Hz, Ar*H*), 8.00 (dd, 1 H, J = 8.3, 0.5 Hz, Ar*H*), 7.92 (dt, 1 H, J = 7.0, 1.7 Hz, Ar*H*), 7.71 (td, 1 H, J = 7.6, 0.9 Hz, Ar*H*), 2.25 (s, 3 H, COCH_3). NMR data correspond to the reported values.[2]

[1] Brand, J. P.; Chevalley, C.; Scopelliti, R.; Waser, J. *Chem. Eur. J.* **2012**, *18*, 5655.
[2] Eisenberger, P.; Gischig, S.; Togni, A. *Chem. Eur. J.* **2006**, *12*, 2579.

1-[Phenylethynyl]-1,2-benziodoxol-3(1H)-one (PhEBX, **2**)

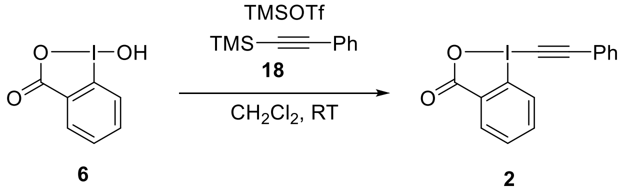

Following a reported procedure,[1] trimethylsilyltriflate (9.1 mL, 50 mmol, 1.1 equiv) was added dropwise to a suspension of 2-iodosylbenzoic acid (**6**) (12.1 g, 45.8 mmol, 1.0 equiv) in CH_2Cl_2 (120 mL) at 0 °C. The mixture was stirred for 1 h, followed by the dropwise addition of trimethyl(phenylethynyl)silane (**18**) (8.8 mL, 50 mmol, 1.1 equiv) (slightly exothermic). The resulting suspension was stirred for 6 h at RT, during this time a white solid was formed. A saturated solution of $NaHCO_3$ (120 mL) was added and the mixture was stirred vigorously for 30 min. The resulting suspension was filtered on a glass filter. The two layers of the mother liquors were separated and the organic layer was washed with sat. $NaHCO_3$ (2x50 mL), dried over $MgSO_4$, filtered and evaporated under reduced pressure. The resulting mixture was combined with the solid obtained by recrystallisation in EtOAc:MeOH (2:1, ca. 28 mL/g). The mixture was cooled down, filtered and dried under high vacuum to afford Ph-EBX (**2**) (6.8 g, 25 mmol, 43% yield) as colorless crystals.

Mp (Dec.) 155 – 160 °C. **^{1}H NMR** (400 MHz, Chloroform-*d*) δ 8.46 (m, 1H, Ar*H*), 8.28 (m, 1H, Ar*H*), 7.80 (m, 2H, Ar*H*), 7.63 (m, 2H, Ar*H*), 7.48 (m, 3H, Ar*H*). **^{13}C NMR** (101 MHz, Chloroform-*d*) δ 163.9, 134.9, 132.9, 132.5, 131.6, 131.3. 130.8, 128.8, 126.2, 120.5, 116.2, 106.6, 50.2. Consistent with reported data.[1]

1-[4-Trifluoromethylphenylethynyl]-1,2-benziodoxol-3(1H)-one (**11a**)

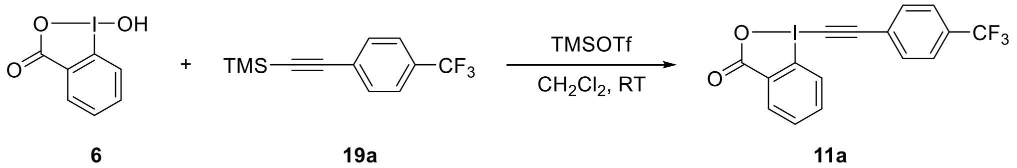

Following a reported procedure,[3] trimethylsilyl triflate (1.0 mL, 5.5 mmol, 1.1 equiv) was added to a suspension of 2-iodosylbenzoic acid (**6**) (1.3 g, 5.0 mmol, 1.0 equiv) in CH_2Cl_2 (15 mL) at RT The resulting suspension was stirred for 1 h, followed by the dropwise addition of trimethyl((4-(trifluoromethyl)phenyl)ethynyl)silane (**19a**) (1.3 mL, 5.5 mmol, 1.1 equiv), which was dissolved in CH_2Cl_2 (1 mL). The resulting suspension was stirred for 6 h at RT A saturated solution of $NaHCO_3$ (20 mL) was then added and the mixture was stirred vigorously for 30 min, the two layers were separated and the organic layer was washed with sat. $NaHCO_3$ (20 mL), dried over $MgSO_4$, filtered and evaporated under reduced pressure. The resulting solid was boiled in CH_3CN (20 mL). The mixture was cooled down, filtered and dried under high vacuum to afford **11a** (1.3 g, 3.2 mmol, 64% yield) as a pale yellow solid.

---

[3] Lu, B.; Wu, J.; Yoshikai, N. *J. Am. Chem. Soc.* **2014**, *136*, 11598.

^{1}H NMR (400 MHz, Chloroform-*d*) δ 8.46 – 8.38 (m, 1H, Ar*H*), 8.28 – 8.19 (m, 1H, Ar*H*), 7.84 – 7.74 (m, 2H, Ar*H*), 7.74 – 7.65 (m, 4H, Ar*H*). **^{13}C NMR** (101 MHz, Chloroform-*d*) δ 166.6, 135.0, 133.0, 132.6, 132.2 (q, *J* = 33.0 Hz), 131.7, 131.2, 126.3, 125.7 (q, *J* = 3.6 Hz), 124.4, 123.4 (q, *J* = 272.6 Hz), 116.1, 104.2, 53.7. Consistent with reported data.[3]

1-[4-Bromophenylethynyl]-1,2-benziodoxol-3(1H)-one (**11b**)

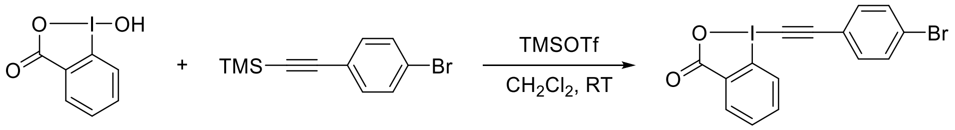

6 **19b** **11b**

Following a reported procedure,[4] trimethylsilyl triflate (1.0 mL, 5.5 mmol, 1.1 equiv) was added to a suspension of 2-iodosylbenzoic acid (**6**) (1.3 g, 5.0 mmol, 1.0 equiv) in CH_2Cl_2 (15 mL) at RT The resulting suspension was stirred for 1 h, followed by the dropwise addition of ((4-bromophenyl)ethynyl)trimethylsilane (**19b**) (1.2 g, 5.5 mmol, 1.1 equiv), which was dissolved in CH_2Cl_2 (1 mL). The resulting suspension was stirred for 6 h at RT A saturated solution of $NaHCO_3$ (20 mL) was then added and the mixture was stirred vigorously for 30 min, the two layers were separated and the organic layer was washed with sat. $NaHCO_3$ (20 mL), dried over $MgSO_4$, filtered and evaporated under reduced pressure. The resulting solid was boiled in CH_3CN (20 mL). The mixture was cooled down, filtered and dried under high vacuum to afford **11b** (1.4 g, 3.3 mmol, 66% yield) as a pale yellow solid.

Mp 158-163 °C (decomposition). **^{1}H NMR** (400 MHz, Chloroform-*d*) δ 8.51 – 8.30 (m, 1H, Ar*H*), 8.30 – 8.13 (m, 1H, Ar*H*), 7.84 – 7.72 (m, 2H, Ar*H*), 7.58 (d, 2H, *J* = 8.5 Hz, Ar*H*), 7.46 (d, 2 H, *J* = 8.5 Hz, Ar*H*). **^{13}C NMR** (101 MHz, Chloroform-*d*) δ 166.6, 135.1, 134.3, 132.7, 132.3, 131.9, 131.4, 126.3, 125.7, 119.6, 116.3, 105.4, 52.1. Consistent with reported data.[6]

1-[2-Bromophenylethynyl]-1,2-benziodoxol-3(1*H*)-one (**11c**)

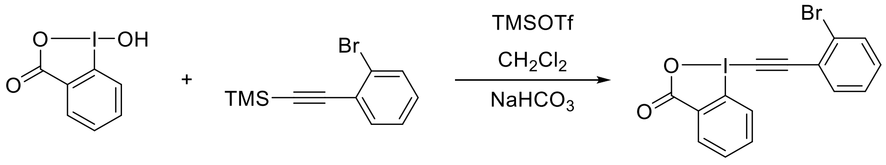

6 **19c** **11c**

Following a reported procedure,[5] trimethylsilyl triflate (1.0 mL, 5.5 mmol, 1.1 equiv) was added to a suspension of 2-iodosylbenzoic acid (**6**) (1.32 g, 5.00 mmol, 1.00 equiv) in CH_2Cl_2 (15 mL) at RT The resulting suspension was stirred for 3 h, followed by the drop wise addition of ((2-bromophenyl)ethynyl)trimethylsilane (**19c**) (1.17 g, 5.50 mmol, 1.10 equiv). The resulting suspension was stirred for 6 h at RT A saturated solution of $NaHCO_3$ (20 mL) was then added and the mixture was stirred vigorously for 30 minutes, the two layers were separated and the organic layer was washed with sat. $NaHCO_3$ (20 mL), dried over $MgSO_4$, filtered and evaporated under reduced pressure. The resulting solid was boiled in CH_3CN (20 mL). The mixture was

[4] Jia, K.; Zhang, F.; Huang, H.; Chen, Y. *J. Am. Chem. Soc* **2016**, *138*, 1514.
[5] Le Vaillant, F. ; Courant, T. ; Waser, J. *Angew. Chem. Int. Ed.* **2015**, *54*, 11200.

cooled down, filtered and the collected solid was dried under high vacuum to afford **11c** (1.50 g, 3.51 mmol, 70% yield) as a colorless solid.

^{1}H NMR (400 MHz, Chloroform-*d*) δ 8.44 (td, *J* = 7.3, 2.1 Hz, 2 H, Ar*H*), 7.84 – 7.74 (m, 2 H, Ar*H*), 7.68 (d, *J* = 1.1 Hz, 1 H, Ar*H*), 7.61 (dd, *J* = 7.6, 1.7 Hz, 1 H,*ArH*), 7.36 (m, 2 H, Ar*H*).**^{13}C NMR** (101 MHz, Chloroform-*d*)[7] δ 166.6, 135.2, 134.7, 133.0, 132.7 , 131.8, 131.3, 127.6, 126.8, 126.4, 123.2, 116.5, 104.3, 55.4. Consistent with reported data.[6]

1-[3-Fluorophenylethynyl]-1,2-benziodoxol-3(1H)-one (**11d**)

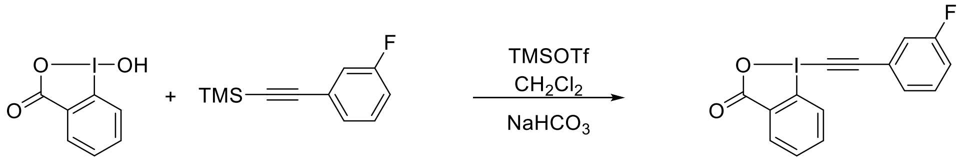

6 **19d** **11d**

Following a reported procedure,[6] trimethylsilyl triflate (1.0 mL, 5.5 mmol, 1.1 equiv) was added to a suspension of 2-iodosylbenzoic acid (**6**) (1.32 g, 5.00 mmol, 1.00 equiv) in CH_2Cl_2 (15 mL) at RT The resulting suspension was stirred for 1 h, followed by the drop wise addition of ((3-fluorophenyl)ethynyl)trimethylsilane (**19d**) (1.1 mL, 5.5 mmol, 1.1 equiv). The resulting suspension was stirred for 6 h at RT A saturated solution of $NaHCO_3$ (20 mL) was then added and the mixture was stirred vigorously for 30 minutes, the two layers were separated and the organic layer was washed with sat. $NaHCO_3$ (20 mL), dried over $MgSO_4$, filtered and evaporated under reduced pressure. The resulting solid was boiled in CH_3CN (20 mL). The mixture was cooled down, filtered and the collected solid was dried under high vacuum to afford **11d** (787 mg, 2.15 mmol, 43% yield) as a colorless solid.

^{1}H NMR (400 MHz, DMSO-*d*$_6$) δ 8.33 (dd, *J* = 8.2, 0.8 Hz, 1H, Ar*H*), 8.13 (dd, *J* = 7.4, 1.7 Hz, 1H, Ar*H*), 7.91 (ddd, *J* = 8.2, 7.2, 1.7 Hz, 1H, Ar*H*), 7.81 (td, *J* = 7.3, 0.9 Hz, 1H, Ar*H*), 7.64 – 7.59 (m, 1H, Ar*H*), 7.58 – 7.53 (m, 2H, Ar*H*), 7.47 – 7.37 (m, 1H, Ar*H*). **^{13}C NMR** (101 MHz, DMSO-*d*$_6$)[7] 166.3, 161.8 (d, *J* = 245.6 Hz), 135.3, 131.9, 131.3, 131.2 (d, *J* = 8.7 Hz), 129.0 (d, *J* = 2.9 Hz), 127.7, 122.4 (d, *J* = 9.6 Hz), 119.2 (d, *J* = 23.4 Hz), 118.1 (d, *J* = 21.1 Hz), 116.4, 102.5 (d, *J* = 3.3 Hz), 53.8. **^{19}F NMR** (376 MHz, DMSO-*d*$_6$) δ -111.7. Consistent with reported data.[5]

1-[4-Methylphenylethynyl]-1,2-benziodoxol-3(1*H*)-one (**11e**)

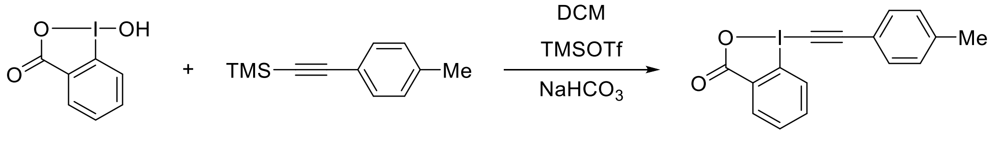

6 **19e** **11e**

Following a reported procedure,[8] trimethylsilyl triflate (1.0 mL, 5.5 mmol, 1.1 equiv) was added to a suspension of 2-iodosylbenzoic acid (**6**) (1.32 g, 5.00 mmol, 1.00 equiv) in CH_2Cl_2 (15 mL) at room temperature. The resulting suspension was stirred for 3 h, followed by the dropwise

---

[6] Le Vaillant, F. ; Garreau, M. ; Nicolai, S. ; Gryn'Ova, G. ; Corminboeuf, C. ; Waser, J. *Chem. Sci.* **2018**, *9*, 5883.

[7] One carbon is not resolved.

[8] Huang, H.; Zhang, G.; Gong, L.; Zhang, S.; Chen, Y. *J. Am. Chem. Soc.* **2014**, *136*, 2280.

addition of trimethyl(*p*-tolylethynyl)silane (**19e**) (1.04 g, 5.50 mmol, 1.10 equiv). The resulting suspension was stirred for 6 h at room temperature. A saturated solution of $NaHCO_3$ (20 mL) was then added and the mixture was stirred vigorously for 30 minutes, the two layers were separated and the organic layer was washed with saturated solution of $NaHCO_3$ (20 mL), dried over $MgSO_4$, filtered and evaporated under reduced pressure. The resulting solid was boiled in MeCN (ca 20 mL). The mixture was cooled down, filtered and dried under high vacuum to afford **11e** (0.540 g, 1.49 mmol, 30% yield) as a white solid.

^{1}H NMR (400 MHz, Chloroform-*d*): δ 8.43 (dd, *J* = 6.1, 2.9 Hz, 1H, Ar*H*), 8.30– 8.14 (m, 1H, Ar*H*), 7.77 (dd, *J* = 6.9, 3.1 Hz, 2H, Ar*H*), 7.50 (d, *J* = 7.8 Hz, 2H, Ar*H*), 7.25 (d, *J* = 7.6 Hz, 2H, Ar*H*), 2.43 (s, 3H, ArC*H*3); **^{13}C NMR** (100 MHz, Chloroform-*d*): δ 166.6, 141.5, 134.9, 132.8, 132.5, 131.6, 131.3, 129.5, 126.2, 117.4, 116.2, 107.25, 49.1, 21.7. The characterization data corresponded to the reported values.[8]

Tri*iso*propylsilyl trimethylsilylacetylene (**19f**)

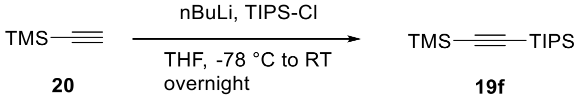

Following a reported procedure,[9] *n*-butyllithium (2.5 M in hexanes, 28 mL, 70 mmol, 0.98 equiv) was added dropwise to a stirred solution of ethynyltrimethylsilane (**20**) (7.0 g, 71 mmol, 1.0 equiv) in THF (100 mL) at -78 °C. The mixture was warmed to 0 °C and stirred for 5 min. The mixture was then cooled back to -78 °C and chlorotri*iso*propylsilane (15 mL, 71 mmol, 1.0 equiv) was added dropwise. The mixture was then allowed to warm to room temperature and stirred overnight. A saturated solution of ammonium chloride (100 mL) was added, and the reaction mixture was extracted with diethyl ether (2 x 100 mL). The combined organic layers were washed with water and brine, then dried over MgSO4, filtered and concentrated under reduced pressure to obtain a colorless liquid which was further purified by filtration on silica eluting with pentane (500 mL) to yield **19f** (16 g, 64 mmol, 90% yield) as a colorless liquid.

^{1}H NMR (400 MHz, Chloroform-*d*) *δ* 1.08 (m, 21H, TIPS), 0.18 (s, 9H, TMS). Consistent with reported data.[9]

1-[(Tri*iso*-propylsilyl)ethynyl]-1,2-benziodoxol-3(1*H*)-one (TIPS-EBX, **11f**)[10]

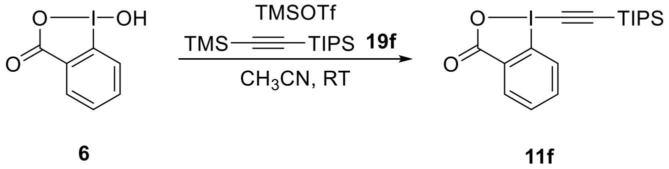

Following a reported procedure,[11] 2-iodosylbenzoic acid (**6**) (8.0 g, 30 mmol, 1.0 equiv) was charged in an oven-*d*ried round-bottomed 250 mL flask equipped with a magnetic stirrer. The

---

[9] Helal, C. J.; Magriotis, P. A.; Corey, E. J. *J. Am. Chem. Soc.* **1996**, *118*, 10938.
[10]In this methodology TIPS-EBX (**11f**) was obtained using its previous synthetic strategy however recently we have developed a one-pot proceedure: Hari, D. P.; Caramenti, P.; Schouwey, L.; Chang, M.; Nicolai, S.; Bachert, D.; Wright, T.; Orella, C.; Jerome Waser, J. *Org. Process Res. Dev.* **2020**, *24*, 106.
[11] Brand, J. P.; Waser, J. *Angew. Chem. Int. Ed.* **2010**, *49*, 7304.

solid was placed under a nitrogen atmosphere and anhydrous acetonitrile (100 mL) was added. The mixture was cooled to 0 °C. Trimethylsilyltriflate (6.0 mL, 33 mmol, 1.1 equiv) was added dropwise. After 15 min, (trimethylsilyl)(tri*iso*propylsilyl)acetylene (**19f**) (8.5 g, 33 mmol, 1.1 equiv) was added dropwise. After 30 min, the suspension became an orange solution. Pyridine (2.7 mL, 33 mmol, 1.1 equiv) was added dropwise. After 15 min, the reaction mixture was transferred in a one-neck 500 mL flask and concentrated under vacuum to afford a yellow solid. The solid was dissolved in CH_2Cl_2 (100 mL) and transferred in a 500 mL separatory funnel. The organic layer was washed with a 1 M HCl solution (50 mL) and the aqueous layer was extracted with CH_2Cl_2 (100 mL). The organic layers were combined, washed with a saturated solution of NaHCO3 (2 x 100 mL), dried over MgSO4, filtered and the solvent was evaporated under reduced pressure. Recrystallization from acetonitrile (40 mL) afforded TIPS-EBX (**11f**) (9.2 g, 21.5 mmol, 71% yield) as colorless crystals.

Mp (Dec.) 170-176 °C. **^{1}H NMR** (400 MHz, Chloroform-*d*) *δ* 8.44 (m, 1H, Ar*H*), 8.29 (m, 1H, Ar*H*), 7.77 (m, 2H, Ar*H*), 1.16 (m, 21H, TIPS). **^{13}C NMR** (100 MHz, Chloroform-*d*) δ 166.4, 134.6, 132.3, 131.4, 131.4, 126.1, 115.6, 114.1, 64.6, 18.4, 11.1. **IR** ν 2943 (m), 2865 (m), 1716 (m), 1618 (m), 1604 (s), 1584 (m), 1557 (m), 1465 (m), 1439 (w), 1349 (m), 1291 (m), 1270 (w), 1244 (m), 1140 (m), 1016 (m), 999 (m), 883 (m), 833 (m), 742 (m), 702 (s), 636 (m). Consistent with reported data.[11]

3-Bromopropyl 4-((trimethylsilyl)ethynyl)benzoate (**19g**)

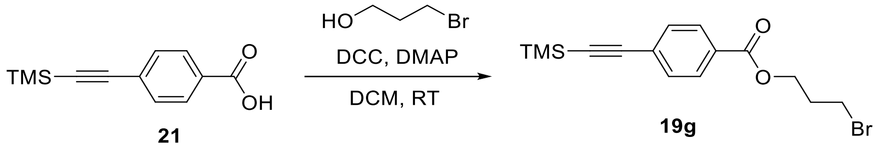

Following a reported procedure,[12] 4-(dimethylamino)-pyridine (67 mg, 0.55 mmol, 12 mol%) was added to a stirred reaction mixture of 4-((trimethylsilyl)ethynyl)benzoic acid (**21**) (1.0 g, 4.6 mmol, 1.0 equiv), dicyclohexylcarbodiimide (1.0 g, 5.0 mmol, 1.1 equiv), 3-bromopropan-1-ol (0.62 mL, 6.9 mmol, 1.5 equiv) in dry CH_2Cl_2 (15 mL) at room temperature. The reaction mixture was filtered after 15 h and the solid was rinsed with dichloromethane (2 x 10 mL). The combined filtrates were concentrated under vacuum. Purification by column chromatography pentane:ethyl acetate 9:1 afforded 3-bromopropyl 4-((trimethylsilyl)ethynyl)benzoate (**19g**) (1.3 g, 3.8 mmol, 82 % yield) as a white solid.

^{1}H NMR (400 MHz, Chloroform-*d*) δ 7.96 (d, *J* = 8.6 Hz, 2H, Ar*H*), 7.52 (d, *J* = 8.7 Hz, 2H, Ar*H*), 4.46 (t, *J* = 6.0 Hz, 2H, OCH_2), 3.54 (t, *J* = 6.6 Hz, 2H, BrCH_2), 2.32 (p, *J* = 6.4 Hz, 2H, CH_2CH_2), 0.26 (s, 9H, TMS). **^{13}C NMR** (101 MHz, Chloroform-*d*) δ 165.8, 131.9, 129.5, 129.4, 127.9, 104.0, 97.9, 62.9, 31.8, 29.4, -0.2. The characterisation data corresponds to the reported literature values.[12]

---

[12] Garreau, M.; Le Vaillant, F.; Waser, J. *Angew. Chem. Int. Ed.* **2019**, *58*, 8182.

1-[(4-(3-Bromoprop-1-yl-benzoate)ethynyl]-1,2-benziodoxol-3(1*H*)-one (**11g**)

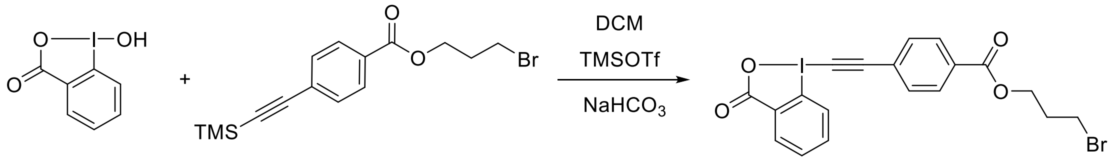

6 **19g** **11g**

Based on a reported procedure,[12] **6** (0.354 g, 1.34 mmol) was charged into a flame-dried 25 mL round bottomed flask equipped with a magnetic stirring bar and dry CH_2Cl_2 (Volume: 3.83 mL) was added. To the later suspension, trimethylsilyl trifluoromethanesulfonate (0.28 mL, 1.5 mmol) was added dropwise over a period of 20 min resulting in a yellowish suspension. The reaction was stirred for 1 hour, at that time, 3-bromopropyl 4-((trimethylsilyl)ethynyl)benzoate (**19g**, 0.500 g, 1.47 mmol) was added portionwise. The suspension coloured grey. The reaction was then stirred 6 hours at room temperature. The reaction was quenched with sat. aq. $NaHCO_3$ (3.5 mL) and stirred for 2 h at 25 °C. A persistent emulsion was obtained, the solution was diluted with 50 mL of EtOAc and 20 mL of water. The organic layer was separated then washed with brine:H_2O 1:1 (50 mL). The organic layer was concentrated to provide an orange crude oil and crude precipitate. The latter was recrystalised from MeCN (ca. 40 mL for 1.0 g of crude). **11g** (0.430 g, 0.838 mmol, 63% yield) was obtained as an off-white partially crystalline solid.

Mp (dec) = 152 °C. **^{1}H NMR** (400 MHz, Chloroform-*d*) δ 8.51 – 8.35 (m, 1H, Ar*H*), 8.25 (dd, *J* = 7.5, 1.6 Hz, 1H, Ar*H*), 8.14 – 7.95 (m, 2H, Ar*H*), 7.88 – 7.74 (m, 2H, Ar*H*), 7.74 – 7.52 (m, 2H, Ar*H*), 4.51 (t, *J* = 6.0 Hz, 2H, OC*H*2), 3.56 (t, *J* = 6.5 Hz, 2H, C*H*2Br), 2.35 (p_{app}, *J* = 6.3 Hz, 2H). **^{13}C NMR** (101 MHz, Chloroform-*d*) δ 166.5, 165.3, 135.1, 132.8, 132.6, 131.8, 131.6, 131.3, 129.8, 126.3, 125.1, 116.1, 105.0, 63.3, 54.1, 31.7, 29.2. **IR** (ν_{max}, cm^{-1}) 3017 (m), 2987 (m), 2971 (m), 2912 (m), 2902 (m), 2154 (m), 1710 (m), 1619 (s), 1600 (s), 1553 (m), 1437 (m), 1392 (m), 1330 (s), 1272 (s), 1258 (s), 1210 (m), 1178 (m), 1102 (s), 1083 (s), 1076 (s), 1016 (m). **HRMS** (ESI/QTOF) m/z: $[M + H]^+$ Calcd for $C_{19}H_{14}{}^{79}BrIO_4^+$ 512.9193; Found 512.9208 Calcd for $C_{19}H_{14}{}^{81}BrIO_4^+$ 514.9195; Found 514.9191.

Prop-2-en-1-yl (3-ethynyl)benzoate (**19h**)

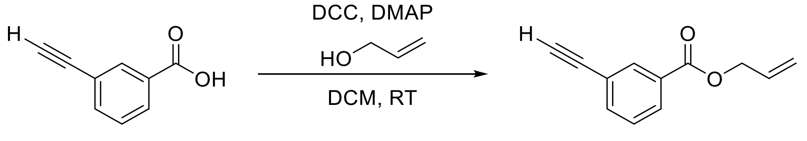

22 **19h**

N,N-dimethylpyridin-4-amine (0.050 g, 0.41 mmol) was added to a stirred reaction mixture of 3-ethynylbenzoic acid (**22**, 0.500 g, 3.42 mmol), *N,N*'-methanediylidenedicyclohexanamine (0.777 g, 3.76 mmol) and prop-2-en-1-ol (0.349 mL, 5.13 mmol) in dry CH_2Cl_2 (14 mL) at RT The reaction mixture was filtered after 15 h and the solid was rinsed with dichloromethane (2 x 5 mL). The combined filtrates were concentrated under vacuum. Purification by column chromatography with pentane:ethyl acetate 10:0 to 9:1 afforded prop-2-en-1-yl 3-(ethynyl)benzoate (**19h**, 0.570 g, 3.06 mmol, 89% yield) as a colorless oil.

^{1}H NMR (400 MHz, Chloroform-*d*) δ 8.19 (t, *J* = 1.7 Hz, 1H, Ar*H*), 8.04 (dt, *J* = 7.9, 1.5 Hz, 1H, Ar*H*), 7.67 (dt, *J* = 7.7, 1.5 Hz, 1H, Ar*H*), 7.41 (t, *J* = 7.8 Hz, 1H, Ar*H*), 6.04 (ddt, *J* = 17.2, 10.4, 5.7 Hz, 1H, C*H*=CH_2), 5.42 (dq, *J* = 17.2, 1.5 Hz, 1H, CH=CH_2), 5.30 (dq, *J* = 10.5, 1.3 Hz, 1H, CH=CH_2), 4.83 (dt, *J* = 5.7, 1.4 Hz, 2H, CH_2-CH=CH_2), 3.13 (s, 1H, aklynyl*H*). Compound was used directly in next step with no further analysis.

1-[(3-(Prop-2-en-1-yl-benzoate)ethynyl]-1,2-benziodoxol-3(1*H*)-one (**11h**)

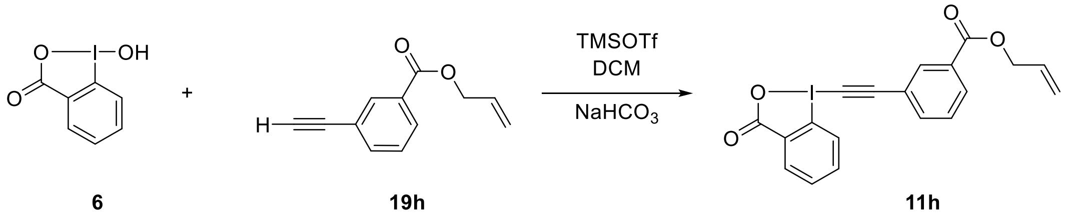

Based on a reported procedure,[12] in an oven-dried 20 mL microwave vial equipped with a magnetic stirring bar, **6** (0.500 g, 1.89 mmol, 1.00 equiv) was suspended in dry CH_2Cl_2 (6 mL, 0.31 M). To the later suspension, trimethylsilyl trifluoromethanesulfonate (0.4 mL, 2 mmol, 1.1 equiv) was added dropwise over a period of 10 min resulting in a yellowish suspension. The reaction was stirred for 1 hour, at that time, allyl 3-ethynylbenzoate (**19h**, 0.388 g, 2.08 mmol, 1.10 equiv) was added dropwise. The suspension coloured grey. The reaction was then stirred 18 hours at 40 °C. The reaction was quenched with sat. aq. $NaHCO_3$ (10 mL) and stirred for 1 h. The layers were separated and the aqueous layer was back-extracted with CH_2Cl_2 (3 x 7 mL). The organic layers were combined, washed with brine:H_2O (1:1) dried over Na_2SO_4 and filtered. The total volume of CH_2Cl_2 was ca. 30 mL. ca. 10 mL of heptane were added. CH_2Cl_2 was removed slowly under reduced pressure until solution clouded. The solution was then left to cool to RT then placed in fridge (5 °C) for 1 h. At this time crystals had started to form. Crystalization proceeded at RT stirring the solution every 5 min with a spatula for 30 min. **11h** (0.312 g, 0.722 mmol, 38% yield) was obtained as an off-white slightly crystalline powder.

Mp (dec.) = 60 °C. **^{1}H NMR** (400 MHz, Chloroform-*d*) δ 8.48 – 8.34 (m, 1H, Ar*H*), 8.29 (d, *J* = 1.8 Hz, 1H, Ar*H*), 8.29 – 8.21 (m, 1H, Ar*H*), 8.17 (dt, *J* = 7.9, 1.5 Hz, 1H, Ar*H*), 7.85 – 7.72 (m, 3H, Ar*H*), 7.54 (t, *J* = 7.8 Hz, 1H, Ar*H*), 6.05 (ddt, *J* = 17.3, 10.4, 5.7 Hz, 1H, C*H*=CH_2), 5.44 (dq, *J* = 17.2, 1.5 Hz, 1H, CH=CH_2), 5.33 (dq, *J* = 10.3, 1.3 Hz, 1H, CH=CH_2), 4.86 (dt, *J* = 5.7, 1.4 Hz, 2H, CH_2-CH=CH_2). **^{13}C NMR** (101 MHz, Chloroform-*d*) δ 166.5, 165.0, 136.8, 135.1, 134.0, 132.6, 131.8, 131.8, 131.7, 131.3, 131.0, 129.0, 126.3, 121.1, 118.9, 116.1, 105.1, 66.1, 51.9. **IR** (ν_{max}, cm^{-1}) 2986 (s), 2973 (s), 2900 (s), 2196 (w), 1717 (s), 1599 (m), 1407 (m), 1271 (s), 1241 (s), 1066 (s). **HRMS** (ESI/QTOF) m/z: $[M + H]^+$ Calcd for $C_{19}H_{14}IO_4^+$ 432.9931; Found 432.9934.

General procedure A: Synthesis of the photocatalysts

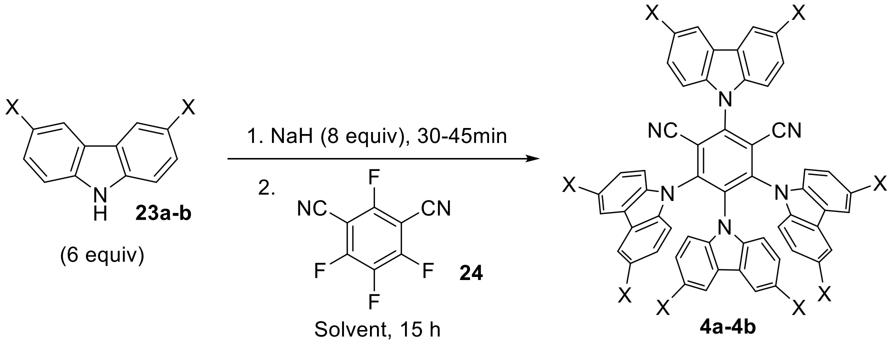

Sodium hydride (60% suspension in mineral oil, 8.0 equiv) was added slowly to a stirred solution of substituted-carbazole **23a-b** (5.0 equiv) in dry THF (0.05 M) under a nitrogen atmosphere at RT After 30 min, 2,4,5,6-tetrafluoroisophthalonitrile **24** (1.0 mmol, 1.0 equiv) was added. After stirring at RT for 15 h, 2 mL water was added to the reaction mixture to quench the excess of NaH. The resulting mixture was then concentrated under reduced pressure. The crude product was purified by recrystallization from hexane:CH_2Cl_2 then filtered. The brown liquid filtrate was concentrated and recrystallized as before. The combined solids were then purified by column chromatography on silica gel with CH_2Cl_2:Hexane.

2,4,5,6-Tetra(9*H*-carbazol-9-yl)isophthalonitrile (4CzIPN, **4a**)

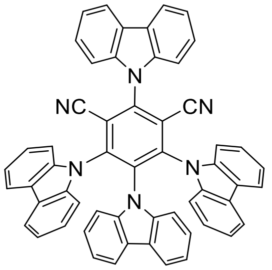

Following **GP A** and starting from 9H-carbazole **23a** (X =H, 1.67 g, 10.0 mmol, 5.00 equiv), sodium hydride (0.60 g, 15 mmol, 7.5 equiv) and 2,4,5,6-tetrafluoroisophthalonitrile **24** (0.40 g, 2.0 mmol) in 40 mL of THF. Recrystallization (Hexanes:CH_2Cl_2 (1:1, 90 mL)) afforded the crude product as a yellow powder. Column chromatography afforded 2,4,5,6-tetra(9H-carbazol-9-yl)isophthalonitrile (**4a**) as a bright yellow crystalline solid (1.14 g, 1.45 mmol, 73 % yield).

Rf (Hexane;CH_2Cl_2 1:1) = 0.29. (yellow spot on TLC). ^{1}**H NMR** (400 MHz, Chloroform-*d*) δ 8.2 (d, *J* = 7.7 Hz, 2H, Ar*H*), 7.8 – 7.6 (m, 8H, Ar*H*), 7.5 (ddd, *J* = 8.0, 6.6, 1.6 Hz, 2H, Ar*H*), 7.3 (d, *J* = 7.5 Hz, 2H, Ar*H*), 7.2 (dd, *J* = 8.4, 1.5 Hz, 4H, Ar*H*), 7.2 – 7.0 (m, 8H, Ar*H*), 6.8 (t, *J* = 7.8 Hz, 4H, Ar*H*), 6.6 (td, *J* = 7.6, 1.2 Hz, 2H, Ar*H*).^{13}**C NMR** (101 MHz, Chloroform-*d*) δ 145.2, 144.6, 140.0, 138.2, 136.9, 134.7, 127.0, 125.8, 124.9, 124.7, 124.5, 123.8, 122.4, 121.9, 121.4, 121.0, 120.4, 119.6, 116.3, 111.6, 109.9, 109.5, 109.4. ^{1}H NMR shift in Chloroform-*d* are consistent with reported data.[13]

(2r,4s,5r)-2,4,5,6-Tetrakis(3,6-*d*ichloro-9*H*-carbazol-9-yl)isophthalonitrile (4ClCzIPN, **4b**)

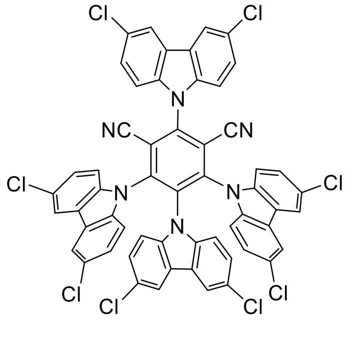

Following **GP A** and starting from 3,6-*d*ichloro-9H-carbazole **23b** (1.96 g, 6.00 mmol, 6.0 equiv), sodium hydride (320 mg, 8.00 mmol, 8.0 equiv) and 2,4,5,6-tetrafluoroisophthalonitrile **24** (200 mg, 1.00 mmol) in 20 mL of THF. Recrystallization (Hexanes:CH_2Cl_2 (1:2, 80 mL)) gave 900 mg of yellow powder, then second recrystallization gave 325 mg of brown powder. Column chromatography of the combined solid afforded (2r,4s,5r)-2,4,5,6-tetrakis(3,6-*d*ichloro-9H-carbazol-9-yl)isophthalonitrile (**4b**) as a bright yellow crystalline solid (830 mg, 0.780 mmol, 87 % yield).

---

[13] Uoyama, H.; Goushi, K.; Shizu, K.; Nomura, H.; Adachi, C. *Nature* **2012**, *492*, 234.

Rf (Hexane:CH_2Cl_2 1:1): 0.25. (yellow spot on TLC). **^{1}H NMR** (400 MHz, DMSO-d_6) δ 8.60 (d, *J* = 2.1 Hz, 2H, Ar*H*), 8.15 (d, *J* = 2.1 Hz, 4H, Ar*H*), 8.08 (d, *J* = 8.8 Hz, 2H, Ar*H*), 7.87 (dd, *J* = 8.8, 2.1 Hz, 2H, Ar*H*), 7.80 (d, *J* = 2.2 Hz, 2H, Ar*H*), 7.69 (d, *J* = 8.8 Hz, 4H, Ar*H*), 7.46 (d, *J* = 8.8 Hz, 2H, Ar*H*), 7.32 (dd, *J* = 8.8, 2.2 Hz, 4H, Ar*H*), 6.93 (dd, *J* = 8.8, 2.2 Hz, 2H, Ar*H*). **^{13}C NMR** (101 MHz, DMSO-d_6) δ 145.0, 144.5, 138.5, 137.4, 136.5, 135.8, 134.5, 127.8, 127.0, 126.4, 125.7, 125.3, 124.2, 123.8, 123.3, 121.6, 120.9, 120.3, 116.8, 112.6, 112.5, 112.3, 111.7. **HRMS** (ESI) calcd for $C_{56}H_{24}Cl_8N_6$ [M+] 1059.9565; found 1059.9573.

Synthesis of electron rich alkenes (1a-k and 9g)

General note on commercial alkenes:

N-vinyl pyrrolidinone (sodium hydroxide as inhibitor) (**1x**) and 2-chloroethyl vinyl ether (triethanolamine as stabiliser) (**9d**) were purchased from Sigma Aldrich. *n*Butyl vinyl ether (**9a**) and 3,4-dihydro-2H-pyran (**9g**) were purchased from Acros. ((Vinyloxy)methyl benzene (**9b**) and 2-ethoxy prop-1-ene (**9f**) were purchased from Fluorochem. Allyl vinyl ether (**9c**) was purchased from abcr. Cyclohexyl vinyl ether (stabilized with KOH) (**9e**) was purchased from TCI. All commercial alkenes were filtered over basic alumina before use and were used within 15 min without direct light exposition.

General note on synthesised alkenes:

All alkenes were used within 6 months of synthesis, stored at 4 °C and hidden from light. Some degradation (coloration) could be observed although it was not detrimental for the reaction yield.

N-vinyloxazolidin-2-one (1a)

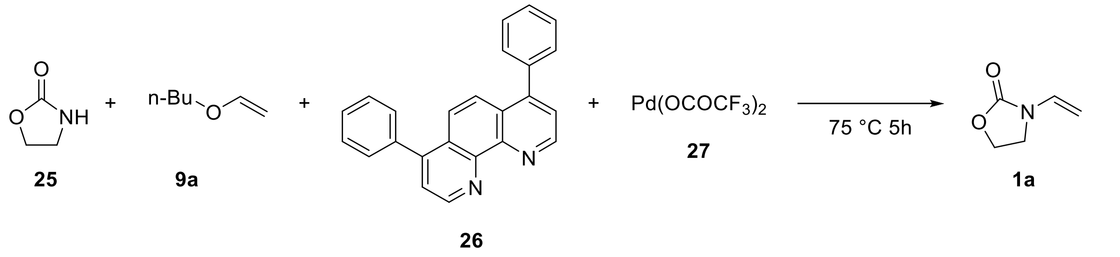

Following a modified reported procedure,[14] in a 25 mL round bottomed flask equipped with magnetic stirrer, bathophenanthroline (**26**, 0.015 g, 0.045 mmol, 0.05 equiv), $Pd(OCOCF_3)_2$ (**27**, 0.015 g. 0.045 mmol, 0.05 equiv) and oxazolidinone (**25**, 0.077 g 0.904 mmol, 1.0 equiv) were dissolved in *n*butyl vinyl ether (**9a**, 1.0 mL, 7.8 mmol, 8.6 equiv). The flask was closed with a septum and then opened to the atmosphere by means of a needle (1.2 x 40 mm). The yellow suspension was then stirred at 75 °C for 2 hours. At this time, the mixture cooled down to room temperature. With no further treatment, the crude oil was then submitted directly to

[14] Brice, J. L.; Meerdink, J. E.; Stahl, S. S. *Org. Lett.* **2004**, *6*, 1845–1848.

column chromatography (SiO_2; Pent:EtOAc 98:2 to 8:2)[15] affording **1a** as a colorless oil (0.095 g, 0.84 mmol, 92% yield).

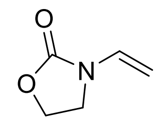

^{1}H NMR (400 MHz, Chloroform-*d*) δ 6.88 (dd, *J* = 15.8, 9.0 Hz, 1H, NC*H*=CH_2), 4.51 – 4.38 (m, 3H, cyclic-CH_2 + CH=CH_2), 4.29 (dd, *J* = 15.8, 1.3 Hz, 1H, NCH=CH_2), 3.79 – 3.64 (m, 2H, cyclic-CH_2). The values of the ^{1}H NMR spectrum are in accordance with reported literature data.[14]

Tert-butyl phenethyl(vinyl)carbamate (**1b**)

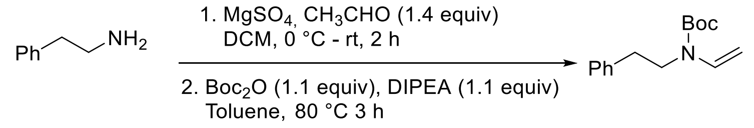

28 **1b**

A flame-*d*ried, 50 mL round-bottomed, two-necked flask was charged with anhydrous $MgSO_4$ (stored in the glove box; 4.5 g), closed, evacuated, and backfilled with nitrogen. CH_2Cl_2 (dry; 10 mL) was added followed by 2-phenylethanamine (**28**, 1.3 mL, 10 mmol, 1.0 equiv). The resulting suspension was cooled down to 0 °C (ice/water bath). Acetaldehyde (0.80 mL, 14 mmol, 1.4 equiv) was then added. The mixture was stirred and allowed to warm to room temperature over a period of 1 hour, and then stirred at room temperature for one additional hour. The solids were then removed through rapid filtration. CH_2Cl_2 was then distilled off (1 atm, 50 °C). To the crude imine was added a solution of di-*tert*-butyl dicarbonate (2.40 g, 11.0 mmol, 1.1 equiv) in toluene (dry; 5.0 mL), followed by DIPEA (freshly distilled over KOH; 1.8 mL, 11 mmol, 1.1 equiv). The resulting yellow solution was stirred at 70 °C for 3 hours. The reaction was stopped and the volatiles were removed under reduced pressure. The resulting crude yellow-orange oil was submitted to column chromatography (Biotage, 40 g SiO_2; EtOAc in pentane, 0 to 8%). The desired product was obtained as a mixture with unreacted Boc_2O. The excess Boc_2O was removed as following: the eluate was dissolved in EtOH (5 mL) and imidazole (300 mg) and DMAP (54.0 mg, 0.1 eq compared to imidazole) were added. The resulting mixture was stirred at room temperature for 10 minutes. It was then concentrated under reduced pressure. The crude oil was submitted to column chromatography (Biotage, 24 g SiO_2; EtOAc in pentane, 0 to 10%) to furnish pure *tert*-butyl phenethyl(vinyl)carbamate **1b** as a pale yellow oil (0.607 g, 2.45 mmol, 25% yield).

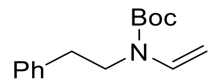

Rf (pentane:Et_2O 95:5) = 0.5.
^{1}H NMR (400 MHz, Acetonitrile-*d*3) δ 7.30 (dd, *J* = 8.0, 6.7 Hz, 2H, Ph*H*), 7.26 - 7.18 (m, 3H, Ph*H*), 7.06 (br s, 1H, -C*H*=CH_2), 4.43 (d, *J* = 16.2 Hz, 1H, -CH=CH_2), 4.25 (br s, 1H, -CH=CH_2), 3.75 - 3.64 (m, 2H, CH_2), 2.88 - 2.76 (m, 2H, CH_2), 1.41 (br s, 9H, C(CH_3)$_3$ in Boc).
^{13}C NMR (101 MHz, Acetonitrile-*d*3) δ 153.7, 140.3, 133.4, 129.9, 129.4, 127.2, 91.4, 81.6, 45.4, 33.7, 28.2. **IR** (ν_{max}, cm^{-1}) 3372 (m), 2969 (m), 2924 (m), 2880 (m), 1704 (w), 1614 (m), 1595 (s), 1557 (m), 1493 (m), 1352 (s), 1148 (s), 1231 (m), 1052 (m), 880 (m), 828 (m), 765 (m), 745 (s), 694 (m). **HRMS** (ESI/QTOF) m/z: [M + Na]$^+$ Calcd for $C_{15}H_{21}NNaO_2^+$ 270.1464; Found 270.1464.

[15] Pentane:EtOAc 95:5 needs to be run for at least 5 column volumes due to remove all the excess butyl vinyl ether.

Benzyl phenethyl(vinyl)carbamate (**1c**)

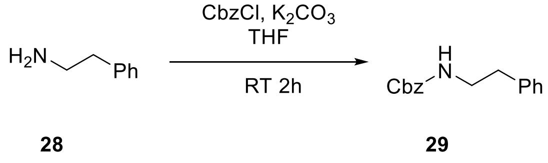

To a flame dried flask and under argon, 2-phenethylamine (4.0 mL, 32 mmol, 1 equiv) and powered K_2CO_3 were suspended in anhydrous THF (125 mL, 0.25 M). Benzyl chloroformate (5.0 mL, 35 mmol, 1.1 equiv) was added dropwise to the suspension. The reaction was stirred at room temperature for 2 h. The solution was quenched with sat. aq. $NaHCO_3$ (40 mL) and the product was extracted with CH_2Cl_2 (3 x 30 mL). The combined organic layers were washed with sat. aq. $NaHCO_3$ (2 x 40 mL), dried over Na_2SO_4 and filtered. The solvent was removed under reduced pressure and the crude oil was purified by flash chromatography (SiO_2, Pentane : EtOAc 1 : 4) affording pure benzyl phenethylcarbamate (8.0 g, 31 mmol, 99% yield). The compound was used directly in next step.

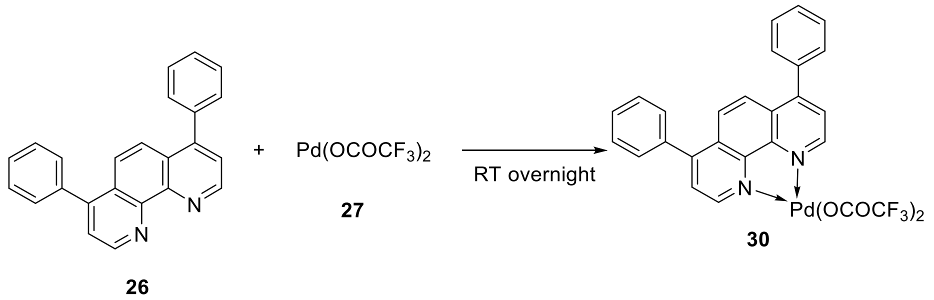

In a 25 mL flask palladium trifluoroacetate (**27**, 0.415 g, 1.21 mmol) was dissolved in MeCN (29.9 mL). The flask was put under nitrogen and stirred at RT. A solution of 4,7-diphenyl-1,10-phenanthroline (**26**, 0.402 g, 1.21 mmol) in CH_2Cl_2 (Ratio: 1.000, Volume: 14.95 mL) was added. The dark brown solution was stirred overnight.

n-Hexane was added slowly under vigorous stirring (ca. 40 mL). The mixture was left to precipitate slowly over 30 min at rt. The suspension was cooled to 0 °C with an ice bath and the reaction was filtered and washed with pentane. 0.100 g of yellow complex was recovered. The filtrate was concentrated under reduced pressure, redisoved in a minimum of DCM (ca. 5 mL) and precipitated out with n-hexane. the suspension was cooled to 0 °C with an ice bath and was filtered a second time affording 0.563 g of yellow complex. The two batches were further dried on the high vaccum affording BphenPd($O_2C_2F_3$)$_2$ (**30**, 0.595 g, 0.895 mmol, 73.9 % yield) (0.095 of first batch and 0.500 g of second batch). *This complexation is quite sensitive to scale and the purity of the palladium and phenathroline these compounds were purchased from Acros and were used with no further purification. Yields were variable between different bottles of the same supplier. Hexane proved more efficient than pentane for the precipitation, but in some cases precipitation was not observed and couldn't be induced.*

^{1}H NMR (400 MHz, DMSO-d_6) δ 8.48 (s, 2H), 8.27 – 8.04 (m, 4H), 7.69 (s, 10H).**^{19}F NMR** (376 MHz, DMSO-d_6) δ -73.3. The ^{1}H NMR data corresponds to the reported literature data.[16]

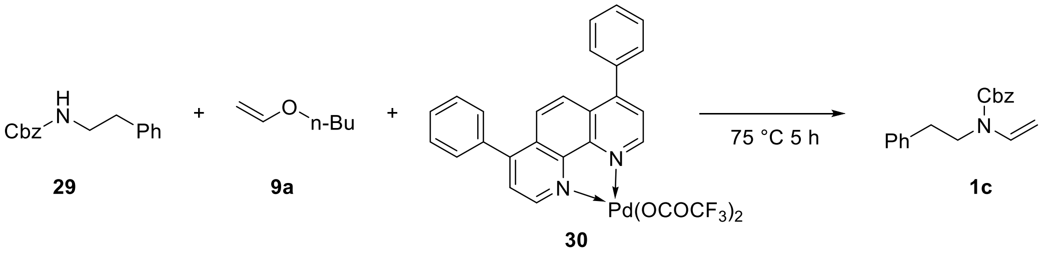

A 5 mL round-bottomed test tube was charged with BphenPd(O_2CCF_3)$_2$ (**30**, 39.0 mg, 0.0590 mmol, 7.5 mol%). *n*Butyl vinyl ether (**9a**, 1.2 mL, 9.4 mmol, 12 equiv) was added, followed by benzyl phenethylcarbamate (0.200 g, 0.783 mmol, 1.0 equiv). The vial was sealed with a PTFE septum, which was then pierced with a needle (gauge 18 - "pink needle") in order to ensure exposure to air. The resulting brown-orange suspension was stirred at 80 °C for 3 hours. After this time, TLC analysis showed complete conversion of the starting material. The reaction mixture was then allowed to cool down to room temperature and was subsequently submitted to column chromatography (Biotage, 25 g SiO_2; EtOAc in pentane, 2 to 20% - 3 CV used to elute the butyl vinyl ether off). Pure benzyl phenethyl(vinyl)carbamate **1c** (0.160 g, 0.569 mmol, 73% yield) was obtained as a colorless oil.

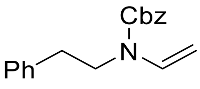

Rf (pentane :EtOAc 9 :1) 0.60. **^{1}H NMR** (400 MHz, Acetonitrile-d_3) δ 7.43 - 7.31 (m, 5H, Ph*H*), 7.27 (d, *J* = 7.1 Hz, 3H, Ph*H*), 7.24 - 7.15 (m, 2H, Ph*H*), 7.08 (dd, *J* = 16.0, 9.4 Hz, 1H, -*CH*=CH$_2$), 5.09 (br s, 2H, Ph*CH$_2$*O), 4.52 (d, *J* = 16.1 Hz, 1H, -CH=*CH$_2$*), 4.32 (br s, 1H, -*CH*=CH$_2$), 3.75 (t, *J* = 7.7 Hz, 2H, *CH$_2$CH$_2$*), 2.84 (dd, *J* = 8.8, 6.6 Hz, 2H, *CH$_2$CH$_2$*).
^{13}C NMR (101 MHz, Acetonitrile-d_3, 2 carbons are not resolved) δ 154.2, 140.0, 137.6, 133.4, 129.8, 129.5, 129.4, 129.1, 128.9, 127.3, 92.7, 68.4, 45.5, 33.9. **IR** (ν_{max}, cm^{-1}) 3027 (w), 3065 (w), 3090 (w), 2964 (w), 1707 (s), 1631 (s), 1389 (s), 1421 (m), 1453 (m), 1345 (m), 1180 (s), 1275 (m), 1104 (m), 837 (m), 748 (s), 698 (s). **HRMS** (ESI/QTOF) m/z: [M + Na]$^+$ Calcd for $C_{18}H_{19}NNaO_2^+$ 304.1308; Found 304.1313.

Tert-butyl benzyl(vinyl)carbamate (**1d**)

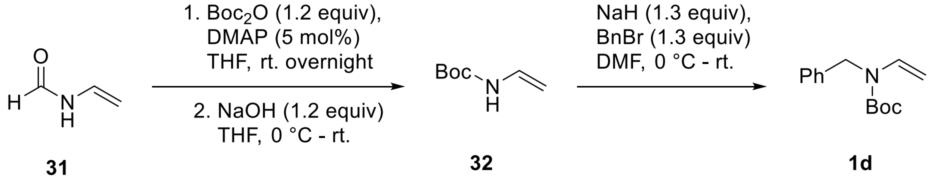

Following a reported procedure,[17] in a 250 mL, three-necked, round-bottomed flask, *N*-vinylformamide (**31**, 2.7 mL, 38 mmol, 1.0 equiv) was dissolved in THF (dry; ca. 130 mL). To the resulting pale yellow solution, di-*tert*-butyl dicarbonate (10.1 g, 46.2 mmol, 1.2 equiv) and

---

[16] Milani, B.; Alessio, E.; Mestroni, G.; Sommazzi, A.; Garbassi, F.; Zangrando, E.;Bresciani-Pahor, N.; Randaccio, L. *J. Chem. Soc., Dalton Trans.*, **1994**, 1903-1911

[17] Kassir, A. F.; Ragab, S. S.; Nguyen, T. A. M.; Charnay-Pouget, F.; Guillot, R.; Scherrmann, M. C.; Boddaert, T.; Aitken, D. J. *J. Org. Chem.* **2016**, *81*, 9983.

DMAP (0.235 g, 1.93 mmol, 5 mol%) were added under nitrogen. The mixture, which rapidly became bright yellow, was then stirred at room temperature overnight. After 16 hours, it was concentrated under reduced pressured. The orange crude oil was then submitted to column chromatography (Biotage, 80 g SiO_2; EtOAc in pentane, 5 to 50%) to afford *tert*-butyl formyl(vinyl)carbamate (4.70 g, 27.5 mmol, 71% yield) as a yellow oil. The compound was used directly in next step, in a 100 mL one-necked, round-bottomed flask, *tert*-butyl formyl(vinyl)carbamate (4.60 g, 26.9 mmol, 1.0 equiv) was dissolved in THF (16 mL). The solution was cooled to 0 °C (ice water bath). Aq. NaOH (2.0 M; 16 mL, 32 mmol, 1.2 equiv) was added drop-wise, over a period of 20 minutes (syringe pump). Once the addition was complete, stirring was continued at 0 °C for another 15 minutes. The suspension was then allowed to warm to room temperature and stirred for additional 3 hours. Water (30 mL) was then added and the aqueous layer was extracted with MeO*t*Bu (4 x 30 mL). The combined organic extracts were washed with water, brine, dried over Na_2SO_4, filtered, and concentrated under reduced pressure. The resulting crude solid was dissolved in pentane at room temperature (in order to reduce the volume of pentane, the dissolution was done under ultrasound irradiation). The solution was then allow to stand at -20 °C (freezer) overnight. The precipitate was then collected by filtration and washed with a minimal amount of ice-cold pentane. *tert*-Butyl vinylcarbamate (**32**, 2.45 g, 17.1 mmol, 64% yield) was obtained as a crystalline, colorless solid.

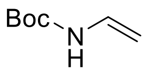

^{1}H NMR (400 MHz, Acetonitrile-d_3) δ 7.31 (s, 1H, -CH=CH$_2$), 6.60 (ddd, J = 15.8, 10.8, 8.9 Hz, 1H, -CH=CH_2), 4.47 (d, J = 15.8 Hz, 1H, -CH=CH_2), 4.14 (d, J = 8.9 Hz, 1H, NH), 1.43 (s, 9H, C(CH_3)$_3$). **^{3}C NMR** (101 MHz, Acetonitrile-d_3) δ 153.9, 131.4, 92.3, 80.5, 28.4.
Compound was used directly in next step with no further analysis.

Following a reported procedure,[18] in a 100 mL, two-necked, round-bottomed flask, *tert*-butyl vinylcarbamate (**32**, 0.773 g, 5.40 mmol, 1.0 equiv) was dissolved in DMF (dry; 12.6 mL). The colorless solution was cooled to 0 °C (ice-water bath) and sodium hydride (60% dispersion in paraffin; 0.281 g, 7.02 mmol, 1.3 equiv) was added in single portion. Bubbling was immediately observed. The suspension was stirred at 0 °C for 30 minutes and at room temperature for another 30 minutes. The mixture looked, at this point, like a yellow-grey turbid solution. Benzyl bromide (0.84 mL, 7.0 mmol, 1.3 equiv) was then added drop-wise, followed by a catalytic amount of TBAI (tip of a spatula). The mixture was stirred at room temperature for 3 hours. After this time, full conversion of the starting material was observed based on TLC analysis (pentane:EtOAc 97:3). The reaction was quenched by cautious addition of water (12 mL), followed by sat. aq. NH_4Cl (12 mL). The aqueous layer was then extracted with ether (4 x 20 mL). The combined organic extracts were washed with water (30 mL), brine (2 x 30 mL), dried over $MgSO_4$, filtered, and concentrated under vacuum. The resulting yellow crude oil was submitted to column chromatography (Biotage, 40 g SiO_2; EtOAc in pentane, 0 to 7%) to provide *tert*-butyl benzyl(vinyl)carbamate **1d** (95% pure; 0.822 g, 3.35 mmol, 62% yield) as a yellow oil.

---

[18] Liu, S.-Y.; Xu, S. WO2015/126400, 2015, A1.

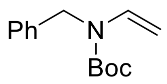

Rf(pentane:EtOAc 9:1) = 0.5.
^{1}H NMR (400 MHz, DMSO-d_6, undefined rotameric mixture) δ 7.48 – 7.27 (m, 3H, Ph*H*), 7.30 – 7.15 (m, 2H, Ph*H*), 7.06 (bs, 1H, C*H*=CH$_2$), 4.69 (s, 2H, PhC*H$_2$*), 4.29 (bs, 1H, CH=C*H$_2$*), 4.19 (bs, 1H, CH=C*H$_2$*), 1.44 (bs, 9H, C(C*H$_3$*)$_3$). **^{13}C NMR** (101 MHz, DMSO-d_6, undefined rotameric mixture) δ 153.0, 138.8, 138.0, 133.1, 128.9, 128.8, 128.7, 128.0, 127.9, 127.3, 126.8, 93.0, 81.5, 71.9. The ^{1}H NMR characterisation data are in accordance to the reported literature data.[19]

T*ert*-butyl methyl(vinyl)carbamate (**1e**)

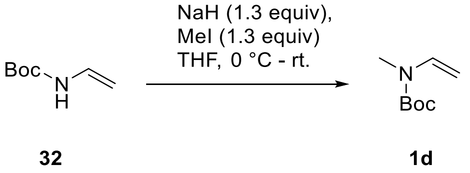

Following a reported procedure,[20] A 100 mL, two-necked, round-bottomed flask was charged with NaH (60% w/w dispersion in mineral oil; 0.192 g, 4.80 mmol, 1.2 equiv). The flask was evacuated, and backfilled with nitrogen (3 times). THF (dry; 30 mL) was then added. The resulting suspension was cooled to 0 °C (ice - water bath) and a solution of *t*-butyl N-vinyl carbamate (**32**, 0.572 g, 4.00 mmol, 1.0 equiv) in THF (dry; 10 mL) was added slowly, by syringe at the same temperature. Immediately, moderate gas bubbling was observed. The resulting pale yellow mixture was stirred at 0 °C for 30 minutes. Methyl iodide (0.39 mL, 6.4 mmol, 1.6 equiv) was then added. The cooling bath was removed and stirring was continued under Ar (balloon) overnight. The reaction was then quenched by addition of sat. aq. NH_4Cl (30 mL). The aqueous layer was separated and extracted with EtOAc (3 x 30 mL). The combined organic layers were washed with brine, dried over $MgSO_4$, filtered, and concentrated under reduced pressure. The resulting crude oil was submitted to column chromatography (SiO_2; EtOAc:pentane 0:100 to 25 / 75) to give *tert*-butyl N-methyl N-vinyl carbamate (**1d**, 0.282 g, 1.70 mmol, 45% yield) as a colorless oil.

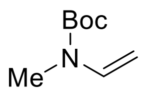

Although this compound is known,[21] no NMR data has been reported at RT.
Rf(pentane:EtOAc = 9:1) = 0.55.
^{1}H NMR (400 MHz, Acetonitrile-d3) δ 7.15 (m, 1H, -*CH*=CH$_2$), 4.27 (d, *J* = 15.8 Hz, 1H, -CH=*CH$_2$*), 4.18 (m, 1H, -CH=*CH$_2$*), 2.96 (s, 3H, *CH$_3$*), 1.47 (s, 9H, C(*CH$_3$*)$_3$). **^{13}C NMR** (101 MHz, Acetonitrile-d_3) δ 153.3, 134.7, 90.7, 81.2, 29.6, 28.0.
IR (ν_{max}, cm^{-1}) 3116 (w), 2978 (w), 1708 (s), 1626 (s), 1479 (m), 1459 (w), 1436 (m), 1410 (m), 1352 (s), 1318 (s), 1290 (m), 1254 (w), 1145 (s), 1060 (m), 979 (w), 865 (m), 835 (m), 768 (m), 659 (w). **HRMS** (APPI/LTQ-Orbitrap) m/z: [M + Na]$^+$ Calcd for $C_8H_{15}NNaO_2^+$ 180.0995; Found 180.0998.

T*ert*-butyl allyl(vinyl)carbamate (**1f**)

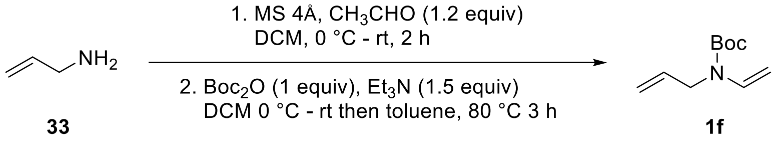

[19] Bach, T. and Schröder, J. *Liebigs Ann./Recl.*, **1997**, *1997*, 2226
[20] Boyington, A. J.; Seath, C. P.; Zearfoss, A. M.; Xu, Z.; Jui, N. T. *J. Am. Chem. Soc.* **2019**, *141*, 4147.
[21] Chu, S.; Münster, N.; Balan, T.; Smith, M. D. *Angew. Chem. Int. Ed.* **2016**, *55*,14306.

Following a modified reported procedure,[22] to a mixture of allylamine (**33**, 1.5 mL, 20.1 mmol, 1.0 equiv) and 4 Å mol sieves (2 g) in CH_2Cl_2 (25 mL) at 0 °C was added acetaldehyde (1.4 mL, 24 mmol, 1.2 equiv) dropwise. The solution was warmed to rt over 1 h, stirred for an additional 1 h, and decanted. To the resultant solution at 0 °C, triethylamine (3.90 mL, 30.1 mmol, 1.5 equiv) and di-*tert*-butyl dicarbonate (4.37 g, 20.1 mmol, 1.0 equiv) were added. The mixture was warmed to rt and stirred for 15 h. The solution was concentrated, and toluene (30 mL) was added. The solution was heated at 70 °C for 3 h and then concentrated. Purification by silica-gel chromatography (SiO_2-Et_3N deactivated; Pent:Et_2O 98:2) afforded *tert*-butyl allyl(vinyl)carbamate **1e** (0.5496 g, 3.00 mmol, 15 % yield) as a colorless oil.

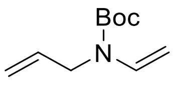

Rf (pentane:Et_2O 95:5) = 0.4.
^{1}H NMR (400 MHz, Acetonitrile-*d*$_3$) δ 7.06 (dd, *J* = 15.8, 9.4 Hz, 1H, N*CH*=CH_2), 5.78 (ddt, *J* = 17.2, 10.3, 5.0 Hz, 1H, *CH=CH$_2$*), 5.17 – 4.94 (m, 2H, *CH=CH$_2$*), 4.30 (d, *J* = 16.0 Hz, 1H, NCH=*CH$_2$*), 4.18 (d, 9.6 Hz, 1H, *CH=CH$_2$*), 4.13 (d, *J* = 5.0 Hz, 2H, *CH$_2$*), 1.46 (s, 9H, C(*CH$_3$*)$_3$).
^{13}C NMR (101 MHz, Acetonitrile-*d*$_3$) δ 152.7, 133.5, 133.3, 115.7, 91.4, 81.4, 45.7, 27.9. **IR** (ν_{max}, cm^{-1}) 3006 (s), 2985 (s), 2942 (s), 2924 (s), 2909 (s), 2883 (s), 1723 (s), 1703 (s), 1633 (s), 1626 (s), 1618 (s), 1419 (s), 1364 (s), 1239 (s), 1146 (s). **HRMS** (APPI/LTQ-Orbitrap) m/z: $[M + H]^+$ Calcd for $C_{10}H_{18}NO_2^+$ 184.1332; Found 184.1327.

Tert-butyl (2-*tert*-butyldimethylsilyl)oxy)ethyl)(vinyl)carbamate (**1g**)

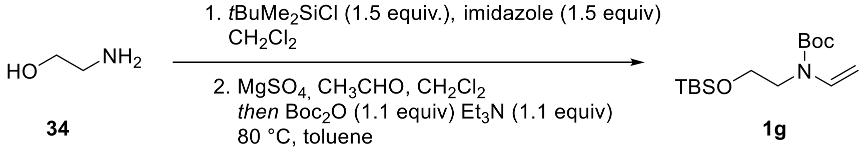

34 **1g**

Under standard silylating conditions: in a 50 mL single-necked, round bottomed flask, a solution of *tert*-butyldimethylchlorosilane (2.41, 16.0 mmol, 1.5 equiv) in dichloromethane (7.0 mL) was added drop-wise over a period of 3 min to a stirred solution of ethanolamine (**34**, 0.80 mL, 13 mmol, 1.0 equiv) and imidazole (1.36 g, 20.0 mmol, 1.5 equiv) in dichloromethane (14 mL) at room temperature. The resulting mixture initially looked like a milky solution that became clear and colorless after being stirred at room temperature for 1 hour. Water (20 mL) was then added, and the layers were separated. The aqueous layer was extracted with dichloromethane (2 x 20 mL), and the combined organic extracts were dried over $MgSO_4$, filtered and concentrated *in vacuo* to give 2-((*tert*-butyldimethylsilyl)oxy)ethanamine (**35**, 2.05 g, 11.7 mmol, 88% yield) as pale yellow oil, which was used directly in next step with no further analysis.

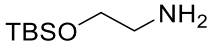

35

^{1}H NMR (400 MHz, Chloroform-*d*) δ 3.62 (t, *J* = 5.3 Hz, 2H, *CH$_2$*), 2.77 (t, *J* = 5.3 Hz, 2H, *CH$_2$*), 1.74 (s, 2H, *NH$_2$*), 0.89 (s, 9H, C(*CH$_3$*)$_3$), 0.06 (s, 6H, *CH$_3$*). **^{13}C NMR** (101 MHz, Chloroform-*d*) δ 65.1, 44.3, 25.9, 18.3, -5.3.

A flame-*d*ried, 100 mL round-bottomed, two-necked flask was charged with anhydrous $MgSO_4$ (stored in the glove box; 4.5 g), closed, evacuated, and backfilled with nitrogen. CH_2Cl_2 (dry; 10 mL) was added, followed by 2-((*tert*-butyldimethylsilyl)oxy)ethanamine (**35**, 1.75 g, 10.0 mmol,

[22] Cossey, K. N.; Funk, R. L. *J. Am. Chem. Soc.* **2004**, *126*, 12216–12217.

1.0 equiv). The resulting suspension was cooled down to 0 °C (ice/water bath). Acetaldehyde (0.80 mL, 14 mmol, 1.4 equiv) was then added. The mixture was stirred at 0 ° for 45 minutes, and then at room temperature for additional 45 minutes. The solids were then removed through rapid filtration. CH_2Cl_2 was then distilled off (rotary evaporator) to give a red crude imine. To the crude imine was added a solution of di-*tert*-butyl dicarbonate (2.40 g, 11.0 mmol, 1.1 equiv) in toluene (dry; 5.0 mL), followed by triethylamine (1.5 mL, 11 mmol, 1.1 equiv). The resulting orange suspension was stirred at 80 °C for 5 hours, slowly becoming a dark orange solution. The reaction was stopped and the volatiles were removed under reduced pressure. The resulting crude yellow-orange oil was submitted to column chromatography (SiO_2; Pentane:Et_2O 248:2 to 24:1). The desired product was obtained as a mixture with unreacted Boc_2O. The excess Boc_2O was removed as following: the eluate was dissolved in EtOH (5 mL) and imidazole (300 mg) and DMAP (54.0 mg, 0.1 eq compared to imidazole) were added. The resulting mixture was stirred at room temperature for 10 minutes. It was then concentrated under reduced pressure. The crude oil was submitted to column chromatography (Biotage, 24 g SiO_2; EtOAc in pentane, 1 to 5%) to furnish pure *tert*-butyl (2-((*tert*-butyldimethylsilyl)oxy)ethyl)(vinyl)carbamate **1g** (0.379 g, 1.26 mmol, 13% yield) as a colorless oil.

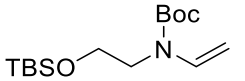

Rf (pentane:Et_2O 95:5) = 0.63.

^{1}H NMR (400 MHz, Acetonitrile-*d*$_3$) δ 7.03 (m, 1H, -*CH*=CH_2), 4.40 (d, *J* = 16.0 Hz, 1H, -CH=*CH_2*), 4.20 (br s, 1H, -CH=*CH_2*), 3.72 (td, *J* = 6.1, 0.8 Hz, 2H, *CH_2*), 3.60 (t, *J* = 6.2 Hz, 2H, *CH_2*), 1.46 (s, 9H, C(*CH_3*)$_3$), 0.88 (s, 9H, C(*CH_3*)$_3$), 0.04 (s, 6H, *CH_3* in TBS). **^{13}C NMR** (101 MHz, Acetonitrile-*d*$_3$) δ 153.8, 134.2, 91.7, 81.7, 60.4, 45.9, 28.4, 26.2, 18.9, -5.2. **IR** (ν_{max}, cm^{-1}) 2957 (m), 2862 (w), 1707 (s), 1631 (m), 1466 (m), 1358 (s), 1250 (m), 1142 (s), 1117 (s), 831 (s), 780 (s), 926 (w), 977 (w). **HRMS** (ESI/QTOF) m/z: [M + Na]$^+$ Calcd for $C_{15}H_{31}NNaO_3Si^+$ 324.1965; Found 324.1966.

Ethyl 3-((*tert*-butoxycarbonyl)(vinyl)amino)propanoate (**1h**)

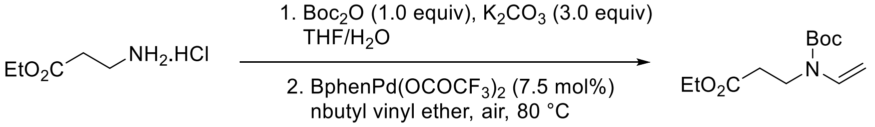

36 **1h**

In a 50 mL single-necked, round-bottomed flask, ethyl 3-aminobutanoate hydrochloride (**36**, 0.550 g, 3.58 mmol, 1.0 equiv), Boc_2O (0.781 g, 3.58 mmol, 1.0 equiv) and K_2CO_3 (1.485 g, 10.74 mmol) were suspended in a mixture of THF:H_2O (10 mL, 1 : 1). The mixture was stirred at room temperature for 4 hours. It was then diluted with water (20 mL) and extracted with EtOAc (20 mL x 2). The combined organic extracts were washed with brine, dried over $MgSO_4$, filtered, and concentrated *in vacuo* to give pure ethyl 3-((*tert*-butoxycarbonyl)amino)propanoate (**37**, 0.707 g, 3.25 mmol, 91% yield) as a colorless oil.

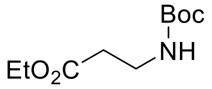

37

^{1}H NMR (400 MHz, Acetonitrile-*d*$_3$) δ 5.37 (s, 1H, N*H*), 4.09 (q, *J* = 7.1 Hz, 2H, O*CH_2*CH_3), 3.26 (q, *J* = 6.5 Hz, 2H, *CH_2*), 2.43 (t, *J* = 6.6 Hz, 2H, *CH_2*), 1.39 (s, 9H, C(*CH_3*)$_3$ in Boc), 1.21 (t, *J* = 7.1 Hz, 3H, OCH_2*CH_3*). **^{13}C NMR** (101 MHz, Acetonitrile-*d*$_3$) δ 172.7, 156.7, 79.3, 61.1, 37.1, 35.4, 28.5, 14.5.
The compound was used directly in next step with no further analysis.

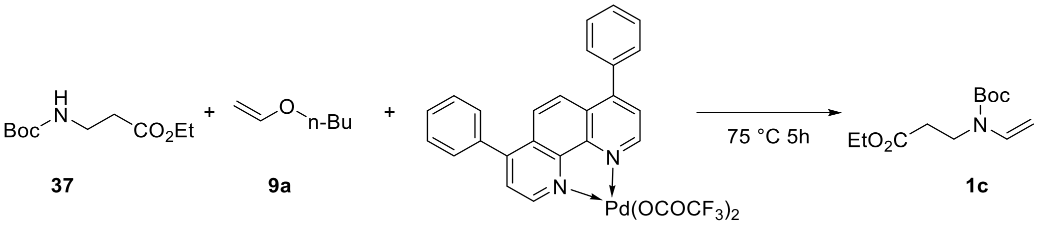

37 **9a** **30** **1c**

Following an adapted reported procedure,[23] a 5 mL round-bottomed test tube was charged with (dpp)Pd(O_2CCF_3)$_2$ (31.0 mg, 0.0460 mmol, 5 mol%). Butyl vinyl ether (1.5 mL, 11 mmol, 12 equiv) was added, followed by ethyl 3-((*tert*-butoxycarbonyl)amino)propanoate (0.200 g, 0.921 mmol, 1.0 equiv). The vial was sealed with a PTFE septum, which was then pierced with a needle (gauge 18 - "pink needle") in order to ensure exposure to air. The resulting brown-orange suspension was stirred at 80 °C for 2 hours. After this time, the mixture looked like a clear orange solution. According to TLC analysis, the starting material was still present after this time, with no further progress upon stirring the mixture at the same temperature for additional 60 minutes. A further amount of catalyst (16.0 mg, 0.0260 mmol, 2.5 mol%; overall 7.5 mol%) was therefore added and stirring was continued at 80 °C for 2 hours. The reaction mixture was then allowed to cool down to room temperature and was subsequently submitted to column chromatography (Biotage, 25 g SiO_2; EtOAc in pentane, 2 to 20%).[15] Pure ethyl 3-((*tert*-butoxycarbonyl)(vinyl)amino)propanoate **1h** (0.121 g, 0.499 mmol, 54% yield) was obtained as a colorless oil.

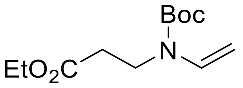

Rf (pentane:EtOAc 9:1) = 0.60.

^{1}H NMR (400 MHz, Acetonitrile-*d*$_3$) δ 7.00 (dd, *J* = 16.1, 9.6 Hz, 1H, -*CH*=CH$_2$), 4.36 (d, *J* = 16.1 Hz, 1H, -CH=*CH$_2$*), 4.21 (d, *J* = 9.5 Hz, 1H, -CH=*CH$_2$*), 4.08 (q, *J* = 7.1 Hz, 2H, O*CH$_2$*CH$_3$), 3.81 - 3.68 (m, 2H, *CH$_2$*), 2.49 (dd, *J* = 8.4, 6.5 Hz, 2H, *CH$_2$*), 1.46 (s, 9H, C(*CH$_3$*)$_3$ in Boc), 1.21 (t, *J* = 7.1 Hz, 3H, OCH$_2$*CH$_3$*). **^{13}C NMR** (101 MHz, Acetonitrile-*d*$_3$) δ 171.8, 153.0, 132.9, 91.0, 81.7, 60.8, 39.3, 32.3, 27.9, 14.1. **IR** (ν_{max}, cm^{-1}) 3382 (w), 2976 (w), 1732 (m), 1688 (m), 1516 (m), 1370 (m), 1415 (w), 1447 (w), 1282 (m), 1250 (s), 1167 (s), 1072 (m), 1021 (m), 977 (m), 856 (m), 787 (m), 647 (m). **HRMS** (ESI/QTOF) m/z: [M + Na]$^+$ Calcd for $C_{12}H_{21}NNaO_4^+$ 266.1363; Found 266.1362.

Tert-butyl cyclohexenyl(vinyl)carbamate (**1i**)

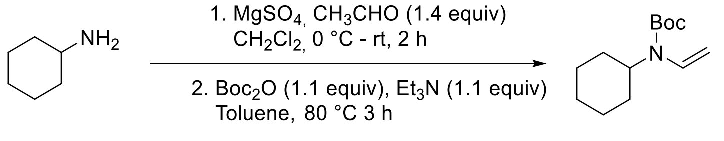

38 **1i**

A flame-*d*ried, 100 mL round-bottomed, two-necked flask was charged with anhydrous $MgSO_4$ (stored in the glove box; 4.5 g), closed, evacuated, and backfilled with nitrogen. CH_2Cl_2 (dry; 10 mL) was added followed by cyclohexanamine (**38**, 1.2 mL, 10 mmol, 1.0 equiv). The resulting suspension was cooled down to 0 °C (ice/water bath). Acetaldehyde (0.80 mL, 14 mmol, 1.4 equiv) was then added. The mixture was stirred and allowed to warm to room temperature

[23] Brice, J. L.; Meerdink, J. E.; Stahl, S. S. *Org. Lett.* **2004**, *6*, 1845–1848.

over a period of 1 hours, and then stirred at room temperature for 1 additional hour. The solids were then removed through rapid filtration. CH_2Cl_2 was then distilled off (rotary evaporator). The crude imine was used directly in the next step with no further purification. To the crude imine was added a solution of di-*tert*-butyl dicarbonate (2.40 g, 11.0 mmol, 1.1 equiv) in toluene (dry; 5.0 mL), followed by triethylamine (1.5 mL, 11 mmol, 1.1 equiv). The resulting yellow solution was stirred at 80 °C for 3 hours. The reaction was stopped and the volatiles were removed under reduced pressure. The resulting crude yellow-orange oil was submitted to column chromatography (SiO_2; Pentane/Et_2O 248/2 to 24/1). The desired product was obtained as a mixture with unreacted Boc_2O. The exceeding Boc_2O was removed as following: the eluate was dissolved in EtOH (5 mL) and imidazole (300 mg) and DMAP (54.0 mg, 0.1 eq compared to imidazole) were added. The resulting mixture was stirred at room temperature for 10 minutes. It was then concentrated under reduced pressure. The crude oil was submitted to column chromatography (Biotage, 24 g SiO_2; Et_2O in pentane, 1 to 5%) to furnish pure *tert*-butyl cyclohexyl(vinyl)carbamate **1i** as a pale yellow oil (0.607 g, 2.45 mmol, 25% yield).

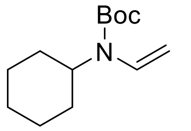

Rf (pentane/Et_2O 95/5) 0.5.
^{1}H NMR (400 MHz, Acetonitrile-*d*$_3$) δ 6.82 (dd, *J* = 16.1, 9.6 Hz, 1H, -*CH*=CH_2), 4.59 (d, *J* = 16.1 Hz, 1H, -CH=*CH$_2$*), 4.26 (d, *J* = 9.6 Hz, 1H, -CH=*CH$_2$*), 3.65 (m, 1H, N*CH*), 2.01 (dd, *J* = 12.6, 3.7 Hz, 2H, *Cy*), 1.84 – 1.74 (m, 2H, *Cy*), 1.68 – 1.57 (m, 3H, *Cy*), 1.46 (s, 9H, $C(CH_3)_3$), 1.33 (qt, *J* = 12.9, 3.7 Hz, 2H, *Cy*), 1.13 (qt, *J* = 13.0, 3.6 Hz, 1H, *Cy*).
^{13}C NMR (101 MHz, Acetonitrile-*d*$_3$) δ 153.6, 134.1, 93.2, 81.1, 55.9, 30.2, 28.1, 26.7, 25.9. **IR** (ν_{max}, cm^{-1}) 2979 (m), 2930 (m), 1704 (s), 1622 (s), 1171 (s), 1146 (s). **HRMS** (APCI/QTOF) m/z: [M + Na]+ Calcd for $C_{13}H_{23}NNaO_2^+$ 248.1621; Found 248.1621.

Benzyl phenethyl(propen-2-yl)carbamate (**1k**)

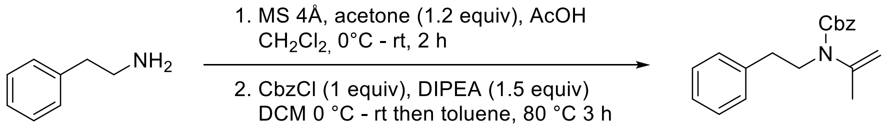

Based on a modified reported procedure, to a solution of 2-phenylethanamine (**29**, 1.6 ml, 13 mmol, 1 equiv), acetic acid (0.14 mL, 2.5 mmol, 0.2 equiv) and 4 Å mol sieves (2.8 g) in CH_2Cl_2 (16 mL) at 0 °C was added propan-2-one (1.0 mL, 14 mmol, 1.2 equiv) dropwise. The solution was warmed to RT and stirred for 14 h and decanted. To the resulting solution at 0 °C were added CH_2Cl_2 (10 mL), benzyl chloroformate (1.9 mL, 13 mmol, 1 equiv) and *N*-ethyl-*N*-*iso*propylpropan-2-amine (3.0 mL, 18 mmol, 1.5 equiv). The reaction mixture was warmed to RT and stirred for 18 h. The solution was concentrated and toluene (10 mL) was added. The solution was heated to 80 °C for 36 h and then concentrated. The crude oil was purified by column chromatography (SiO_2, pentane:Et_2O, 100:0 to 90:10) affording **1k** as a colorless oil (0.351 g, 1.20 mmol, 90% purity, 8% yield).

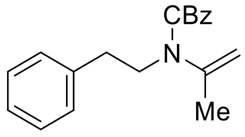

Rf (pentane:EtOAc 9:1) = 0.7.
^{1}H NMR (400 MHz, Acetonitrile-*d*$_3$) δ 7.49 – 7.27 (m, 7H, Ph*H*), 7.28 – 7.19 (m, 3H, Ph*H*), 5.12 (s, 2H, O*CH$_2$*Ph), 4.93 (q, *J* = 1.3 Hz, 1H, C=*CH$_2$*), 4.83 (m, 1H, C=*CH$_2$*), 3.77 – 3.62 (m, 2H, *CH$_2$*), 3.02 – 2.86 (m, 2H, *CH$_2$*), 1.88 (s, 3H, *CH$_3$*).

^{13}C NMR (101 MHz, Acetonitrile-d_3, as a not fully resolved mixture of rotamers) δ 154.9, 145.7, 139.8, 137.7, 129.4, 129.4, 129.2, 129.0, 129.0, 128.8, 128.8, 128.7, 128.5, 128.3, 128.1, 128.1, 127.8, 126.8, 126.8, 110.9, 90.5, 67.2, 67.1, 64.6, 51.1, 46.8, 36.9, 35.2, 27.0, 21.1. **IR** (ν_{max}, cm^{-1}) 2987 (s), 2972 (s), 2959 (s), 2900 (s), 1760 (s), 1699 (s), 1685 (s), 1649 (s), 1403 (s), 1304 (s), 1099 (s), 1067 (s). **HRMS** (APPI/LTQ-Orbitrap) m/z: $[M + H]^+$ Calcd for $C_{19}H_{22}NO_2^+$ 296.1645; Found 296.1636.

(*E*)-3-(hex-1-en-1-yl)oxazolidine-2-one (**1l**)

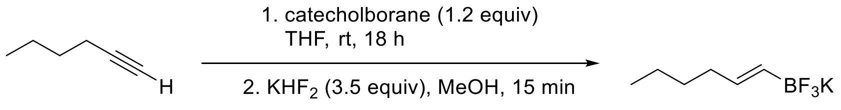

39 **40**

Following a modified reported procedure,[24] in sealed 20 mL, round-bottomed vial, 1-hexyne (**39**, 0.70 mL, 5.9 mmol, 1.0 equiv) and catecholborane (0.77 mL, 7.1 mmol, 1 .20 equiv) were dissolved in THF (dry; 19 mL) and the mixture was stirred under reflux for 18 hours. After this time, the pale yellow solution was allowed to cool down to room temperature, transfered into a 25 mL, round bottomed flask and concentrated under reduced pressure. Water (0.8 mL) was added to the residue, with immediate formation of a colorless solid. The suspension was vigorously stirred for 4 hours at room temperature. The solid was collected by filtration and washed with water. (*E*)-Hex-1-en-1-ylboronic acid (0.469 g, 3.66 mmol, 62% yield) was obtained as a colorless solid, which was directly submitted to the following step.

Following a reported procedure,[25] in a 25 mL, round bottomed flask, (*E*)-hex-1-en-1-ylboronic acid (0.469 g, 3.66 mmol, 1.0 equiv) was dissolved in the minimal volume of MeOH (1.2 mL). A solution of KHF_2 (1.00 g, 12.8 mmol, 3.5 equiv) in water (2.9 mL; 4.5 M) was added slowly, causing the rapid precipitation of a colorless solid. The suspension was stirred at room temperature for 15 minutes. The solid (potassium (*E*)-trifluoro(hex-1-en-1-yl)borate (**40**, 0.244 g, 1.28 mmol, 35% yield) was then collected by filtration and dried under vacuum.

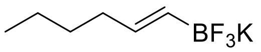

^{1}H NMR (400 MHz, Acetonitrile-d_3) δ 5.62 (dq, *J* = 12.6, 6.0 Hz, 1H, BCH=*CH*), 5.30 (dqd, *J* = 17.6, 3.7, 1.8 Hz, 1H, B*CH*=CH), 1.99 – 1.94 (m, 2H, CH-C*H*$_2$), 1.37 - 1.24 (m, 4H, *CH*$_2$*CH*$_2$), 0.89 (m, 3H, *CH*$_3$). The ^{1}H NMR data corresponds to the reported literature data.[25]

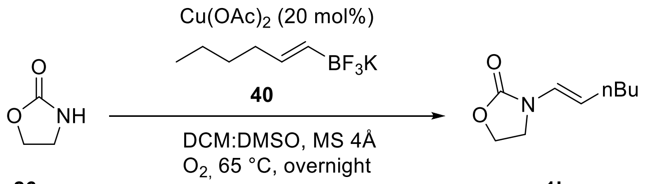

26 **1l**

[24] Perner, R. J.; Lee, C.-H.; Jiang, M., Gu, Y.-G., DiDomenico, S.; Bayburt, E. K.; Alexander, K. M.; Kohlhaas, K. L.; Jarvis, M. F.; Kowaluk, E., L.; Bhagwat, S. S. *Bioorg. Med. Chem. Lett.* **2005**, *15*, 2803.
[25] Batey R. A.; Thadani, A. N.; Smil, D. V. *Org. Lett.* **1999**, *1*, 1683.

Following a reported procedure,[26] in a sealed 5.0 mL round bottomed test tube, potassium (*E*)-trifluoro(hex-1-en-1-yl)borate **40** (0.244 g, 1.28 mmol, 1.7 equiv), oxazolidin-2-one (0.066 g, 0.76 mmol, 1.0 equiv), and copper(II) acetate (0.027 g, 0.15 mmol, 0.20 equiv) were dissolved in a mixture of CH_2Cl_2 (dry; 1.5 mL) and DMSO (dry; 1.5 mL) in the presence of MS (4Å; 0.60 g). The resulting blue suspension was stirred overnight at 65 °C under an atmosphere of oxygen (balloon). After 20 hours, it was allowed to cool down to room temperature and filtered through a plug of celite, which was then washed with several portions of EtOAc. The resulting filtrate was concentrated under reduced pressure and submitted to column chromatography (still retaining DMSO) (SiO_2; EtOAc in pentane 5 to 20%). (*E*)-3-(Hex-1-en-1-yl)oxazolidin-2-one (**1ka**, 0.081 g, 0.48 mmol, 63% yield) was obtained as a colorless oil.

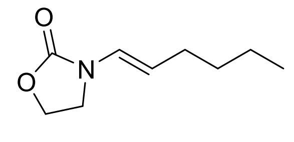

^{1}H NMR (400 MHz, Chloroform-*d*) δ 6.64 (d, *J* = 14.3 Hz, 1H, N*CH*=CH-), 4.80 (dt, *J* = 14.2, 7.1 Hz, 1H, NCH=*CH*-), 4.42 (m, 2H, *CH_2* in the oxazolidinone cycle), 3.72 - 3.60 (m, 2H, *CH_2* in the oxazolidinone cycle), 2.06 (qd, *J* = 7.1, 1.4 Hz, 2H, CH=CH*CH_2*), 1.44 - 1.26 (m, 4H, *CH_2CH_2*), 0.90 (t, *J* = 7.1 Hz, 3H, CH_3).

^{13}C NMR (101 MHz, Chloroform-*d*) δ 155.4, 123.8, 111.4, 62.1, 42.6, 32.2, 29.4, 22.1, 13.9.

The characterisation data corresponds to the reported literature data.[26]

(*Z*)-3-(hex-1-en-1-yl)oxazolidine-2-one (**3l'**)

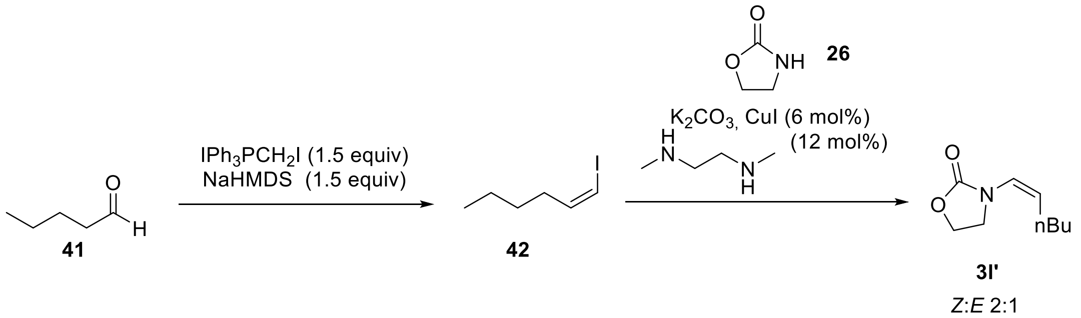

Following a reported procedure,[27] under nitrogen atmosphere, iodo(iodomethyl)triphenylphosphorane (5.16 g, 9.25 mmol) was suspended in THF (13 mL). At room temperature, sodium bis(trimethylsilyl)amide (2 M in THF, 4.6 mL, 9.3 mmol, 1.5 equiv) was added dropwise over 5 min. The reaction was stirred for 30 min then cooled to -78 °C. A solution of valeraldehyde (**41**, 0.658 mL, 6.16 mmol, 1.0 equiv) in THF (13 mL) was added dropwise over 15 min. The reaction was then stirred at -78 °C for 1 h then quenched with ammonium chloride (0.561 g, 10.5 mmol) dissolved in 10 mL water then warmed to RT. The crude was diluted with Et_2O (20 mL) and brine:water (1:1, 20 mL). The layers were separated, the aqueous layers were extracted with Et_2O (5x30 mL). The organic layer was washed with $NaHCO_3$ sat (2*20 mL), then brine (20 mL). The organic layers were combined and concentrated under reduced pressure. The compound was purified by column chromatography: SiO_2 using pentane affording (*Z*)-1-iodohex-1-ene (**42**, 0.830 g, 3.95 mmol, 64 % yield). The later was used directly in the next step. Following a reported literature procedure, an oven-dried pointed 2 mL microwave vial was charged with oxazolidin-2-one (**26**, 0.237 g, 2.72 mmol, 1 equiv) and potassium carbonate (0.414 g, 2.99 mmol, 1.2 equiv). Under the inert atmosphere of a glove

---

[26] Bolshan, Y.; Batey, R. *Angew. Chem. Int. Ed.* **2008**, *47*, 2109.
[27] Selter, L. Harms, K.; Koert, U. *Eur. J. Org. Chem.* **2017**, 1215.

box copper(I) iodide (0.036 g, 0.19 mmol, 6 mol%) was added and the vial was sealed. *N,N'*-dimethylethylenediamine (0.041 mL, 0.38 mmol, 12 mol%) and a solution of (*Z*)-1-iodohex-1-ene (0.80 g, 3.8 mmol, 1.4 equiv) in anhydrous toluene (0.9 mL) were added *via* syringe. The resulting greenish suspension was stirred at 110-115 °C for 20 hours. The solution colored a darkish orange. The crude was filtered over a celite pad and washed with CH_2Cl_2. The crude oil was submitted to column chromatography (SiO_2; EtOAc in pentane, 24/1 to 80/20) to afford 3-(hex-1-en-1-yl)oxazolidin-2-one as a mixture of Z and E isomers (**3l'** and **3l**, 0.340 g, 0.200 mmol, 74% yield, Z:E ratio 2:1)

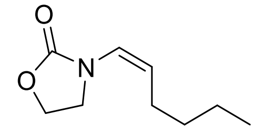

Characterisation of the Z :E mixture :
Rf (pentane :EtOAc 6 :4) = 0.4.
^{1}H NMR (400 MHz, Benzene-d_6, 2 :1 mixture of (*Z*) in **bold** and (*E*) alkene in *italic*) δ *7.01 – 6.78* (m, 0.5H, RC*H*=CHR, (*E*)-isomer), **6.44** (dt, *J* = 9.7, 1.6 Hz, 1H, RC*H*=CHR, (*Z*)-isomer), **4.46** (dt, *J* = 9.7, 7.6 Hz, 1H, RC*H*=CHR, (*Z*)-isomer), *4.32* (dd, *J* = 14.3, 7.1 Hz, 0.5H, RC*H*=CHR, (*E*)-isomer), ***3.48 – 3.01*** (m, 3H, cyclic-CH_2, (*E*)-isomer + (*Z*)-isomer), **2.85 – 2.58** (m, 2H, cyclic-CH_2, (*Z*)-isomer), *2.45 – 2.28* (m, 1H, cyclic-CH_2, (*E*)-isomer), *1.87* (tdt, *J* = 7.1, 4.4, 1.5 Hz, 1H, allylic-CH_2, (*E*)-isomer), **1.81** (tdt, *J* = 7.4, 5.8, 1.7 Hz, 2H, allylic-CH_2, (*Z*)-isomer), *1.30 – 1.20* (m, 2H, CH_2-CH_2-CH_3, (*E*)-isomer), **1.19** (m, 4H, CH_2-CH_2-CH_3, (*Z*)-isomer), ***0.93 – 0.81*** (m, 2+3H, CH_3, (*E*)-isomer + (*Z*)-isomer). **^{13}C NMR** (101 MHz, Benzene-d_6, the C=O of (*E*)-isomer is not resolved) δ 156.9, 125.5, 123.9, 113.3, 110.3, 61.9, 61.8, 45.3, 42.4, 33.4, 33.4, 30.3, 26.8, 23.1, 23.0, 14.7. **IR** (ν_{max}, cm^{-1}) 2959 (m), 2931 (m), 2871 (m), 1752 (s), 1415 (s), 1243 (m), 1094 (s), 1075 (s), 1040 (m). **HRMS** (nanochip-ESI/LTQ-Orbitrap) m/z: $[M + H]^+$ Calcd for $C_9H_{16}NO_2^+$ 170.1176; Found 170.1177.

(*E*)-((hex-1-en-1-yloxy)methyl)benzene

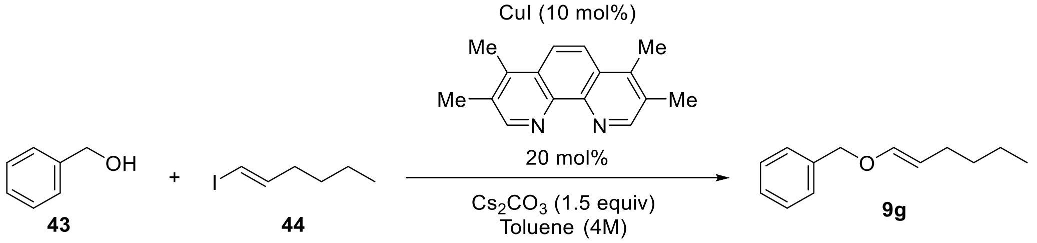

Following a slightly modified reported procedure,[28] inside a glove box, a 5 mL test tube was charged with CuI (0.038 g, 0.10 mmol, 10 mol%), 3,4,7,8-tetramethyl-1,10-phenanthroline (0.94.6 mg, 0.200 mmol, 20 mol%) and Cs_2CO_3 (0.977 g, 3.00 mmol, 1.5 equiv). The tube was sealed and withdrawn from the glovebox. Toluene (dry, 1.0 mL), followed by benzyl alcohol (**43**, 0.41 mL, 2.0 mmol, 2 equiv) and *E*-1-iodohexene (**44**, 0.210 g, 1.00 mmol, 1 equiv) were then added via syringe. The reaction mixture was heated to 80 °C for 8 hours. After this time TLC analysis showed full consumption of the iodo-alkene. The reaction was stopped, the now brown suspension was allowed to cool down to room temperature, and the solids were then filtered off through a short pad of SiO_2, which was washed with several portions of CH_2Cl_2. The resulting brown-orange solution was concentrated under reduced pressure. The so obtained

[28]Nordmann, G.; Buchwald, S. L. *J. Am. Chem. Soc.* **2003**, *125*, 4978.

brown crude oil was submitted to column chromatography (Biotage, 12 g SiO_2; CH_2Cl_2 in pentane, 1 to 10%) to afford (*E*)-((hex-1-en-1-yloxy)methyl)benzene (**9g**, 0.253 g, 1.32 mmol, 66% yield) as a colorless oil.

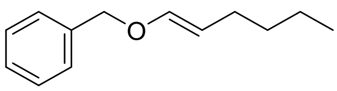

Rf (pentane: CH_2Cl_2 96:4) = 0.6.
^{1}H NMR (400 MHz, Chloroform-*d*) δ 7.40 – 7.34 (m, 4H, Ph*H*), 7.31 (m, 1H, Ph*H*), 6.34 (dt, *J* = 12.5, 1.4 Hz, 1H, OC*H*=CH-), 4.90 (dt, *J* = 12.5, 7.3 Hz, 1H, OCH=*CH*-), 4.72 (s, 2H, Ph*CH₂*O), 1.94 (dtt, *J* = 8.5, 7.2, 1.4 Hz, 2H, CH=CH*CH₂*), 1.40 – 1.25 (m, 4H, *CH₂CH₂*), 0.90 (m, 3H, *CH₃*).
^{13}C NMR (101 MHz, Chloroform-*d*) δ 145.8, 137.4, 128.5, 127.8, 127.6, 105.3, 71.1, 32.8, 27.4, 22.1, 13.9. **IR** (ν_{max}, cm^{-1}) 3011 (m), 2998 (m), 2970 (s), 2955 (s), 2923 (s), 2902 (s), 2850 (m), 1672 (m), 1455 (m), 1380 (m), 1258 (m), 1213 (s), 1153 (s), 1124 (s), 1075 (s), 1046 (s), 1038 (s). **HRMS** (APPI/LTQ-Orbitrap) m/z: $[M + H]^+$ Calcd for $C_{13}H_{19}O^+$ 191.1430; Found 191.1433.

Ene-carbamate and enol-ether oxyalkynylation

Optimisation of the reaction conditions and control reactions:

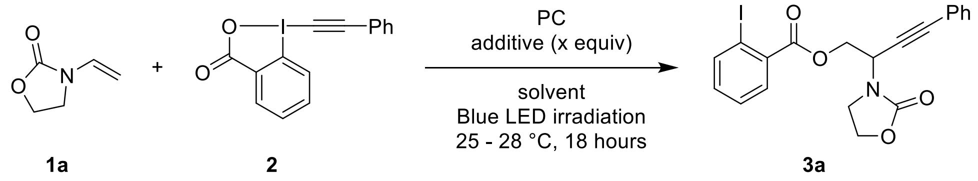

An oven-dried screw-top vial was charged with PC (5 µmol, 5 mol%), PhEBX (**2**, 0.10 mmol, 1.0 equiv) and additive (X equiv). After 3 vacuum/N_2 cycles, dry degassed (*via* freeze-pump-thaw technique) solvent was added (1.1 mL, 0.097 M; unless specified) followed by **1a** (1.2 equiv). The reaction was sealed under a flow of Argon and the reaction vessel was placed under irradiation (blue leds) and stirred overnight (18 hours). After this time, the conversion of PhEBX could be observed by TLC analysis (Pent:EtOAc, 7:3, p-anisaldehyde stain, blue-green spot). The reaction mixture was concentrated under reduced pressure. CH_2Br_2 (7 µL, 1.0 equiv) were added as an internal standard and the crude was solubilised immediately in Chloroform-*d*.

$[Ir(dF(CF_3)ppy)_2(tbbpy)]PF_6$: **45**

Table 1: Optimisation of the reaction conditions

entry	PC	Additive (x equiv)	Solvent (M)	yield (%)	comments
1	**4a**	None	DCE	30	
2	**4b**	None	DCE	42	
3	**5**	None	DCE	5	
4	**4b**	None	DCE	36-65	
5[b]	**4b**	None	DCE	34	Recrystalised Ph-EBX
6	**4b**	BIOH **6** (1.5 equiv)	DCE (0.1 M)	46	Recrystalised Ph-EBX
7	**4b**	BIOAc **7** (1.5 equiv)	DCE (0.1 M)	70	Recrystalised Ph-EBX
8	**4b**	BIOAc **7** (1.5 equiv)	DCE (0.1 M)	20	With 1-iodo-2-phenylacetylene instead of **2**
9	**4b**	BIOAc **7** (1.0 equiv)	DCE (0.1 M)	73	Recrystalised Ph-EBX
10	**4b**	BIOAc **7** (0.5 equiv)	DCE (0.1 M)	75	Recrystalised Ph-EBX
11	**4b**	BIOAc **7** (0.5 equiv)	DMSO (0.1 M)	75	Recrystalised Ph-EBX
12	**4b**	BIOAc **7** (0.5 equiv)	CH_2Cl_2 (0.1 M)	80	Recrystalised Ph-EBX
13	**4b**	BIOAc **7** (0.5 equiv)	CH_2Cl_2 (0.25 M)	80	Recrystalised Ph-EBX
14	**4b**	BIOAc **7** (0.5 equiv)	CH_2Cl_2 (0.5 M)	80	Recrystalised Ph-EBX
15	**4b**	BIOAc **7** (0.5 equiv)	CH_2Cl_2 (0.25 M)	80	Recrystalised Ph-EBX, **4b**: 2 mol% **1a** (1.5 equiv)
16	**4b**	BIOAc **7** (0.5 equiv)	CH_2Cl_2 (0.25 M)	80	Recrystalised Ph-EBX, **4b**: 2 mol%, **1a** (1.5 equiv)
17	**8**	BIOAc **7** (0.5 equiv)	CH_2Cl_2 (0.25 M)	21	Recrystalised Ph-EBX, **7**: 2 mol%**1a** (1.5 equiv)
18	**45**	BIOAc **7** (0.5 equiv)	CH_2Cl_2 (0.25 M)	24	Recrystalised Ph-EBX, **45**: 2 mol%**1a** (1.5 equiv)
19	**4b**	BIOAc **7** (0.05 equiv)	CH_2Cl_2 (0.25 M)	80	Recrystalised Ph-EBX, **4b**: 2 mol%**1a** (1.5 equiv)

Control experiments and mechanistic studies

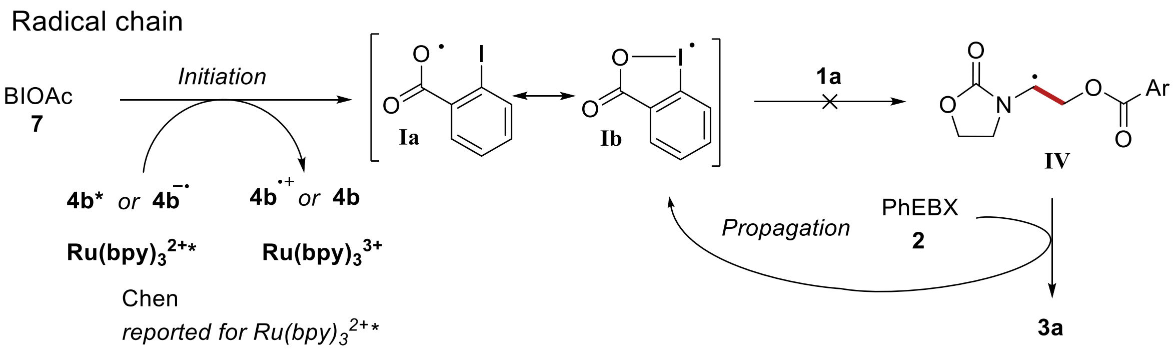

Scheme S1. ATRA mechanism for the addition of EBX **2** to enamide **1c**.

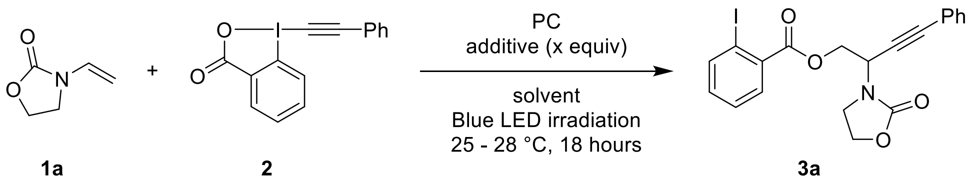

An oven-dried screw-top vial was charged with PC (5 µmol, 5 mol%), PhEBX (**2**, 0.10 mmol, 1.0 equiv) and additive (X equiv). After 3 vacuum/N_2 cycles, dry degassed (*via* freeze-pump-thaw technique) solvent was added (1.1 mL, 0.097 M; unless specified) followed by **1a** (1.2 equiv). The reaction was sealed under a flow of Argon and the reaction vessel was placed under irradiation (blue leds) and stirred overnight (18 hours). After this time, the conversion of PhEBX could be observed by TLC analysis (Pent:EtOAc, 7:3, p-anisaldehyde stain, blue-green spot). The reaction mixture was concentrated under reduced pressure. CH_2Br_2 (7 µL, 1.0 equiv) were added as an internal standard and the crude was solubilised immediately in Chloroform-*d*.

$[Ru(bpy)_3](PF_6)_2$: **46**

Table 2: Control experiments

entry	PC	Additive (x equiv)	Solvent (M)	yield (%)	comments
1	**4b**	BIOAc **7** (0.5 equiv)	DCE	-	No light
2	-	BIOAc **7** (0.5 equiv)	DCE	-	Light, no conversion observed
3	-	BIOAc **7** (0.5 equiv)	DCE	-	No light
4	**5**	BIOAc **7** (0.5 equiv)	CH_2Cl_2	10-15	80% conversion of starting alkene
5	**46**	BIOAc **7** (0.5 equiv)	CH_2Cl_2	-	Performed with **1c**. No conversion observed
6	**4b**	None	CH_2Cl_2	65	Reaction time: 5 days. 20% residual PhEBX,

Control experiment eq. c:

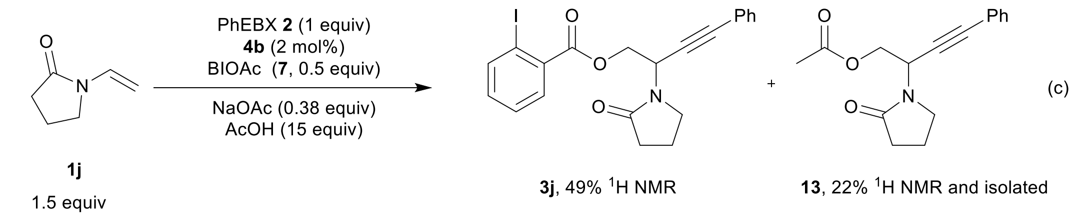

Following the general procedure for difunctionalisation, an oven dried 1.5 mL screw cap vial, equipped with a magnetic stirrer was charged with, PhEBX (**2**, 0.035 g, 0.10 mmol, 1.00 equiv), BIOAc (**7**, 0.015 g, 0.050 mmol, 0.5 equiv), sodium acetate (0.003 g, 0.004 mmol, 0.4 equiv) and 4-ClCzIPN (**4b**, 2 mg, 2 µmol, 2 mol%). The vial was sealed with a septum and flushed with Ar for 5 min. Degassed DCM (0.4 mL, 2.5 M) was added followed by acetic acid (86 µL, 1.5 mmol, 15.0 equiv) and N-vinyl pyrolidinone (**1j**, 16 µL, 0.015 mmol, 1.50 equiv). The reaction was irradiated with Blued LED strips for 15 h under agitation. The reaction was concentrated under vaccum, solubilised in $CDCl_3$ and 1 equiv of CH_2Br_2 was added (7 uL) to determine the crude ^{1}H NMR ratio of **3j** and **13** (**3j**:**13** 5:2 ^{1}H NMR). A triethylamine deactivated silica solid deposit of the crude was prepared and the latter was submitted to column chromatography (SiO_2, 15 g) pentane:EtOAc 7:3 to 4:6.affording **3j** (0.016 g, 0.035 mmol, 35% yield) and **13** (0.009 g, 60% purity, 0.02 mmol, 22% yield) as pale yellow oils.

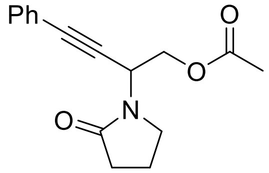

13 characterised as a mixture of iodobenzoic acid, **3j** and residual starting material **1j**. Purity 60%.

Rf (pentane:EtOAc 1:1) =0.3. **^{1}H NMR** (400 MHz, Chloroform-*d*) δ 7.46 – 7.37 (m, 2H, Ph), 7.37 – 7.28 (m, 3H, Ph), 5.46 (dd, *J* = 8.6, 4.8 Hz, 1H, N-C*H*), 4.37 (dd, *J* = 11.3, 8.6 Hz, 1H, O-C*H*$_2$), 4.25 (dd, *J* = 11.3, 4.8 Hz, 1H, O-C*H*$_2$), 3.65 (ddd, *J* = 9.4, 7.8, 6.4 Hz, 1H, N-C*H*$_2$), 3.59 – 3.46 (m, 1H, N-C*H*$_2$), 2.47 – 2.38 (m, 2H, cyclic-C*H*$_2$), 2.11-2.03 (m, 5H, COC*H*$_3$ + cyclic-C*H*$_2$). **^{13}C NMR** (101 MHz, Chloroform-*d*) δ 175.0, 170.6, 141.5, 131.9, 128.8, 128.4, 86.0, 82.2, 63.0, 43.6, 43.5, 31.0, 20.8, 18.0. **IR** (ν_{max}, cm^{-1}) 2983 (s), 2958 (s), 2925 (s), 2853 (s), 1745 (s), 1692 (s), 1491 (s), 1462 (s), 1420 (s), 1268 (s), 1229 (s), 1044 (s), 913 (s). **HRMS** (nanochip-ESI/LTQ-Orbitrap) m/z: $[M + H]^+$ Calcd for $C_{16}H_{18}NO_3^+$ 272.1281; Found 272.1286.

^{1}H NMR (400 MHz) of the crude mixture:

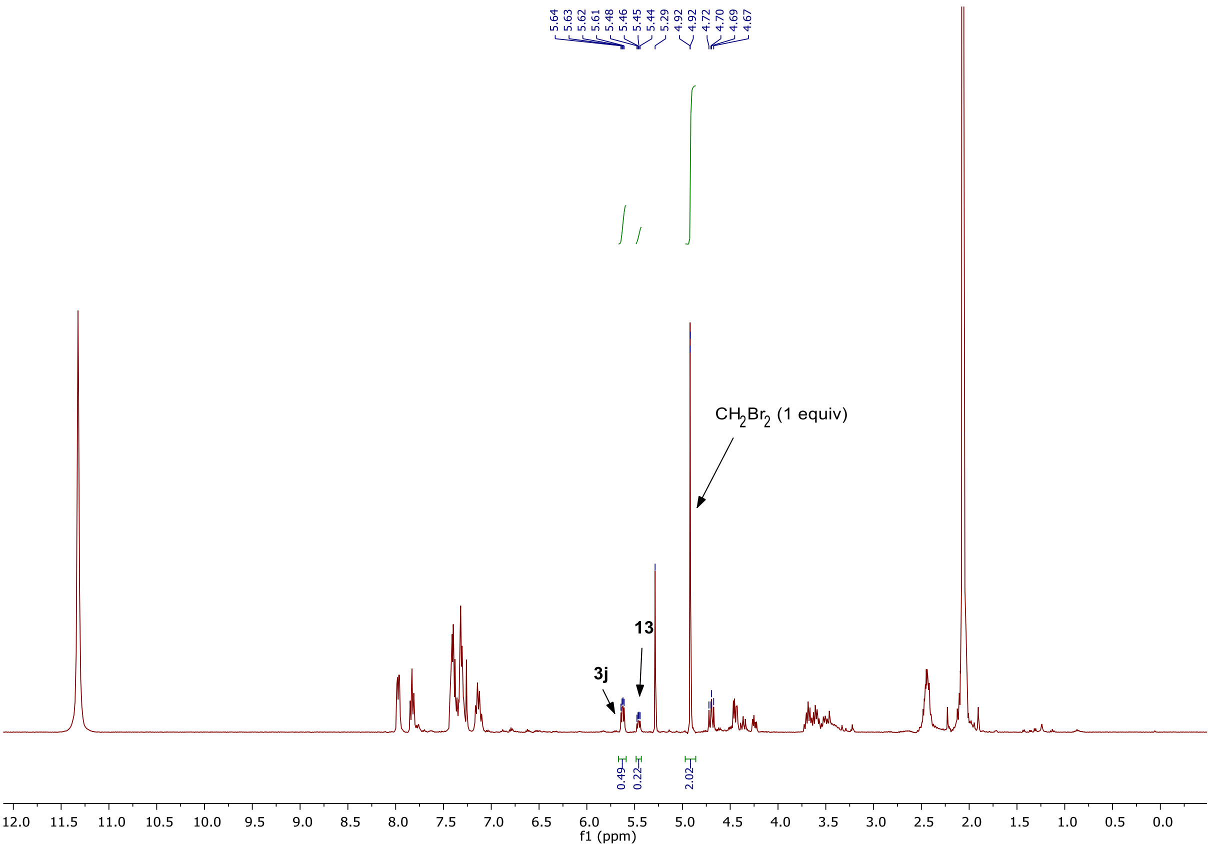

Electrochemical experiments

An Autolab potentiostat with a 3 electrode cell configuration: glassy carbon (working electrode), Pt wire as (control electrode), and Ag/AgCl (KCl, 3M aq.) as (reference electrode) was used for the measures. Tetrabutyl ammonium hexafluorophosphhate (TBAP, 0.1M in MeCN) was used as an electrolyte. The sample (0.01 mmol) was dissolved in a stock solution of TBAP (0.1 M, 10 mL in MeCN) and was degassed by bubbling Argon directly before measure. The redox couple E(alkene$^{+\bullet}$/alkene) is defined as the potential E measured for $\frac{I_{max}}{2}$.

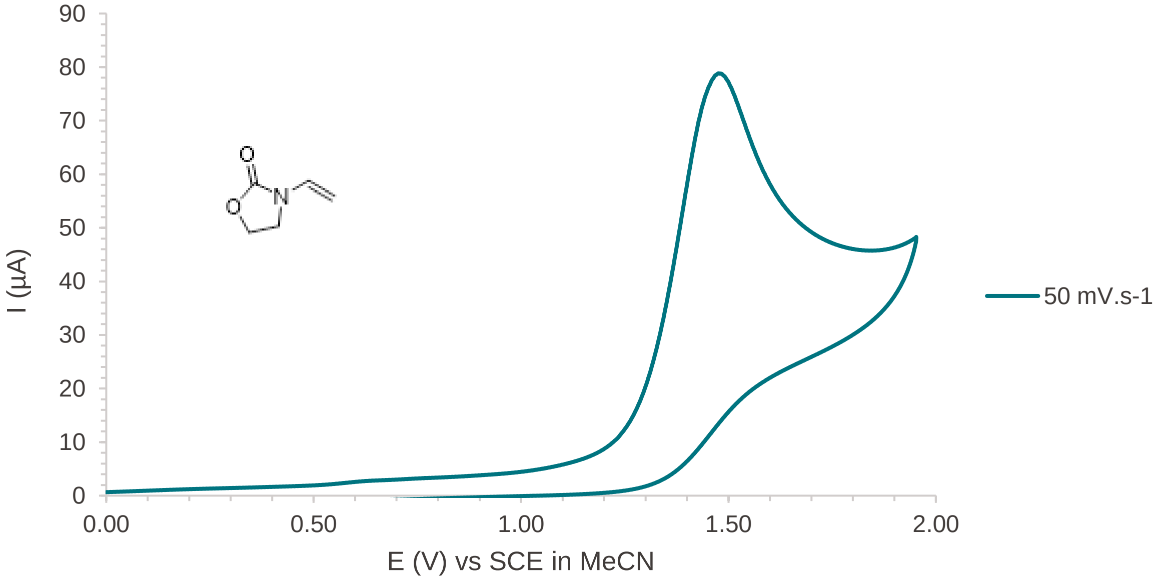

Graph S1. Cyclic voltagram of **1a**

$$I_{max} = 78\ \mu A;\ \frac{I_{max}}{2} = 39\ \mu A\ ; E = 1.30\ V \text{ so that } I = 39\ \mu A$$

E(**1a**$^{+\bullet}$/**1a**) = +1.30 V

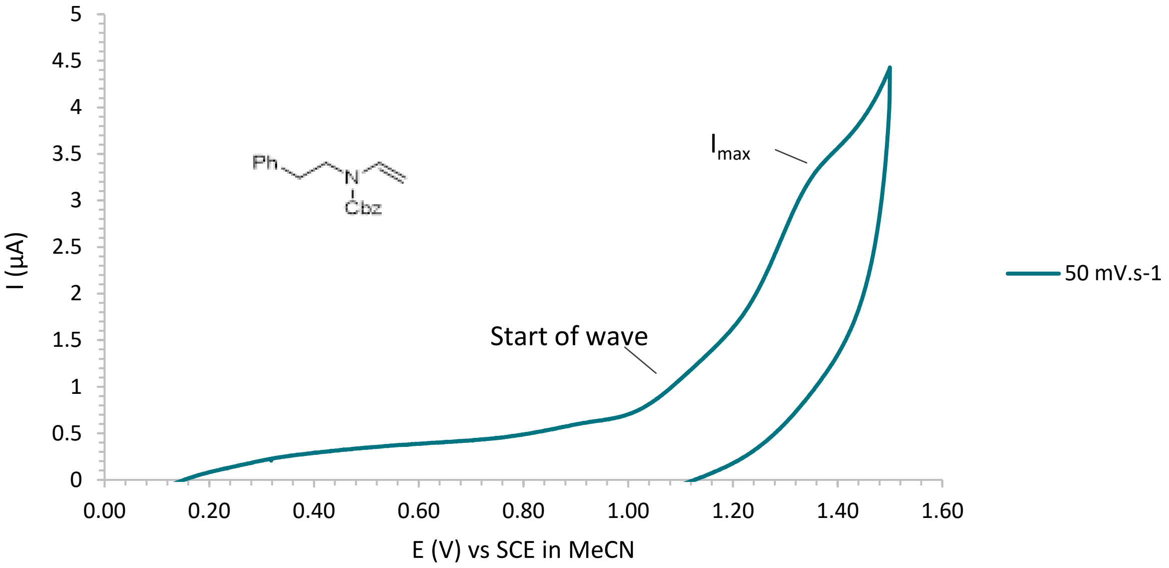

Graph S2. Cyclic voltagram of **1c**

$$I_{max} = 3.24\ \mu A;\ I_{start\ of\ wave} = 0.78\ \mu A;\ \frac{I_{max} - I_{start\ of\ wave}}{2} = 2.46\ \mu A;$$

$$E = 1.28 \text{ V so that I} = 2.46\ \mu\text{A}$$

$$\mathrm{E}(\mathbf{1c}^{+\bullet}/\mathbf{1c}) = +1.28 \text{ V}$$

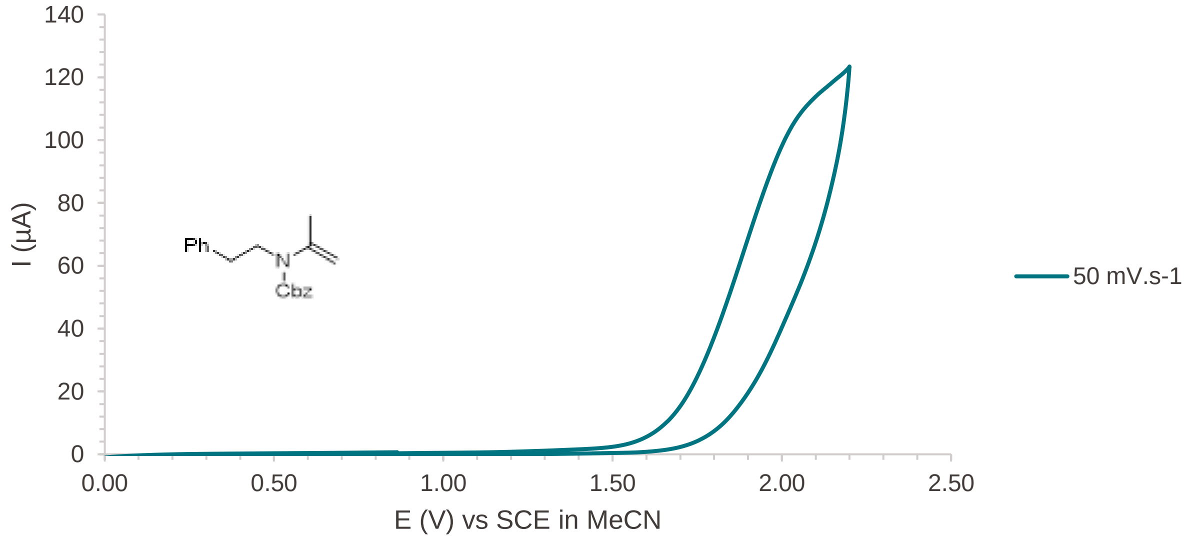

Graph S3. Cyclic voltagram of **1k**

$$\mathrm{I_{max}} \approx 110\ \mu\text{A};\ \frac{\mathrm{I_{max}}}{2} \approx 55\ \mu\text{A}\ ;\ \mathrm{E} = 1.86 \text{ V so that I} = 55\ \mu\text{A}$$

$$\mathrm{E}(\mathbf{1k}^{+\bullet}/\mathbf{1k}) \approx +1.86 \text{ V}$$

Stern Volmer quenching expermients

Stern-Volmer fluorescence quenching experiments were conducted on a Varian Cary Eclipse machine.

A degassed stock solution of 48 µM of photocatalyst **4b** in DCM was prepared. 0.5 mL of this solution was placed in a 0.5 mL fluorimeter cuvette. Progressively, the quencher **1a** or **1k** was added via Hamilton syringe in portions of 5 µL or 10 µL between each addition the fluorescence spectra was measured.

The emission intensity was recorded at 527 nm.

Important note: Pre-prepared stock solutions of quencher and photocatalyst gave poorly reproducible results, it was found that the fluorescence spectra were the most reproducible when adding pure **1a** or **1k**, for this reason (in addition to the poor solubility of BIOAc) the fluorescence quenching spectra for **7** were not measured. The concentrations for the Stern-Volmer plots were calculated based on approximate volumetric masses of **1a** and **1k** at RT and ambient pressure. 40 µL of **1a** weigh 49 µg at RT and ambient pressure. Approximated volumetric mass was estimated to be 1.2 g/mL. 40 µL of **1k** weigh 42 µg Approximated volumetric mass was estimated to be 1.1 g/mL.

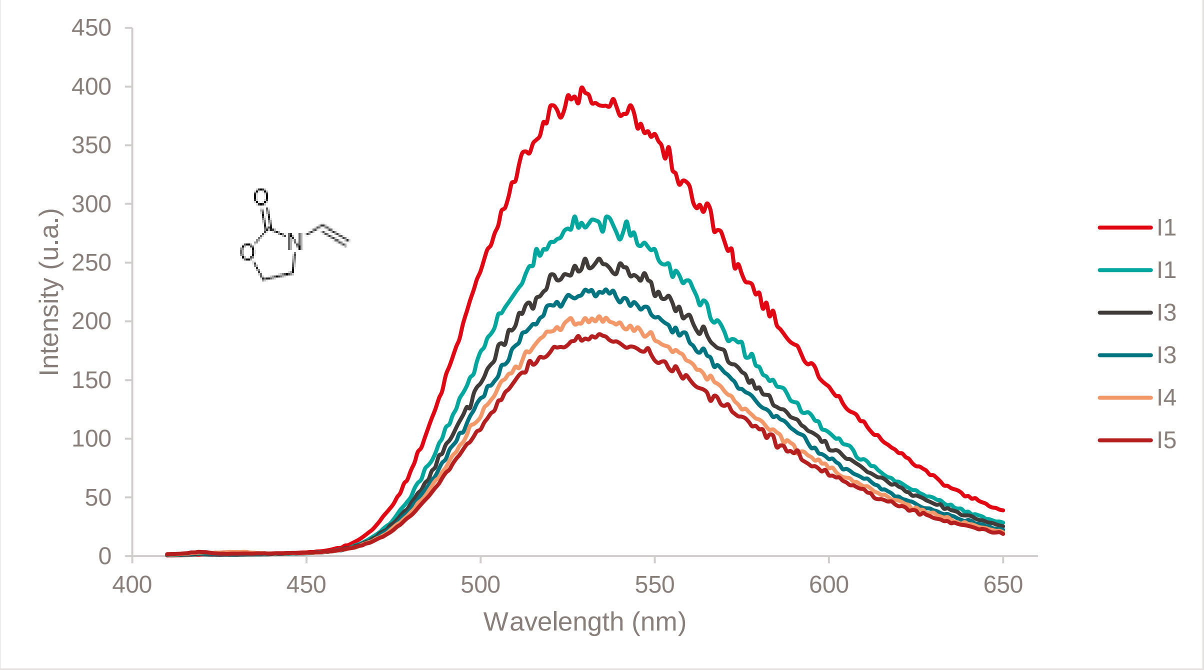

Graph S4. Fluorescence emission spectra of **4b**: fluorescence quenching by **1a**.

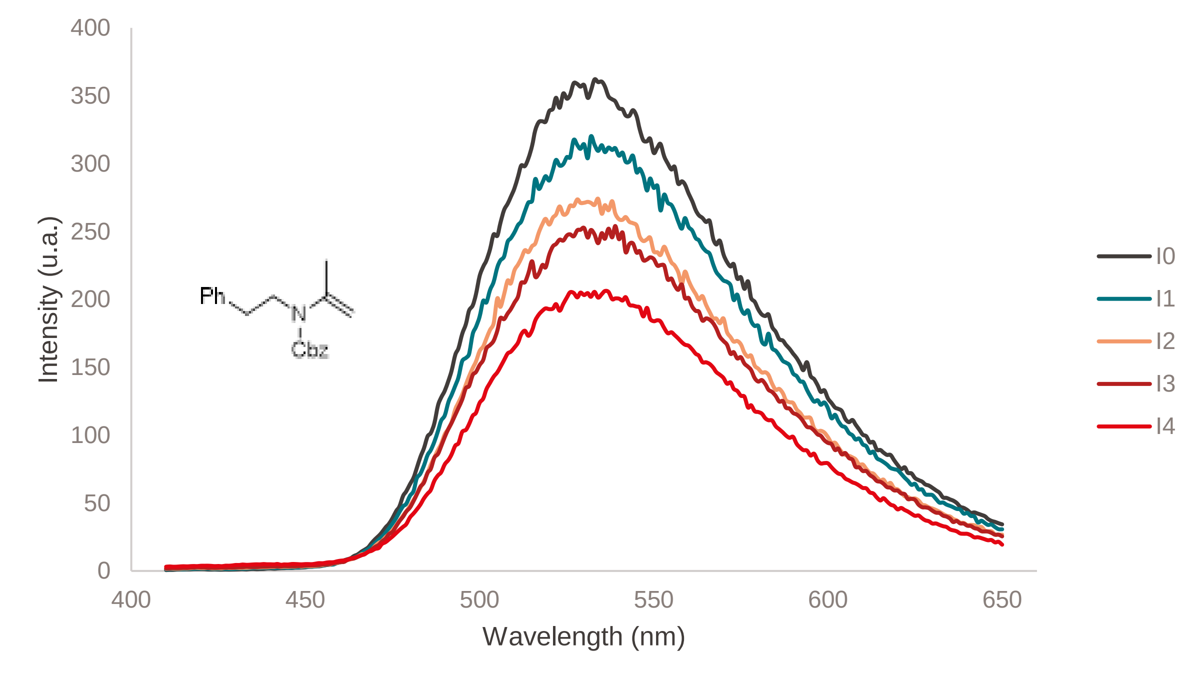

Graph S5. Fluorescence emission spectra of **4b**: fluorescence quenching by **1k**.

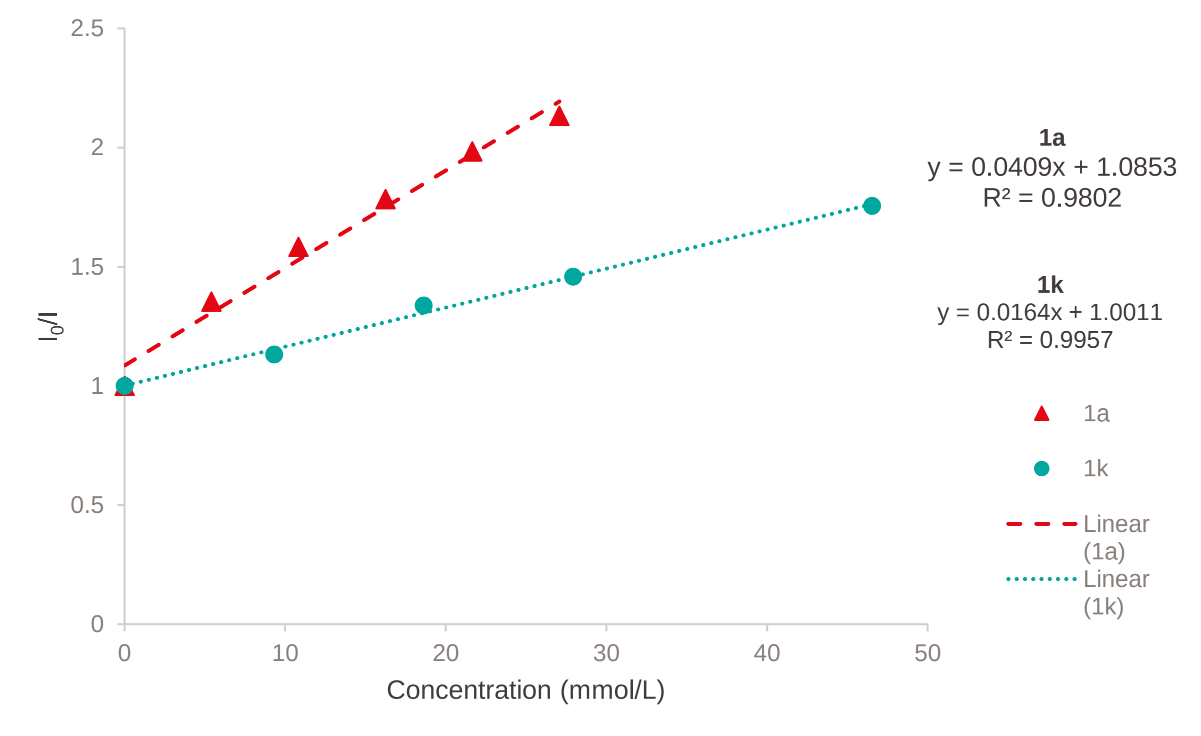

Graph 6. Stern-Volmer plot for **1a** and **1k**.

Interestingly **1k** also quenches the photocatalyst however the process is non-productive (no conv. with **1k**).

Experimental set-up

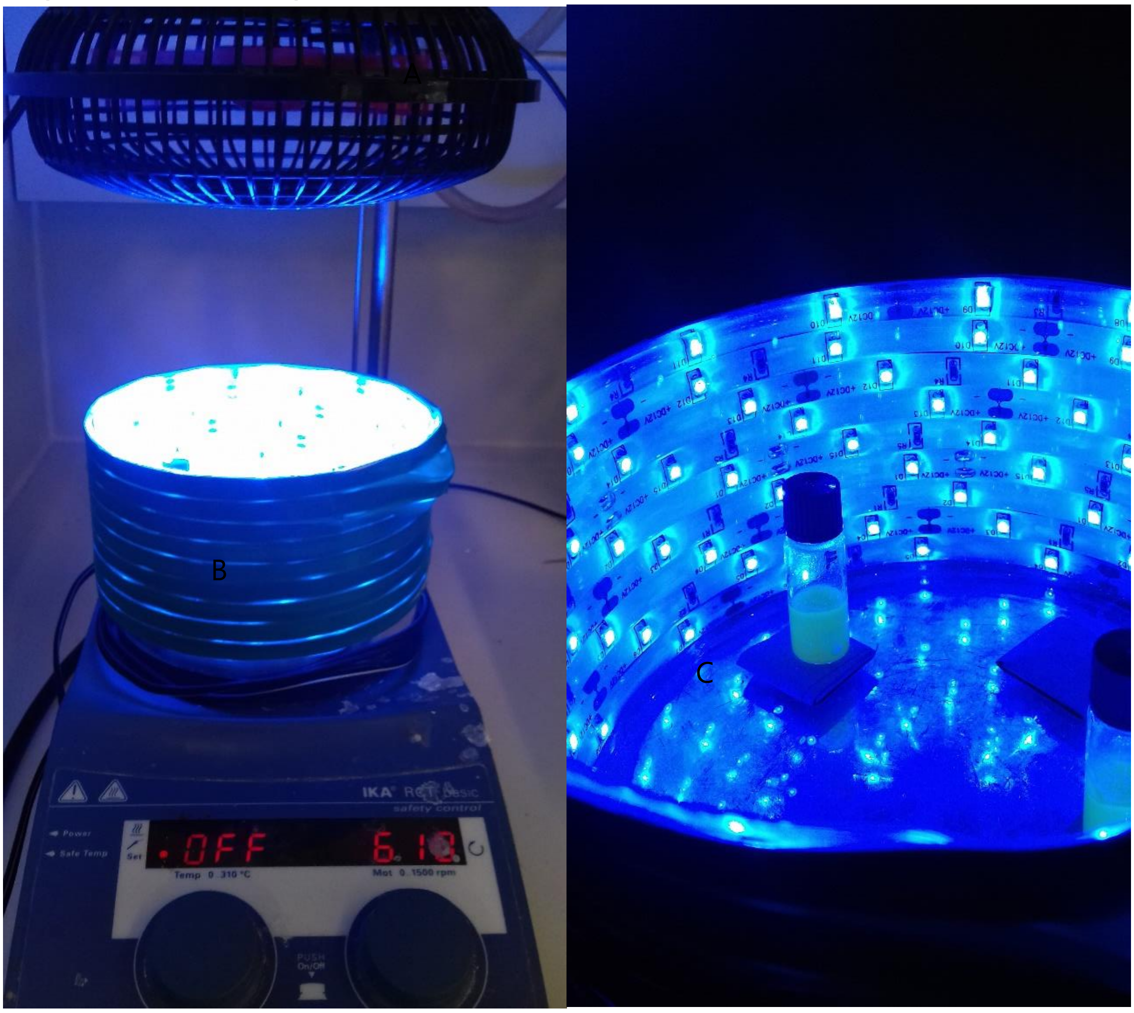

A : Overtop ventilation, B : crystalisation bowl wrapped with LEDs. C flask stuck to bottom of crystalisation bowl ca. 3 cm away from LEDs. The whole set up is concealed from the external light sources with a reflective shield.

General procedure for scope scale reactions:

An oven-dried flat-bottomed screw-cap vial equipped with a magnetic stirrer was charged with R-EBX (0.25 mmol, 1.0 equiv), BIOAc (0.13 mmol, 0.5 equiv) and 4-ClCzIPN (**4b**, 5 mg, 5 μmol, 2 mol%). The reaction vessel was sealed with a rubber septum. Following three vacuum/nitrogen cycles, dry, degassed (*via* freeze-pump-thaw technique) CH_2Cl_2 (0.25 M based on R-EBX, 1.0 mL) was then added. The substrate (0.38 mmol, 1.5 equiv)[29] was then added *via* syringe. The rubber septum was replaced with the corresponding screw-cap under a flux of Argon. The reaction was irradiated overnight (15 h-18 h) with blue LED strips under ventilation (T = 25°C) and stirring. The volatiles were evaporated off. The crude was then dissolved in CH_2Cl_2 with 0.2 mL of Et_3N. A solid deposit for flash chromatography was prepared with SiO_2 (ca. 3g). The crude was then purified though flash chromatography (Et_3N deactivated SiO_2 ca. 40 g or biotage : SiO_2 25 g, Pentane:Et_2O or Pentane:EtOAc)[30] affording the desired difunctionalised product.

Characterisation data

2-(2-Oxooxazolidin-3-yl)-4-phenylbut-3-yn-1-yl 2-iodo benzoate (**3a**)

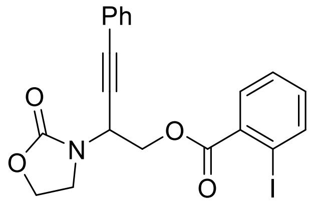

Performed on 0.2 mmol scale.
Obtained from *N*-vinyloxazolidin-2-one (**1a**, 0.027 g, 0.24 mmol, 1.5 equiv); PhEBX (**2**, 0.070 g, 0.20 mmol, 1.0 equiv); BIOAc (**7**, 0.031 g, 0.10 mmol, 0.5 equiv) and 4-ClCzIPN (**4b**, 4 mg, 4 μmol, 2 mol%); CH_2Cl_2 (0.8 mL, 0.25 M) after 18 hours.
Column: Pentane:EtOAc, 9:1 to 8:2. Yield 80% (0.074 g, 0.16 mmol). Yellow oil with residual grease.

Rf (Pentane:EtOAc 8:2) = 0.3.
^{1}H NMR (400 MHz, Chloroform-*d*) δ 8.00 (dd, *J* = 7.9, 1.2 Hz, 1H, Ar*H*), 7.84 (dd, *J* = 7.8, 1.7 Hz, 1H, Ar*H*), 7.44 (dd, *J* = 7.9, 1.8 Hz, 2H, Ph*H*), 7.41 (dd, *J* = 7.7, 1.2 Hz, 1H, Ar*H*), 7.38 - 7.31 (m, 3H, Ph*H*), 7.17 (ddd, *J* = 7.9, 7.4, 1.7 Hz, 1H, Ar*H*), 5.37 (dd, *J* = 9.0, 4.4 Hz, 1H, NC*H*), 4.77 (dd, *J* = 11.5, 9.0 Hz, 1H, OC*H₂*), 4.47 (dd, *J* = 11.5, 4.4 Hz, 1H, OC*H₂*), 4.44 - 4.33 (m, 2H, C*H₂*), 3.85 (dt, *J* = 9.2, 8.1 Hz, 1H, C*H₂*), 3.78 (td, *J* = 8.6, 5.6 Hz, 1H, C*H₂*). **^{13}C NMR** (101 MHz, Chloroform-*d*) δ 165.9, 158.0, 141.4, 134.2, 133.1, 131.9, 131.3, 129.2, 128.5, 128.2, 121.5, 94.3, 87.5, 80.9, 63.8, 62.4, 46.2, 41.2. **IR** (ν_{max}, cm^{-1}) 3060 (w), 2993 (w), 2920 (w), 2851 (w), 2229 (w), 1750 (s), 1487 (m), 1422 (m), 1249 (s). **HRMS** (ESI) calcd for $C_{20}H_{17}INO_4^+$ $[M+H]^+$ 462.0197; found 462.0206.

2-((T*ert*-butoxycarbonyl)(phenethyl)amino)-4-phenylbut-3-yn-1-yl 2-iodobenzoate (**3b**)

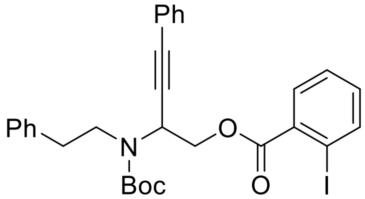

Performed on 0.2 mmol scale.
Obtained from *tert*-butyl phenethyl(vinyl)carbamate (**1b**, 0.054 g, 0.24 mmol, 1.2 equiv); PhEBX (**2**, 0.070 g, 0.20 mmol, 1.0 equiv); BIOAc (**7**, 0.031 g, 0.10 mmol, 0.5 equiv) and 4-ClCzIPN (**4b**, 4 mg, 4 μmol, 2 mol%), CH_2Cl_2 (0.8 mL, 0.25 M) after 18 hours.
Column: Pentane:EtOAc, 1:0 to 9:1. Yield 63% (0.074 g, 0.12 mmol). Pale yellow oil.

[29] Some reactions were performed on 0.2 mmol scale of PhEBX with 1.2 equiv of alkene.
[30] Coelution of residual alkene was often observed hence the greater quantities of SiO_2. The corresponding dilution can lead to poor detection of the compound by TLC (both UV and p-anisaldehyde stains should be combined).

Rf (Pent.:EtOAc 9:1) = 0.25
^{1}H NMR (400 MHz, Acetonitrile-d_3, 7:3 mixture of rotamers) δ 8.06 (d, *J* = 7.9 Hz, 1H, Ar*H*), 7.85 (dd, *J* = 7.8, 1.7 Hz, 1H, Ar*H*), 7.56 – 7.37 (m, 6H, Ph*H* + Ar*H*), 7.33 – 7.19 (m, 6H, Ph*H* + Ar*H*), 5.64 (bs, 0.7H, major, NC*H*), 5.37 (bs, 0.3H, minor, NC*H*), 4.55 (d, *J* = 6.9 Hz, 2H, OC*H*$_2$), 3.66 – 3.43 (m, 2H, C*H*$_2$), 3.01 (dq, *J* = 12.5, 7.5, 6.9 Hz, 2H, C*H*$_2$), 1.46 (s, 9H, C(C*H*$_3$)$_3$). **^{13}C NMR** (101 MHz, Acetonitrile-d_3, mixture of rotamers, signals not fully resolved) δ 172.0, 166.2, 142.0, 135.3, 133.7, 132.2, 131.6, 129.6, 129.2, 128.8, 122.5, 94.0, 81.1, 65.0, 60.8, 47.6, 40.9, 35.1, 28.0, 14.1. **IR** ($ν_{max}$, cm^{-1}) 2977 (s), 2952 (m), 1733 (s), 1692 (s), 1403 (s), 1366 (s), 1247 (s), 1159 (s). **HRMS** (ESI/QTOF) m/z: [M + Na]$^+$ Calcd for $C_{30}H_{30}INNaO_4^+$ 618.1112; Found 618.1109.

2-(((Benzyloxy)carbonyl)(phenethyl)amino)-4-phenylbut-3-yn-1-yl 2-iodobenzoate (**3c**)

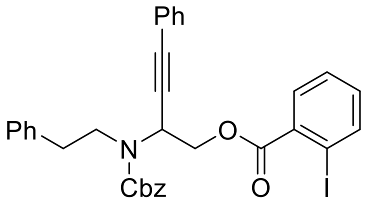

Performed on 0.2 mmol scale.
Obtained from benzyl phenethyl(vinyl)carbamate (**1c**, 0.068 g, 0.24 mmol, 1.2 equiv); PhEBX (**2**, 0.070 g, 0.2 mmol, 1.0 equiv); BIOAc (**7**, 0.031 g, 0.1 mmol, 0.5 equiv) and 4-ClCzIPN (**4b**, 4 mg, 4 μmol, 2 mol%) after 18 hours.
Column: Pentane:EtOAc, 10:0 to 9:1. Yield 86% (0.108 g, 0.172 mmol). Pale yellow oil.
Rf (Pent.:EtOAc 9:1) = 0.25.
^{1}H NMR (400 MHz, Acetonitrile-d_3, 6:4 mixture of rotamers) δ 8.01 (dd, *J* = 8.0, 1.2 Hz, 1H, Ar*H*), 7.75 (d, *J* = 7.7 Hz, 1H, Ar*H*), 7.61 – 7.07 (m, 12H, Ph*H*), 5.62 (bs, 0.6H, major, NC*H*), 5.48 (bs, 0.4H minor, NC*H*)., 5.12 (d, *J* = 3.7 Hz, 2H, OC*H*$_2$Ph), 4.58 – 4.51 (m, 2H, CHC*H*$_2$O), 3.72 – 3.47 (m, 2H, CH_2CH_2), 3.12 – 2.90 (m, 2H, CH_2CH_2). **^{13}C NMR** (101 MHz, Acetonitrile-d_3, mixture of rotamers, signals not fully resolved) δ 166.7, 156.8, 142.2, 140.2, 137.7, 134.0, 132.6, 131.8, 130.0, 129.7, 129.6, 129.4, 129.3, 128.9, 122.8, 94.3, 86.8, 84.5, 68.2, 65.5, 49.0, 47.4, 36.9, 35.9. **IR** ($ν_{max}$, cm^{-1}) 3033 (m), 3071 (m), 2951 (m), 2862 (m), 1732 (s), 1700 (s), 1586 (m), 1491 (m), 1453 (s), 1409 (s), 1370 (m), 1282 (s), 1250 (s), 1174 (m), 1129 (s), 1104 (s), 1047 (m), 1015 (s), 977 (m), 742 (s), 685 (m), 691 (s). **HRMS** (ESI/QTOF) m/z: [M + Na]$^+$ Calcd for $C_{33}H_{28}INNaO_4^+$ 652.0955; Found 652.0961.

2-(Benzyl(*tert*-butoxycarbonyl)amino)-4-phenylbut-3-yn-1-yl 2-iodobenzoate (**3d**)

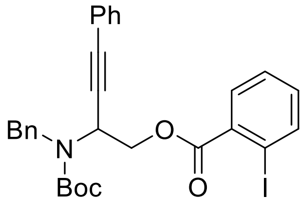

Performed on 0.2 mmol scale.
Obtained from *tert*-butyl benzyl(vinyl)carbamate (**1d**, 0.056 g, 0.24 mmol, 1.2 equiv); PhEBX (**2**, 0.070 g, 0.2 mmol, 1.0 equiv); BIOAc (**7**, 0.031 g, 0.1 mmol, 0.5 equiv) and 4-ClCzIPN (**4b**, 4 mg, 4 μmol, 2 mol%) after 18 hours.
Column: Pentane:EtOAc, 10:0 to 9:1. Yield 73% (0.085 g, 0.146 mmol). Pale yellow oil.
Rf (Pent.:EtOAc 9:1) = 0.25.
^{1}H NMR (400 MHz, Acetonitrile-d_3, 6:4 mixture of rotamers) δ 8.06 (dd, *J* = 8.0, 1.2 Hz, 1H, Ar*H*), 7.83 (dd, *J* = 7.8, 1.7 Hz, 1H, Ar*H*), 7.49 (t, *J* = 7.6 Hz, 1H, Ar*H*), 7.45 – 7.21 (m, 11H, Ar*H* + Ph*H*), 5.78 (m, 0.6H, major, NC*H*), 5.45 (m, 0.4H, minor, NC*H*), 4.71 (d, *J* = 16.3 Hz, 1H, PhC*H*$_2$), 4.60 (d, *J* = 16.3 Hz, 1H, PhC*H*$_2$), 4.52 (dd, *J* = 11.2, 5.5 Hz, 1H, OC*H*$_2$), 4.46 (dd, *J* = 11.2, 8.4 Hz, 1H, OC*H*$_2$), 1.62 – 1.16 (m, 9H, C(C*H*$_3$)$_3$). **^{13}C NMR** (101 MHz, Acetonitrile-d_3, mixture of rotamers, signals not fully resolved) δ 166.1, 155.7, 141.9, 140.4, 135.1, 133.7, 132.1, 131.6, 129.4, 129.1, 128.9, 128.8, 128.7, 127.5, 127.3, 122.5, 94.1, 86.6, 84.4, 81.0, 65.2, 48.2, 48.1, 28.0. **IR** ($ν_{max}$, cm^{-1}) 3062 (w), 3029 (w), 2976 (m), 2930 (w),

1733 (m), 1704 (s), 1685 (s), 1392 (s), 1366 (m), 1287 (m), 1242 (s), 1162 (s), 1131 (m), 1119 (s), 1101 (s), 1016 (m). **HRMS** (ESI/QTOF) m/z: [M + Na]$^+$ Calcd for $C_{29}H_{28}INNaO_4^+$ 604.0955; Found 604.0957.

2-(Methyl(*tert*-butoxycarbonyl)amino)-4-phenylbut-3-yn-1-yl 2-iodobenzoate (**3e**)

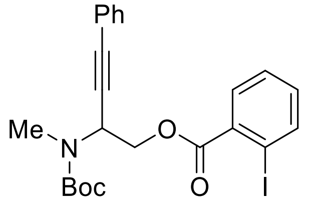

Obtained from *tert*-butyl methyl(vinyl)carbamate (**1e**, 0.059 g, 0.38 mmol, 1.5 equiv); PhEBX (**2**, 0.087 g, 0.2 mmol, 1.0 equiv); BIOAc (**7**, 0.038 g, 0.13 mmol, 0.5 equiv) and 4-ClCzIPN (**4b**, 5 mg, 5 µmol, 2 mol%) after 18 hours.
Column: Pentane:EtOAc, 10:0 to 9:1. Yield 83% (0.105 g, 0.208 mmol). Colorless oil.

Rf (Pent.:EtOAc 9:1) = 0.25.

^{1}H NMR (400 MHz, Acetonitrile-*d*$_3$, 7 : 3 mixture of rotamers) δ 8.06 (d, *J* = 7.9 Hz, 1H, Ar*H*), 7.83 (d, *J* = 8.1 Hz, 1H, Ar*H*), 7.54 – 7.42 (m, 3H, Ar*H* + Ph*H*), 7.46 – 7.35 (m, 3H, Ph*H*), 7.26 (td, *J* = 7.7, 1.7 Hz, 1H, Ar*H*), 5.70 (bs, 0.7H, major, NC*H*), 5.54 (bs, 0.3H, minor, NC*H*), 4.64 – 4.44 (m, 2H, OC*H*$_2$), 2.98 (s, 3H, NC*H*$_3$), 1.40 (bs, *J* = 9.8 Hz, 9H, C(C*H*$_3$)$_3$). **^{13}C NMR** (101 MHz, Acetonitrile-*d*$_3$, mixture of rotamers, signals not fully resolved) δ 166.6, 156.3, 155.3, 142.3, 135.7, 134.1, 132.6, 132.0, 129.9, 129.6, 129.2, 122.9, 94.5, 94.4, 86.7, 84.1, 80.9, 64.4, 49.1, 47.9, 30.2, 28.4. **IR** (ν_{max}, cm^{-1}) 3059 (w), 2976 (m), 2932 (w), 2875 (w), 1733 (m), 1685 (s), 1388 (s), 1245 (s), 1147 (s), 741 (s). **HRMS** (ESI/QTOF) m/z: [M + Na]$^+$ Calcd for $C_{23}H_{24}INNaO_4^+$ 528.0642; Found 528.0651.

2-(Allyl(*tert*-butoxycarbonyl)amino)-4-phenylbut-3-yn-1-yl 2-iodobenzoate (**3f**)

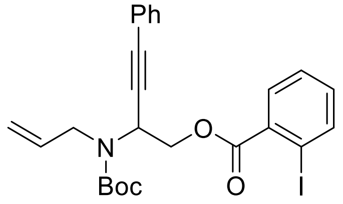

Obtained from *tert*-butyl allyyl(vinyl)carbamate (**1f**, 0.069 g, 0.38 mmol, 1.5 equiv); PhEBX (**2**, 0.087 g, 0.25 mmol, 1.0 equiv); BIOAc (**7**, 0.038 g, 0.13 mmol, 0.5 equiv) and 4-ClCzIPN (**4b**, 5 mg, 5 µmol, 2 mol%) after 18 hours.
Column: Pentane:EtOAc, 10:0 to 9:1. Yield 54% (0.072 g, 0.14 mmol). Pale yellow oil.

Rf (Pent.:Et_2O 9:1) = 0.2.

^{1}H NMR (400 MHz, Acetonitrile-*d*$_3$, 6:4 mixture of rotamers) δ 8.04 (dd, *J* = 8.0, 1.2 Hz, 1H, Ar*H*), 7.83 (dd, *J* = 7.8, 1.7 Hz, 1H, Ar*H*), 7.52 – 7.43 (m, 3H, Ar*H* + Ph*H*), 7.43 – 7.33 (m, 3H, Ph*H*), 7.24 (td, *J* = 7.7, 1.7 Hz, 1H, Ar*H*), 6.08 – 5.90 (m, 1H, R-C*H*=CH$_2$), 5.63 (bs, 0.6H, NC*H*), 5.33 (bs, 0.4H, NC*H*), 5.28 – 5.19 (m, 1H, CH=C*H*$_2$), 5.19 – 5.06 (m, 1H, CH=C*H*$_2$), 4.49 (m, 2H, C*H*$_2$), 4.09 – 3.89 (m, 2H, C*H*$_2$), 1.39 (s, 9H, C(C*H*$_3$)$_3$). **^{13}C NMR** (101 MHz, Acetonitrile-d3) δ 166.2, 155.6, 141.9, 136.3, 136.0, 135.3, 134.2, 133.7, 132.1, 131.6, 130.4, 129.5, 129.2, 128.8, 128.4, 122.6, 116.2, 114.8, 94.0, 80.8, 65.0, 28.2, 28.0. **IR** (ν_{max}, cm^{-1}) 3377 (w), 3073 (w), 2977 (w), 2933 (w), 1733 (m), 1691 (s), 1583 (w), 1449 (m), 1395 (s), 1368 (m), 1322 (m), 1284 (m), 1247 (s), 1168 (s), 1139 (s), 1103 (m), 1043 (w), 1014 (m), 989 (m), 920 (w), 862 (w), 743 (s), 692 (m). **HRMS** (ESI/QTOF) m/z: [M + Na]$^+$ Calcd for $C_{25}H_{26}INNaO_4^+$ 554.0799; Found 554.0809.

2-((*Tert*-butoxycarbonyl)(2-((*tert*-butyldimethylsilyl)oxy)ethyl)amino)-4-phenylbut-3-yn-1-yl 2-iodobenzoate (**3g**)

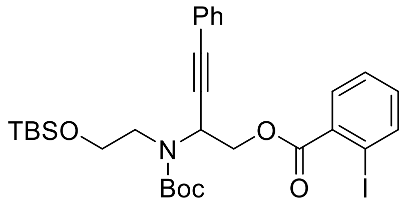

Obtained from *tert*-butyl (2-((*tert*-butyldimethylsilyl)oxy)ethyl)(vinyl)carbamate (**1g**, 0.072 g, 0.24 mmol, 1.2 equiv); PhEBX (**2**, 0.070 g, 0.2 mmol, 1.0 equiv); BIOAc (**7**, 0.031 g, 0.1 mmol, 0.5 equiv) and 4-ClCzIPN (**4b**, 4 mg, 4 µmol, 2 mol%) after 18 hours.
Column: Pentane:EtOAc, 10:0 to 9:1. Yield 52% (0.068 g, 0.11 mmol). Pale yellow oil.

Rf (Pent.:EtOAc 9:1) = 0.4.

^{1}H NMR (400 MHz, Acetonitrile-d_3, 7:3 mixture of rotamers) δ 8.05 – 7.98 (m, 1H, Ar*H*), 7.80 (dd, *J* = 7.8, 1.7 Hz, 1H, Ar*H*), 7.48 – 7.38 (m, 3H, Ar*H* + Ph*H*), 7.41 – 7.29 (m, 3H, Ph*H*), 7.21 (td, *J* = 7.7, 1.8 Hz, 1H, , Ar*H*), 5.53 (bs, 0.6H, major, NC*H*), 5.24 (s, 0.4H, minor, NC*H*), 4.65 – 4.35 (m, 2H, C*H*$_2$), 3.82 – 3.74 (m, 2H, C*H*$_2$), 3.44 (d, *J* = 6.9 Hz, 2H, C*H*$_2$), 1.38 (s, 9H, C(C*H*$_3$)$_3$), 0.83 (s, 9H, C(C*H*$_3$)$_3$), 0.00 (s, 6H, Me$_2$). **^{13}C NMR** (101 MHz, Acetonitrile-d_3, mixture of rotamers, signals not fully resolved) δ 166.7, 142.3, 135.9, 134.1, 132.6, 132.0, 129.9, 129.6, 129.2, 94.4, 81.3, 65.7, 62.5, 48.7, 48.3, 47.5, 28.5, 26.3, 26.3, 18.9, -5.1. **IR** (ν_{max}, cm^{-1}) 2986 (s), 2900 (s), 1735 (s), 1698 (s), 1405 (s), 1250 (s), 1050 (s). **HRMS** (ESI/QTOF) m/z: [M + H]$^+$ Calcd for $C_{30}H_{41}INO_5Si^+$ 650.1793; Found 650.1791.

2-((*Tert*-butoxycarbonyl)(3-ethoxy-3-oxopropyl)amino)-4-phenylbut-3-yn-1-yl 2-iodobenzoate (**3h**)

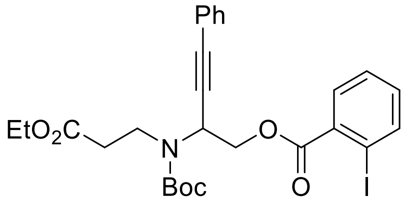

Obtained from ethyl 3-((*tert*-butoxycarbonyl)(vinyl)amino)propanoate (**1h**, 0.058 g, 0.24 mmol, 1.2 equiv); PhEBX (**2**, 0.070 g, 0.2 mmol, 1.0 equiv); BIOAc (**7**, 0.031 g, 0.1 mmol, 0.5 equiv) and 4-ClCzIPN (**4b**, 4 mg, 4 µmol, 2 mol%) after 18 hours.

Column: Pentane:EtOAc, 10:0 to 9:1. Yield 69% (0.082 g, 0.14 mmol). Pale yellow oil.

Rf (Pent.:EtOAc 9:1) = 0.35.

^{1}H NMR (400 MHz, Acetonitrile-d_3, 7:3 mixture of rotamers) δ 8.05 – 7.98 (m, 1H, Ar*H*), 7.80 (d, *J* = 7.8 Hz, 1H, Ar*H*), 7.48 – 7.41 (m, 3H, Ar*H* and Ph*H*), 7.41 – 7.30 (m, 3H, Ar*H* and Ph*H*), 7.22 (td, *J* = 7.7, 1.7 Hz, 1H, Ar*H*), 5.53 (bs, 0.7H, major, NC*H*), 5.31 (bs, 0.3H, minor, NC*H*), 4.50 (d, *J* = 7.0 Hz, 2H, OC*H*$_2$-CHN), 4.02 (q, *J* = 7.1 Hz, 2H, OC*H*$_2$CH$_3$), 3.60 (m, 2H, C*H*$_2$), 2.68 (d, *J* = 8.7 Hz, 2H, C*H*$_2$), 1.37 (s, 9H, C(C*H*$_3$)$_3$), 1.13 (t, *J* = 7.1 Hz, 3H, OCH$_2$C*H*$_3$). **^{13}C NMR** (101 MHz, Acetonitrile-d_3, mixture of rotamers, signals not fully resolved) δ 172.0, 166.2, 142.0, 135.3, 133.7, 132.2, 131.6, 129.6, 129.2, 128.8, 122.5, 94.0, 81.1, 65.0, 60.8, 47.6, 35.1, 28.0, 14.1. **IR** (ν_{max}, cm^{-1}) 3009 (s), 3004 (s), 2974 (s), 2943 (s), 2928 (s), 2883 (s), 1739 (s), 1726 (s), 1716 (s), 1705 (s), 1696 (s), 1685 (s), 1678 (s), 1406 (s), 1248 (s), 1159 (s), 1046 (s). **HRMS** (ESI/QTOF) m/z: [M + Na]$^+$ Calcd for $C_{27}H_{30}INNaO_6^+$ 614.1010; Found 614.1011.

2-((*Tert*-butoxycarbonyl)(cyclohexyl)amino)-4-phenylbut-3-yn-1-yl 2-iodobenzoate (**3i**)

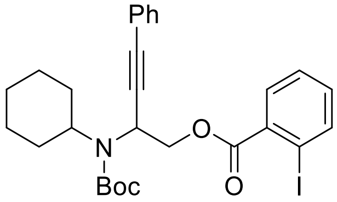

Obtained from cyclohexyl(vinyl)carbamate (**1i**, 0.068 g, 0.24 mmol, 1.2 equiv); PhEBX (**2**, 0.070 g, 0.2 mmol, 1.0 equiv); BIOAc (**7**, 0.031 g, 0.1 mmol, 0.5 equiv) and 4-ClCzIPN (**4b**, 4 mg, 4 µmol, 2 mol%) after 18 hours.

Column: Pentane:EtOAc, 10:0 to 9:1. Yield 65% (0.074 g, 0.13 mmol). Pale yellow oil.

Rf (Pent.:EtOAc 9:1) = 0.3.

^{1}H NMR (400 MHz, Acetonitrile-d_3, 1:1 mixture of rotamers) δ 8.04 (dd, *J* = 7.9, 1.2 Hz, 1H, Ar*H*), 7.82 (dd, *J* = 7.8, 1.7 Hz, 1H, Ar*H*), 7.50 – 7.41 (m, 3H, Ar*H* and Ph*H*), 7.41 – 7.32 (m, 3H, Ar*H* and Ph*H*), 7.24 (td, *J* = 7.7, 1.7 Hz, 1H, Ar*H*), 5.49 (bs, 0.5H, N-*CH*-alkyne), 5.01 (bs, 0.5H, N*CH*-alkyne), 4.59 (bs, 1H, O*CH*$_2$), 4.50 (bs, 1H, O*CH*$_2$) 3.70 (bs, 0.5H, Cy*H*-N), 3.44 (bs, 0.5H, Cy*H*-N), 1.88 – 1.69 (m, 4H, *Cy*), 1.70 – 1.53 (m, 2H, *Cy*), 1.44 (s, 9H, C(CH$_3$)$_3$), 1.37 – 1.22 (m, 2H, *Cy*), 1.12 (ddt, *J* = 16.3, 12.7, 3.4 Hz, 2H, *Cy*). **^{13}C NMR** (101 MHz, Acetonitrile-d_3, mixture of rotamers, signals not fully resolved) δ 166.4, 141.9, 135.5, 133.7, 132.0, 131.5, 129.3, 129.2, 128.8, 122.9, 94.0, 80.6, 65.7, 65.4, 56.6, 33.6, 31.5, 28.2, 26.6, 25.9, 25.4. **IR** (ν_{max}, cm^{-1}) 2987 (s), 2972 (s), 2960 (s), 2901 (s), 1733 (s), 1705 (s), 1698 (s), 1686 (s). **HRMS** (ESI/QTOF) m/z: [M + Na]$^+$ Calcd for $C_{28}H_{32}INNaO_4^+$ 596.1268; Found 596.1270.

2-(2-oxopyrrolidin-1-yl)-4-phenylbut-3-yn-1-yl 2-iodobenzoate (**3j**)

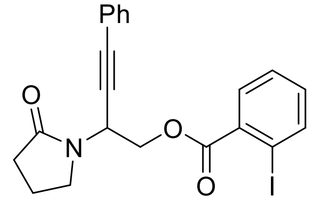

Obtained from *N*-vinyl pyrrolidinone (**1j**, 0.042 g, 0.38 mmol, 1.5 equiv); PhEBX (**2**, 0.087 g, 0.25 mmol, 1.0 equiv); BIOAc (**7**, 0.031 g, 0.1 mmol, 0.5 equiv) and 4-ClCzIPN (**4b**, 4 mg, 4 μmol, 2 mol%) after 18 hours.

Column: Pentane:EtOAc, 9:1 to 8:2. Yield 85% (0.098 g, 0.21 mmol). Pale yellow oil.

Rf (Pent.:EtOAc 9:1) = 0.25.

^{1}H NMR (400 MHz, Acetonitrile-d_3) δ 8.06 (dd, *J* = 7.9, 1.2 Hz, 1H, Ar*H*), 7.81 (dd, *J* = 7.8, 1.7 Hz, 1H, Ar*H*), 7.58 – 7.47 (m, 3H, Ar*H* and Ph*H*), 7.47 – 7.33 (m, 3H, Ph*H*), 7.27 (td, *J* = 7.7, 1.8 Hz, 1H, Ar*H*), 5.53 (dd, *J* = 8.6, 4.8 Hz, 1H, NC*H*CH_2O), 4.64 (dd, *J* = 11.3, 8.6 Hz, 1H, NCHCH_2O), 4.50 (dd, *J* = 11.2, 4.8 Hz, 1H, NCHCH_2O), 3.75 – 3.52 (m, 2H, CH_2), 2.39 – 2.30 (m, 2H, CH_2), 2.12 – 2.02 (m, 2H, CH_2). **^{13}C NMR** (101 MHz, Acetonitrile-d_3) δ 175.4, 166.5, 141.8, 135.6, 133.7, 132.3, 131.4, 129.6, 129.2, 129.1, 128.9, 122.4, 93.8, 86.0, 83.1, 64.4, 43.9, 31.1, 18.2. **IR** (ν_{max}, cm^{-1}) 3054 (s), 2972 (s), 2894 (s), 1732 (s), 1686 (s), 1417 (s), 1284 (s), 1246 (s), 1132 (s), 1104 (s). **HRMS** (ESI/QTOF) m/z: [M + H]$^+$ Calcd for $C_{21}H_{19}INO_3^+$ 460.0404; Found 460.0414

3-(2-Oxooxazolidin-3-yl)-1-phenyloct-1-yn-4-yl 2-iodobenzoate (**3l**)

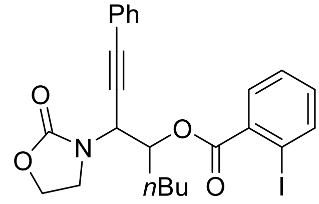

Obtained from (*E*)-3-(Hex-1-en-1-yl)oxazolidin-2-one ((*E*)-**1l**, 0.064 g, 0.38 mmol, 1.5 equiv); PhEBX (**2**, 0.087 g, 0.25 mmol, 1.0 equiv); BIOAc (**7**, 0.038 g, 0.13 mmol, 0.5 equiv) and 4-ClCzIPN (**4b**, 5 mg, 5 μmol, 2 mol%) after 18 hours.

Column: Pentane:EtOAc, 19:1 to 8:2. Isolated separately as two diastereoisomers (0.076 g, 60% and 0.027 g, 20% yield) as 2 colorless oils. Overall yield 80% (0.103 g, 0.199 mmol, dr: 3:1).

Obtained from 2:1 mixture of (*Z*)-3-(Hex-1-en-1-yl)oxazolidin-2-one and (*E*)-isomer ((*Z*:*E*)-**1k**, 0.064 g, 0.38 mmol, 1.5 equiv); PhEBX (**2**, 0.087 g, 0.25 mmol, 1.0 equiv);
BIOAc (**7**, 0.038 g, 0.13 mmol, 0.5 equiv) and 4-ClCzIPN (**4b**, 5 mg, 5 μmol, 2 mol%) after 18 hours.
Column: Pentane:EtOAc, 19:1 to 8:2. Isolated seperately as two diastereoisomers (0.079 g, 61% yield and 0.025 g, 19% yield) as 2 colorless oils.. Overall yield 80% (0.104g, 0.201 mmol, dr: 3:1).

Major diastereoisomer:

Rf (Pent:EtOAc 7:3) = 0.4.

^{1}H NMR (400 MHz, Chloroform-*d*) δ 7.99 (dd, *J* = 8.0, 1.2 Hz, 1H, Ar*H*), 7.78 (dd, *J* = 7.8, 1.7 Hz, 1H, Ar*H*), 7.45 - 7.42 (m, 2H, Ph*H*), 7.40 (dd, *J* = 7.6, 1.3 Hz, 1H, Ar*H*), 7.38 - 7.30 (m, 3H, Ph*H*), 7.16 (td, *J* = 7.7, 1.7 Hz, 1H, Ar*H*), 5.43 (td, *J* = 8.7, 3.4 Hz, 1H, OC*H*), 5.11 (d, *J* = 8.5 Hz, 1H, NC*H*), 4.37 (td, *J* = 8.1, 6.0 Hz, 1H, CH_2), 4.30 (q, *J* = 8.8 Hz, 1H, CH_2), 3.86 - 3.76 (m, 2H, CH_2), 2.02 (m, 1H, CH_2), 1.86 (dtd, *J* = 14.4, 8.8, 5.8 Hz, 1H, CH_2), 1.59 - 1.27 (m, 4H CH_2- CH_2- CH_3), 0.92 (t, *J* = 7.2 Hz, 3H, CH_3). **^{13}C NMR** (101 MHz, Chloroform-*d*) δ 166.3, 158.3, 141.3, 134.8, 132.9, 131.9, 130.9, 129.1, 128.5, 128.2, 121.6, 94.1, 87.8, 81.6, 73.8, 62.5, 50.5, 41.6, 31.1, 27.2, 22.4, 13.9. **IR** (ν_{max}, cm^{-1}) 2960 (w), 2929 (w), 2862 (w), 2252 (w), 1742 (m), 1736 (m), 1420 (m), 1286 (m), 1249 (m), 907 (s), 727 (s). **HRMS** (ESI/QTOF) m/z: [M + Na]$^+$ Calcd for $C_{24}H_{24}INNaO_4^+$ 540.0642; Found 540.0640.

Minor diastereoisomer:

Rf (Pent:EtOAc 7:3) = 0.35.

^{1}H NMR (400 MHz, Chloroform-*d*) δ 7.99 (dd, *J* = 8.0, 1.2 Hz, 1H, Ar*H*), 7.78 (dd, *J* = 7.8, 1.7 Hz, 1H, Ar*H*), 7.50 – 7.30 (m, 6H, Ph*H* + Ar*H*), 7.16 (td, *J* = 7.7, 1.7 Hz, 1H, Ar*H*), 5.43 (td, *J* = 8.7, 3.4 Hz, 1H OC*H*), 5.11 (d, *J* = 8.5 Hz, 1H, NC*H*), 4.52 – 4.24 (m, 2H, CH_2), 3.91 – 3.77 (m, 2H, CH_2), 2.05 (m, 1H, CH_2), 1.86 (m, 5.7 Hz, 1H, CH_2), 1.56 – 1.21 (m, 4H, CH_2-CH_2-CH_3), 0.90 (dt, *J* = 18.0, 7.2 Hz, 3H, CH_2-CH_2-CH_3). **^{13}C NMR** (101 MHz, Chloroform-*d*) δ 165.7, 158.2, 141.7, 134.7, 133.1, 132.0, 131.3, 129.1, 128.5, 128.2, 122.0, 94.5, 87.7, 81.7, 75.8, 62.6, 50.2, 42.7, 31.3, 27.5, 22.6, 14.1. **IR** (ν_{max}, cm^{-1}) 2957 (m),

2925 (m), 2871 (m), 1743 (s), 1727 (s), 1490 (m), 1417 (m), 1246 (s), 1133 (s), 759 (s), 741 (s). **HRMS** (ESI/QTOF) m/z: [M + H]$^+$ Calcd for $C_{24}H_{25}INO_4^+$ 518.0823; Found 518.0830.

2-Butoxy-4-phenylbut-3-yn-1-yl 2-iodobenzoate (**10a**)

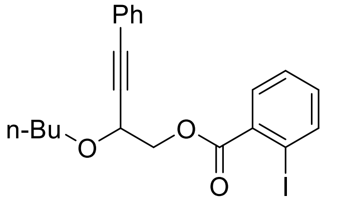

Obtained from *n*butyl vinyl ether (**9a**, 0.048 mL, 0.38 mmol, 1.5 equiv); PhEBX (**2**, 0.087 g, 0.25 mmol, 1.0 equiv); BIOAc (**7**, 0.038 g, 0.1 mmol, 0.5 equiv) and 4-ClCzIPN (**4b**, 5 mg, 5 μmol, 2 mol%) after 18 hours.
Column: Pentane:Et_2O, 100:0 to 95:5. Yield 89% (0.100 g, 0.223 mmol). Colorless oil.

Rf (Pentane:Et_2O 95:5) = 0.4.

^{1}H NMR (400 MHz, Chloroform-*d*) δ 8.00 (dd, *J* = 7.9, 1.3 Hz, 1H, Ar*H*), 7.88 (dd, *J* = 7.8, 1.7 Hz, 1H, Ar*H*), 7.48 – 7.41 (m, 2H, Ph*H*), 7.39 (t, *J* = 7.6 Hz, 1H, Ar*H*), 7.36 – 7.27 (m, 3H, Ph*H*), 7.15 (td, *J* = 7.7, 1.8 Hz, 1H, Ar*H*), 4.68 (dd, *J* = 6.8, 4.8 Hz, 1H, O*CH*CH$_2$), 4.57 (d, *J* = 5.2 Hz, 2H, CH_2), 3.86 (dt, *J* = 9.1, 6.6 Hz, 1H, CH_2), 3.55 (dt, *J* = 9.1, 6.5 Hz, 1H, CH_2), 1.63 (dq, *J* = 8.3, 6.6 Hz, 2H, CH_2), 1.49 – 1.35 (m, 2H, CH_2), 0.92 (t, *J* = 7.4 Hz, 3H, CH_3). **^{13}C NMR** (101 MHz, Chloroform-*d*) δ 166.2, 141.5, 134.8, 132.9, 132.0, 131.4, 128.8, 128.4, 128.0, 122.4, 94.4, 87.1, 84.9, 69.2, 68.3, 66.8, 31.8, 19.4, 14.0. **IR** (ν_{max}, cm^{-1}) 2987 (s), 2978 (s), 2934 (s), 2912 (s), 2901 (s), 2855 (s), 1756 (s), 1465 (s), 1428 (s), 1378 (s), 1269 (s), 1103 (s), 1076 (s), 1057 (s), 1027 (s). **HRMS** (ESI/QTOF) m/z: [M + Na]$^+$ Calcd for $C_{21}H_{21}INaO_3^+$ 471.0428; Found 471.0438.

2-Benzyloxy-4-phenylbut-1-yn-4-yl 2-iodobenzoate (**10b**)

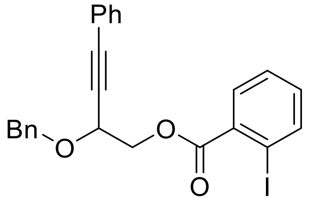

Obtained from ((vinyloxy)methyl)benzene (**9b**, 50 μl, 0.38 mmol, 1.5 equiv); PhEBX (**2**, 0.087 g, 0.2 mmol, 1.0 equiv); BIOAc (**7**, 0.038 g, 0.1 mmol, 0.5 equiv) and 4-ClCzIPN (**4b**, 4 mg, 4 μmol, 2 mol%) after 18 hours.
Column: Pentane:EtOAc, 9:1 to 8:2. Yield 58% (70 mg, 0.15 mmol). Yellow oil.

Rf (Pent:EtOAc = 9:1) = 0.4.

^{1}H NMR (400 MHz, Acetonitrile-d_3) δ 8.03 (dd, *J* = 8.0, 1.2 Hz, 1H, Ar*H*), 7.79 (dd, *J* = 7.8, 1.7 Hz, 1H, Ar*H*), 7.58 – 7.44 (m, 3H, Ph*H* + Ar*H*), 7.45 – 7.26 (m, 8H, Ph*H*), 7.24 (td, *J* = 7.7, 1.7 Hz, 1H, Ar*H*), 4.87 (d, *J* = 11.7 Hz, 1H, PhCH_2), 4.77 (dd, *J* = 6.6, 4.2 Hz, 1H, O*CH*), 4.66 (d, *J* = 11.7 Hz, 1H, PhCH_2), 4.64 – 4.50 (m, 2H, OCH_2). **^{13}C NMR** (101 MHz, Acetonitrile-d_3) δ 166.6, 141.8, 138.4, 135.9, 133.6, 132.3, 131.3, 129.6, 129.2, 129.0, 128.9, 128.7, 128.4, 122.5, 93.8, 87.5, 85.1, 71.2, 68.1, 66.8. **IR** (ν_{max}, cm^{-1}) 2987 (s), 2972 (s), 2959 (s), 2920 (s), 2909 (s), 2901 (s), 2884 (s), 1726 (s), 1394 (s), 1375 (s), 1286 (s), 1265 (s), 1243 (s), 1135 (s), 1076 (s), 1038 (s), 1016 (s). **HRMS** (APPI/LTQ-Orbitrap) m/z: [M + H]$^+$ Calcd for $C_{24}H_{20}IO_3^+$ 483.0452; Found 483.0435.

2-(Allyloxy)-4-phenylbut-3-yn-1-yl 2-iodobenzoate (**10c**)

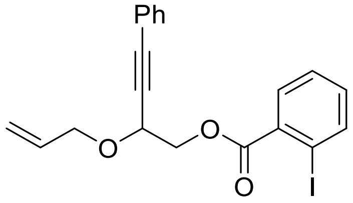

Obtained from (allyloxy)ethene (**9c**, 0.039 mL, 0.38 mmol, 1.5 equiv); PhEBX (**2**, 0.087 g, 0.25 mmol, 1.0 equiv); BIOAc (**7**, 0.038 g, 0.13 mmol, 0.5 equiv) and 4-ClCzIPN (**4b**, 5 mg, 5 μmol, 2 mol%) after 18 hours.

Column: Pentane:Et_2O, 95:5 to 90:10. Some fractions were not pure and were purified by preparative TLC (Pentane:Et_2O 93:7). Combined yield 46% (0.050 g, 0.12 mmol). Colorless oil.

Rf (Pent:Et_2O = 9:1) = 0.5.

^{1}H NMR (400 MHz, Acetonitrile-d3) δ 8.03 (dd, *J* = 7.9, 1.1 Hz, 1H, Ar*H*), 7.82 (d, *J* = 1.7 Hz, 1H, Ar*H*), 7.53 – 7.43 (m, 3H, Ph*H* + Ar*H*), 7.42 – 7.32 (m, 3H, Ph*H*), 7.25 (ddd, *J* = 8.0, 7.5, 1.7 Hz, 1H, Ar*H*), 5.97 (dddd, *J* = 17.2, 10.4, 6.0, 5.2 Hz, 1H, C*H*=CH$_2$), 5.35 (dq, *J* = 17.3, 1.7 Hz, 1H, CH=C*H*$_2$), 5.22 – 5.18 (m, 1H, CH=C*H*$_2$), 4.75 (dd, *J* = 6.3, 4.4 Hz, 1H, OC*H*), 4.62 – 4.47 (m, 2H, OC*H*$_2$), 4.33 (ddt, *J* = 12.7, 5.2, 1.5 Hz, 1H, OC*H*$_2$), 4.13 (ddt, *J* = 12.7, 6.0, 1.4 Hz, 1H, OC*H*$_2$). **^{13}C NMR** (101 MHz, Acetonitrile-*d$_3$*) δ 166.7, 141.7, 136.0, 135.0, 133.6, 132.2, 131.2, 129.5, 129.2, 128.9, 122.5, 117.4, 93.7, 87.2, 85.2, 70.2, 68.0, 66.8. **IR** (ν_{max}, cm^{-1}) 3078 (m), 2987 (s), 2972 (s), 2900 (s), 1732 (s), 1286 (s), 1243 (s), 1134 (s), 1097 (s), 1079 (s), 1045 (s), 1016 (s). **HRMS** (APPI/LTQ-Orbitrap) m/z: [M]$^+$ Calcd for $C_{20}H_{17}IO_3^+$ 432.0217; Found 432.0210.

2-(2-Chloroethoxy)-4-phenylbut-3-yn-1-yl 2-iodobenzoate (**10d**)

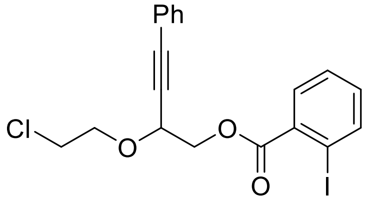

Obtained from (2-chloroethoxy)ethene (**9d**, 0.038 mL, 0.38 mmol, 1.5 equiv); PhEBX (**2**, 0.087 g, 0.25 mmol, 1.0 equiv); BIOAc (**7**, 0.038 g, 0.13 mmol, 0.5 equiv) and 4-ClCzIPN (**4b**, 5 mg, 5 µmol, 2 mol%) after 18 hours.

Column: Pentane:Et_2O, 95:5 to 90:10. Some fractions were not pure and were purified by preparative TLC (Pentane:Et_2O 93:7). Combined yield 77% (0.088 g, 0.19 mmol, > 90% pure). Pale yellow oil with residual grease.

Rf (Pentane:EtOac 9:1) = 0.7.

^{1}H NMR (400 MHz, Chloroform-*d*) δ 8.01 (dd, *J* = 8.0, 1.2 Hz, 1H, Ar*H*), 7.90 (dd, *J* = 7.8, 1.7 Hz, 1H, Ar*H*), 7.48 – 7.43 (m, 2H, Ph*H*), 7.43 – 7.37 (m, 1H, Ar*H*), 7.36 – 7.30 (m, 3H, Ph*H*), 7.17 (td, *J* = 7.7, 1.7 Hz, 1H, Ar*H*), 4.79 (dd, *J* = 6.2, 5.3 Hz, 1H, OC*H*), 4.69 – 4.53 (m, 2H, OC*H*$_2$), 4.11 (dt, *J* = 10.5, 5.7 Hz, 1H, OC*H*$_2$), 3.85 (ddd, *J* = 10.5, 6.5, 5.6 Hz, 1H, OC*H*$_2$), 3.78 – 3.66 (m, 2H, C*H*$_2$Cl). **^{13}C NMR** (101 MHz, Chloroform-*d*) δ 166.0, 141.4, 134.5, 132.9, 131.9, 131.4, 128.9, 128.4, 128.0, 121.9, 94.3, 87.9, 83.6, 69.1, 68.7, 66.4, 42.6. **IR** (ν_{max}, cm^{-1}) 2958 (m), 2925 (m), 2853 (m), 1728 (s), 1286 (s), 1267 (s), 1249 (s), 1120 (s), 1102 (s), 1015 (s). **HRMS** (ESI/QTOF) m/z: [M + Na]$^+$ Calcd for $C_{19}H_{16}ClINaO_3^+$ 476.9725; Found 476.9733.

2-Cyclohexyloxy-4-phenylbut-1-yn-4-yl 2-iodobenzoate (**10e**)

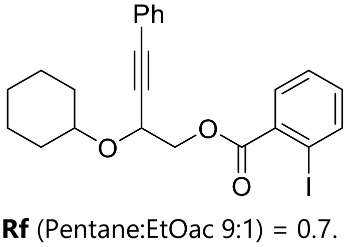

Obtained from cyclohexyl(vinyl)ether (**9e**, 0.053 mL, 0.047 g, 0.38 mmol, 1.5 equiv); PhEBX (**2**, 0.087 g, 0.52 mmol, 1.0 equiv); BIOAc (**7**, 0.038 g, 0.13 mmol, 0.5 equiv) and 4-ClCzIPN (**4b**, 5 mg, 5 µmol, 2 mol%) after 18 hours.

Column: Pentane:EtOAc, 100:0 95:5 to 8:2. Yield 62% (0.073 g, 0.15 mmol). Colorless oil.

Rf (Pentane:EtOac 9:1) = 0.7.

^{1}H NMR (400 MHz, Chloroform-*d*) δ 8.00 (dd, *J* = 7.9, 1.2 Hz, 1H, Ar*H*), 7.89 (dd, *J* = 7.8, 1.7 Hz, 1H, Ar*H*), 7.47 – 7.43 (m, 2H, Ph*H*), 7.42 – 7.36 (m, 1H, Ar*H*), 7.36 – 7.28 (m, 3H, Ph*H*), 7.16 (ddd, *J* = 7.9, 7.4, 1.7 Hz, 1H, Ar*H*), 4.80 (dd, *J* = 7.4, 4.5 Hz, 1H, OC*H*), 4.61 – 4.46 (m, 2H, OC*H*$_2$), 3.70 (tt, *J* = 9.3, 3.8 Hz, 1H, Cy*H*), 2.06 – 1.84 (m, 2H, Cy*H*), 1.84 – 1.65 (m, 2H, Cy*H*), 1.62 – 1.40 (m, 2H, Cy*H*), 1.40 – 1.17 (m, 4H, Cy*H*). **^{13}C NMR** (101 MHz, Chloroform-d) δ 166.1, 141.4, 134.7, 132.8, 131.8, 131.3, 128.6, 128.3, 127.9, 122.4, 94.4, 86.2, 85.6, 67.2, 65.4, 33.2, 31.5, 25.7, 24.0. **IR** (ν_{max}, cm^{-1}) 2997 (s), 2987 (s), 2971 (s), 2933 (s), 2901 (s), 1725 (s), 1287 (s), 1243 (s), 1133 (s), 1098 (s), 1075 (s), 1038 (s), 1016 (s). **HRMS** (ESI/QTOF) m/z: [M + Na]$^+$ Calcd for $C_{23}H_{23}INaO_3^+$ 497.0584; Found 497.0592.

2-Ethoxy-2-methyl-4-phenylbut-1-yn-4-yl 2-iodobenzoate (**10f**)

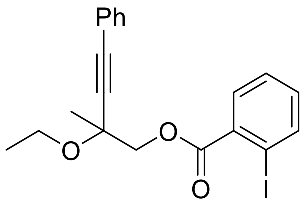

Obtained from 2-ethoxypro-1-ene (**9f**, 42 µL, 0.37 mmol, 1.5 equiv); PhEBX (**2**, 0.087 g, 0.25 mmol, 1.0 equiv); BIOAc (**7**, 0.038 g, 0.15 mmol, 0.5 equiv) and 4-ClCzIPN (**4b**, 5 mg, 5 µmol, 2 mol%) after 18 hours.
Column: Pentane:EtOAc, 9:1 to 7:3. Yield 82% (0.089 g, 0.21 mmol). Colorless oil.

Rf (Pentane:EtOAc 9:1) = 0.2.
^{1}H NMR (400 MHz, Chloroform-*d*) δ 7.91 (dd, *J* = 8.0, 1.2 Hz, 1H, Ar*H*), 7.82 (dd, *J* = 7.8, 1.7 Hz, 1H, Ar*H*), 7.38 – 7.26 (m, 3H, Ar*H* + Ph*H*), 7.26 – 7.19 (m, 3H, Ph*H*), 7.06 (ddd, *J* = 8.0, 7.4, 1.7 Hz, 1H, Ar*H*), 4.51 (d, *J* = 11.2 Hz, 1H, OC*H*$_2$), 4.35 (d, *J* = 11.2 Hz, 1H, OC*H*$_2$), 3.74 – 3.64 (q, *J* = 7.0 Hz, 2H, OC*H*$_2$CH$_3$), 1.57 (s, 3H, Me), 1.17 (t, *J* = 7.0 Hz, 3H, OCH$_2$C*H*$_3$). **^{13}C NMR** (101 MHz, Chloroform-*d*) δ 166.0, 141.4, 134.8, 132.8, 131.8, 131.3, 128.6, 128.3, 127.9, 122.3, 94.4, 87.7, 86.6, 72.3, 69.0, 60.2, 24.8, 15.8. **IR** (ν_{max}, cm^{-1}) 3004 (s), 2987 (s), 2972 (s), 2911 (s), 2901 (s), 2883 (s), 1732 (s), 1379 (s), 1288 (s), 1243 (s), 1125 (s), 1099 (s), 1066 (s), 1046 (s), 1016 (s). **HRMS** (ESI/QTOF) m/z: [M + Na]$^+$ Calcd for $C_{20}H_{19}INaO_3^+$ 457.0271; Found 457.0272.

1-(Benzyloxy)-2-(phenylethynyl)hexyl 2-iodobenzoate (**10ga**) and 3-(benzyloxy)-1-phenyloct-1-yn-4-yl 2-iodobenzoate (**10gb**)

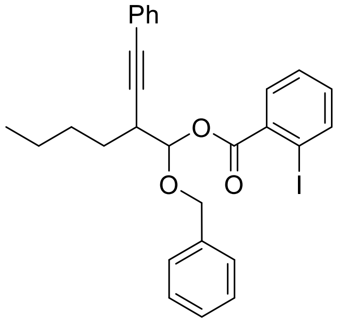

Obtained from (E)-((hex-1-en-1-yloxy)methyl)benzene (0.071 g, 0.38 mmol, 1.5 equiv); PhEBX (**2**, 0.087 g, 0.25 mmol, 1.0 equiv); BIOAc (**7**, 0.038 g, 0.13 mmol, 0.5 equiv) and 4-ClCzIPN (**4b**, 4 mg, 4 µmol, 2 mol%) after 18 hours.
First column: Pentane:EtOAc, 100:0 to 90:10, then second column: Pentane:EtOAc 98:2 to 92:8. Fraction 1: **10ga** (0.015 g, 0.028 mmol, 11% yield). Pale yellow oil. Fraction 2 (based on 1H NMR analysis): **10gb** (85 mol% and wt%) **10ga** (15 mol% and wt%) overall mass 0.014 g, 0.026 mmol. Pale yellow oil.
Overall yields:
10ga: 0.015 g + 0.002 g = 0.017 g, 0.032 mmol, 13% yield.
10gb: 0.015 g – 0.002 g = 0.013 g, 0.024 mmol, 10% yield

Major product **10ga** as a 1:1 mixture of diastereoisomers based on OC*H*$_2$Ph signals: 4.72 ppm and 4.69 ppm:
^{1}H NMR (400 MHz, Chloroform-*d*, 1:1 mixture of diastereoisomers) δ 7.94 (ddd, *J* = 7.8, 6.5, 1.3 Hz, 1H, Ar*H*), 7.79 (ddd, *J* = 7.9, 6.8, 1.8 Hz, 1H, Ar*H*), 7.37 – 7.16 (m, 11H, Ph*H* + Ar*H*), 7.09 (tdd, *J* = 7.9, 6.2, 1.8 Hz, 1H, Ar*H*), 6.18 (m, 1H, O$_2$C*H*), 4.86 (d, *J* = 6.7 Hz, 0.5H, OC*H*$_2$Ph), 4.83 (d, *J* = 6.6 Hz, 0.5H, OC*H*$_2$Ph), 4.72 (d, *J* = 7.2 Hz, 0.5H, OC*H*$_2$Ph), 4.69 (d, *J* = 7.1 Hz, 0.5H, OC*H*$_2$Ph), 3.04 (dt_{app}, *J* = 9.9, 5.0 Hz, 1H, C*H*R-alkyne), 1.77 – 1.45 (m, 2H, C*H*$_2$-CH$_2$-CH$_2$-CH$_3$), 1.43 – 1.13 (m, 4H, CH$_2$-C*H*$_2$-C*H*$_2$-CH$_3$), 0.84 (t, *J* = 7.2 Hz, 3H, CH$_2$-CH$_2$-CH$_2$-C*H*$_3$). **^{13}C NMR** (101 MHz, Chloroform-*d*, mixture of diasteroisomers, not all carbons are resolved) δ 166.2, 166.1, 141.5, 141.3, 137.1, 137.0, 134.8, 134.6, 132.9, 132.8, 131.8, 131.7, 131.2, 131.2, 128.5, 128.4, 128.2, 128.1, 128.0, 128.0, 127.9, 127.9, 123.5, 123.4, 98.9, 98.6, 88.0, 87.9, 83.5, 72.0, 72.0, 38.3, 38.1, 29.7, 29.4, 29.4, 29.1, 22.5, 22.5, 14.0. **IR** (ν_{max}, cm^{-1}) 3076 (w), 2986 (s), 2972 (s), 2931 (m), 2917 (s), 2893 (m), 1728 (m), 1454 (m), 1402 (m), 1271 (m), 1242 (m), 1090 (s), 1064 (s), 1050 (s). **HRMS** (ESI/QTOF) m/z: [M + K]$^+$ Calcd for $C_{28}H_{27}IKO_3^+$ 577.0636; Found 577.0647.

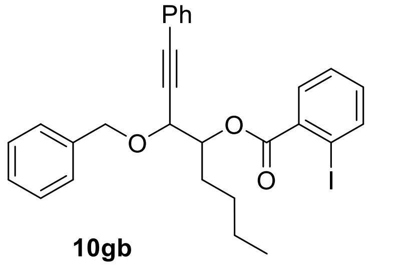

10gb

Minor product **10gb** as a 1:1 mixture of diastereoisomers based on peak at 4.90 ppm, isolated with 15% of **10ga**.
For ease of interpretation: the ^{1}H NMR data is reported without the signals corresponding to **10ga**; the ^{13}C NMR data is reported as measured.

^{1}H NMR (400 MHz, Chloroform-*d*) δ 7.99 (dd, *J* = 8.0, 1.3 Hz, 1H, Ar*H*), 7.83 (ddd, *J* = 9.7, 7.8, 1.8 Hz, 1H, Ar*H*), 7.47 – 7.43 (m, 2H, Ph*H*), 7.40 – 7.24 (m, 9H, Ph*H* + Ar*H*), 7.14 (tt, *J* = 7.5, 1.5 Hz, 1H, Ar*H*), 5.43 (tdd, *J* = 8.5, 6.0, 4.3 Hz, 1H, OC*H*), 4.90 (d, 0.5H, 1 diastereoisomer, OCH_2Ph) , 4.90 (d, 0.5H, 1 diastereoisomer, OCH_2Ph), 4.65 (d, *J* = 12.0 Hz, 1H, OCH_2Ph), 4.59 – 4.53 (m, 1H, OC*H*), 2.04 – 1.86 (m, 1H, CH_2-CH_2-CH_2-CH_3), 1.47 – 1.30 (m, 3H, CH_2-CH_2-CH_2-CH_3), 0.95 – 0.82 (m, 5H, CH_2-CH_2-CH_2-CH_3). **^{13}C NMR** (101 MHz, Chloroform-*d*) δ 166.1, 166.0, 141.5, 141.3, 137.6, 135.4, 135.2, 134.6, 132.9, 132.6, 132.5, 131.9, 131.8, 131.2, 131.1, 131.1, 128.7, 128.6, 128.4, 128.4, 128.4, 128.3, 128.3, 128.2, 128.1, 128.0, 128.0, 127.9, 127.9, 127.8, 125.5, 122.4, 122.4, 98.6, 94.2, 94.2, 87.7, 87.5, 84.8, 76.0, 75.6, 72.0, 70.9, 70.7, 70.5, 66.7, 38.8, 38.1, 34.5, 31.9, 30.4, 30.3, 29.9, 29.7, 29.6, 29.6, 29.5, 29.4, 29.2, 28.9, 27.7, 27.5, 25.1, 23.8, 23.0, 22.7, 22.6, 22.5, 14.1, 14.1, 14.0, 11.0. **IR** (ν_{max}, cm^{-1}) 3009 (m), 2996 (m), 2931 (m), 2916 (s), 2908 (m), 2892 (m), 1732 (m), 1394 (m), 1089 (m), 1076 (s), 1062 (s), 1024 (m). **HRMS** (ESI/QTOF) m/z: $[M + K]^+$ Calcd for $C_{28}H_{27}IKO_3^+$ 577.0636; Found 577.0646.

2-(Phenylethynyl)tetrahydro-2H-pyran-3-yl 2-iodobenzoate (**10ha**) 3-(phenylethynyl)tetrahydro-2H-pyran-2-yl 2-iodobenzoate (**10hb**); 4:1 regioisomeric mixture

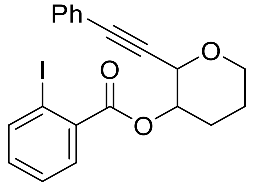

58%

:

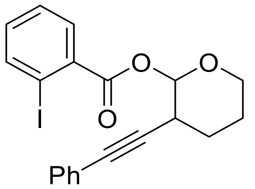

15%

Obtained from 2,3-dihydropyran (**9h**, 34 µL, 0.38 mmol, 1.5 equiv); PhEBX (**2**, 0.087 g, 0.25 mmol, 1.0 equiv); BIOAc (**7**, 0.114 g, 0.1 mmol, 1.5 equiv) and 4-ClCzIPN (**4b**, 5 mg, 5 µmol, 2 mol%) after 18 hours.
Column: Pentane:EtOAc, 9:1 to 8:2. Yield 73% (0.076 g, 0.17 mmol). Yellow oil.

Rf (pentane:EtOAc 9:1) = 0.3.
^{1}H NMR (400 MHz, Chloroform-*d*, 8:2 mixture of regioisomers major in **bold**, minor in *italic* those corresponding to both are both ***bold and italic***) δ *8.02* (dd, *J* = 7.6, 1.2 Hz, 0.2H, minor, Ar*H*), **8.00** (dd, *J* = 7.9, 1.2 Hz, 0.8H, major, Ar*H*), *7.91* (dd, *J* = 7.8, 1.7 Hz, 0.2H, minor, Ar*H*), **7.86** (dd, *J* = 7.8, 1.7 Hz, 0.8H, major, Ar*H*), ***7.48 – 7.36*** (m, 3H, Ph*H*), ***7.37 – 7.26*** (m, 3H, Ph*H* + Ar*H*), ***7.20 – 7.12*** (m, 1H, Ar*H*), *6.19* (d, *J* = 4.4 Hz, 0.2H, minor, $ArCO_2$-C*H*R-OR), **5.24 – 5.14** (m, 0.8H, major, alkynyl-C*H*R-OR), **4.74** (d, *J* = 5.3 Hz, 0.8H, major, $ArCO_2$-C*H*R_2), *4.11* (ddd, *J* = 11.6, 7.7, 3.5 Hz, 1H, RO-CH_2R), ***3.70*** (ddd, *J* = 11.1, 6.3, 3.7 Hz, 1H, RO-CH_2R), *3.06* (dt, *J* = 6.5, 4.5 Hz, 0.2H, alkynyl-C*H*R_2, minor), ***2.36*** (ddd, *J* = 13.1, 8.8, 4.2 Hz, 1H, CH_2), ***2.06 – 1.85*** (m, 2H, CH_2), ***1.69*** (dtt, *J* = 13.3, 6.9, 3.8 Hz, 1H, CH_2). **^{13}C NMR** (101 MHz, Chloroform-*d*, mixture of regioisomers some carbons are not resolved) δ 165.7, 141.5, 141.3, 135.0, 133.0, 132.8, 131.9, 131.8, 131.4, 131.1, 128.7, 128.3, 128.2, 128.1, 128.0, 122.2, 95.3, 94.2, 87.8, 87.6, 84.6, 72.0, 69.0, 64.9, 64.4, 32.4, 29.7, 26.2, 25.8, 22.5, 22.3. **IR** (ν_{max}, cm^{-1}) 3060 (m), 2987 (s), 2901 (s), 1725 (m), 1428 (m), 1379 (m), 1249 (s), 1078 (s), 1044 (s). **HRMS** (ESI/QTOF) m/z: $[M + Na]^+$ Calcd for $C_{20}H_{17}INaO_3^+$ 455.0115; Found 455.0122.

2-(((Benzyloxy)carbonyl)(phenethyl)amino)-4-(4-(trifluoromethyl)phenyl)but-3-yn-1-yl 2-iodobenzoate (**12a**)

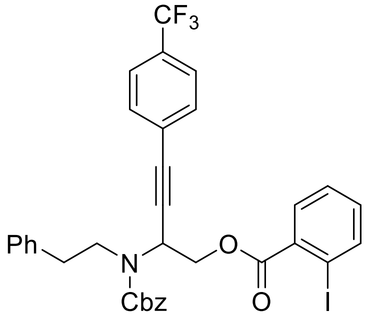

Obtained from benzyl phenethyl(vinyl)carbamate (**1c**, 0.106 g, 0.375 mmol, 1.5 equiv); *p*CF_3PhEBX (**11a**, 0.105 g, 0.250 mmol, 1.0 equiv); BIOAc (**7**, 0.038 g, 0.13 mmol, 0.5 equiv) and 4-ClCzIPN (**4b**, 5 mg, 5 µmol, 2 mol%) after 18 hours.

Column: Pentane:EtOAc, 10:0 to 9:1. Yield 75% (0.130 g, 0.186 mmol). Pale yellow oil.

Rf (Pent.:EtOAc 9:1) = 0.25.

^{1}H NMR (400 MHz, Acetonitrile-*d*$_3$, 6:4 mixture of rotamers) δ 8.02 (dd, *J* = 8.0, 1.1 Hz, 1H, Ar*H*), 7.88 - 7.72 (m, 1H, Ar*H*), 7.72 - 7.56 (m, 5H, Ph*H* + Ar*H*), 7.50 - 7.06 (m, 11H, Ph*H* + Ar*H*), 5.65 (bs, 0.6H, major, NC*H*), 5.50 (bs, 0.4H, minor, NC*H*), 5.13 (m, 2H, PhC*H*$_2$O), 4.72 - 4.41 (m, 2H, OC*H*$_2$), 3.62 (t, *J* = 8.1 Hz, 2H, C*H*$_2$), 3.17 - 2.85 (m, 2H, C*H*$_2$). **^{13}C NMR** (101 MHz, Acetonitrile-*d*$_3$, mixture of rotamers, 2 carbons not resolved) δ 166.4, 156.4, 141.9, 139.7, 137.3, 135.5, 133.7, 132.8, 131.4, 130.41 (q, *J* = 32.6 Hz), 129.3, 129.1, 129.0, 128.9, 128.76 - 128.28 (m), 126.9, 126.6, (q, *J* = 4.0 Hz), 123.3, 93.9, 86.9, 85.0, 67.8, 65.0, 48.7, 47.2, 36.5. **^{19}F NMR** (376 MHz, Acetonitrile-*d*$_3$) δ -63.4. **IR** (ν_{max}, cm^{-1}) 2986 (s), 2970 (s), 2961 (s), 2934 (s), 2901 (s), 1733 (s), 1715 (s), 1705 (s), 1699 (s), 1685 (s), 1410 (s), 1322 (s), 1247 (s). **HRMS** (ESI/QTOF) m/z: [M + H]$^+$ Calcd for $C_{34}H_{28}F_3INO_4^+$ 698.1010; Found 698.1006.

2-(((Benzyloxy)carbonyl)(phenethyl)amino)-4-(4-bromophenyl)but-3-yn-1-yl 2-iodobenzoate (**12b**)

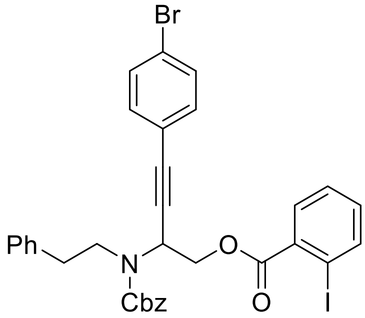

Obtained from benzyl phenethyl(vinyl)carbamate (**1c**, 0.106 g, 0.375 mmol, 1.2 equiv); *p*BrPhEBX (**11b**, 0.107 g, 0.250 mmol, 1.0 equiv); BIOAc (**7**, 0.038 g, 0.13 mmol, 0.5 equiv) and 4-ClCzIPN (**4b**, 4 mg, 5 µmol, 2 mol%) after 18 hours.

Column: Pentane:EtOAc, 10:0 to 9:1. Yield 76% (0.191 g, 0.135 mmol). Pale yellow oil.

Rf (Pent.:EtOAc 9:1) = 0.25.

^{1}H NMR (400 MHz, Acetonitrile-*d*$_3$) δ 8.05 (d, *J* = 7.9 Hz, 1H), 7.77 (d, *J* = 7.8 Hz, 1H), 7.57 (d, *J* = 8.2 Hz, 2H), 7.52 – 7.07 (m, 13H), 5.61 (s, 1H), 5.22 – 5.03 (m, 2H), 4.66 – 4.47 (m, 2H), 3.63 (dq, *J* = 15.1, 7.6 Hz, 2H), 3.36 (q, *J* = 6.8 Hz, 0H), 3.03 (dq, *J* = 16.5, 8.6, 7.3 Hz, 2H), 2.80 (t, *J* = 7.2 Hz, 0H).

^{1}H NMR (400 MHz, Acetonitrile-*d*$_3$, 9:1 mixture of rotamers)[31] δ 8.05 (dd, *J* = 8.0, 1.1 Hz, 1H, Ar*H*), 7.80 – 7.73 (m, 1H, Ar*H*), 7.61 – 7.53 (m, 2H, Ar*H* or Ph*H*), 7.47 (td, *J* = 7.6, 1.2 Hz, 1H, Ar*H*), 7.43 – 7.25 (m, 7H, Ph*H* + Ar*H*), 7.29 – 7.16 (m, 5H, Ar*H*), 7.16 – 7.11 (m, 1H, Ar*H*), 5.61- (bs, "0.7H", major, NC*H*), 5.48 (bs, "0.3H", minor, NC*H*), 5.15 (m, 2H, PhC*H*$_2$O), 4.56 (q, *J* = 5.4, 4.3 Hz, 2H, OC*H*$_2$), 3.64 (dq, *J* = 17.8, 7.6, 7.2 Hz, 1.8H, major, C*H*$_2$), 3.36 (q, *J* = 6.8 Hz, 0.2H, minor, C*H*$_2$), 3.00 (t, *J* = 8.6 Hz, 1.8H, major, C*H*$_2$), 2.80 (t, *J* = 7.2 Hz, 0.2H, minor, C*H*$_2$). **^{13}C NMR** (101 MHz, Acetonitrile-*d*$_3$, mixture of rotamers,

[31] The rotamer ratio was based on the signals at 3.00 and 2.80 ppm as they are better defined; the proton signals associated to the NC*H* (5.61 and 5.48 ppm) are broad signals therefore precise integration cannot be guaranteed.

not all peaks are resolved) δ 166.4, 156.4, 141.9, 139.8, 137.4, 135.5, 133.9, 133.7, 132.4, 132.1, 131.4, 129.3, 129.1, 129.0, 128.9, 128.9, 128.6, 126.9, 123.3, 121.7, 93.9, 85.5, 85.3, 67.8, 65.1, 48.7, 47.1, 36.5. **IR** (ν_{max}, cm^{-1}) 2987 (s), 2972 (s), 2901 (s), 1749 (s), 1470 (s), 1419 (s), 1376 (s), 1286 (s), 1242 (s), 1133 (s), 1076 (s), 1048 (s), 1017 (s). **HRMS** (ESI/QTOF) m/z: $[M + Na]^+$ Calcd for $C_{20}H_{15}{}^{79}BrINNaO_4^+$ 561.9121; Found 561.9118.

4-(2-Bromophenyl)-2-(2-oxooxazolidin-3-yl)but-3-yn-1-yl 2-iodobenzoate (**12c**)

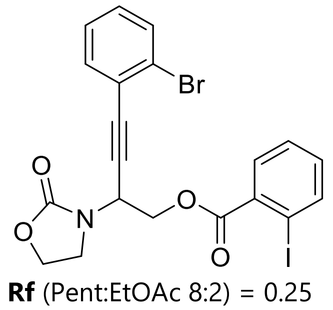

Obtained from *N*-vinyloxazolidin-2-one (**1a**, 0.042 g, 0.37 mmol, 1.5 equiv); *o*BrPhEBX (**11c**, 0.107 g, 0.250 mmol, 1.0 equiv); BIOAc (**7**, 0.038 g, 0.15 mmol, 0.5 equiv) and 4-ClCzIPN (**4b**, 5 mg, 5 µmol, 2 mol%) after 18 hours.

Column: Pentane:EtOAc, 8:2 to 5:5. Yield 56% (0.080 g, 0.14 mmol). Yellow oil.

Rf (Pent:EtOAc 8:2) = 0.25

^{1}H NMR (400 MHz, Chloroform-*d*) δ 8.00 (dd, *J* = 7.9, 1.2 Hz, 1H, Ar*H*), 7.86 (dd, *J* = 7.8, 1.7 Hz, 1H, Ar*H*), 7.60 (dd, *J* = 8.0, 1.3 Hz, 1H, Ar*H*), 7.47 (dd, *J* = 7.7, 1.8 Hz, 1H, Ar*H*), 7.42 (td, *J* = 7.6, 1.2 Hz, 1H, Ar*H*), 7.29 (td, *J* = 7.6, 1.3 Hz, 1H, Ar*H*), 7.20 (dtd, *J* = 20.1, 7.8, 1.8 Hz, 2H, Ar*H*), 5.41 (dd, *J* = 8.8, 4.3 Hz, 1H, NC*H*), 4.80 (dd, *J* = 11.5, 8.8 Hz, 1H, CH_2), 4.51 (dd, *J* = 11.5, 4.3 Hz, 1H, CH_2), 4.48 – 4.33 (m, 2H, CH_2), 3.96 (dt, *J* = 9.4, 8.1 Hz, 1H, CH_2), 3.81 (td, *J* = 8.6, 5.6 Hz, 1H, CH_2). **^{13}C NMR** (101 MHz, Acetonitrile-d_3) δ 166.4, 158.5, 141.9, 135.5, 134.3, 133.7, 133.1, 131.5, 131.2, 128.9, 128.2, 125.6, 124.2, 93.9, 87.1, 85.4, 64.5, 63.2, 46.4, 42.0. **IR** (ν_{max}, cm^{-1}) 2987 (s), 2972 (s), 2901 (s), 1749 (s), 1470 (s), 1419 (s), 1376 (s), 1286 (s), 1242 (s), 1133 (s), 1076 (s), 1048 (s), 1017 (s). **HRMS** (ESI/QTOF) m/z: $[M + Na]^+$ Calcd for $C_{20}H_{15}{}^{79}BrINNaO_4^+$ 561.9121; Found 561.9118.

4-(3-Fluorophenyl)-2-(2-oxooxazolidin-3-yl)but-3-yn-1-yl 2-iodobenzoate (**12d**)

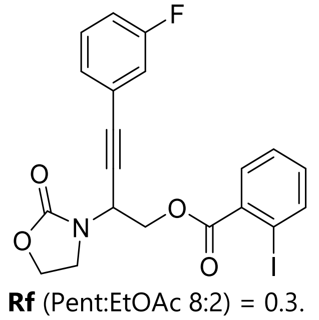

Obtained from *N*-vinyloxazolidin-2-one (**1a**, 0.042 g, 0.37 mmol, 1.5 equiv); *m*FPhEBX (**11d**, 0.092 g, 0.25 mmol, 1.0 equiv); BIOAc (**7**, 0.038 g, 0.15 mmol, 0.5 equiv) and 4-ClCzIPN (**4b**, 5 mg, 5 µmol, 2 mol%) after 18 hours.

Column: Pentane:EtOAc, 8:2 to 5:5. Yield 32% (0.038 g, 0,079 mmol). Yellow oil.

Rf (Pent:EtOAc 8:2) = 0.3.

^{1}H NMR (400 MHz, Chloroform-*d*) δ 7.92 (dd, *J* = 7.9, 1.2 Hz, 1H, Ar*H*), 7.76 (dd, *J* = 7.8, 1.7 Hz, 1H, Ar*H*), 7.34 (td, *J* = 7.6, 1.2 Hz, 1H, Ar*H*), 7.28 – 7.18 (m, 1H, Ar*H*), 7.15 (dt, *J* = 7.7, 1.2 Hz, 1H, Ar*H*), 7.14 – 7.02 (m, 2H, Ar*H*), 7.00 (tdd, *J* = 8.4, 2.6, 1.1 Hz, 1H, Ar*H*), 5.30 (dd, *J* = 8.9, 4.4 Hz, 1H, NC*H*), 4.69 (dd, *J* = 11.5, 8.9 Hz, 1H, CH_2), 4.44 – 4.25 (m, 3H, CH_2), 3.81 – 3.71 (m, 1H, CH_2), 3.75 – 3.66 (m, 1H, CH_2). **^{13}C NMR** (101 MHz, Chloroform-*d*) δ 165.9, 162.3 (d, *J* = 247.3 Hz), 157.9, 141.5, 134.1, 133.1, 131.3, 130.2 (d, *J* = 8.5 Hz), 128.2, 127.9 (d, *J* = 3.1 Hz), 123.3 (d, *J* = 9.3 Hz), 118.8 (d, *J* = 23.0 Hz), 116.6 (d, *J* = 21.0 Hz), 94.3, 86.1 (d, *J* = 3.3 Hz), 81.9, 63.6, 62.4, 46.1, 41.3. **^{19}F NMR** (377 MHz, Chloroform-*d*) δ -112.3. **IR** (ν_{max}, cm^{-1}) 2973 (s), 2932 (m), 2889 (m), 1734 (s), 1581 (s), 1485 (s), 1419 (s), 1376 (s), 1286 (s), 1246 (s), 1226 (s), 1172 (s), 1152 (s), 1133 (s), 1093 (s), 1045 (s), 1016 (s). **HRMS** (ESI/QTOF) m/z: $[M + H]^+$ Calcd for $C_{20}H_{16}FINO_4^+$ 480.0103; Found 480.0098.

4-(4-Methylphenyl)-2-(2-oxooxazolidin-3-yl)but-3-yn-1-yl 2-iodobenzoate (**12e**)

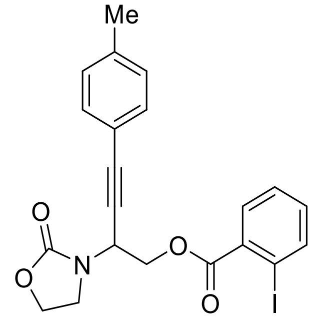

Obtained from *N*-vinyloxazolidin-2-one (**1a**, 0.042 g, 0.37 mmol, 1.5 equiv); *p*TolEBX (**11e**, 0.91 g, 0.25 mmol, 1.0 equiv); BIOAc (**7**, 0.038 g, 0.15 mmol, 0.5 equiv) and 4-ClCzIPN (**4b**, 5 mg, 5 µmol, 2 mol%) after 18 hours.
Column: Pentane:EtOAc, 8:2 to 5:5. Yield 59% (0.070 g, 0.15 mmol). Yellow oil.

Rf (Pent:EtOAc 8:2) = 0.3.
^{1}H NMR (400 MHz, Acetonitrile-d_3) δ 8.04 (dd, *J* = 8.0, 1.2 Hz, 1H, Ar*H*), 7.80 (dd, *J* = 7.8, 1.7 Hz, 1H, Ar*H*), 7.49 (td, *J* = 7.6, 1.2 Hz, 1H, Ar*H*), 7.41 – 7.33 (m, 2H, Ar*H*), 7.25 (ddd, *J* = 8.0, 7.4, 1.7 Hz, 1H, Ar*H*), 7.22 – 7.16 (m, 2H, Ar*H*), 5.23 (dd, *J* = 8.6, 4.6 Hz, 1H, NC*H*), 4.65 (dd, *J* = 11.4, 8.6 Hz, 1H, CH_2), 4.49 (dd, *J* = 11.4, 4.6 Hz, 1H, CH_2), 4.41 – 4.27 (m, 2H, CH_2), 3.86 – 3.70 (m, 2H, CH_2), 2.34 (s, 3H, Me). **^{13}C NMR** (101 MHz, Acetonitrile-d_3) δ 166.5, 158.5, 141.8, 140.2, 135.6, 133.7, 132.2, 131.4, 129.8, 128.9, 119.2, 93.8, 87.2, 81.4, 64.5, 63.2, 46.3, 41.7, 21.1. **IR** (ν_{max}, cm^{-1}) 2998 (s), 2972 (s), 2943 (s), 2901 (s), 1748 (s), 1481 (s), 1408 (s), 1377 (s), 1286 (s), 1248 (s), 1075 (s), 1066 (s), 1038 (s), 1016 (s). **HRMS** (ESI/QTOF) m/z: [M + Na]$^+$ Calcd for $C_{21}H_{18}INNaO_4^+$ 498.0173; Found 498.0182.

2-(((Benzyloxy)carbonyl)(phenethyl)amino)-4-(triisopropylsilyl)but-3-yn-1-yl 2-iodobenzoate (**12f**)

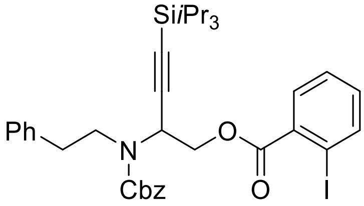

Obtained from benzyl phenethyl(vinyl)carbamate (**1c**, 0.106 g, 0.375 mmol, 1.5 equiv); **11f** (0.107 g, 0.25 mmol, 1.0 equiv); BIOAc (**7**, 0.038 g, 0.15 mmol, 0.5 equiv) and 4-ClCzIPN (**4b**, 5 mg, 5 µmol, 2 mol%) after 18 hours.
Column: Pentane:EtOAc, 93:7 to 9:1. Yield 9% (0.007 g, 0.03 mmol). Colorless oil.

Rf (pentane:EtOAc 8:2) = 0.15.
^{1}H NMR (400 MHz, Acetonitrile-d_3, 6:3 mixture of rotamers) δ 8.02 (dd, *J* = 7.9, 1.2 Hz, 1H, Ar*H*), 7.75 (d, *J* = 7.7 Hz, 1H, Ar*H*), 7.43 (dd, *J* = 8.4, 7.2 Hz, 1H, Ar*H*), 7.34 (d, *J* = 11.1 Hz, 6H, Ph*H* + Ar*H*), 7.29 – 7.14 (m, 4H, Ph*H*), 7.13 – 7.09 (m, 1H, Ar*H*), 5.45 (s, 0.6H, NC*H*), 5.35 (s, 0.3H, NC*H*), 5.14 (d, *J* = 12.4 Hz, 1H, PhCH_2), 5.09 (s, 1H, PhCH_2), 4.44 (dd, *J* = 6.2, 3.6 Hz, 2H, OCH_2), 3.59 (dd, *J* = 10.2, 6.8 Hz, 2H, PhCH_2-CH_2), 3.01 (ddd, *J* = 13.0, 9.5, 6.3 Hz, 1H, NCH_2), 2.93 (s, 1H, NCH_2), 1.05 (d, *J* = 2.3 Hz, 18H, Si*i*Pr_3). **^{13}C NMR** (101 MHz, Acetonitrile-d_3) δ 166.2, 156.2, 141.9, 139.8, 137.4, 135.3, 133.7, 131.5, 129.4, 129.2, 129.1, 129.0, 128.8, 128.8, 128.6, 127.0, 126.9, 102.4, 94.1, 88.4, 68.9, 67.8, 65.5, 48.8, 46.9, 42.4, 36.4, 34.5, 32.2, 29.9, 23.0, 18.5, 18.4, 18.3, 18.2, 17.9, 14.0, 12.8, 11.9, 11.5, 11.2. **HRMS** (ESI/QTOF) m/z: [M + Na]$^+$ Calcd for $C_{36}H_{44}INNaO_4Si^+$ 732.1977; Found 732.1976.
*Unfortunately, insufficient quantities of compound **12f** was obtained to allow interpretable IR analysis.*

4-(4-((3-Bromopropoxy)carbonyl)phenyl)-2-(2-oxooxazolidin-3-yl)but-3-yn-1-yl 2-iodobenzoate (**12g**)

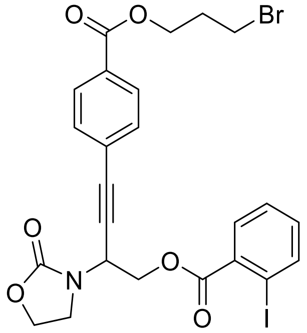

Obtained from *N*-vinyloxazolidin-2-one (**1a**, 0.042 g, 0.37 mmol, 1.5 equiv); **11f** (0.124 g, 0.250 mmol, 1.0 equiv); BIOAc (**7**, 0.038 g, 0.15 mmol, 0.5 equiv) and 4-ClCzIPN (**4b**, 5 mg, 5 μmol, 2 mol%) after 18 hours.
Column: Pentane:EtOAc, 8:2 to 5:5. Yield 51% (0.077 g, 0.12 mmol). Yellow oil.

Rf (pentane:EtOAc 8:2) = 0.15.
^{1}H NMR (400 MHz, Chloroform-*d*) δ 8.04 – 7.96 (m, 3, Ar*H*), 7.83 (dd, *J* = 7.8, 1.7 Hz, 1H, Ar*H*), 7.55 – 7.47 (m, 2H, Ar*H*), 7.42 (td, *J* = 7.6, 1.2 Hz, 1H, Ar*H*), 7.18 (ddd, *J* = 8.0, 7.4, 1.7 Hz, 1H, Ar*H*), 5.40 (dd, *J* = 8.8, 4.4 Hz, 1H, NC*H*), 4.78 (dd, *J* = 11.5, 8.9 Hz, 1H, OCH_2), 4.54 – 4.34 (m, 5H, CH_2 + CO_2CH_2), 3.92 – 3.74 (m, 2H, CH_2), 3.55 (t, *J* = 6.5 Hz, 2H, CH_2Br), 2.33 (p_{app}, *J* = 6.3 Hz, 2H, CH_2-CH_2Br). **^{13}C NMR** (101 MHz, Chloroform-*d*) δ 165.9, 165.6, 157.9, 141.5, 134.1, 133.2, 131.9, 131.3, 130.3, 129.6, 128.2, 126.3, 94.3, 86.5, 84.0, 63.6, 63.0, 62.4, 46.2, 41.3, 31.7, 29.3. **IR** (ν_{max}, cm^{-1}) 2987 (s), 2972 (s), 2920 (s), 2900 (s), 1749 (s), 1715 (s), 1480 (s), 1407 (s), 1382 (s), 1268 (s), 1249 (s), 1104 (s), 1045 (s), 1017 (s). **HRMS** (nanochip-ESI/LTQ-Orbitrap) m/z: [M + Na]$^+$ Calcd for $C_{24}H_{21}{}^{79}BrINNaO_6{}^+$ 647.9489; Found 647.9478.

4-(4-((Allyloxy)carbonyl)phenyl)-2-(2-oxooxazolidin-3-yl)but-3-yn-1-yl 2-iodobenzoate (**12h**)

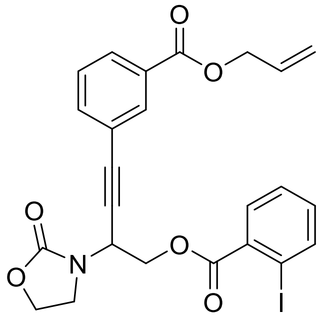

Obtained from *N*-vinyloxazolidin-2-one (**1a**, 0.042 g, 0.37 mmol, 1.5 equiv); **11g** (0.108 g, 0.250 mmol, 1.0 equiv); BIOAc (**7**, 0.038 g, 0.15 mmol, 0.5 equiv) and 4-ClCzIPN (**4b**, 5 mg, 5 μmol, 2 mol%) after 18 hours.
Column: Pentane:EtOAc, 8:2 to 5:5. Yield 37% (0.047 g, 0.093 mmol). Clear oil.

Rf (pentane:EtOAc 8:2) = 0.15.
^{1}H NMR (400 MHz, Chloroform-*d*) δ 8.13 (td, *J* = 1.7, 0.6 Hz, 1H, Ar*H*), 8.09 – 8.03 (m, 1H, Ar*H*), 8.00 (dd, *J* = 7.9, 1.2 Hz, 1H, Ar*H*), 7.84 (dd, *J* = 7.8, 1.7 Hz, 1H, Ar*H*), 7.63 (dt, *J* = 7.7, 1.4 Hz, 1H, Ar*H*), 7.43 (tdd, *J* = 7.7, 2.7, 0.9 Hz, 2H, Ar*H*), 7.18 (ddd, *J* = 8.0, 7.4, 1.7 Hz, 1H, Ar*H*), 6.05 (ddt, *J* = 17.2, 10.4, 5.7 Hz, 1H, C*H*=CH_2), 5.45 (q, *J* = 1.5 Hz, 1H, NC*H*), 5.38 (d, *J* = 4.4 Hz, 1H, CH=CH_2), 5.33 (q, *J* = 1.3 Hz, 1H, CH_2-CH=CH_2), 4.84 (dt, *J* = 5.7, 1.4 Hz, 2H, CH=CH_2), 4.78 (dd, *J* = 11.5, 8.9 Hz, 1H, CH_2), 4.53 – 4.33 (m, 3H, CH_2), 3.91 – 3.74 (m, 2H, CH_2). **^{13}C NMR** (101 MHz, Chloroform-*d*) δ 165.9, 165.3, 157.9, 141.4, 136.1, 134.1, 133.1, 133.1, 131.9, 131.3, 130.6, 130.2, 128.7, 128.2, 122.0, 118.7, 94.3, 86.3, 81.9, 66.0, 63.7, 62.4, 46.2, 41.3. **IR** (ν_{max}, cm^{-1}) 3004 (s), 2986 (s), 2972 (s), 2911 (s), 2900 (s), 2883 (s), 1732 (s), 1487 (s), 1419 (s), 1376 (s), 1285 (s), 1242 (s), 1226 (s), 1133 (s), 1101 (s), 1080 (s), 1038 (s), 1028 (s). **HRMS** (nanochip-ESI/LTQ-Orbitrap) m/z: [M + Na]$^+$ Calcd for $C_{24}H_{20}INNaO_6{}^+$ 568.0228; Found 568.0211.

2-Butoxy-4-(4-(trifluoromethyl)phenyl)but-3-yn-1-yl 2-iodobenzoate (**12i**)

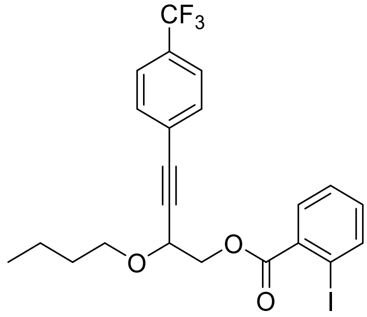

Obtained from *n*butyl vinyl ether (**9a**, 0.048 mL, 0.38 mmol, 1.5 equiv); **11a** (0.104 g, 0.250 mmol, 1.0 equiv); BIOAc (**7**, 0.038 g, 0.15 mmol, 0.5 equiv) and 4-ClCzIPN (**4b**, 5 mg, 5 µmol, 2 mol%) after 18 hours.

Column: Pentane:EtOAc, 8:2 to 5:5. Yield 62% (0.082 g, 0.15 mmol). Yellow oil.

Rf (pentane:EtOAc 9:1) = 0.55.

^{1}H NMR (400 MHz, Chloroform-*d*) δ 8.01 (dd, *J* = 8.0, 1.2 Hz, 1H, Ar*H*), 7.88 (dd, *J* = 7.8, 1.7 Hz, 1H, Ar*H*), 7.67 – 7.47 (m, 4H, Ar*H*), 7.40 (td, *J* = 7.6, 1.2 Hz, 1H, Ar*H*), 7.16 (td, *J* = 7.7, 1.7 Hz, 1H, Ar*H*), 4.69 (dd, *J* = 6.8, 4.8 Hz, 1H, OC*H*), 4.63 – 4.49 (m, 2H, OC*H*$_2$), 3.85 (dt, *J* = 9.2, 6.6 Hz, 1H, OC*H*$_2$), 3.56 (dt, *J* = 9.2, 6.5 Hz, 1H, OC*H*$_2$), 1.70 – 1.58 (m, 2H, C*H*$_2$-CH$_2$-CH$_3$), 1.55 – 1.33 (m, 2H, CH$_2$-C*H*$_2$-CH$_3$), 0.93 (t, *J* = 7.4 Hz, 3H, CH$_2$-CH$_2$-C*H*$_3$). **^{13}C NMR** (101 MHz, Chloroform-*d*) δ 166.0, 141.5, 134.6, 132.9, 132.1, 131.3, 130.4 (q, *J* = 33.0 Hz), 127.9, 126.0, 125.3 (q, *J* = 3.8 Hz), 123.8 (q, *J* = 272.3 Hz), 94.3, 87.4, 85.5, 69.4, 68.2, 66.5, 31.6, 19.3, 13.9. **^{19}F NMR** (376 MHz, Chloroform-*d*) δ -62.9. **IR** (ν_{max}, cm^{-1}) 1732 (s), 1321 (s), 1286 (s), 1243 (s), 1167 (s), 1125 (s), 1104 (s), 1092 (s), 1086 (s), 1066 (s), 1044 (s), 1016 (s). **HRMS** (ESI/QTOF) m/z: [M + Na]$^+$ Calcd for $C_{22}H_{20}F_3INaO_3^+$ 539.0301; Found 539.0306.

4-(4-((3-Bromopropoxy)carbonyl)phenyl)-2-butoxybut-3-yn-1-yl 2-iodobenzoate (**12j**)

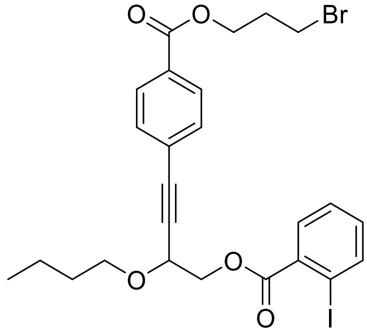

Obtained from *n*butyl vinyl ether (**9a**, 0.048 mL, 0.38 mmol, 1.5 equiv); **11f** (0.124 g, 0.250 mmol, 1.0 equiv); BIOAc (**7**, 0.038 g, 0.15 mmol, 0.5 equiv) and 4-ClCzIPN (**4b**, 5 mg, 5 µmol, 2 mol%) after 18 hours.

Column: Pentane:EtOAc, 98:2 to 90:10. Yield 52% (0.079 g, 0.13 mmol). Yellow oil.

Rf (pentane:EtOAc 9:1) = 0.5

^{1}H NMR (400 MHz, Chloroform-*d*) δ 7.96 – 7.87 (m, 3H, Ar*H*), 7.80 (dd, *J* = 7.8, 1.7 Hz, 1H, Ar*H*), 7.49 – 7.40 (m, 2H, Ar*H*), 7.33 (td, *J* = 7.6, 1.2 Hz, 1H, Ar*H*), 7.09 (td, *J* = 7.7, 1.7 Hz, 1H, Ar*H*), 4.62 (dd, *J* = 6.7, 4.8 Hz, 1H, OC*H*), 4.57 – 4.47 (m, 2H, C*H*$_2$), 4.40 (t, *J* = 6.0 Hz, 2H, C*H*$_2$), 3.78 (dt, *J* = 9.2, 6.6 Hz, 1H, C*H*$_2$), 3.48 (dt, *J* = 9.8, 6.5 Hz, 3H, C*H*$_2$), 2.25 (p_{app}, *J* = 6.3 Hz, 2H, C*H*$_2$-CH$_2$Br), 1.65 – 1.49 (m, 2H, C*H*$_2$-CH$_2$-CH$_3$), 1.49 – 1.28 (m, 2H, CH$_2$-C*H*$_2$-CH$_3$), 0.85 (t, *J* = 7.4 Hz, 2H, CH$_2$-CH$_2$-C*H*$_3$). **^{13}C NMR** (101 MHz, Chloroform-*d*) δ 166.0, 165.7, 141.4, 134.6, 132.9, 131.8, 131.3, 129.8, 129.5, 129.4, 127.9, 127.1, 94.3, 88.0, 86.1, 69.4, 68.2, 66.5, 62.9, 31.8, 31.6, 29.4, 19.3, 13.9. **IR** (ν_{max}, cm^{-1}) 2997 (s), 2987 (s), 2977 (s), 2971 (s), 2901 (s), 2892 (s), 1732 (m), 1394 (m), 1266 (s), 1242 (s), 1088 (s), 1066 (s), 1040 (s). **HRMS** (ESI/QTOF) m/z: [M + Na]$^+$ Calcd for $C_{25}H_{26}BrINaO_5^+$ 634.9901; Found 634.9907

Gram scale synthesis and product modification

Sunlight experiment

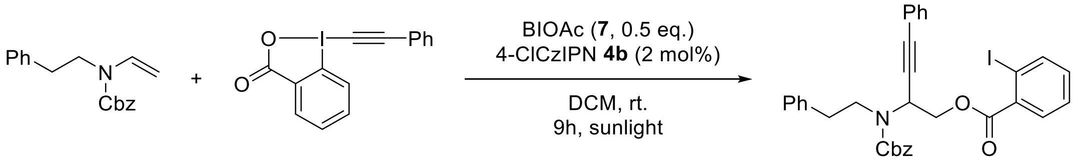

1c **2** **3c**

An oven-dried flat-bottomed 2 mL screw-cap vial equipped with a magnetic stirrer was charged with Ph-EBX (**2**, 0.087 g, 0.25 mmol, 1.0 equiv), BIOAc (**7**, 0.038 g, 0.13 mmol, 0.5 equiv) and 4-ClCzIPN (**4b**, 5 mg, 5 µmol, 2 mol%). The reaction vessel was sealed with a rubber septum. Following three vacuum/nitrogen cycles, the vial was refilled with argon. Then, dry, degassed (*via* freeze-pump-thaw technique) CH_2Cl_2 (10.0 mL, 0.29 M) was added. Benzyl phenethyl(vinyl) carbamate (**1c**, 0.106 g, 0.375 mmol, 1.5 equiv) was then added *via* syringe. The reaction was placed in direct sunlight for 5 h under stirring then 4 h without stirring. A deactivated solid deposit for flash chromatography was prepared: a slurry of SiO_2 (ca. 3 g) and ca. 0.2 mL of Et_3N was prepared and then combined with the crude reaction mixture before concentration. The crude was then purified though flash chromatography (biotage: SiO_2 25 g, Pentane:EtOAc 0% to 15%) affording **3c** (0.107 g, 0.170 mmol, 68% yield).

Weather report:[32] sunny with partial clouds temperature from 23-30 °C.

Gram scale

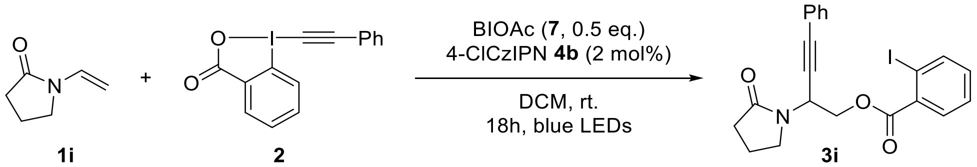

1i **2** **3i**

An oven-dried flat-bottomed 10 mL snap-cap vial equipped with a magnetic stirrer was charged with Ph-EBX (**2**, 1.00 g, 2.87 mmol, 1.0 equiv), BIOAc (**7**, 0.440 g, 2.87 mmol, 0.5 equiv) and 4-ClCzIPN (**4b**, 61 mg, 57 µmol, 2 mol%). The reaction vessel was sealed with a rubber septum. Following three vacuum/nitrogen cycles, the vial was refilled with argon. Then, dry, degassed (*via* freeze-pump-thaw technique) CH_2Cl_2 (10.0 mL, 0.29 M) was added. *N*-vinyl pyrrolidinone (**1i**, 0.46 mL, 4.3 mmol, 1.5 equiv) was then added *via* syringe. The reaction was irradiated overnight (18 h) with blue LED strips under ventilation (T = ca. 25°C) and stirring. A deactivated solid deposit for flash chromatography was prepared: a slurry of SiO_2 (ca. 20 g) and ca. 1 mL of Et_3N was prepared and then combined with the crude reaction mixture before concentration. The crude was then purified though flash chromatography (biotage : SiO_2 120 g, Pentane:EtOAc 20% to 50%) affording **3i** (0.998 g, 1.83 mmol, 64% yield).

[32] https://www.historique-meteo.net/europe/suisse/lausanne/2019/07/24/ consulted on the 02.06.2020

1-(1-Hydroxy-4-phenylbut-3-yn-2-yl)pyrrolidin-2-one (**14**)

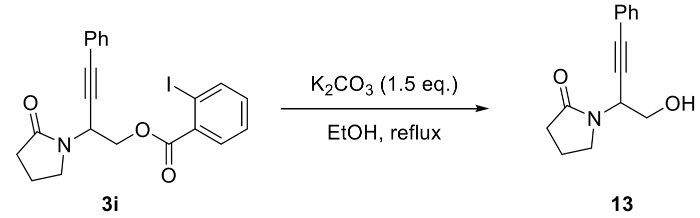

Following a modified reported procedure,[33] a flame dried 5 mL microwave vial with a rubber septum and magnetic stirring bar was charged with **3i** (100.0 mg, 217.0 µmol, 1.00 equiv), potassium;carbonate (0.045 mg, 0.32 mmol, 1.50 equiv) and ethanol (2 mL). The vial was sealed and stirred at reflux for 2h. At this time TLC showed full conversion of the starting material. The solvent was evaporated on top a silica plug then submitted to flash chromatography (Biotage SiO_2 12 g: CH_2Cl_2:AcOEt:MeOH 80:20:0, 60:40:0, 80:0:20) to afford 1-(1-hydroxy-4-phenylbut-3-yn-2-yl)pyrrolidin-2-one (**14**, 0.048 g, 0.21 mmol, 96% yield) as a yellowish oil.

Rf (CH_2Cl_2:MeOH 8:2) = 0.3.

^{1}H NMR (400 MHz, Chloroform-*d*) δ 7.45 (dd, *J* = 7.4, 2.1 Hz, 2H, Ar*H*), 7.34 (td, *J* = 4.8, 2.3 Hz, 3H, Ar*H*), 5.27 (dd, *J* = 8.1, 4.8 Hz, 1H, N-C*H*R_2), 3.92 (dd, *J* = 11.4, 4.7 Hz, 1H, O-C*H*$_2$R), 3.82 (dd, *J* = 11.4, 8.1 Hz, 1H, O-C*H*$_2$R), 3.74 – 3.65 (m, 1H, cyclic-C*H*$_2$-N), 3.60 (dt, *J* = 9.5, 7.0 Hz, 1H, cyclic-C*H*$_2$-N), 3.05 (bs, 1H, OH), 2.54 – 2.44 (m, 2H, cyclic-CO-C*H*$_2$), 2.17 – 2.05 (m, 2H, cyclic-C*H*$_2$). **^{13}C NMR** (101 MHz, Chloroform-*d*) δ 175.8, 131.9, 128.7, 128.4, 122.1, 86.0, 83.0, 63.7, 47.0, 44.4, 31.3, 17.9. **IR** (ν_{max}, cm^{-1}) 3373 (w), 2938 (w), 2879 (w), 1662 (s), 1420 (m), 1287 (m), 1069 (m). **HRMS** (ESI/QTOF) m/z: $[M + Na]^+$ Calcd for $C_{14}H_{15}NNaO_2^+$ 252.0995; Found 252.1001.

2-Butoxy-4-phenylbut-3-yn-1-ol (**15**)

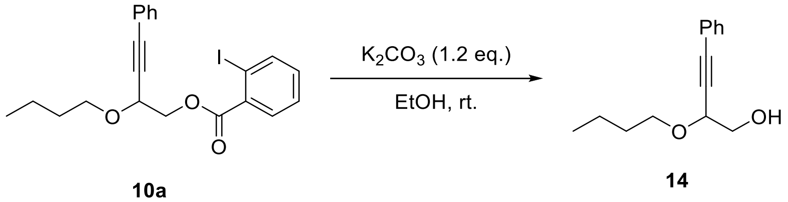

Following a slightly modified reported procedure,[33] a flame dried 5 mL microwave vial with a rubber septum and magnetic stirring bar was charged with **10a** (26.0 mg, 58.0 µmol, 1.00 equiv), potassium;carbonate (9.62 mg, 69.6 µmol, 1.20 equiv) and ethanol (600 µL). The mixture was stirred at room temperature for 60 h, then the solvent was evaporated and submitted to flash chromatography (SiO_2, CH_2Cl_2: EtOAc) to afford (**15**, 11.5 mg, 52.7 mmol, 91% yield).

Rf (CH_2Cl_2) = 0.3.

^{1}H NMR (400 MHz, Chloroform-*d*) δ 7.48 – 7.41 (m, 2H, Ph*H*), 7.36 – 7.27 (m, 3H, Ph*H*), 4.36 (dd, *J* = 6.2, 5.5 Hz, 1H, OC*H*), 3.87 (dt, *J* = 9.3, 6.6 Hz, 1H, OC*H*$_2$), 3.80 (t, *J* = 5.1 Hz, 2H, OC*H*$_2$), 3.51 (dt, *J* = 9.2, 6.6 Hz, 1H, OC*H*$_2$), 2.26 (d, *J* = 18.6 Hz, 1H, OH), 1.70 – 1.58 (m, 2H, C*H*$_2$-CH_2-

[33]Hari, D. P. and Waser, J. *J. Am. Chem. Soc.* **2016**, *138*, 2190

CH_3), 1.49 – 1.35 (m, 2H, CH_2-C*H*$_2$-CH_3), 0.95 (t, *J* = 7.4 Hz, 3H, CH_2-CH_2-C*H*$_3$). **^{13}C NMR** (101 MHz, Chloroform-*d*) δ 131.8, 128.6, 128.3, 122.3, 86.8, 85.2, 70.9, 69.3, 65.4, 31.7, 19.3, 13.9. **IR** (ν_{max}, cm^{-1}) 3426 (m), 3006 (s), 2987 (s), 2958 (s), 2892 (s), 2867 (s), 1382 (m), 1103 (s), 1066 (s), 1047 (s). **HRMS** (ESI/QTOF) m/z: $[M + Na]^+$ Calcd for $C_{14}H_{18}NaO_2^+$ 241.1199; Found 241.1195.

2-(Phenethylamino)-4-phenylbut-3-yn-1-yl 2-iodobenzoate (**16**)

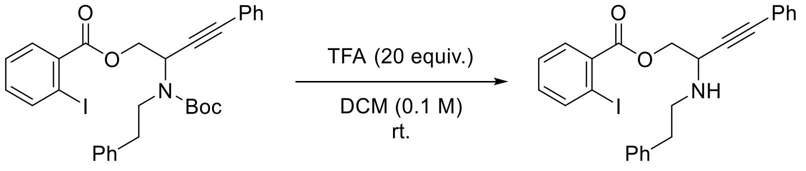

3b **15**

To a 5 mL vial equipped with a magnetic stirrer, **3b** (0.047 g, 0.078 mmol, 1 equiv) were dissolved in CH_2Cl_2 (0.8 mL), trifluoroacetic acid (130 µL, 1.90 mmol, 25 equiv) was added dropwise at room temperature. The reaction was stirred for 1h 30 min. At this time, TLC showed full conversion of **3b**. TFA and CH_2Cl_2 were evaporated off. The crude was diluted with Et_2O (1 mL) and basified with Na_2CO_3 aq. sat. (0.5 mL) until ph > 11. The aqueous layer was extracted a second time with Et_2O (1.5 mL). The combined organic layers were combined, dried over $MgSO_4$, then passed through a short plug of deactivated silica. The plug was then washed through with EtOAc. Upon concentration pure **15** was obtained as a colorless oil (0.029 g, 0.059, 74% yield).

Rf (CH_2Cl_2) = 0.15.

^{1}H NMR (400 MHz, Acetonitrile-*d*$_3$) δ 8.02 (dd, *J* = 8.0, 1.1 Hz, 1H, Ar*H*), 7.74 (dd, *J* = 7.7, 1.7 Hz, 1H, Ar*H*), 7.47 (td, *J* = 7.6, 1.2 Hz, 1H, Ar*H*), 7.43 – 7.36 (m, 2H, Ph*H*), 7.39 – 7.30 (m, 3H, Ph*H*), 7.30 – 7.21 (m, 5H, Ph*H*), 7.24 – 7.10 (m, 1H, Ar*H*), 4.68 – 4.31 (m, 2H, O-C*H*$_2$), 4.16 – 3.99 (m, 1H, N-C*H*R), 3.17 (dt, *J* = 11.2, 7.2 Hz, 1H, N-C*H*$_2$R), 2.96 (ddd, *J* = 11.3, 7.6, 6.1 Hz, 1H, N-C*H*$_2$R), 2.91 – 2.67 (m, 2H, Ph-C*H*$_2$R), 2.26 – 2.00 (m, 1H, R_2N*H*). **^{13}C NMR** (101 MHz, Acetonitrile-*d*$_3$) δ 166.8, 141.7, 141.0, 136.2, 133.5, 132.1, 131.4, 129.3, 129.1, 129.0, 128.9, 128.9, 126.6, 123.3, 93.7, 88.3, 84.7, 67.4, 50.0, 48.9, 36.5. **IR** (ν_{max}, cm^{-1}) 3312 (w), 3060 (w), 3025 (w), 2955 (m), 2899 (m), 2847 (w), 1949 (w), 1730 (s), 1583 (m), 1490 (m), 1442 (m), 1285 (s), 1247 (s), 1132 (s), 1099 (s), 1015 (s). **HRMS** (ESI/QTOF) m/z: $[M + H]^+$ Calcd for $C_{25}H_{23}INO_2^+$ 496.0768; Found 496.0769.

NMR spectra for synthesised alkenes and new compounds

Starting materials

1-[(4-(3-bromoprop-1-yl-benzoate)ethynyl]-1,2-benziodoxol-3(1H)-one (**11g**)

^{1}H NMR (400 MHz, Chloroform-*d*)

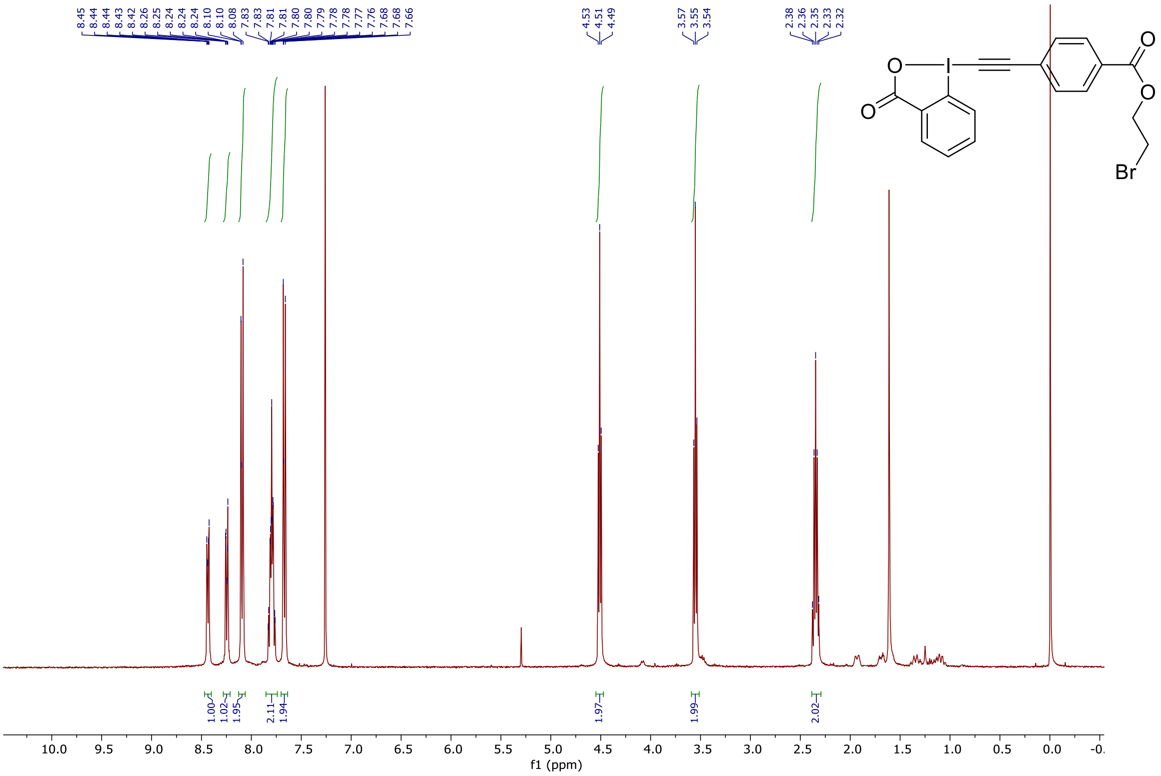

^{13}C NMR (101 MHz, Chloroform-*d*)

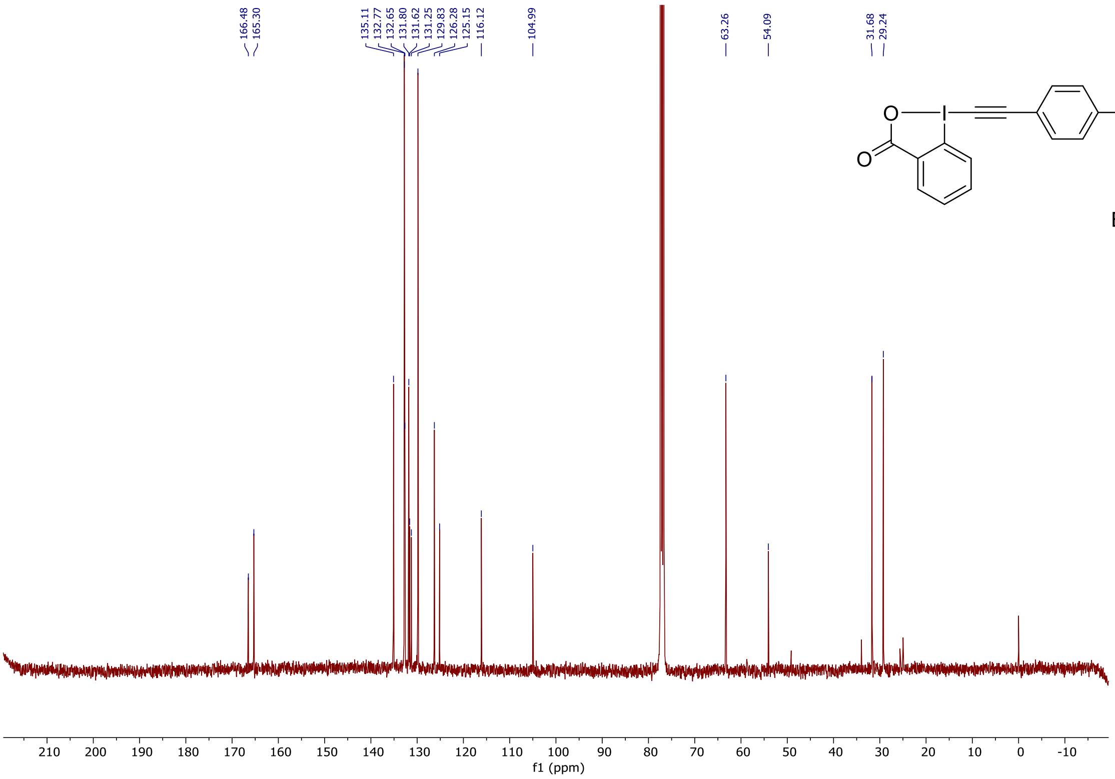

1-[(3-(prop-2-en-1-yl-benzoate)ethynyl]-1,2-benziodoxol-3(1*H*)-one (**11h**)

^{1}H NMR (400 MHz, Chloroform-*d*)

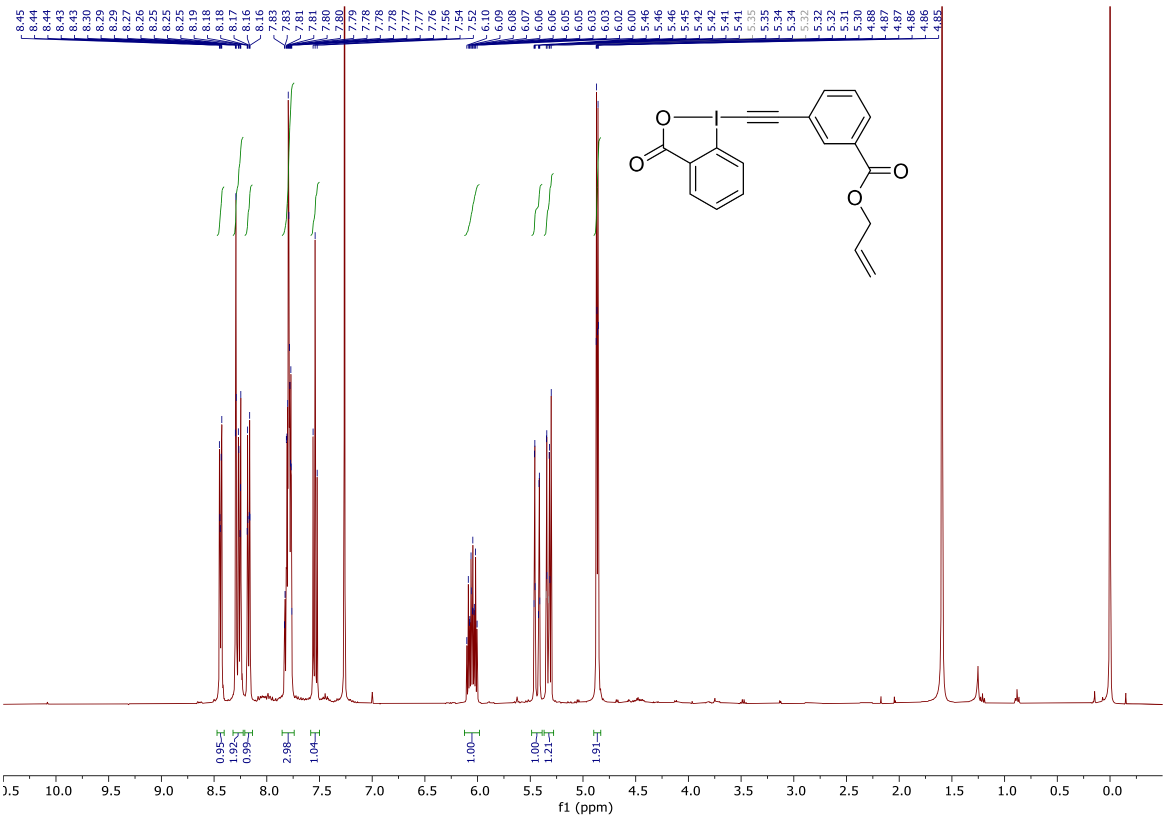

^{13}C NMR (101 MHz, Chloroform-*d*)

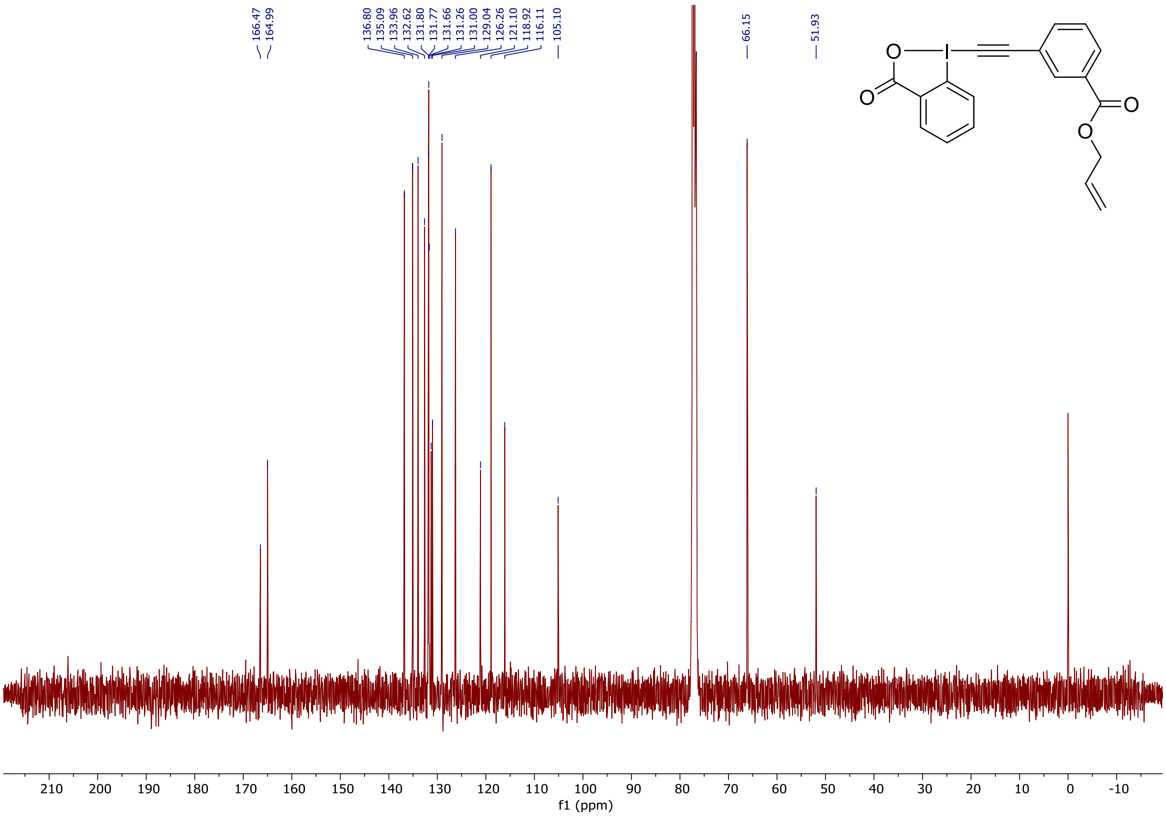

<u>*N*-vinyloxazolidin-2-one (**1a**)</u>

^{1}H NMR (400 MHz, Chloroform-*d*)

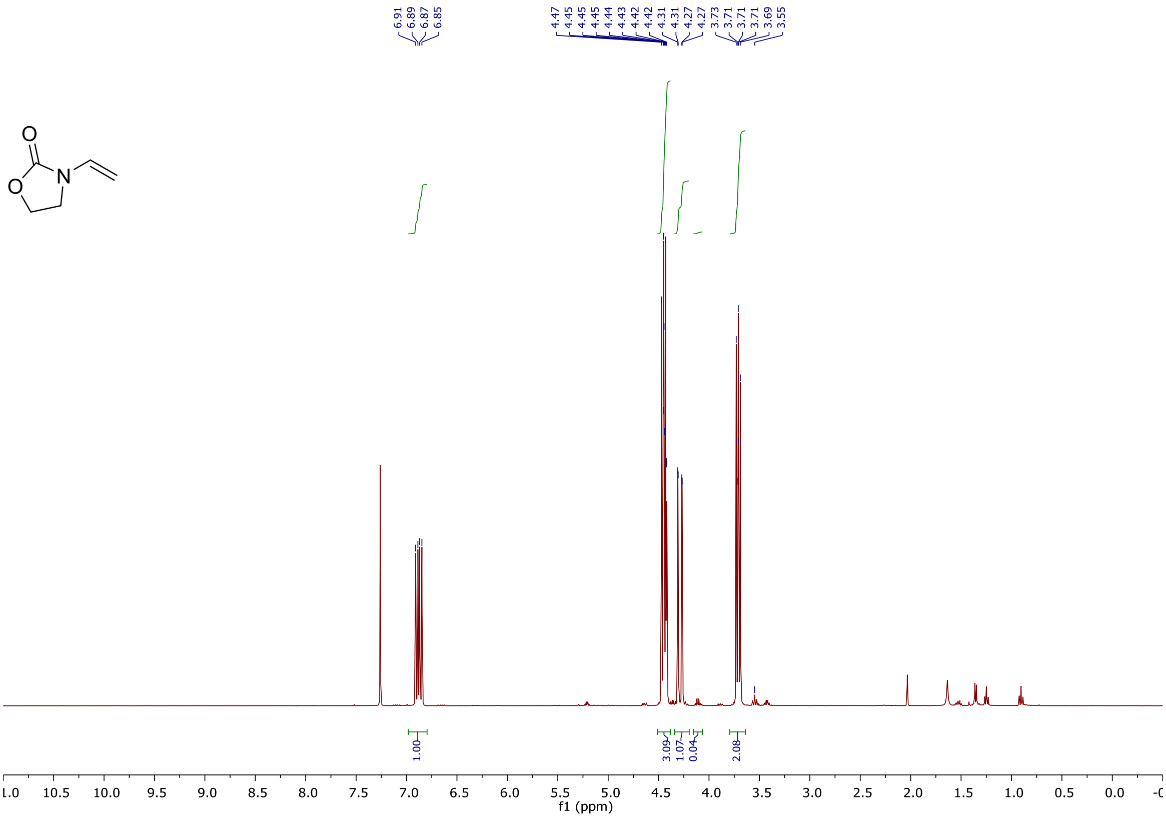

tert-butyl phenethyl(vinyl)carbamate (**1b**)

^{1}H NMR (400 MHz, Chloroform-*d*)

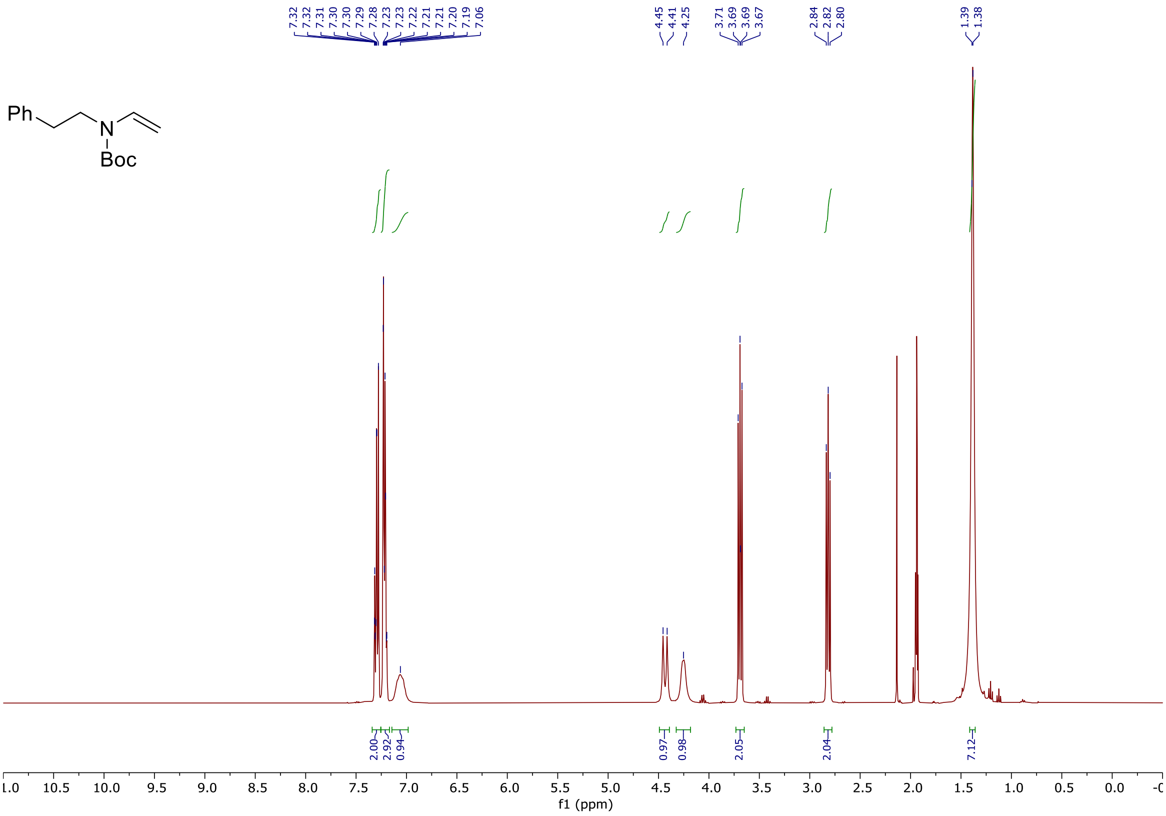

^{13}C NMR (101 MHz, Chloroform-*d*)

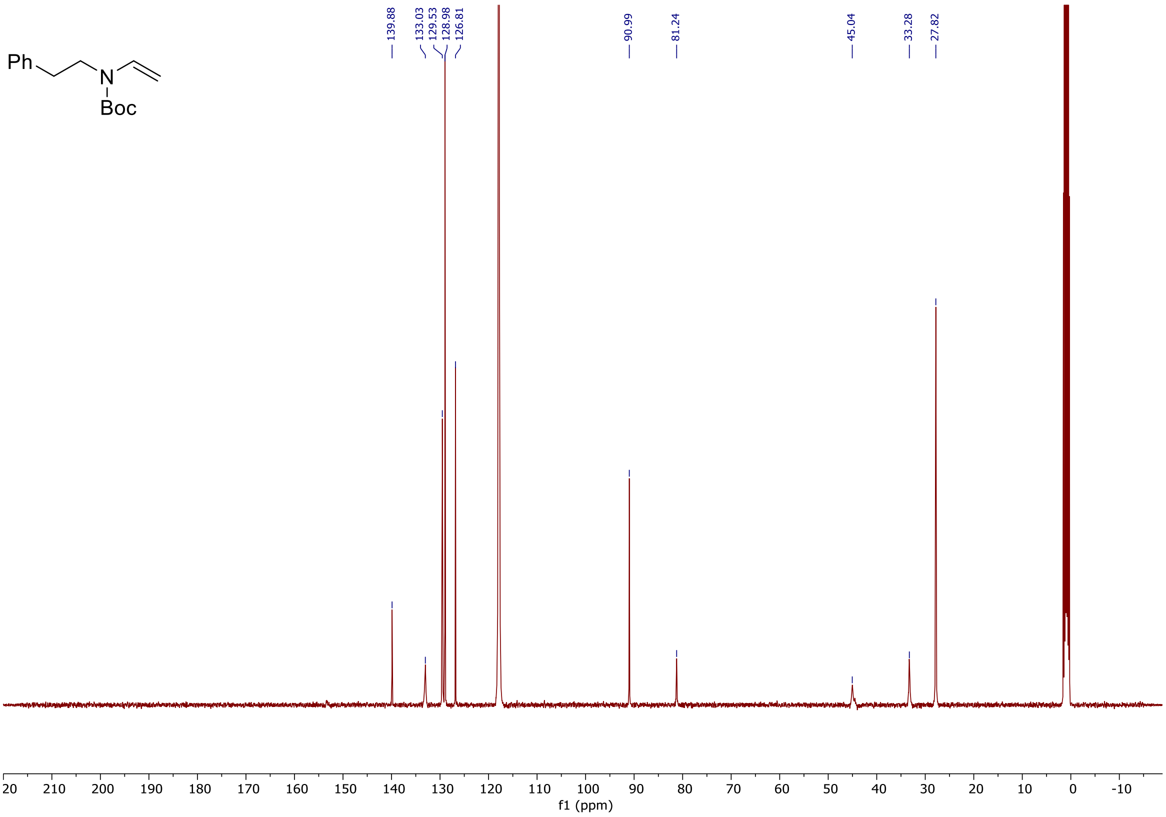

Benzyl phenethyl(vinyl)carbamate (**1c**)

^{1}H NMR (400 MHz, Acetonitrile-d_3)

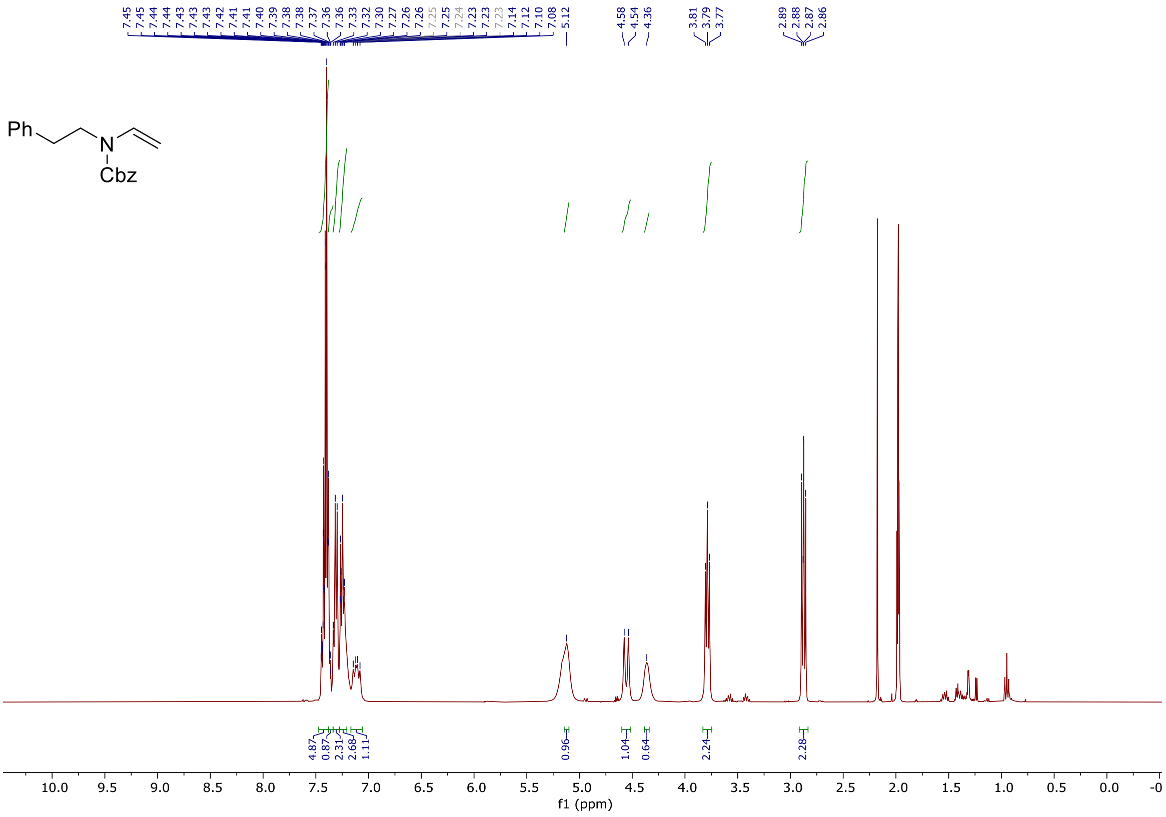

^{13}C NMR (101 MHz, Acetonitrile-d_3)

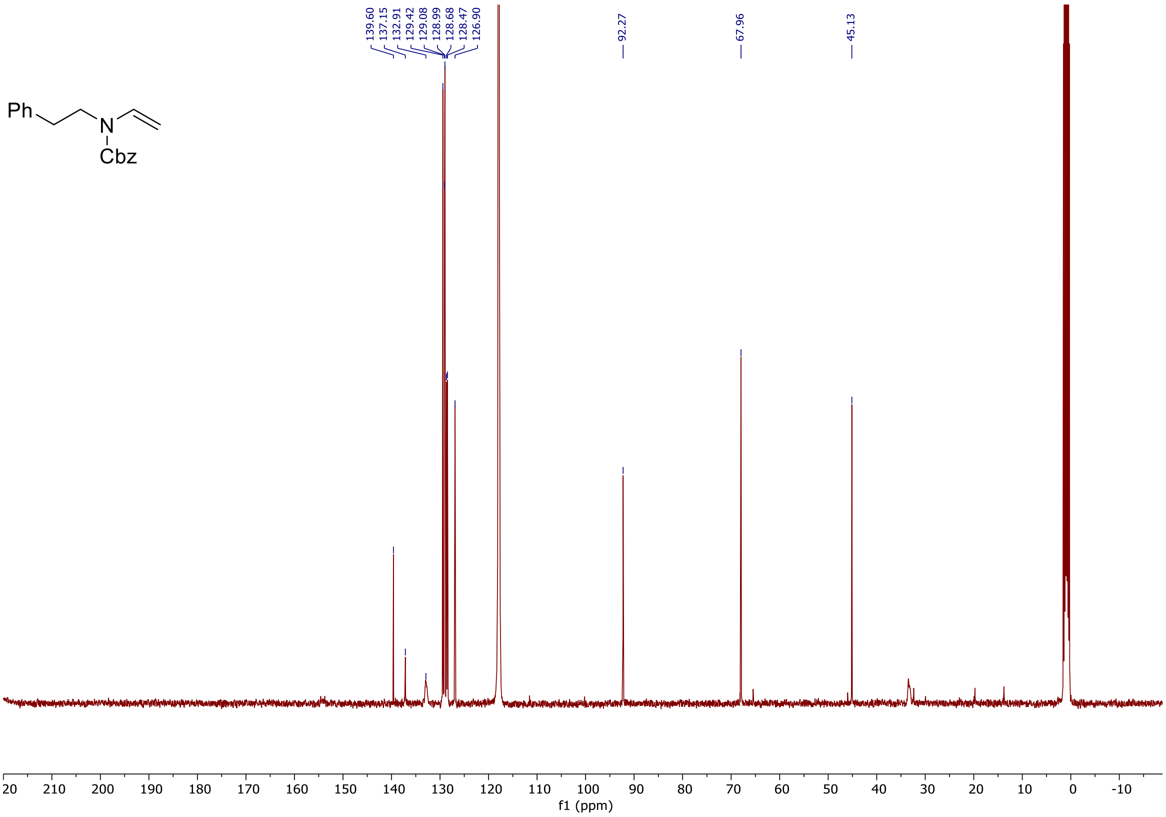

tert-butyl benzyl(vinyl)carbamate (**1d**)

^{1}H NMR (400 MHz, DMSO-d_6)

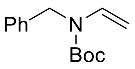

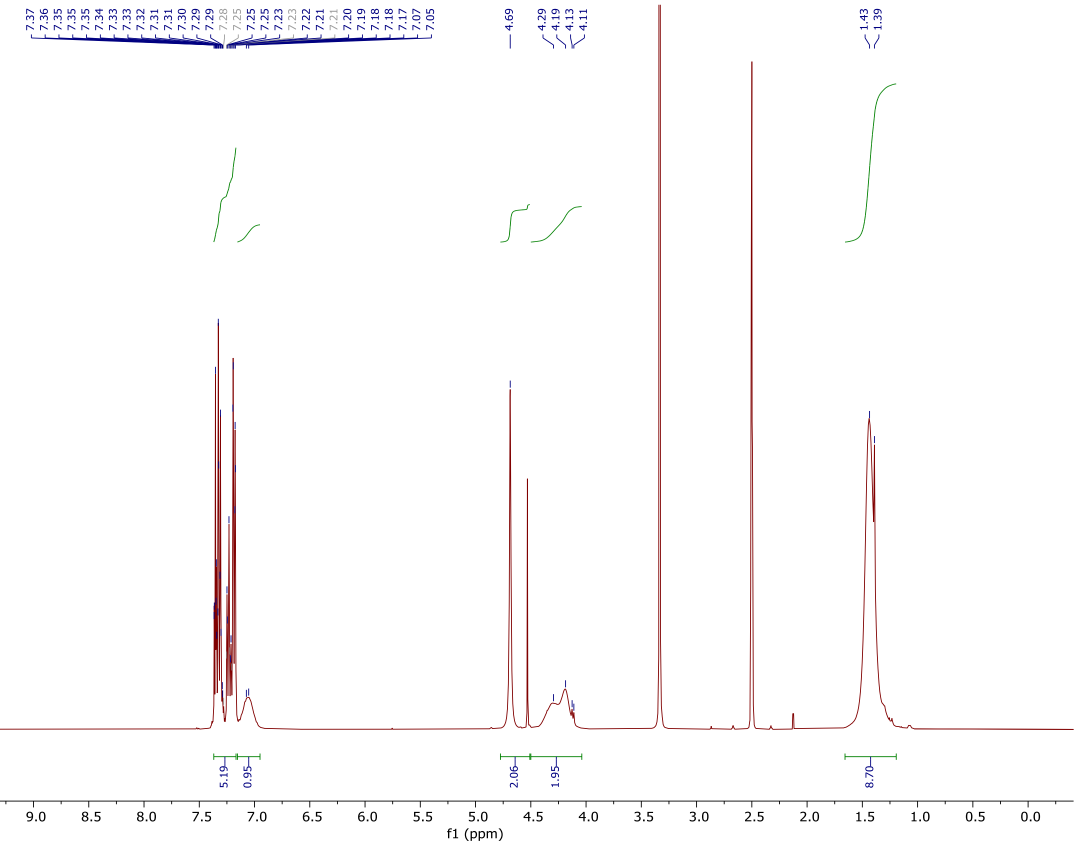

tert-butyl methyl(vinyl)carbamate (**1e**)

1H NMR (400 MHz, Acetonitrile-d_3)

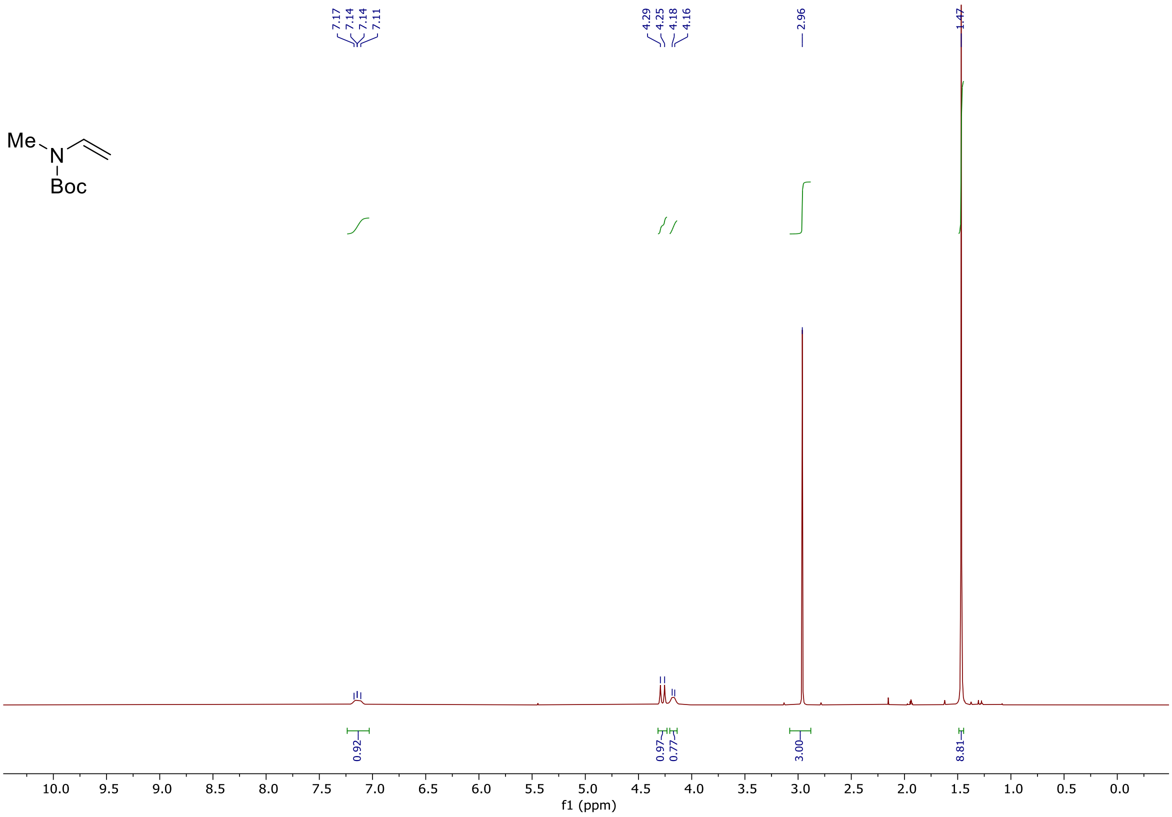

^{13}C NMR (101 MHz, Acetonitrile-d_3)

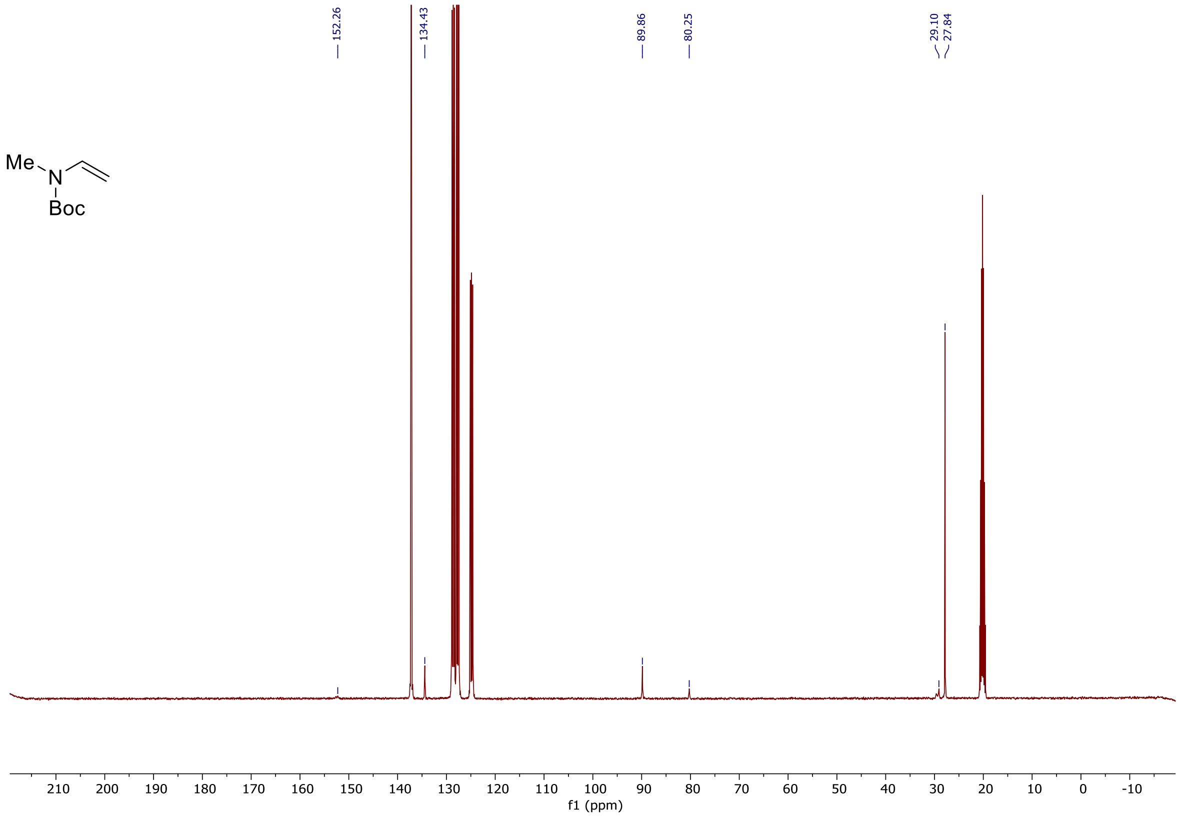

tert-Butyl allyl(vinyl)carbamate (**1f**)

^{1}H NMR (400 MHz, Chloroform-*d*)

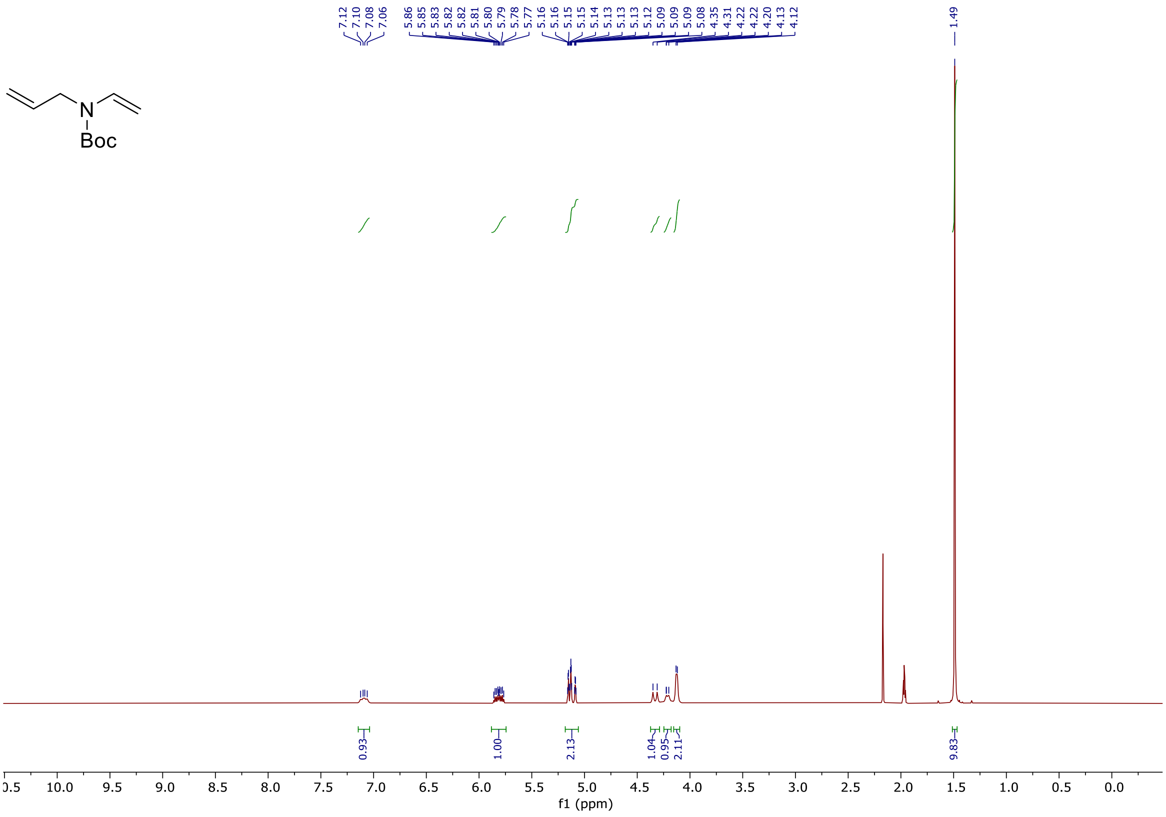

^{13}C NMR (101 MHz, Chloroform-*d*)

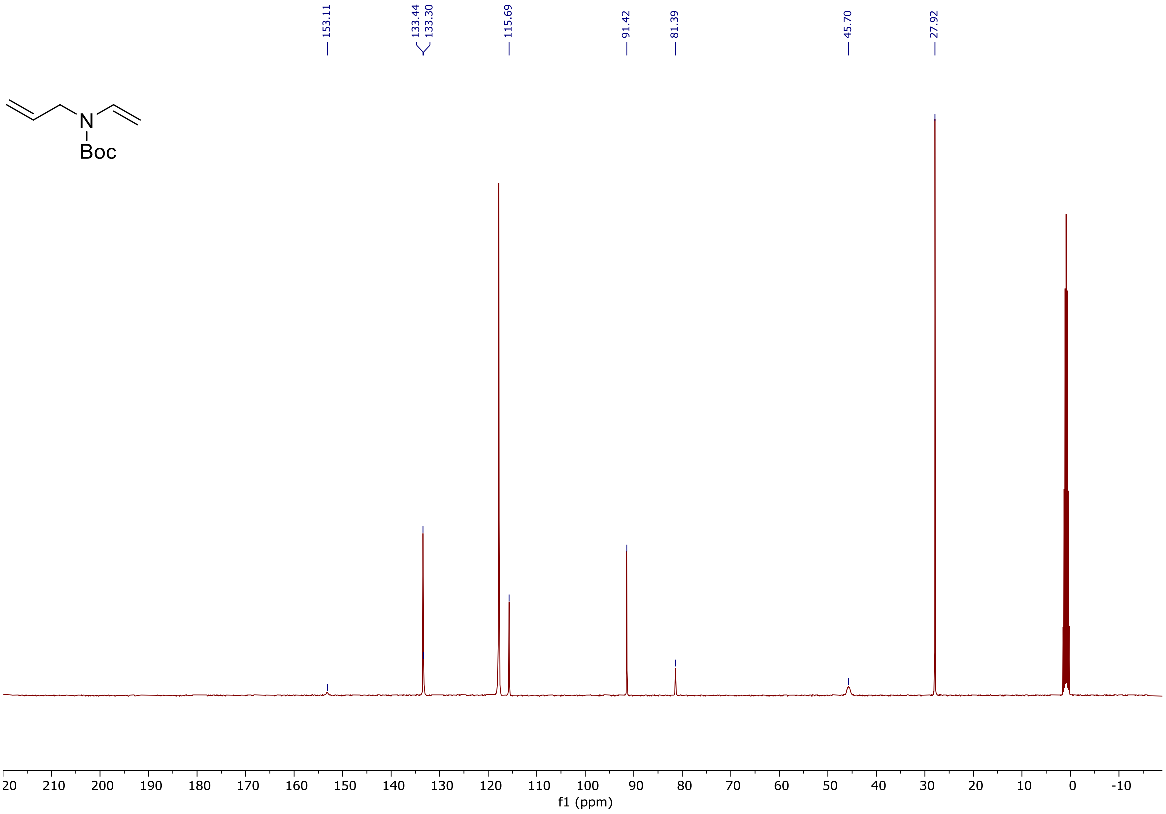

tert-Butyl (2-*tert*-butyldimethylsilyl)oxy)ethyl)(vinyl)carbamate (**1g**)

^{1}H NMR (400 MHz, Chloroform-*d*)

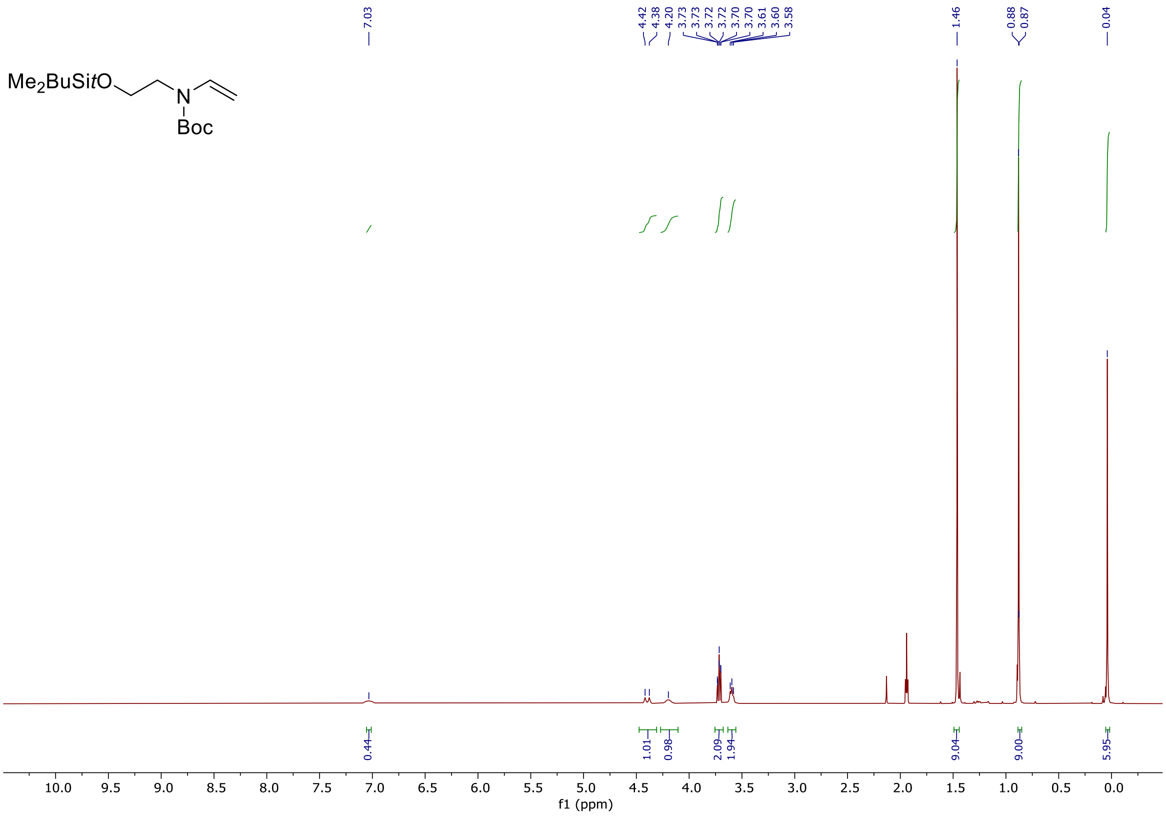

^{13}C NMR (101 MHz, Chloroform-*d*)

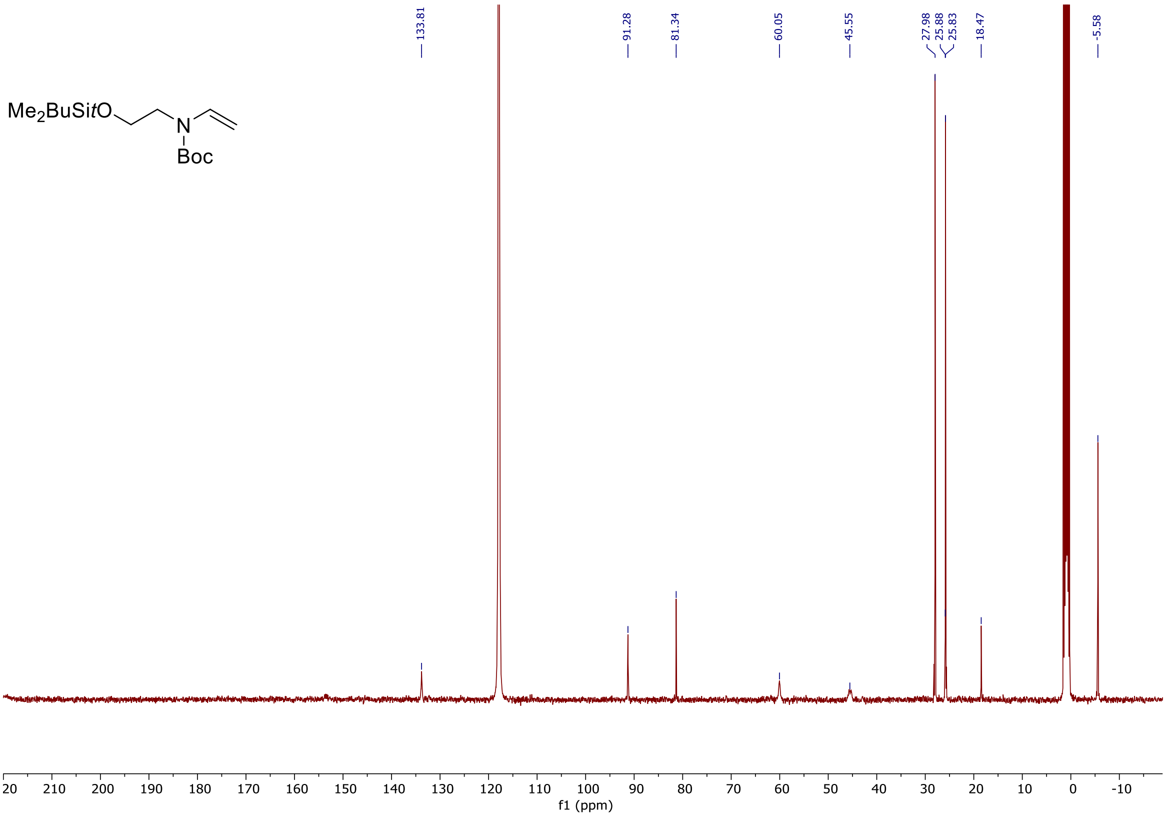

Ethyl 3-((tert-butoxycarbonyl)(vinyl)amino)propanoate (**1h**)

^{1}H NMR (400 MHz, Chloroform-*d*)

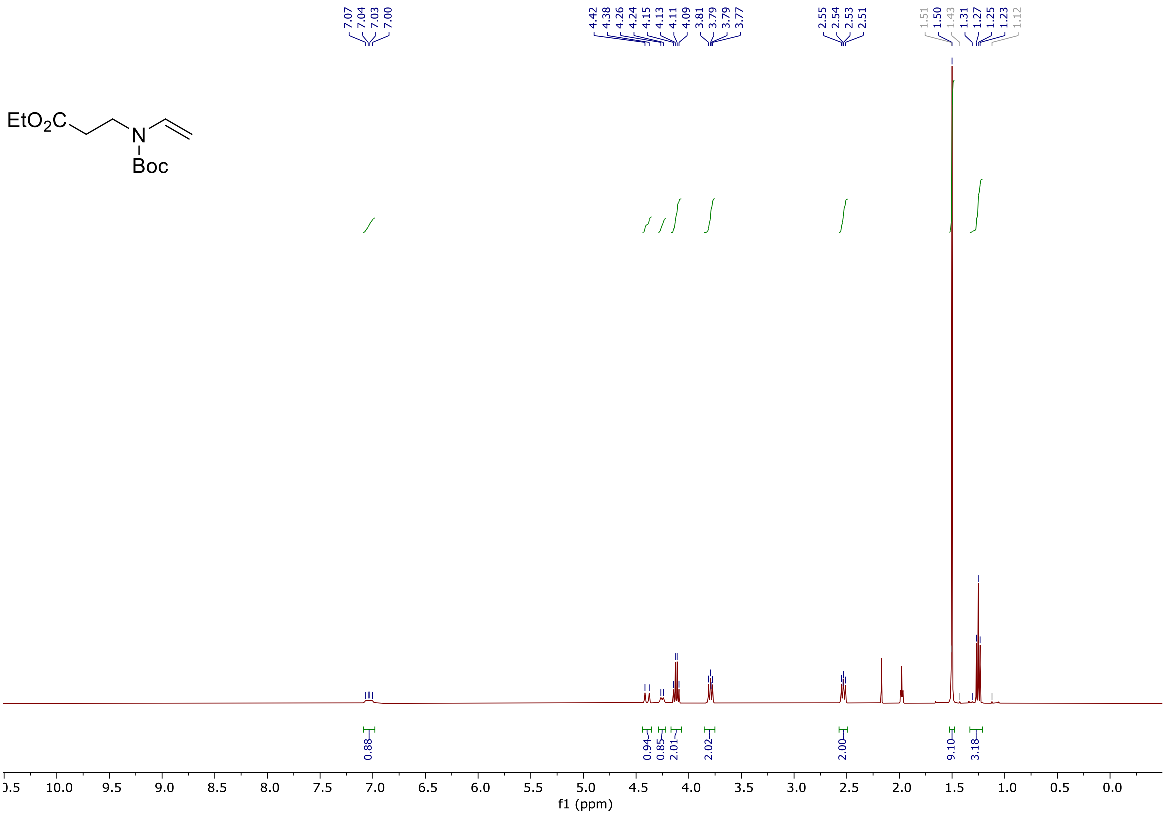

^{13}C NMR (101 MHz, Chloroform-*d*)

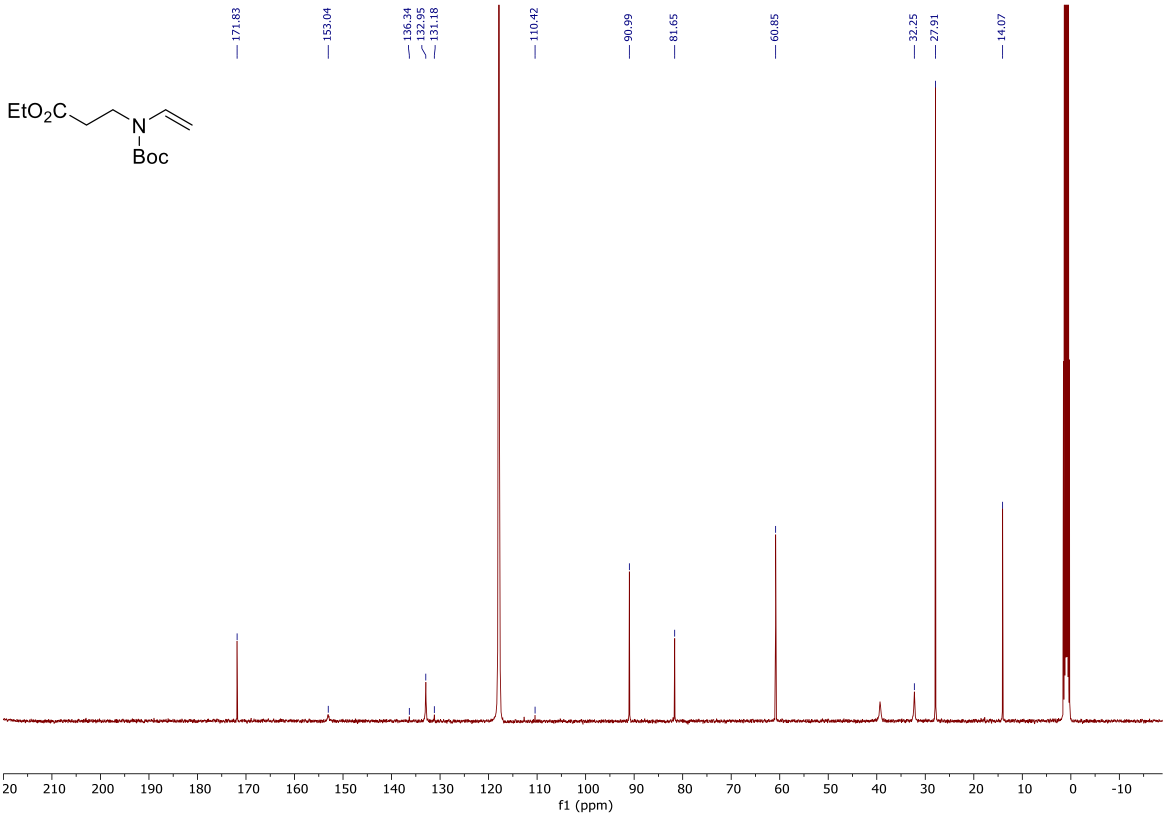

tert-Butyl cyclohexyl(vinyl)carbamate (**1i**)

^{1}H NMR (400 MHz, Chloroform-*d*)

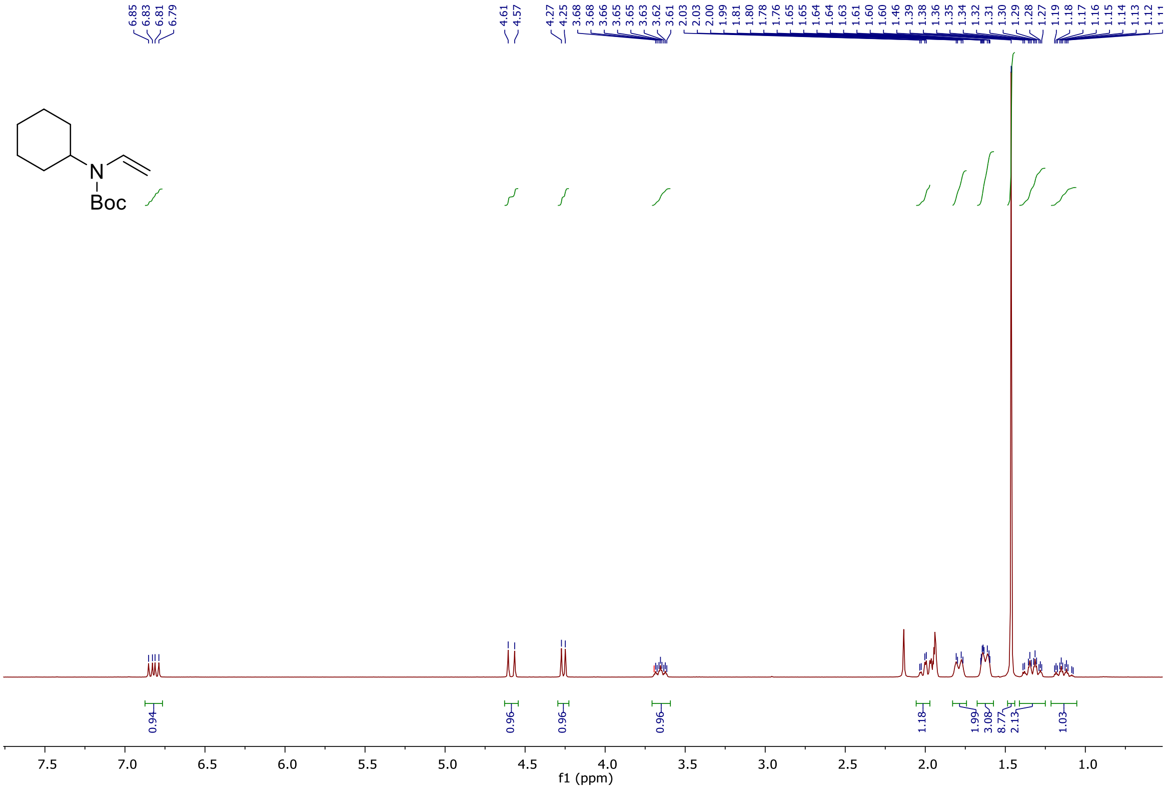

^{13}C NMR (101 MHz, Chloroform-*d*)

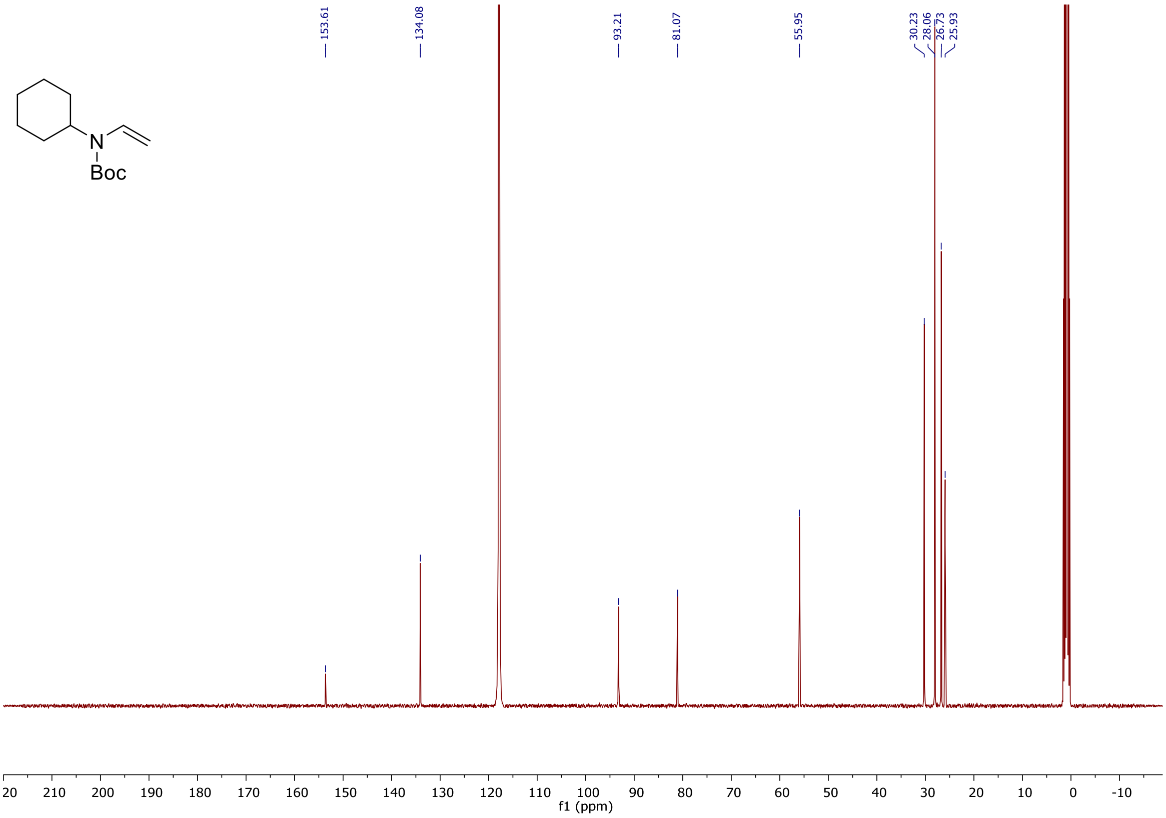

Benzyl phenethyl(propen-2-yl)carbamate (**1k**)

^{1}H NMR (400 MHz, Chloroform-*d*)

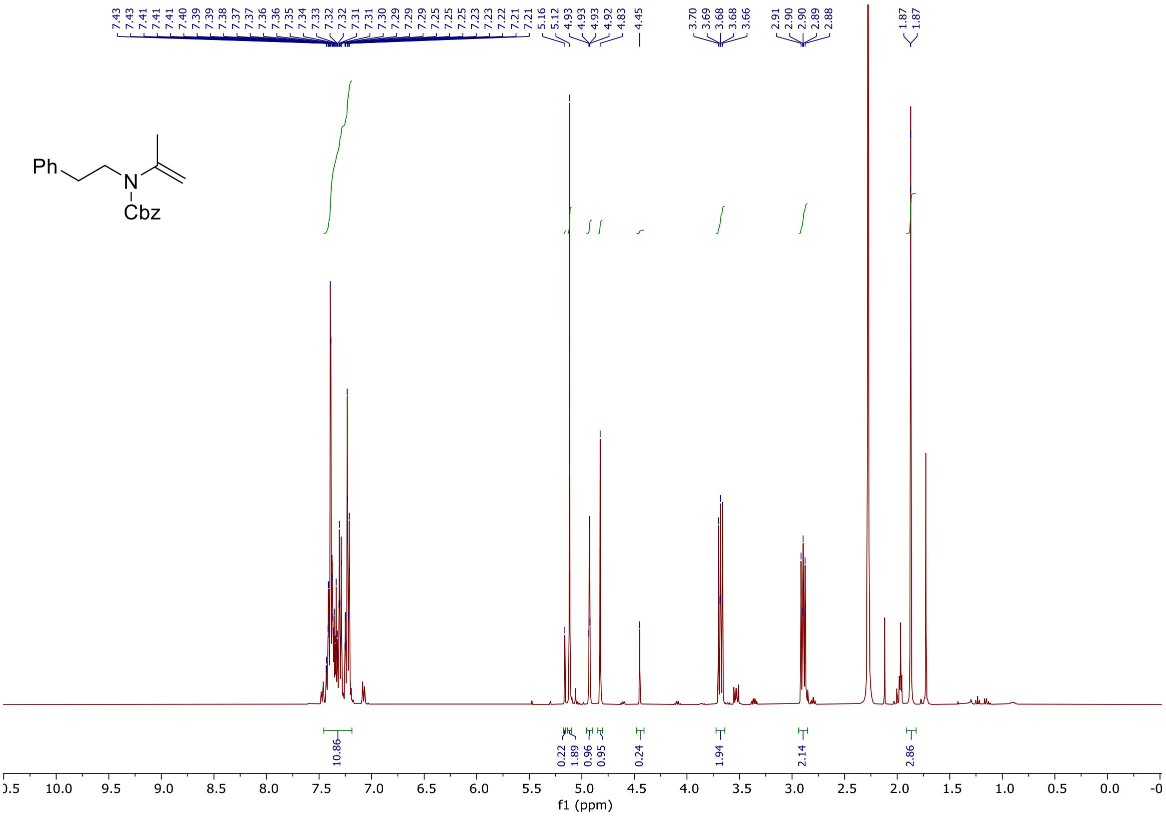

^{13}C NMR (101 MHz, Chloroform-*d*)

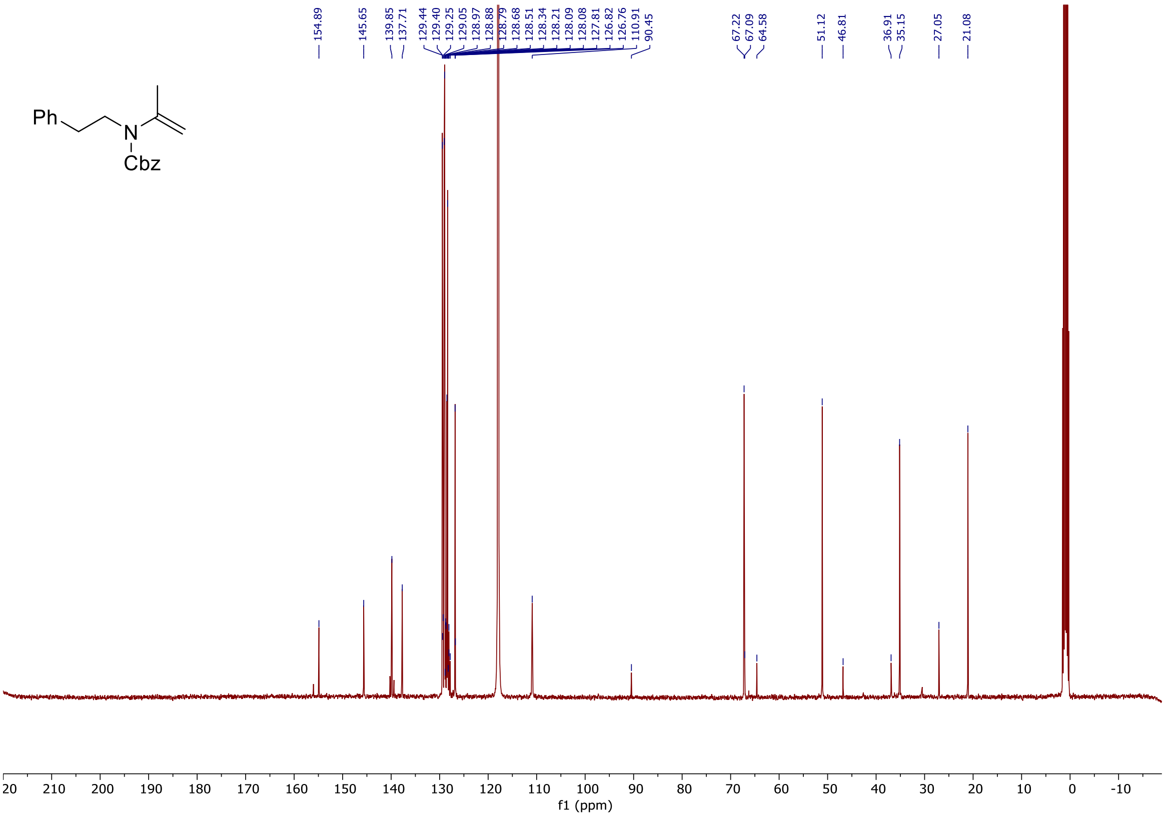

(*E*)-3-(hex-1-en-1-yl)oxazolidine-2-one (**1l**)

^{1}H NMR (Chloroform-*d*)

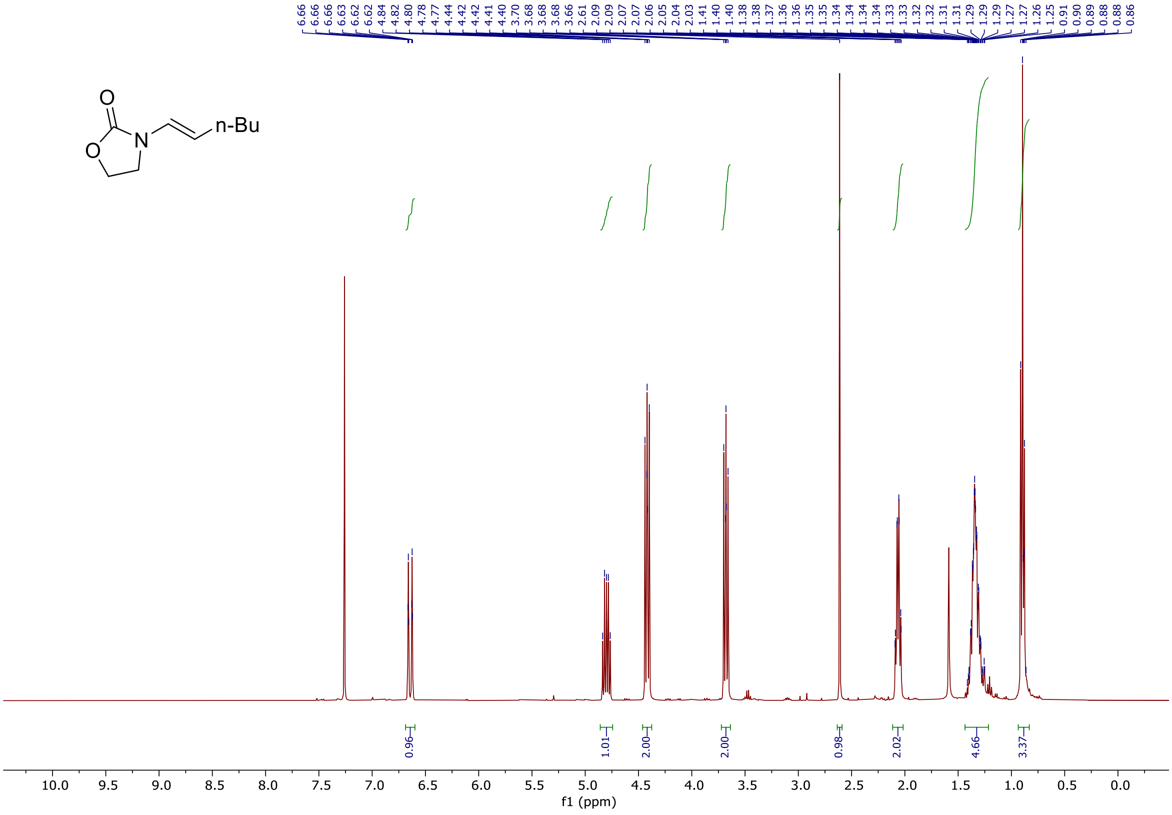

3-(hex-1-en-1-yl)oxazolidine-2-one (*Z*:*E* 2:1 **1l + 1l'**)

^{1}H NMR (400 MHz, Benzene-d_6)

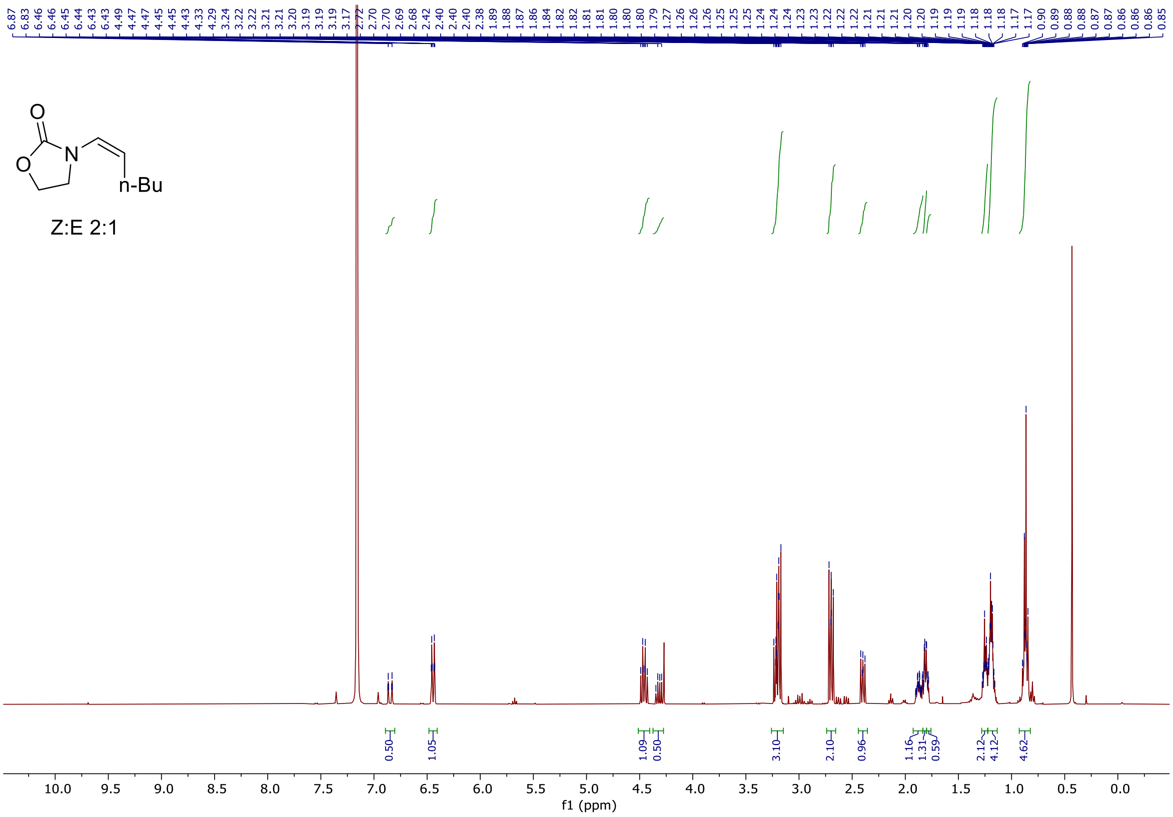

^{13}C NMR (101 MHz, Benzene-d_6)

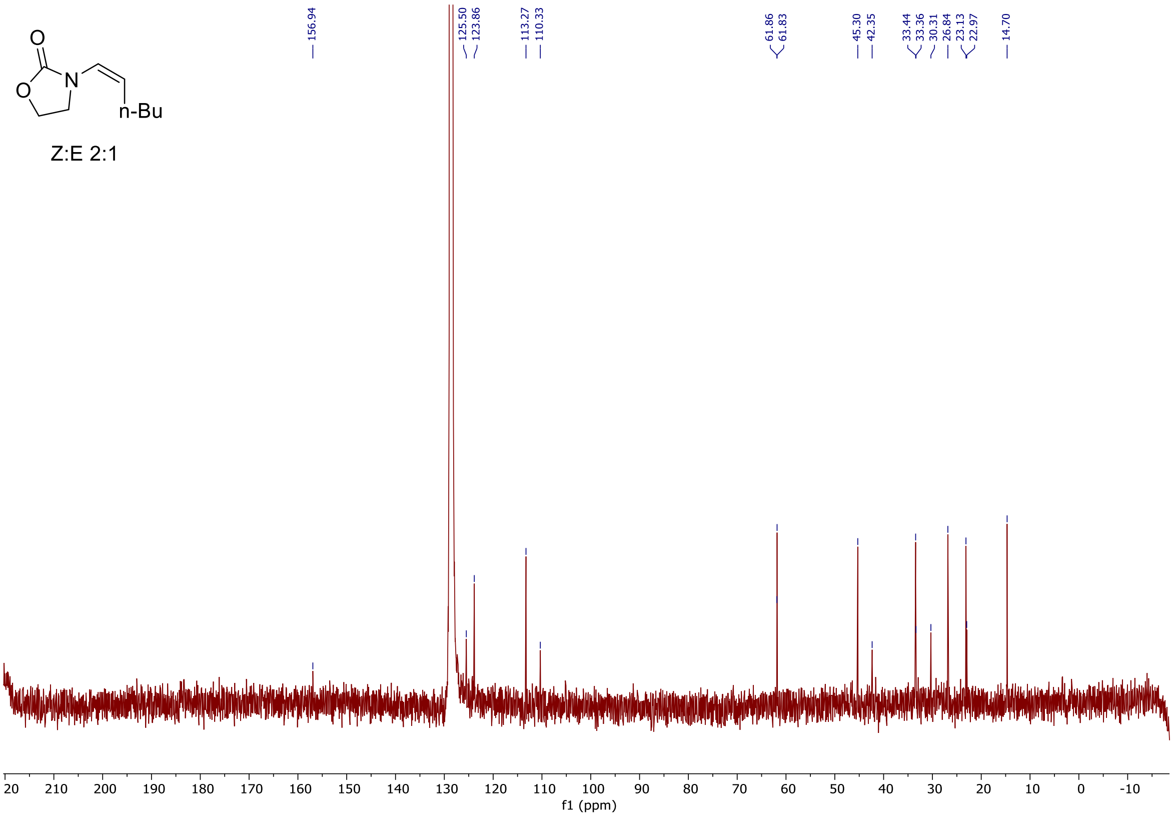

(*E*)-((hex-1-en-1-yloxy)methyl)benzene (**9g**)

^{1}H NMR (400 MHz, Chloroform-*d*)

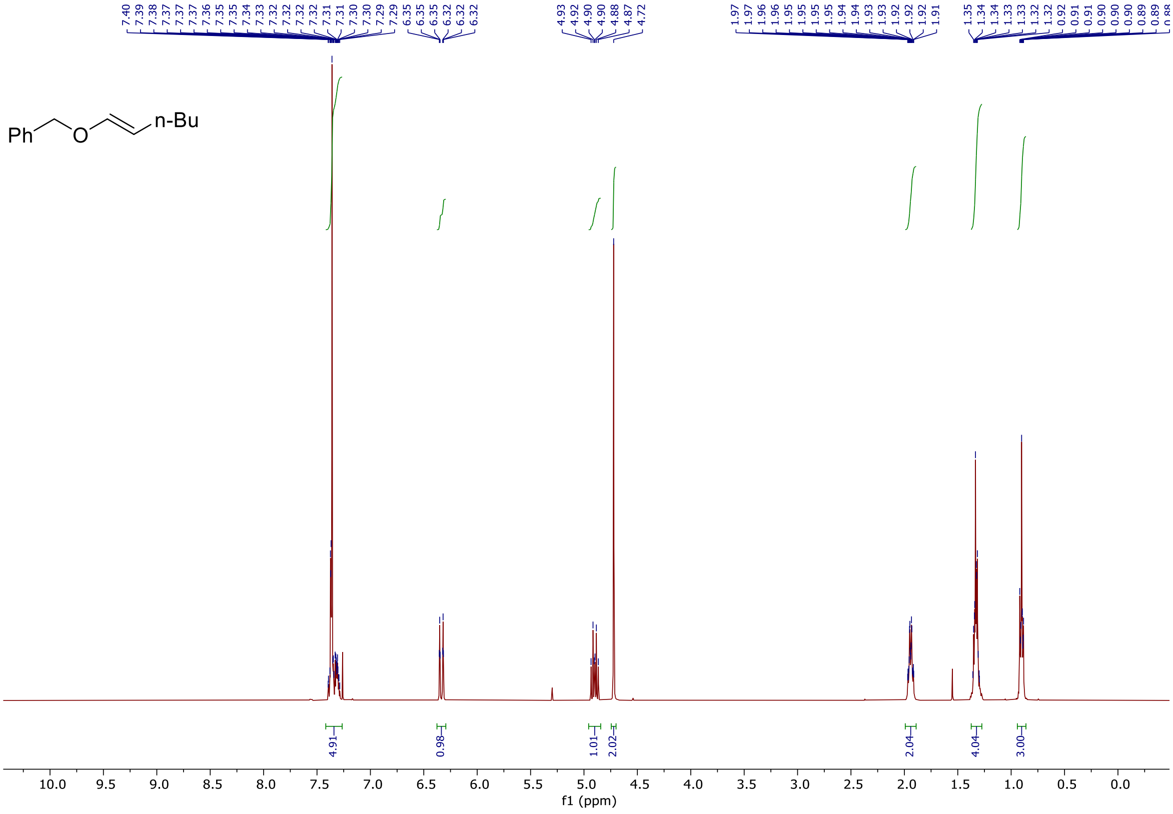

^{13}C NMR (101 MHz, Chloroform-*d*)

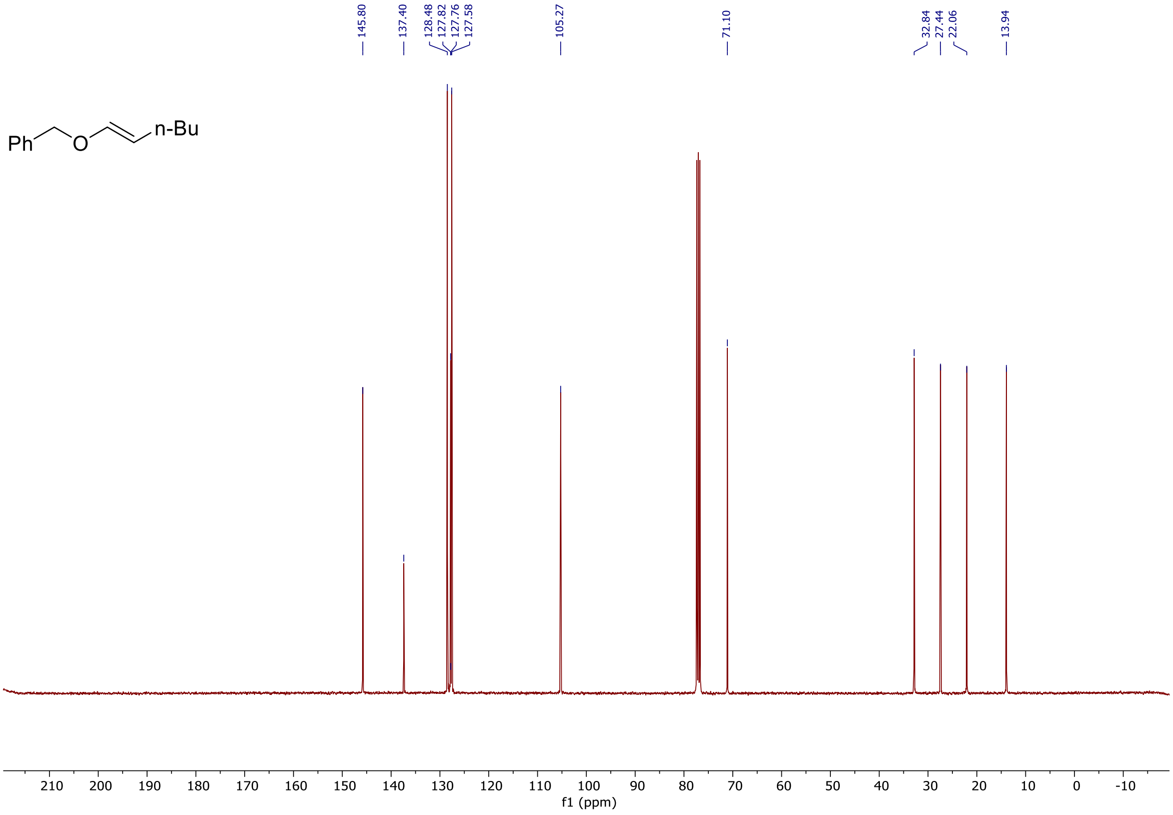

Products

2-(2-oxooxazolidin-3-yl)-4-phenylbut-3-yn-1-yl 2-iodo benzoate (**3a**) (traces of ethyl acetate)

^{1}H NMR (400 MHz, Chloroform-*d*)

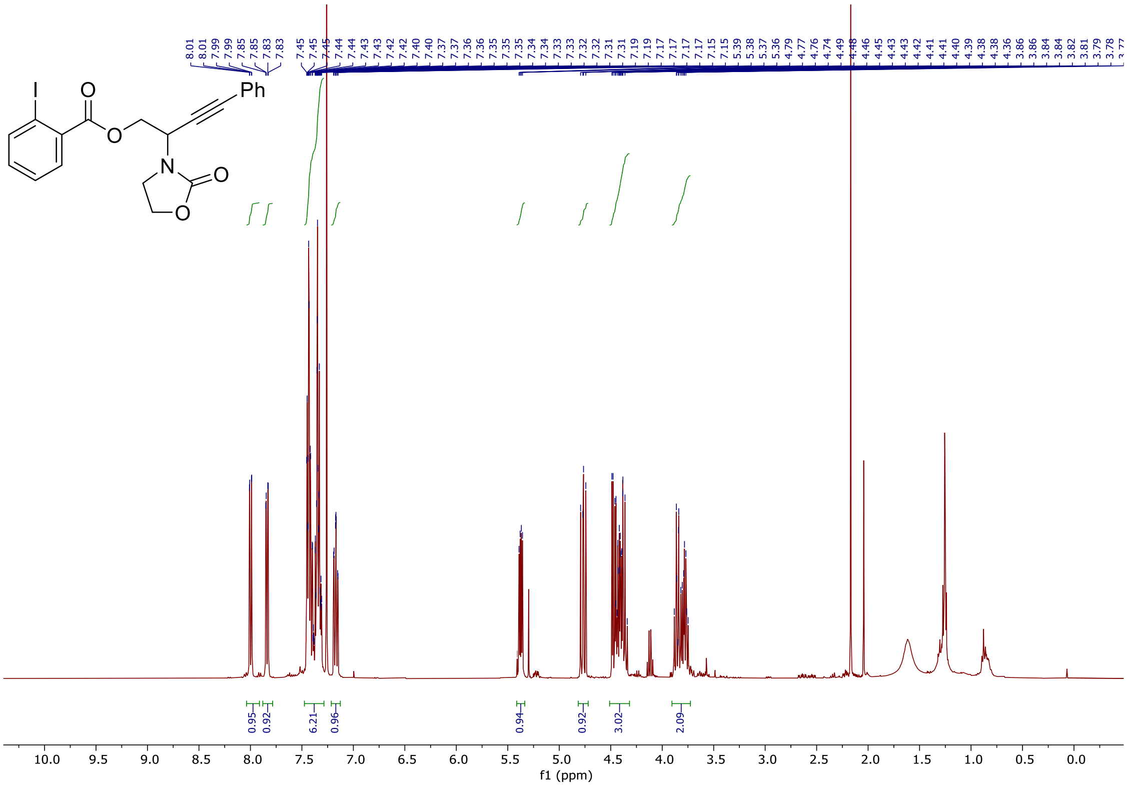

^{13}C NMR (101 MHz, Chloroform-*d*)

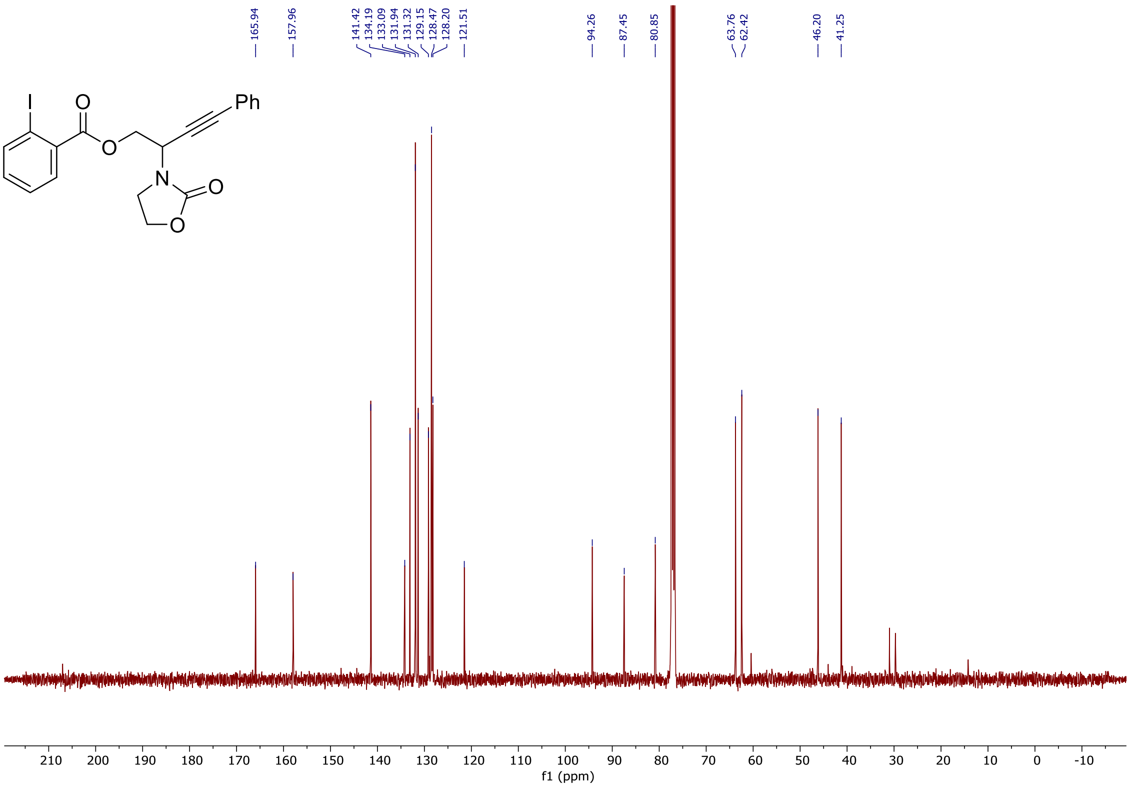

2-((*tert*-butoxycarbonyl)(phenethyl)amino)-4-phenylbut-3-yn-1-yl 2-iodobenzoate (**3b**)

^{1}H NMR (400 MHz, Acetonitrile-d_3)

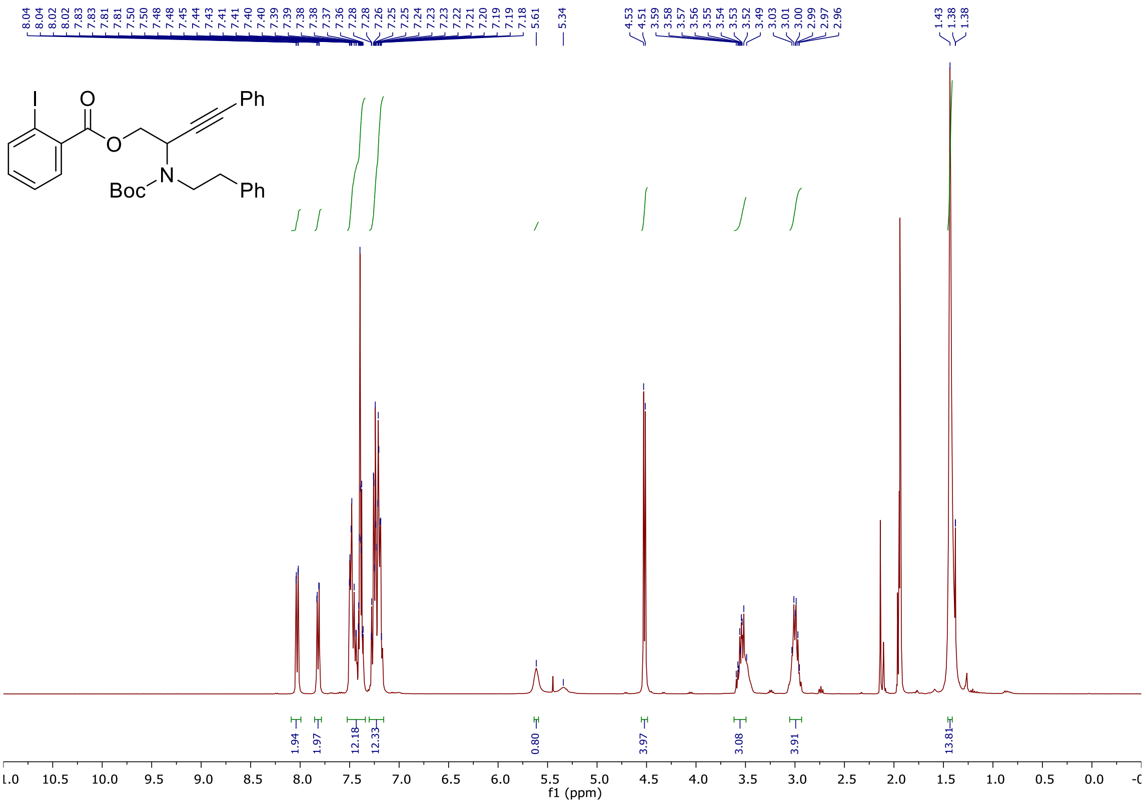

^{13}C NMR (101 MHz, Acetonitrile-d_3)

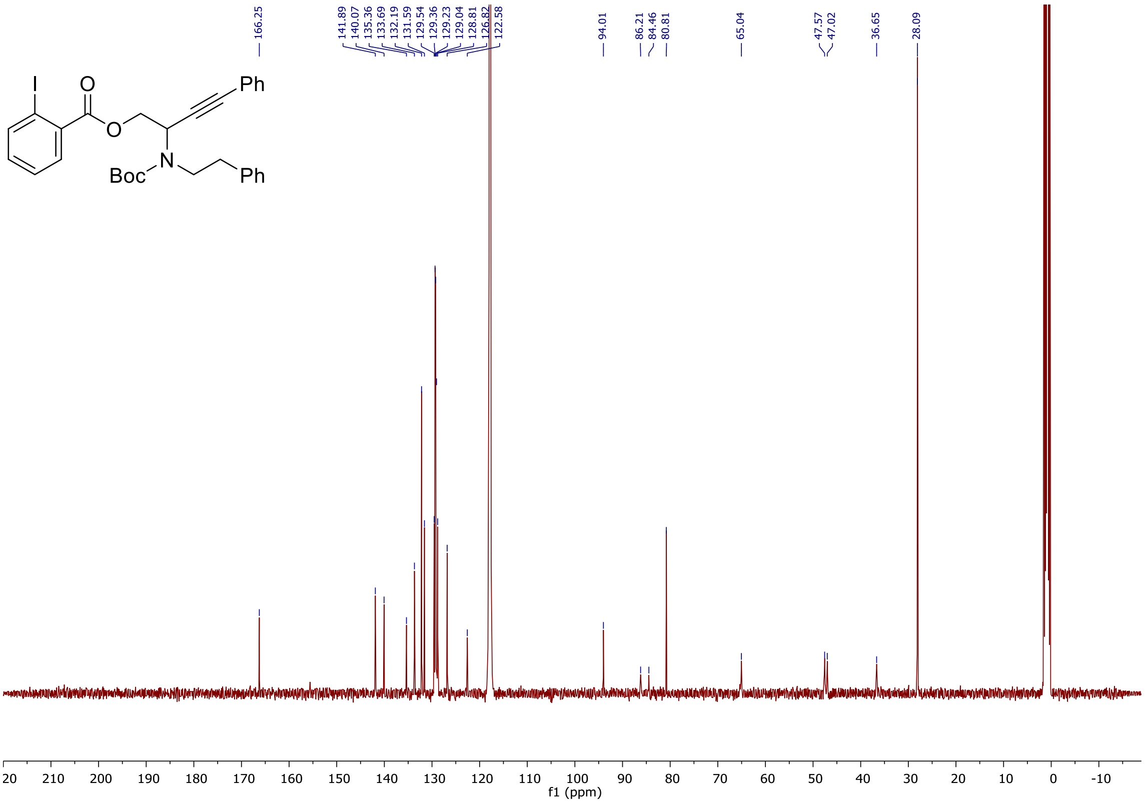

2-(((benzyloxy)carbonyl)(phenethyl)amino)-4-phenylbut-3-yn-1-yl 2-iodobenzoate (**3c**)

^{1}H NMR (400 MHz, Acetonitrile-*d*$_3$)

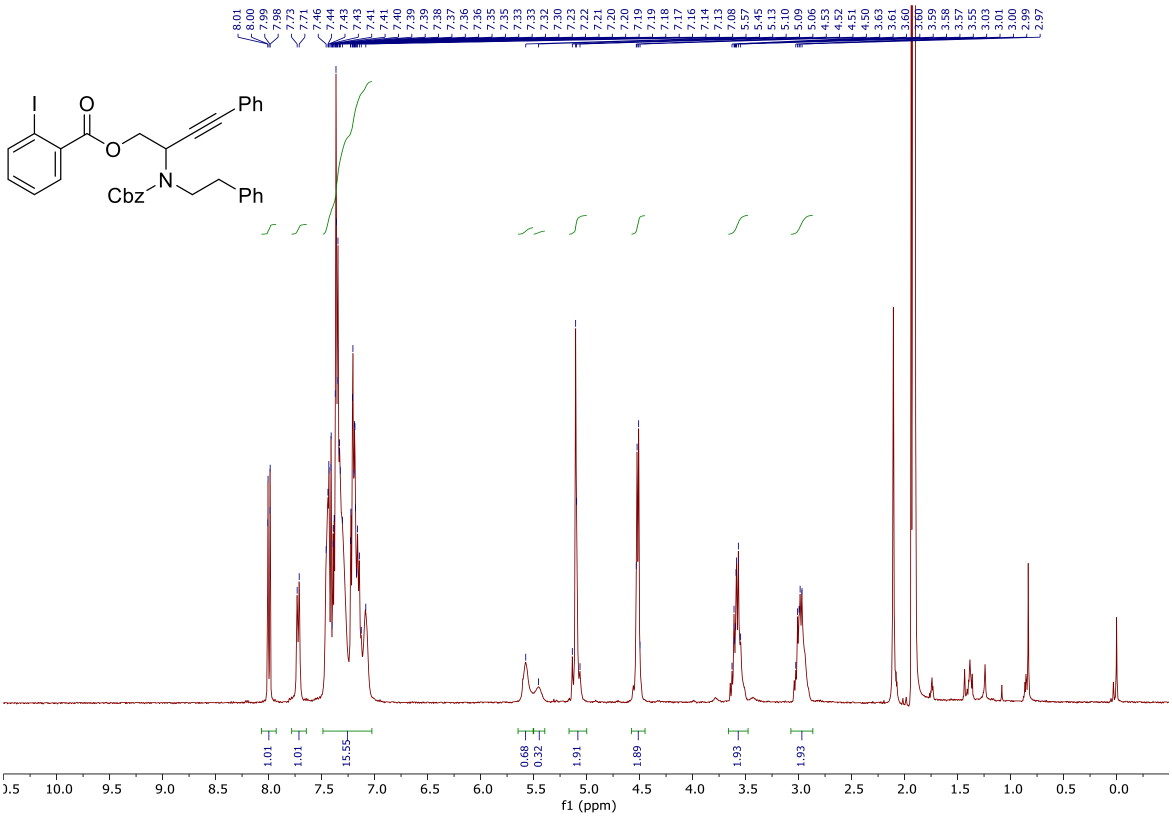

^{13}C NMR (101 MHz, Acetonitrile-*d*$_3$)

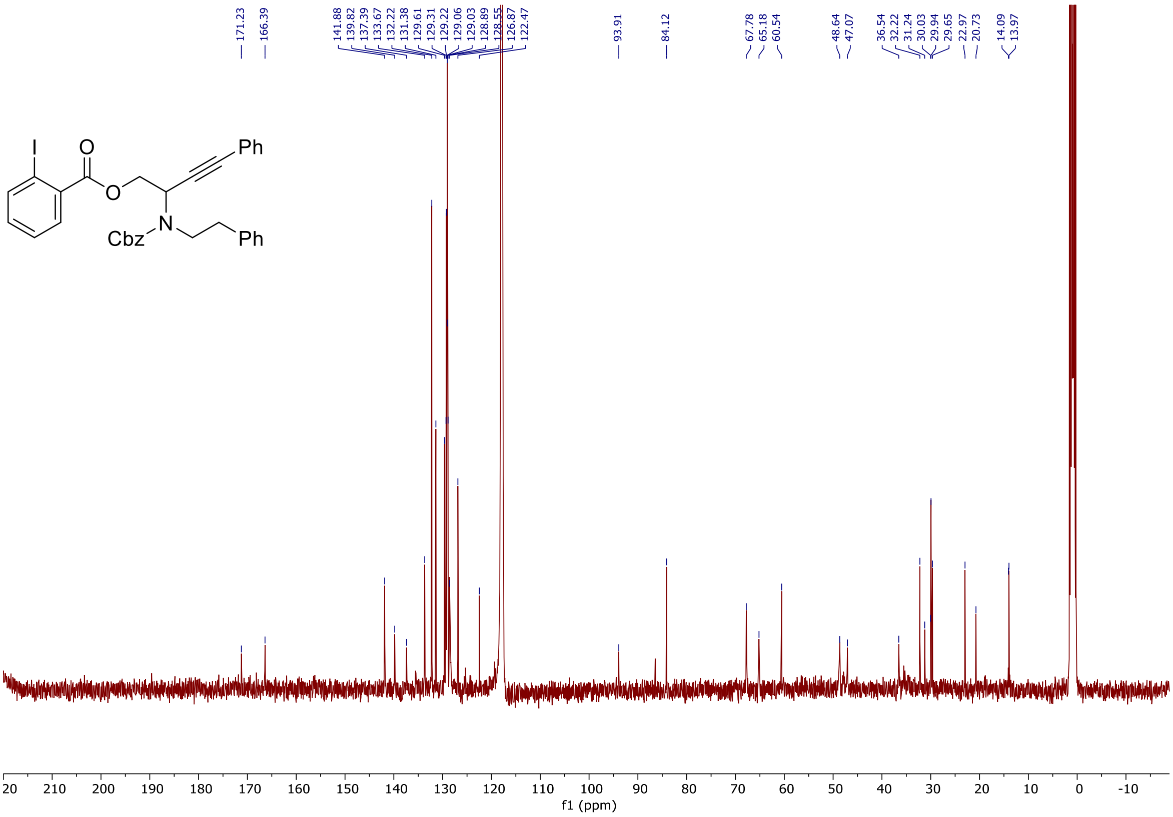

2-(benzyl(*tert*-butoxycarbonyl)amino)-4-phenylbut-3-yn-1-yl 2-iodobenzoate (**3d**)

^1H NMR (400 MHz, Acetonitrile-d_3)

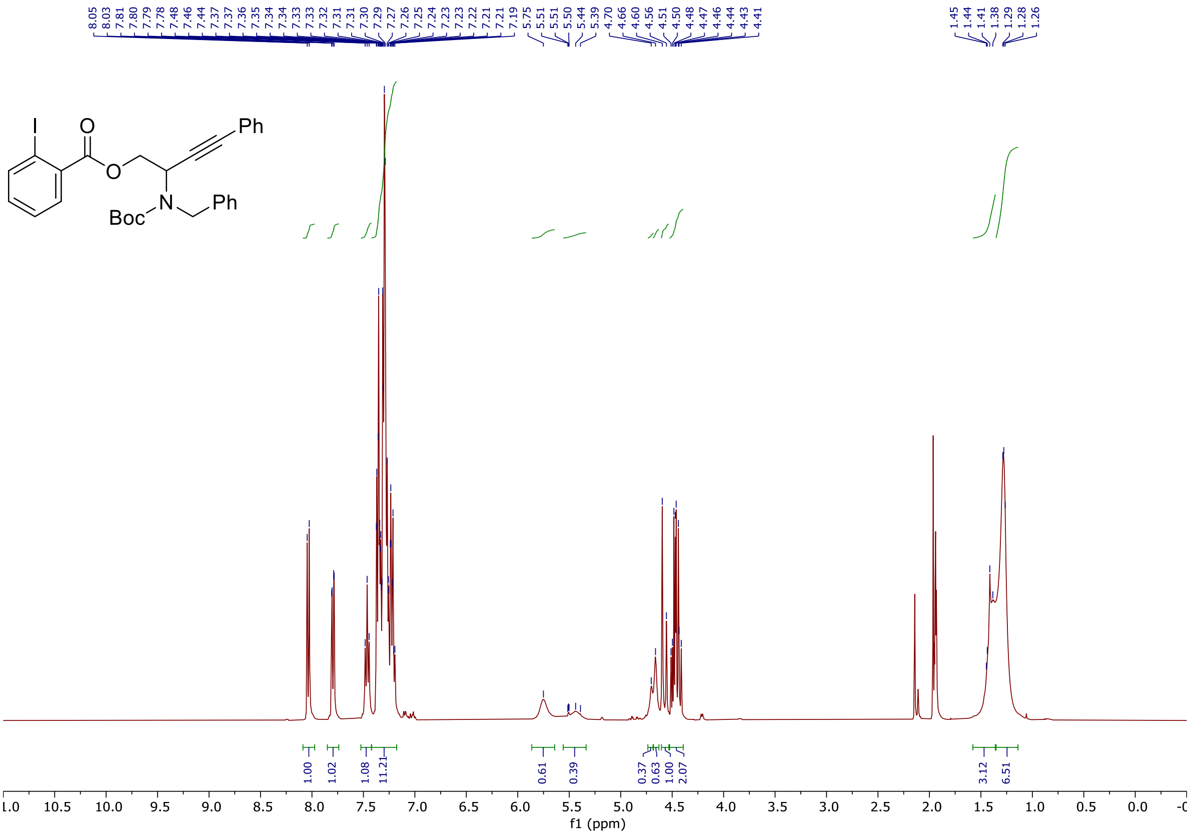

^{13}C NMR (101 MHz, Acetonitrile-d_3)

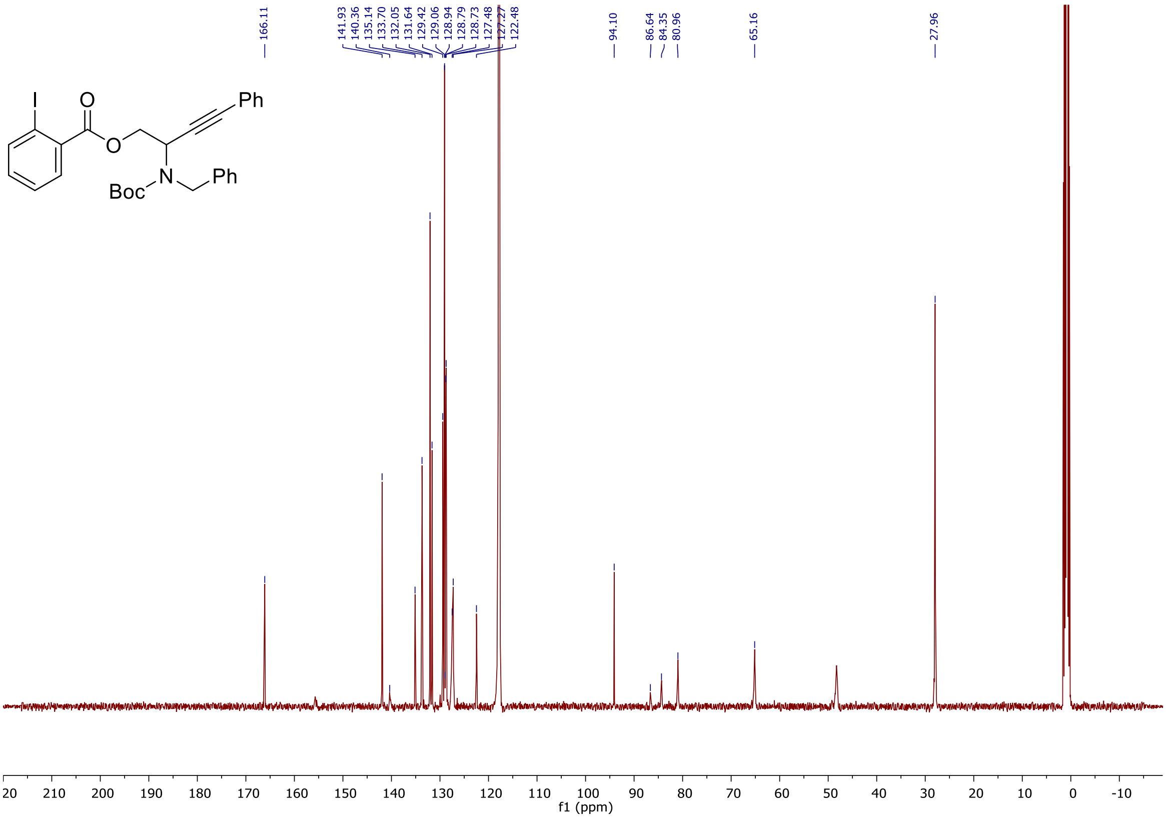

2-(methyl(*tert*-butoxycarbonyl)amino)-4-phenylbut-3-yn-1-yl 2-iodobenzoate (**3e**)

^{1}H NMR (400 MHz, Acetonitrile-*d*$_3$)

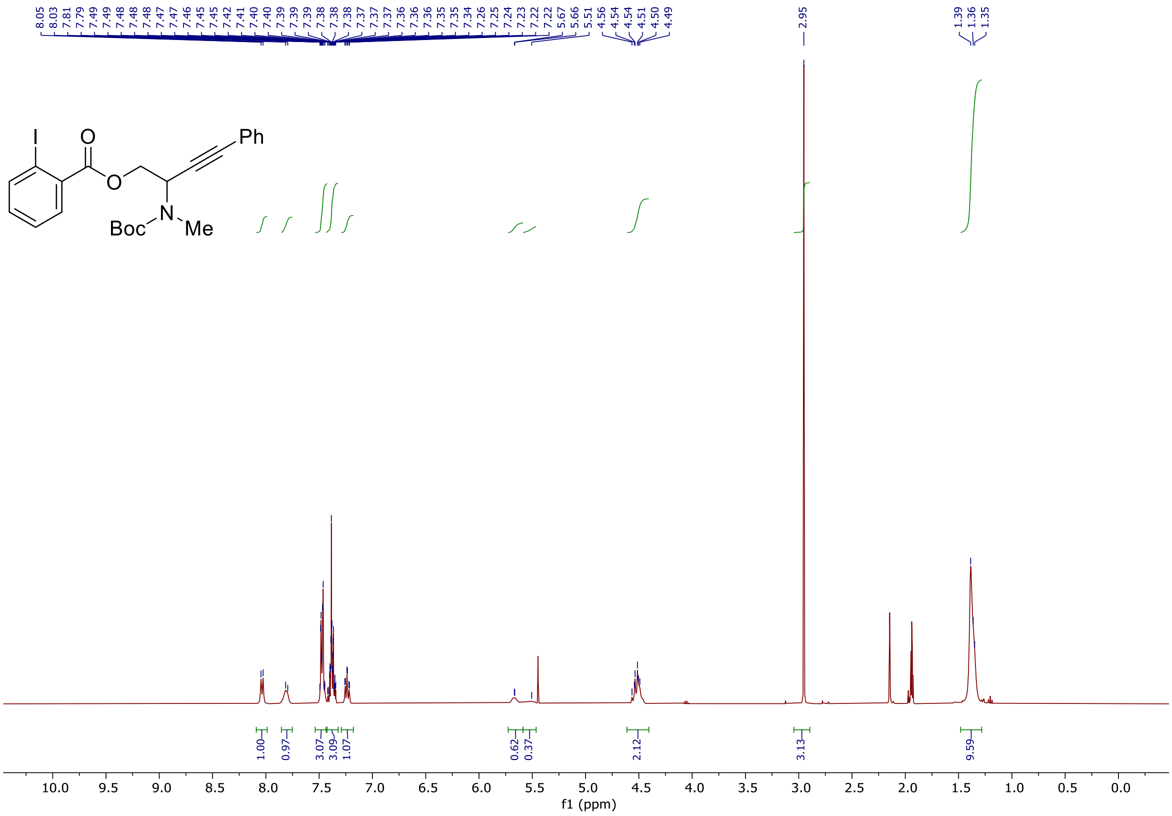

^{13}C NMR (101 MHz, Acetonitrile-*d*$_3$)

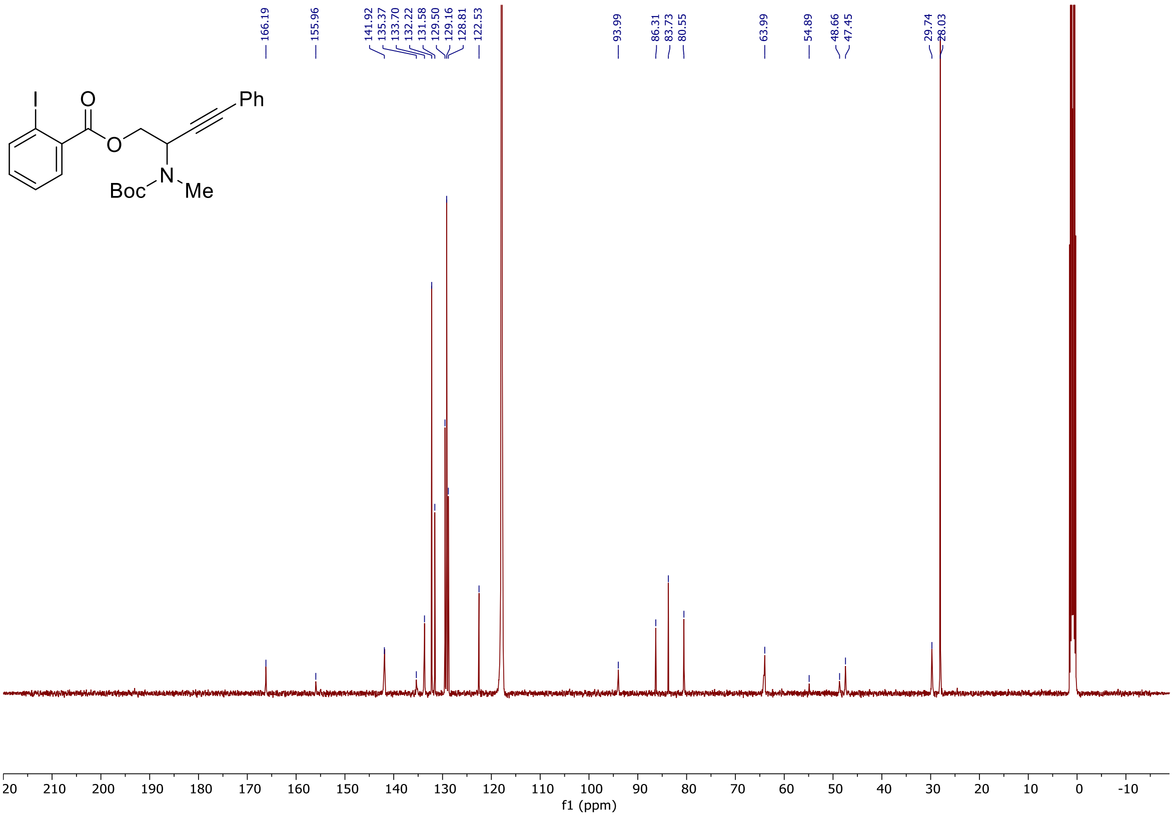

2-(allyl(*tert*-butoxycarbonyl)amino)-4-phenylbut-3-yn-1-yl 2-iodobenzoate (**3f**)

^{1}H NMR (400 MHz, Acetonitrile-d_3)

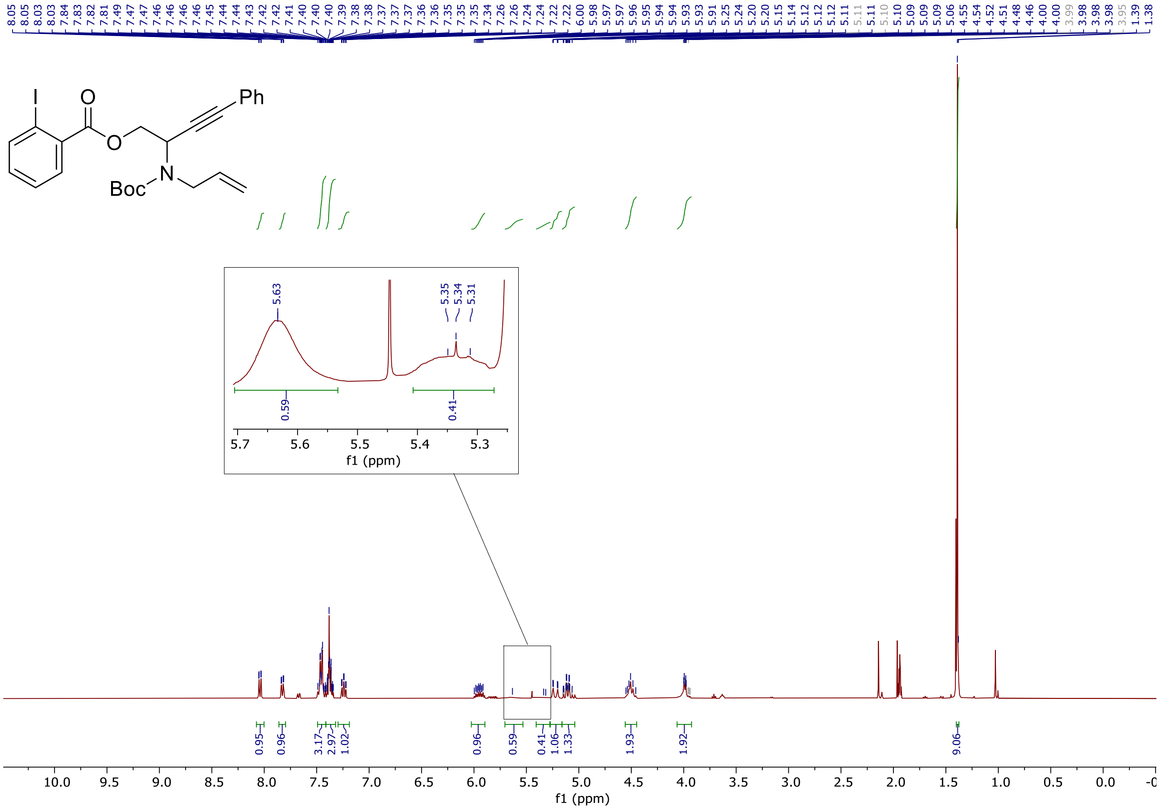

^{13}C (101 MHz, Acetonitrile-d_3)

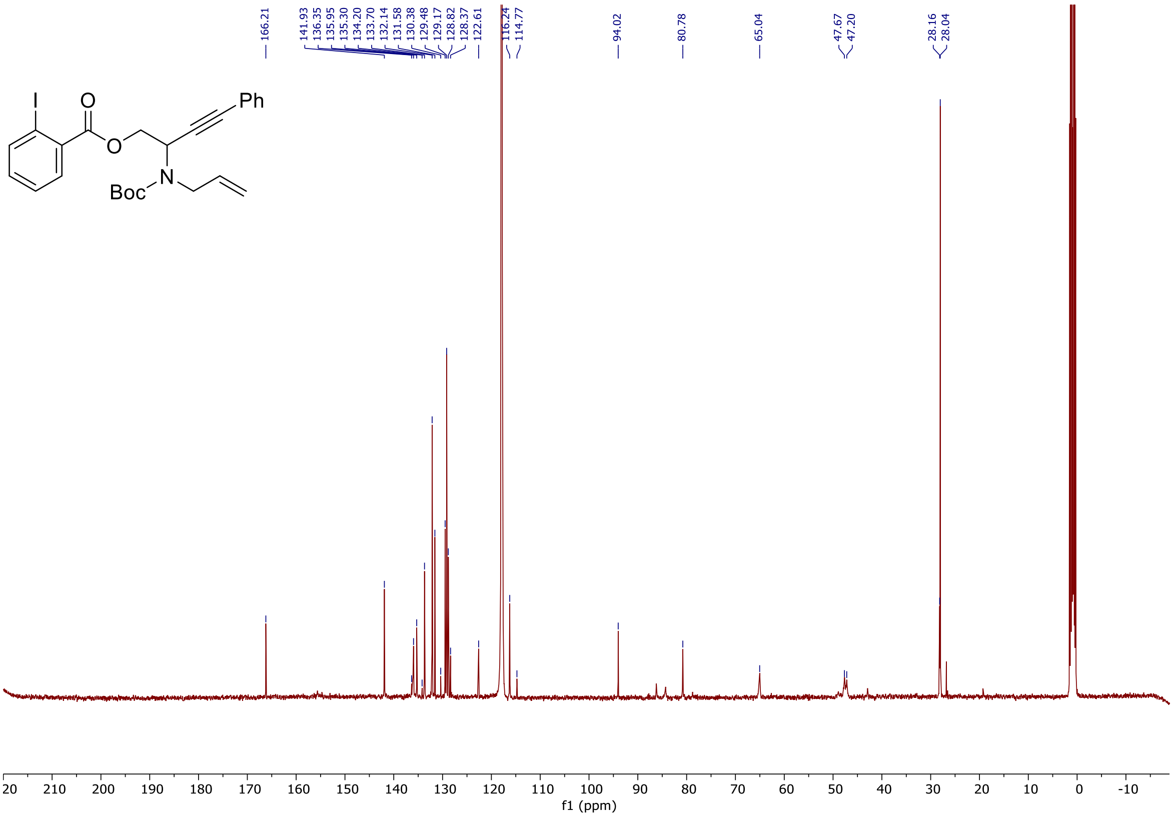

2-((tert-butoxycarbonyl)(2-((tert-butyldimethylsilyl)oxy)ethyl)amino)-4-phenylbut-3-yn-1-yl 2-iodobenzoate (**3g**)

^{1}H NMR (400 MHz, Acetonitrile-d_3)

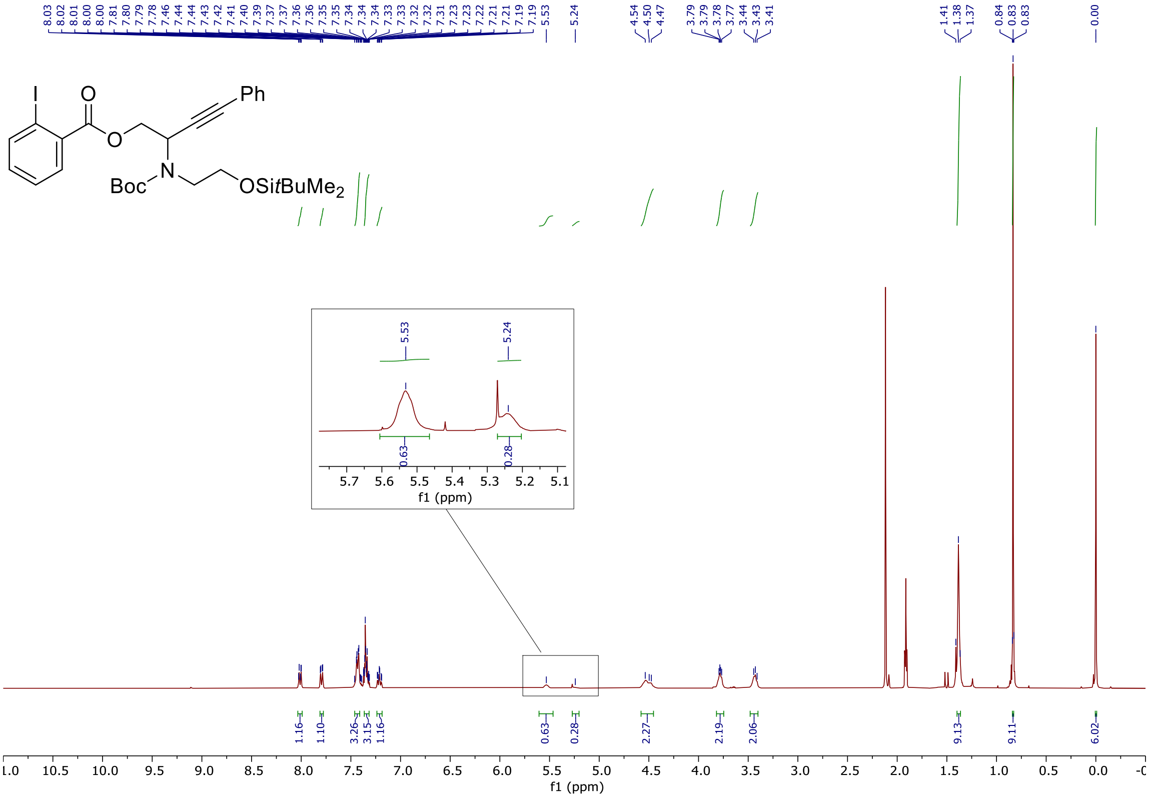

^{13}C NMR (101 MHz, Acetonitrile-d_3)

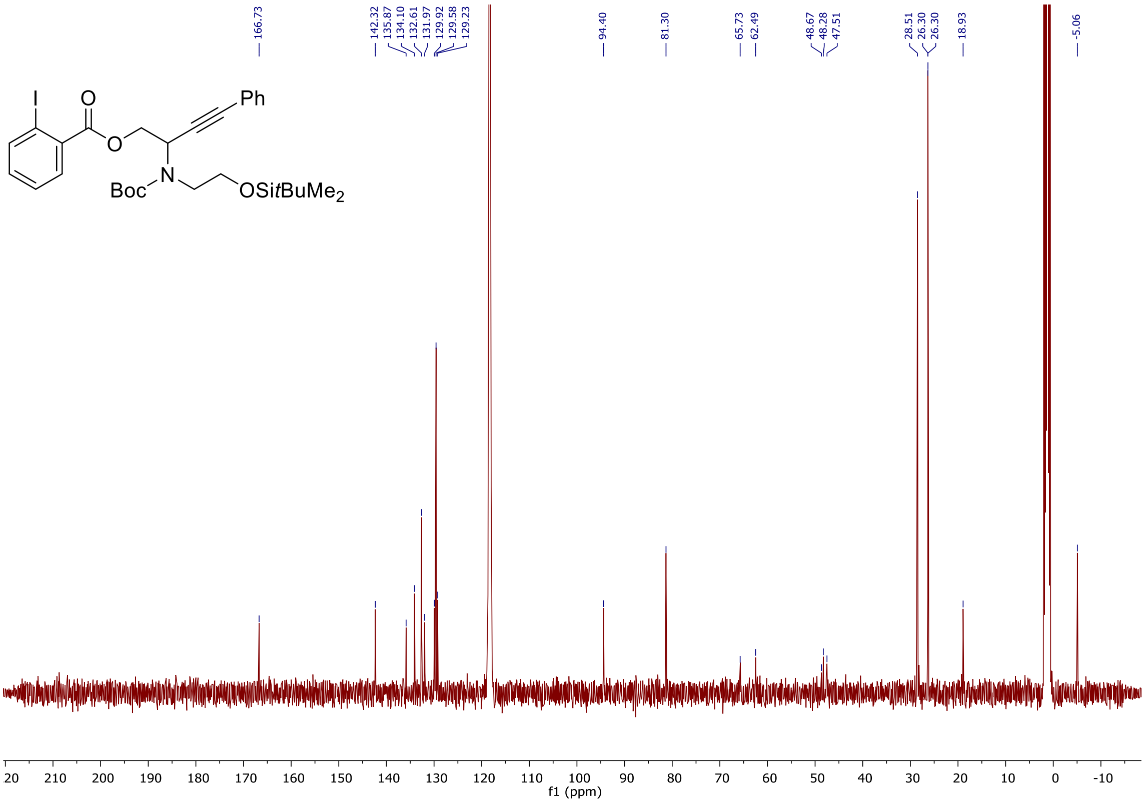

2-((tert-butoxycarbonyl)(3-ethoxy-3-oxopropyl)amino)-4-phenylbut-3-yn-1-yl 2-iodobenzoate (**3h**)

^{1}H NMR (400 MHz, Acetonitrile-d_3)

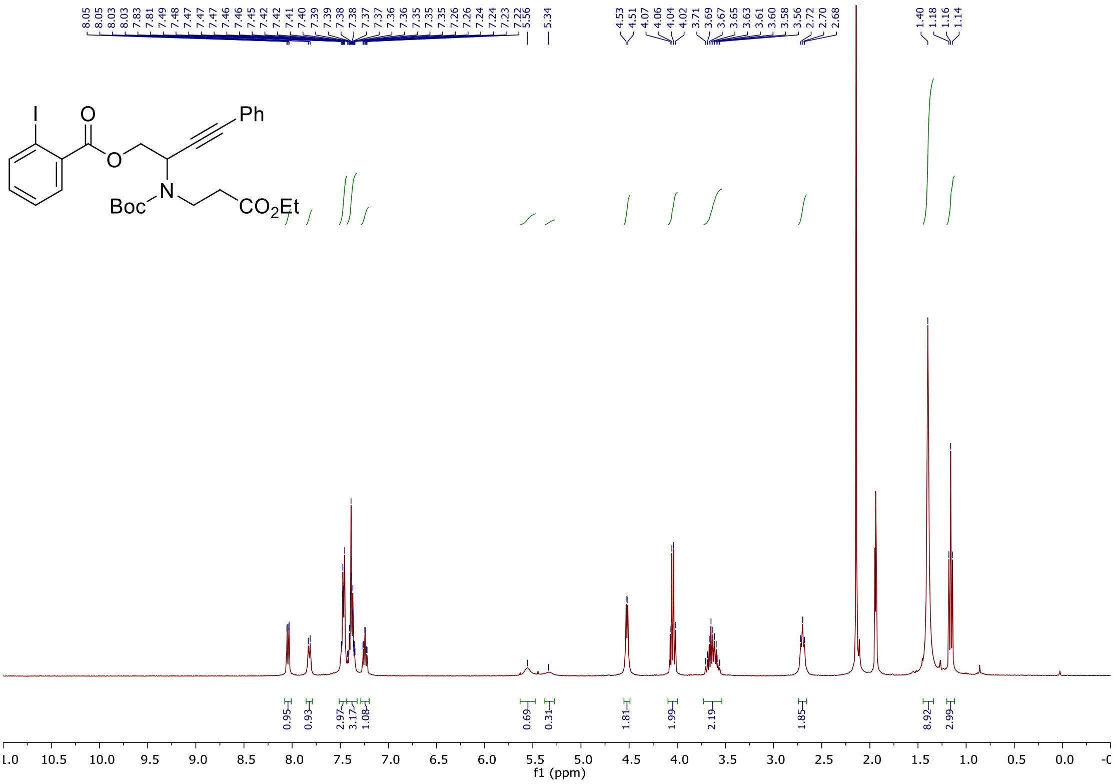

^{13}C NMR (101 MHz, Acetonitrile-d_3)

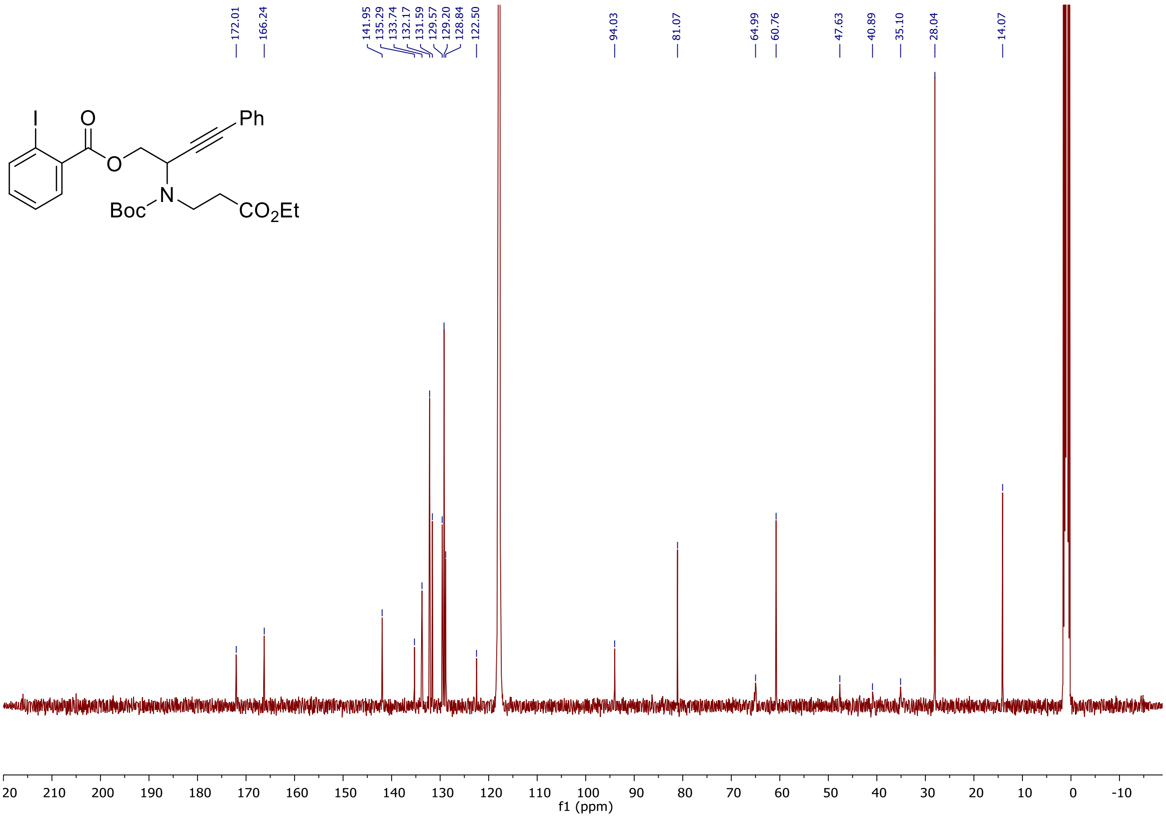

2-((*tert*-butoxycarbonyl)(cyclohexyl)amino)-4-phenylbut-3-yn-1-yl 2-iodobenzoate (**3i**)

^{1}H NMR (400 MHz, Acetonitrile-d_3)

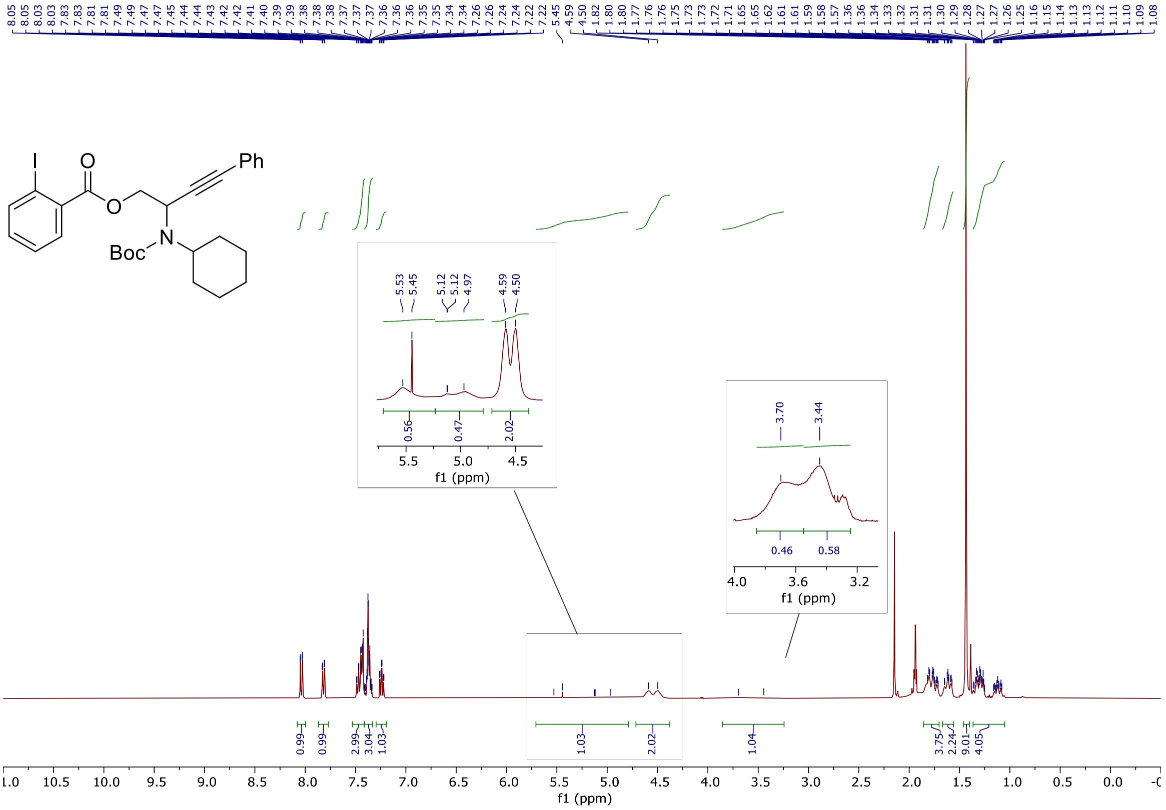

^{13}C NMR (101 MHz, Acetonitrile-d)

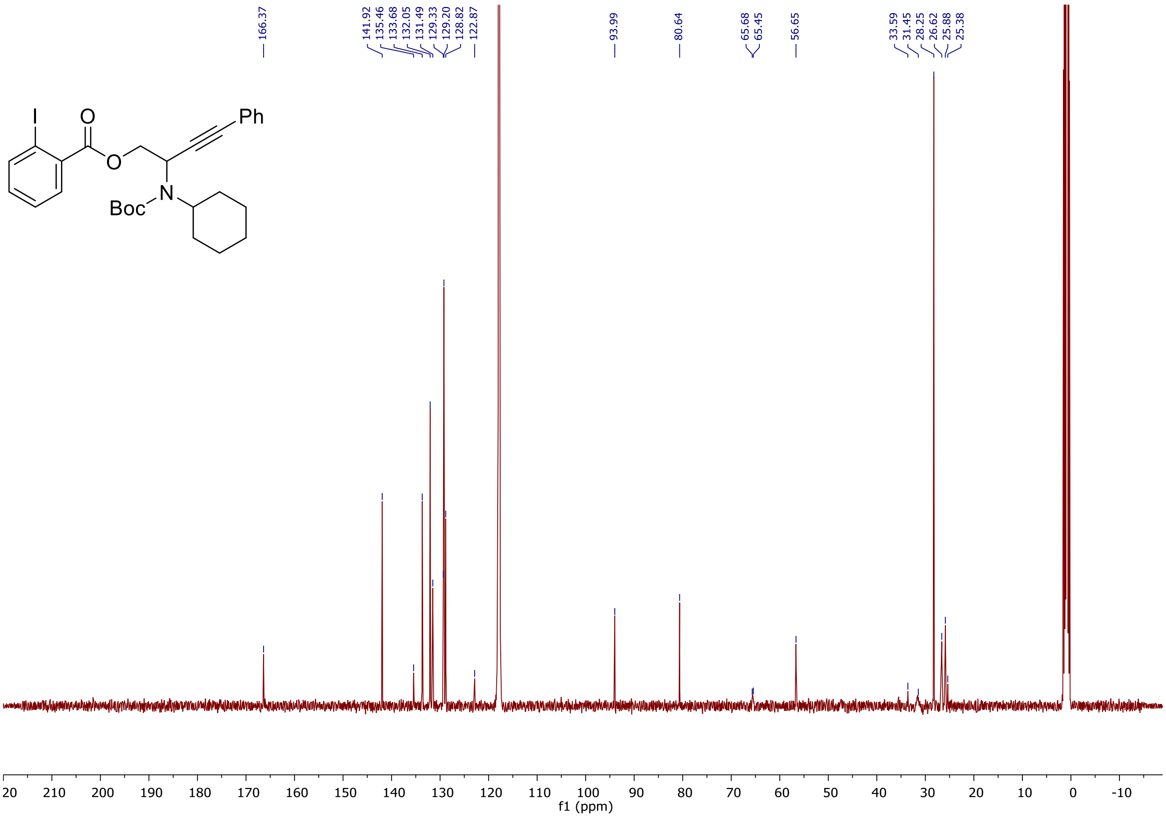

2-(2-oxopyrrolidin-1-yl)-4-phenylbut-3-yn-1-yl 2-iodobenzoate (**3j**)

^{1}H NMR (400 MHz, Acetonitrile-d_3)

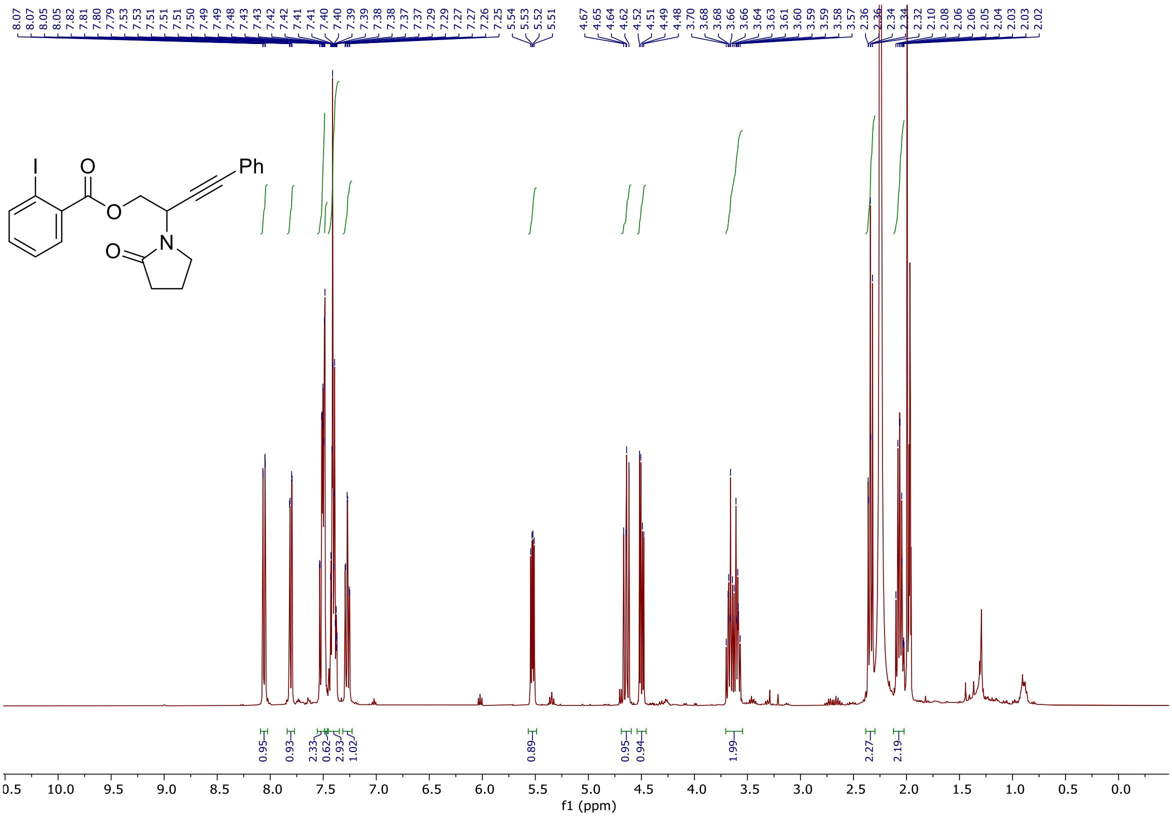

^{13}C NMR (101 MHz, Acetonitrile-d_3)

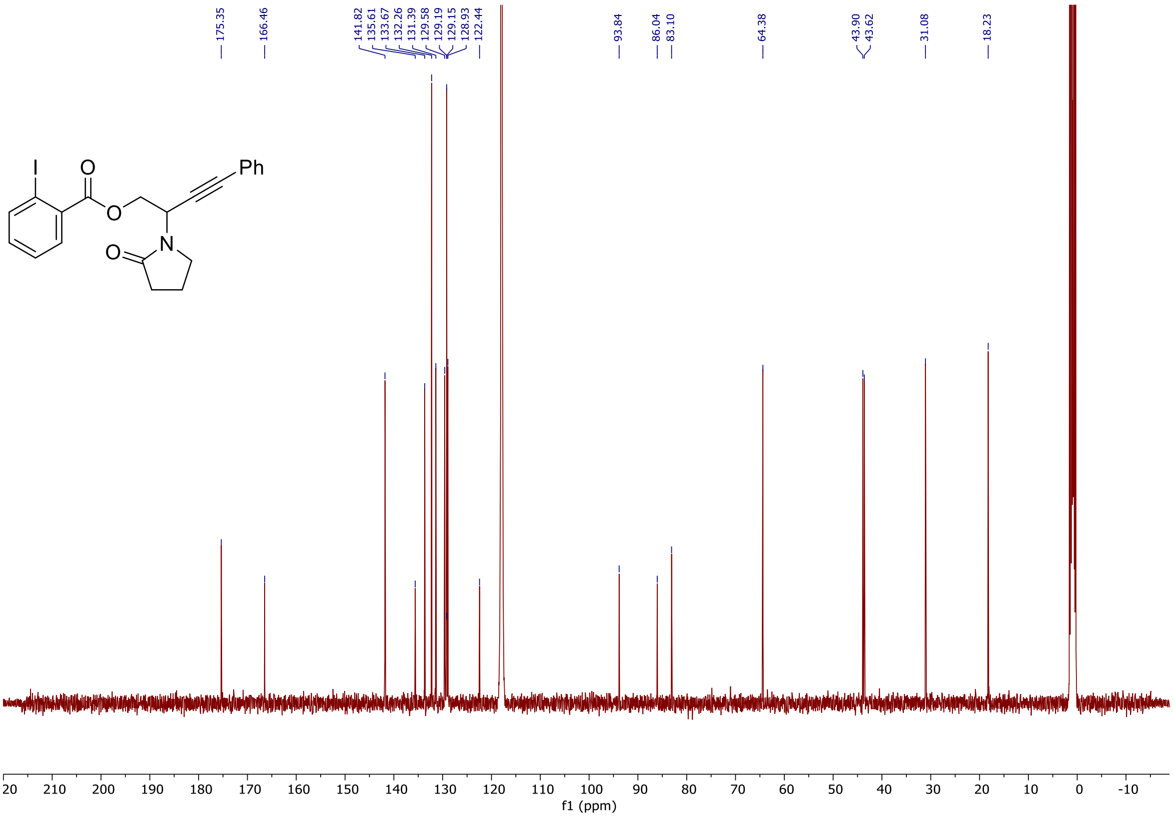

3-(2-oxooxazolidin-3-yl)-1-phenyloct-1-yn-4-yl 2-iodobenzoate (**3l**-major diastereoisomer)

^{1}H NMR (400 MHz, Chloroform-*d*)

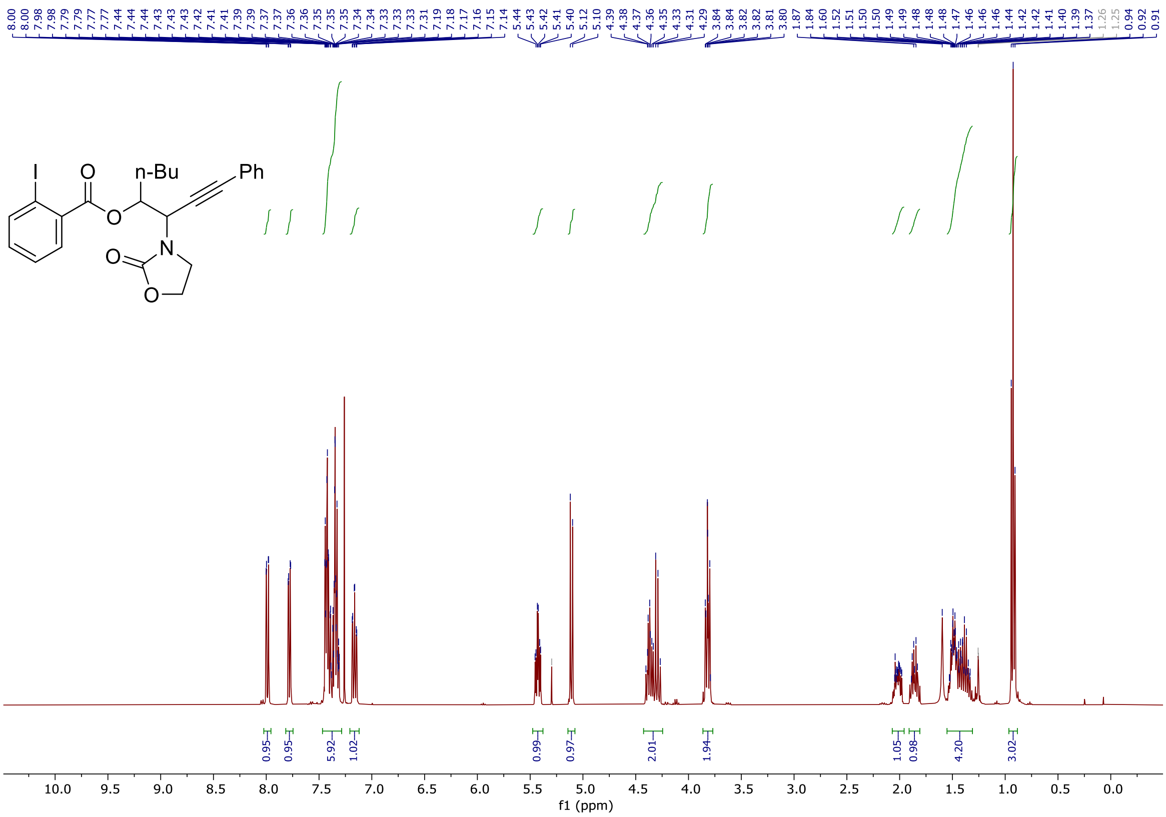

^{13}C NMR (101 MHz, Chloroform-*d*)

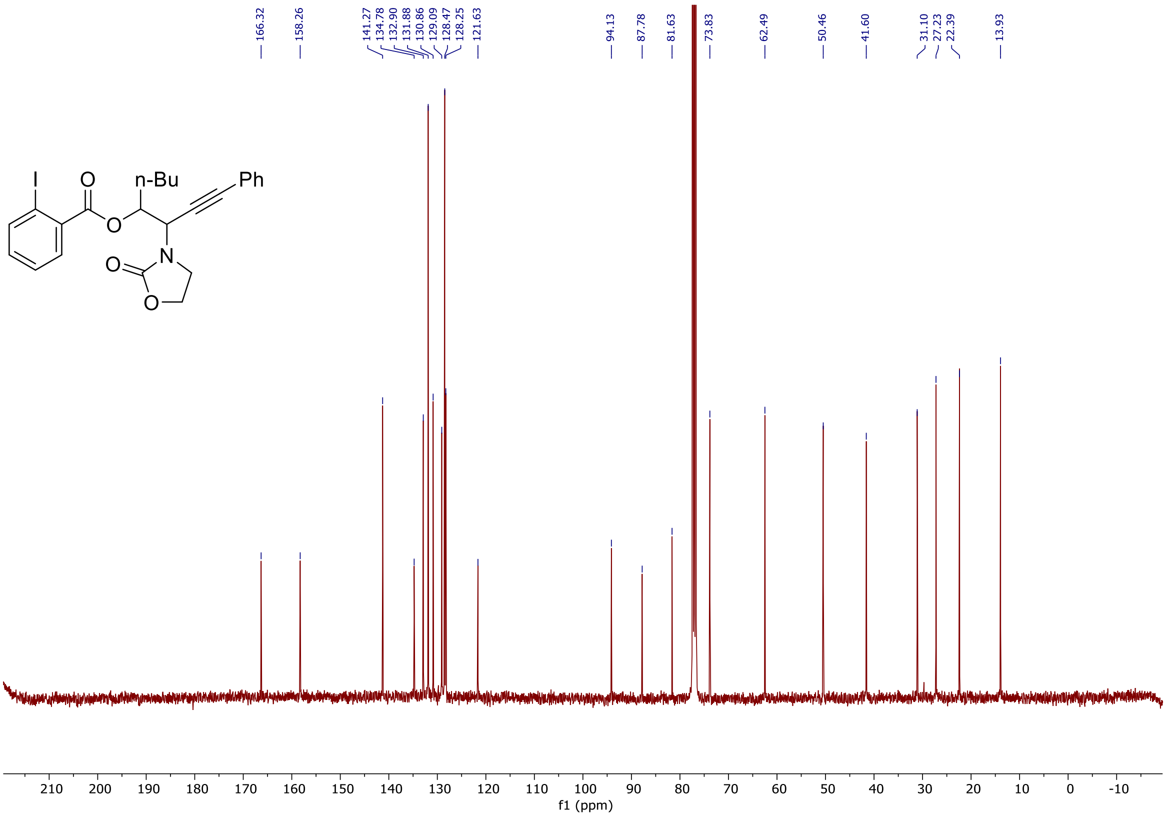

3-(2-oxooxazolidin-3-yl)-1-phenyloct-1-yn-4-yl 2-iodobenzoate (**3l**-minor diastereoisomer) (traces of DCM)

^{1}H NMR (400 MHz, Chloroform-*d*)

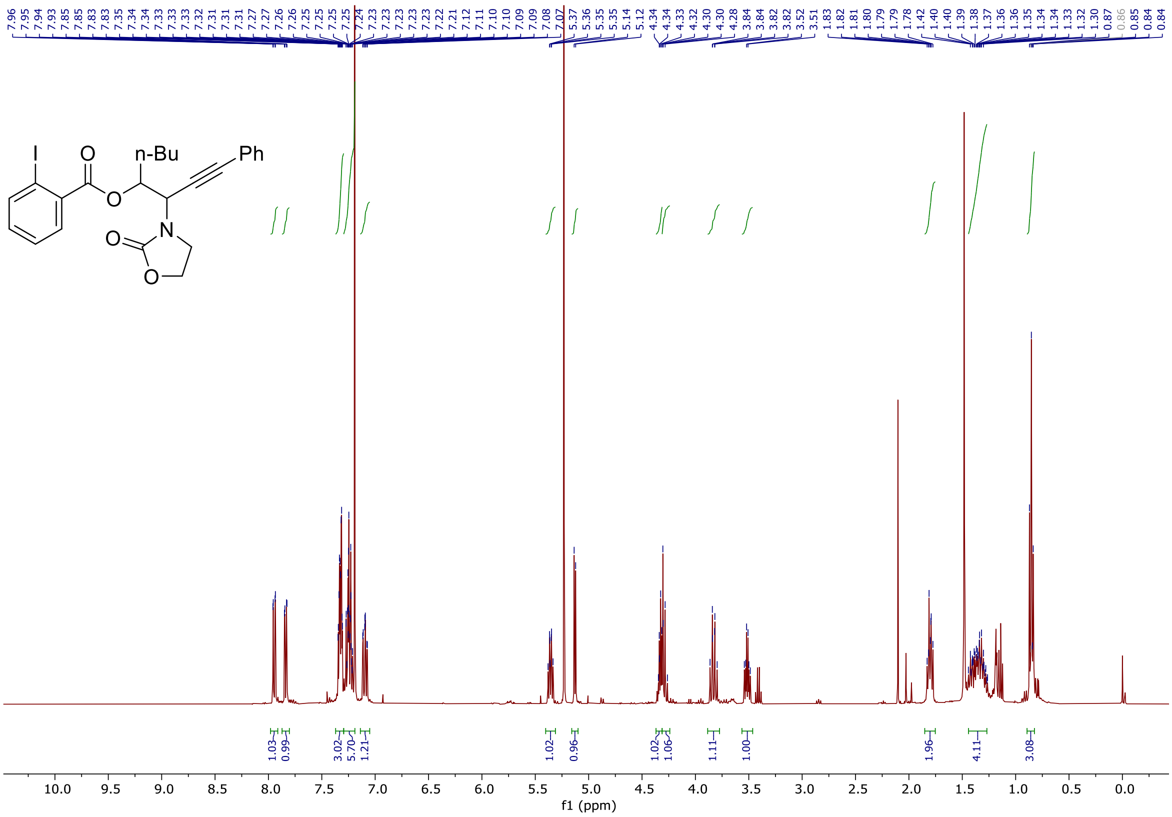

^{13}C NMR (101 MHz, Chloroform-*d*)

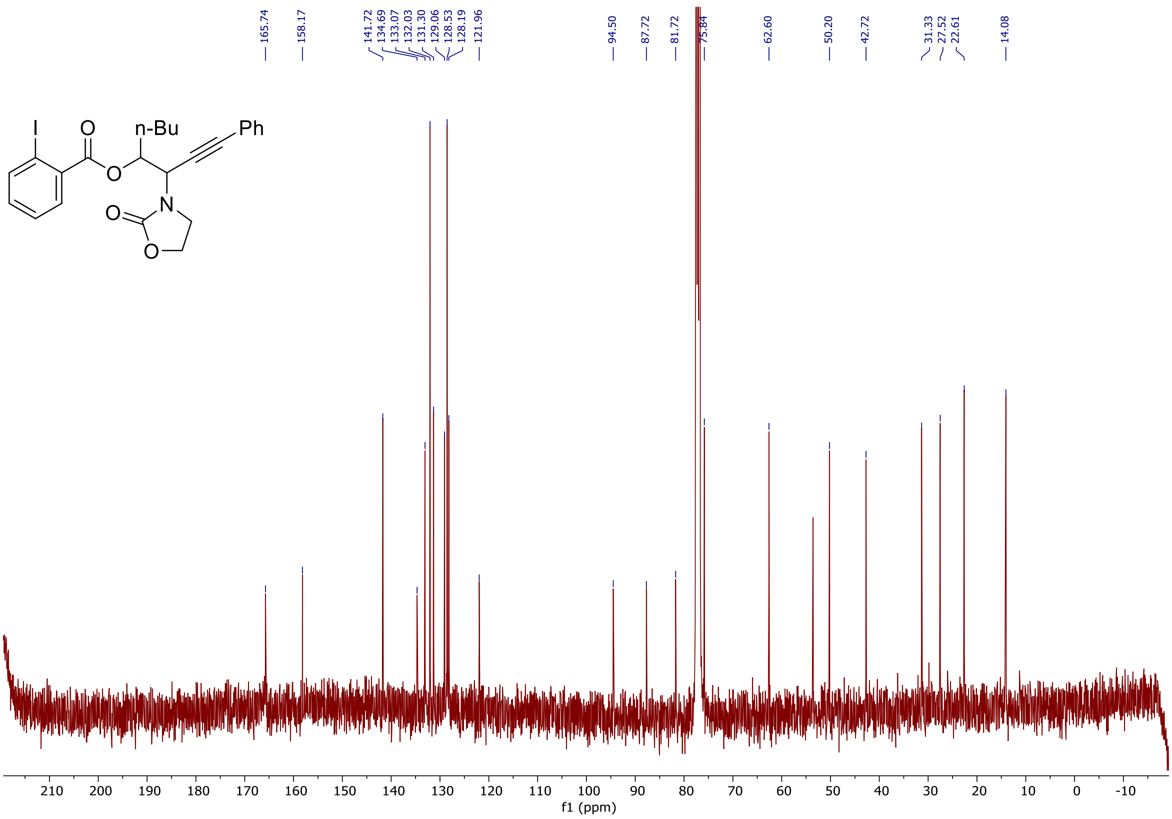

2-butoxy-4-phenylbut-3-yn-1-yl 2-iodobenzoate (**10a**)

^{1}H NMR (400 MHz, chloroform-*d*)

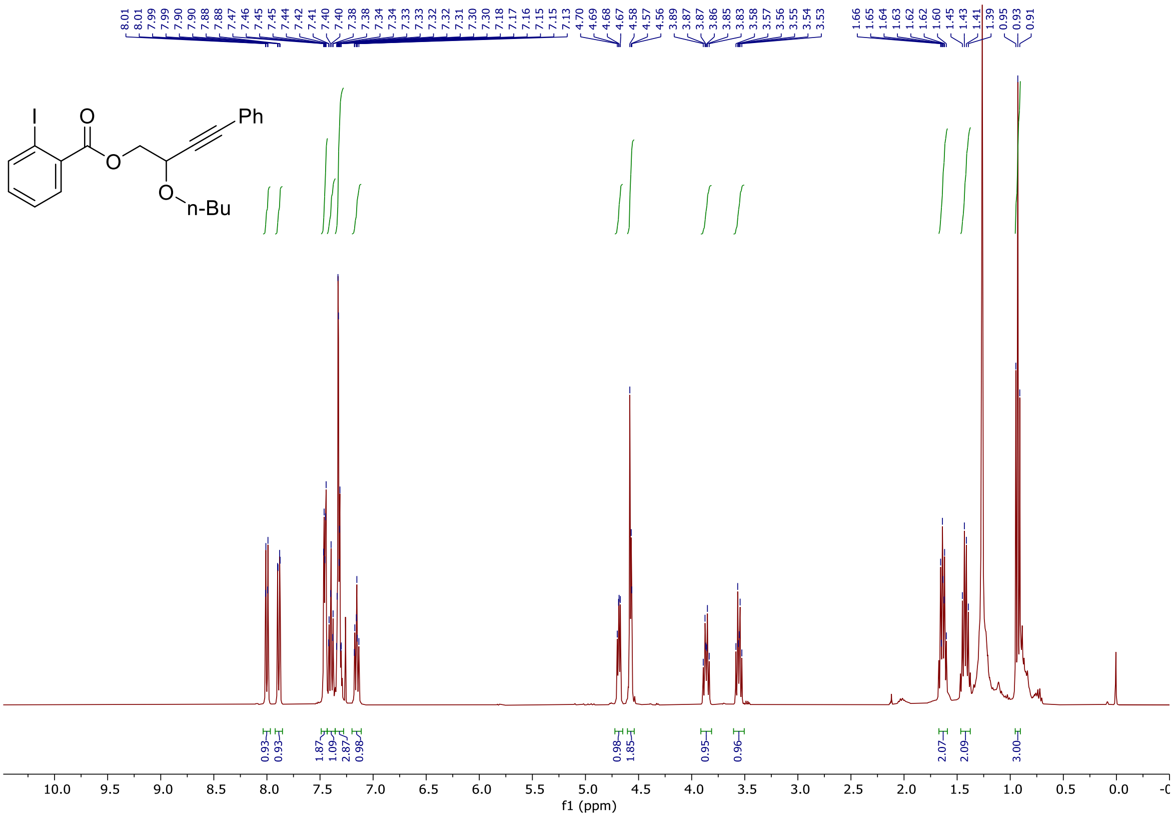

^{13}C NMR (400 MHz, chloroform-*d*)

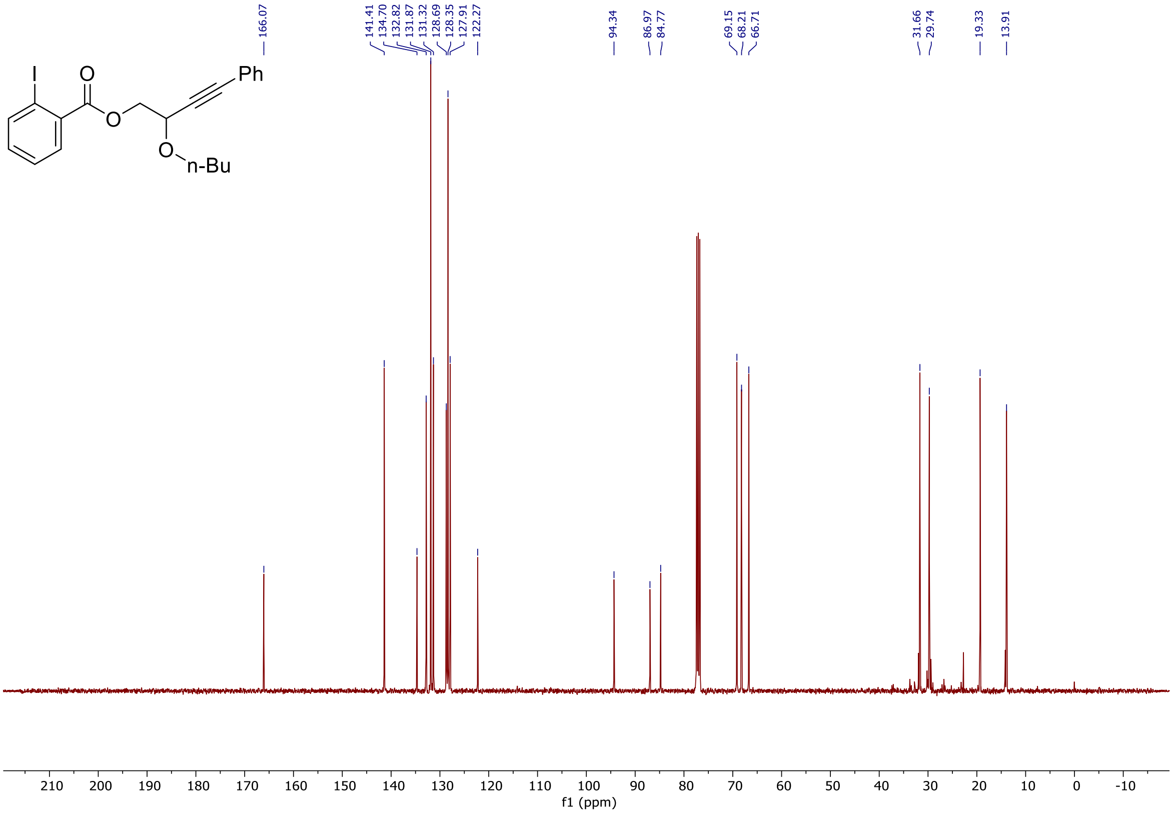

2-benzyloxy-4-phenylbut-1-yn-4-yl 2-iodobenzoate (**10b**)

^{1}H NMR (400 MHz, Acetonitrile-d_3)

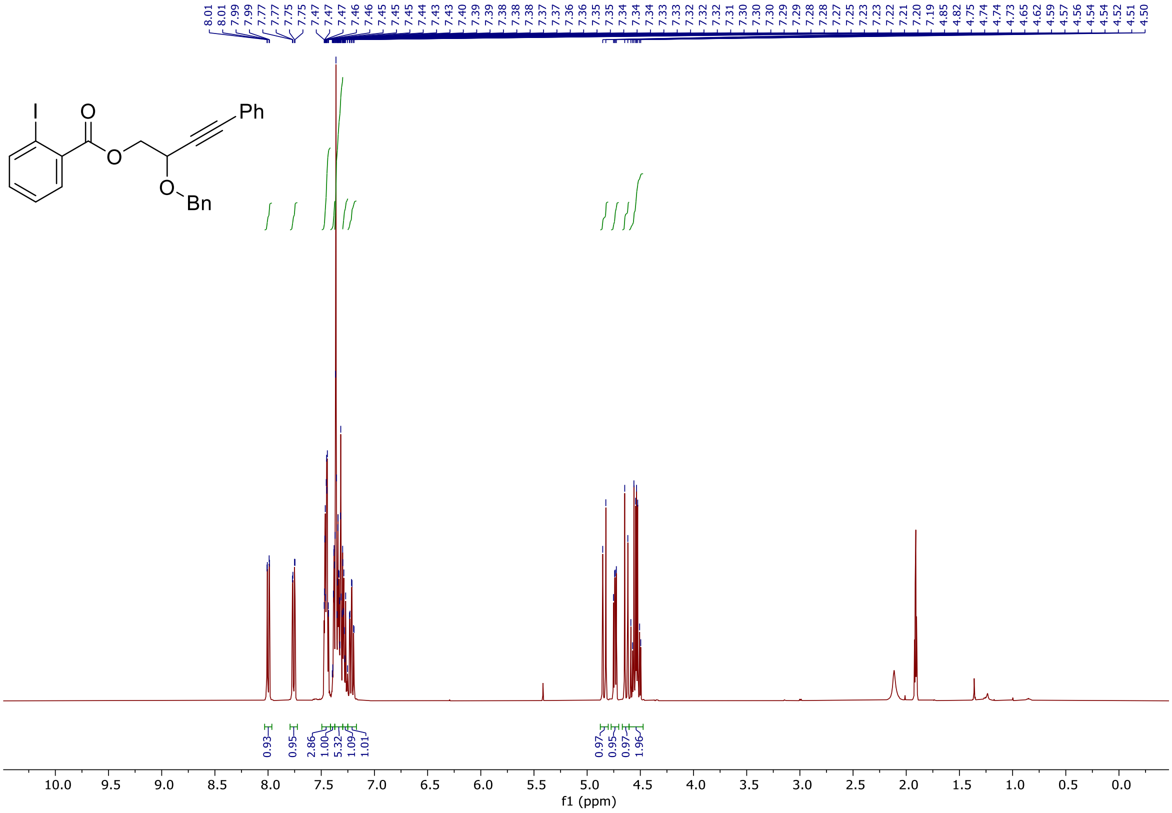

^{13}C NMR (400 MHz, Acetonitrile-d3)

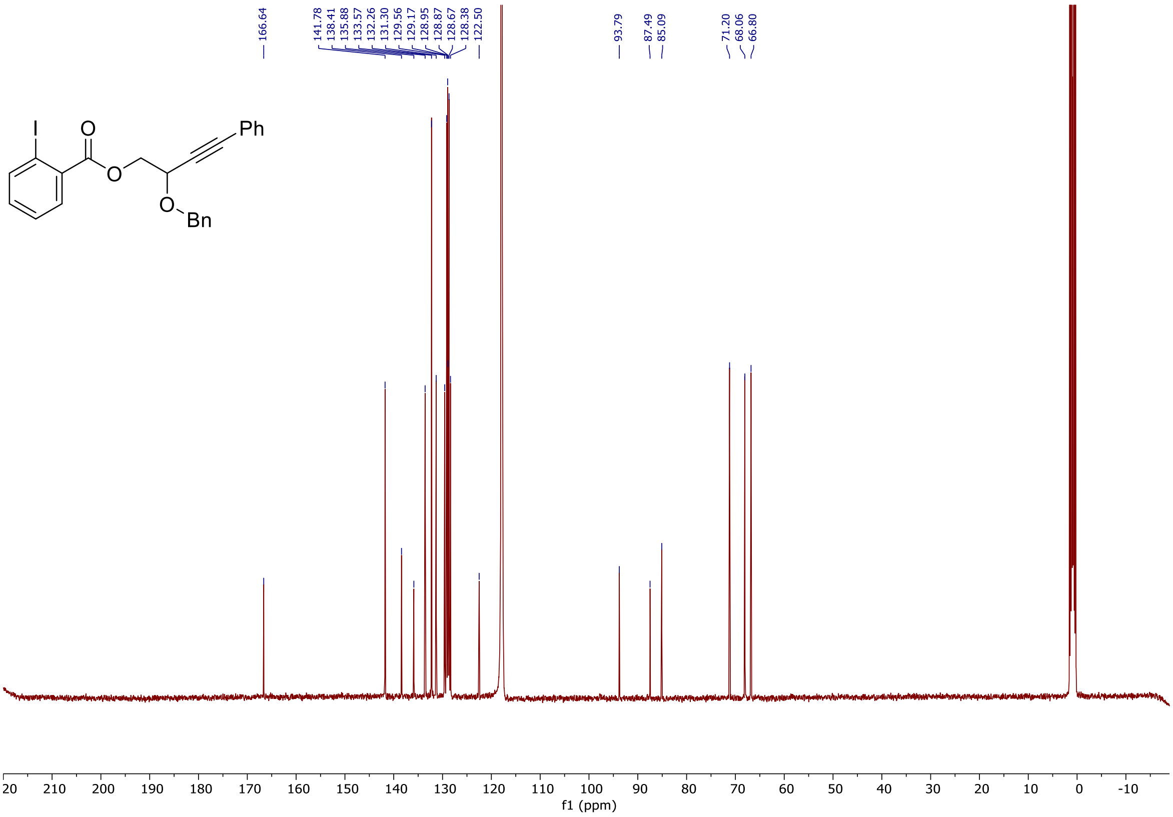

2-(allyloxy)-4-phenylbut-3-yn-1-yl 2-iodobenzoate (**10c**)

^{1}H NMR (400 MHz, Acetonitrile-d_3)

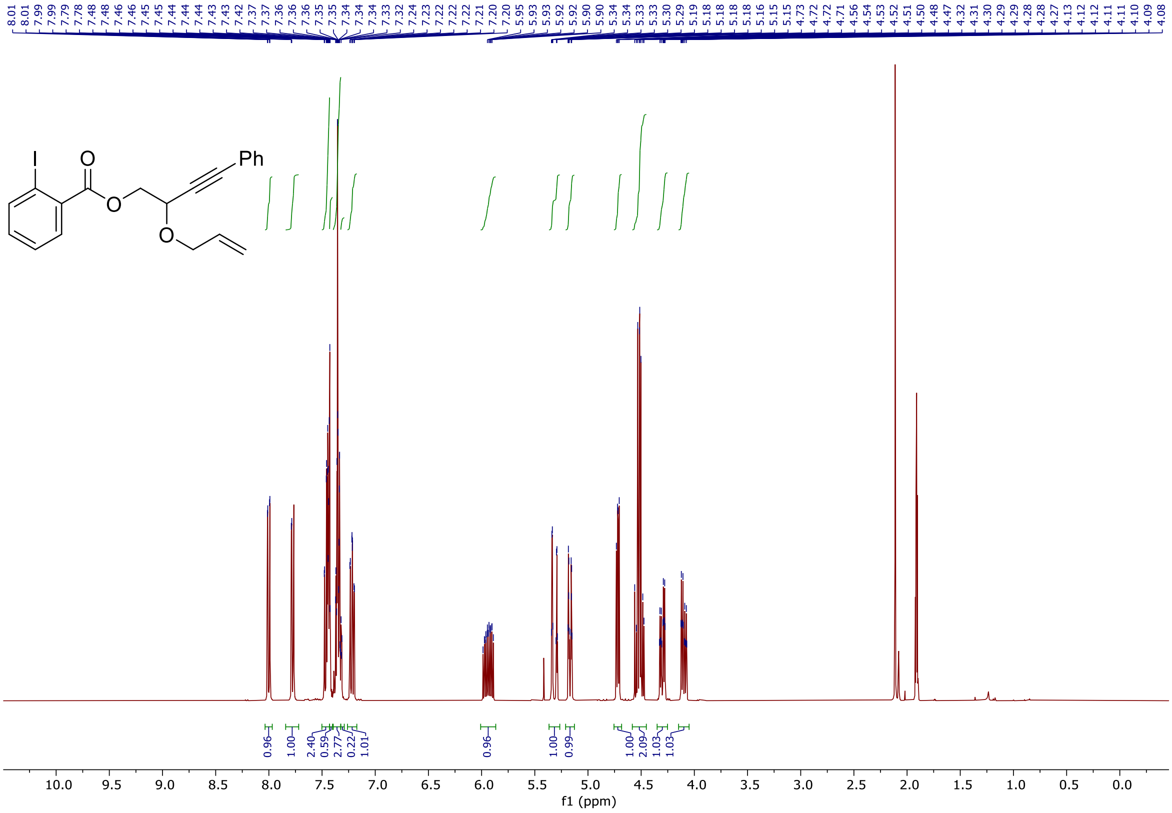

^{13}C NMR (400 MHz, Acetonitrile-d_3)

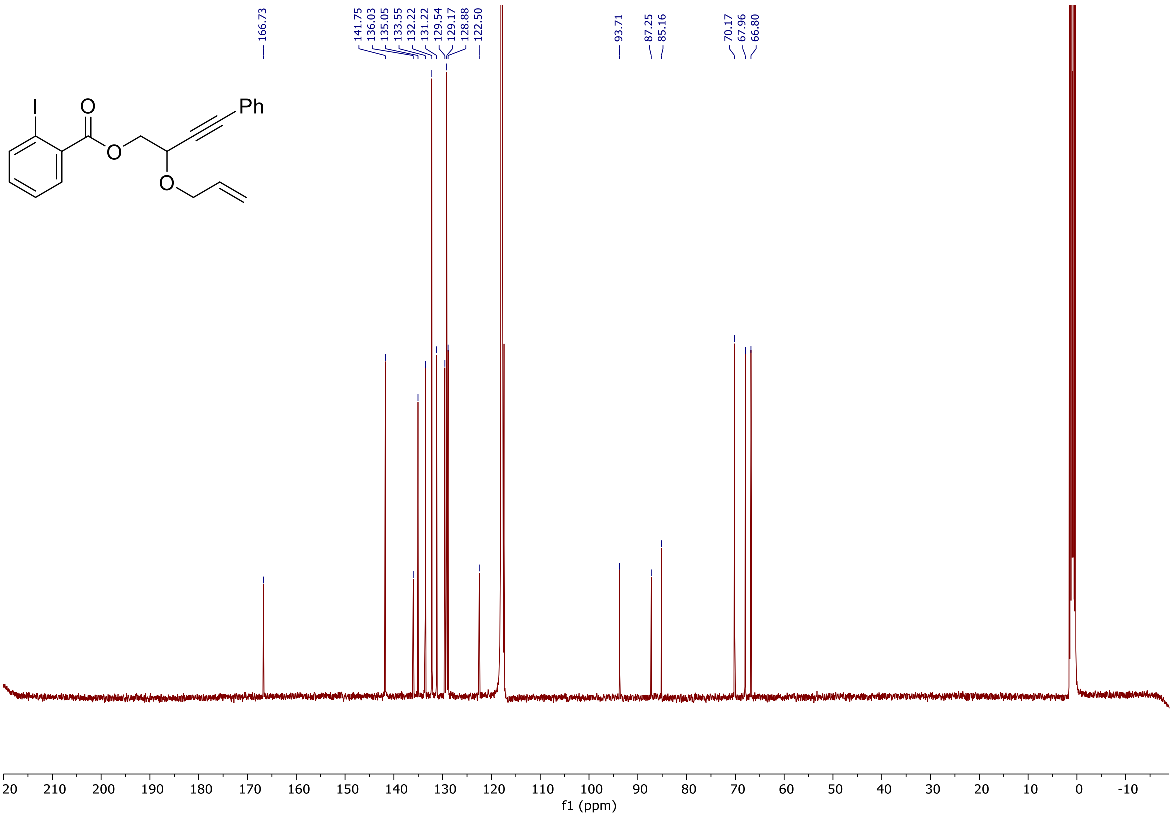

2-(2-chloroethoxy)-4-phenylbut-3-yn-1-yl 2-iodobenzoate (**10d**)

^{1}H NMR (400 MHz, chloroform-*d*)

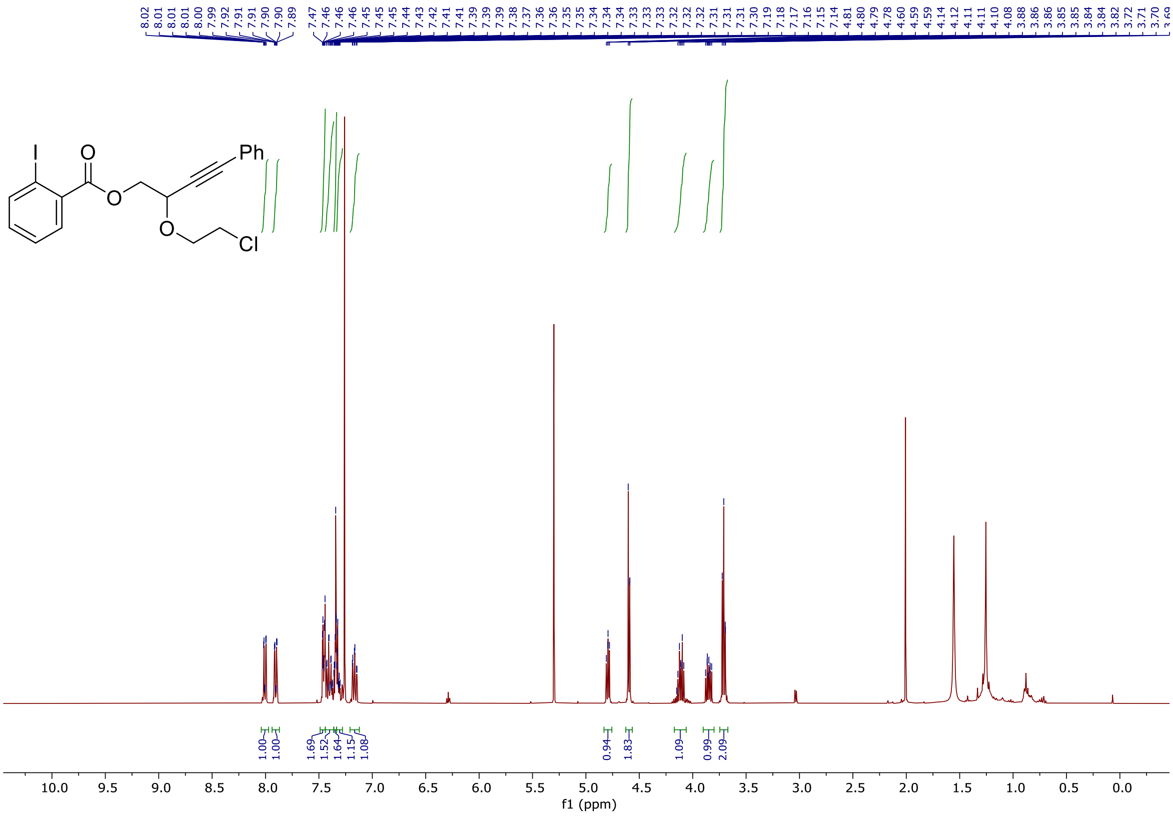

^{13}C NMR (400 MHz, chloroform-*d*)

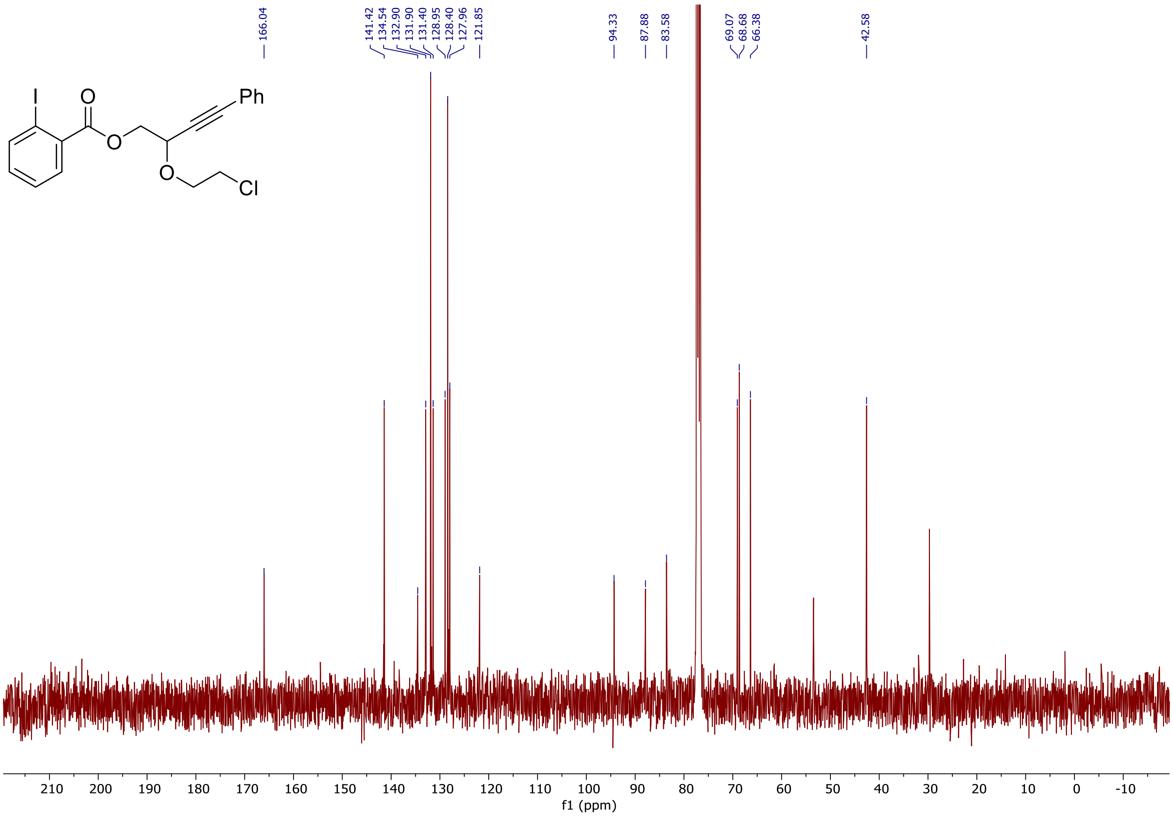

2-cyclohexyloxy-4-phenylbut-1-yn-4-yl 2-iodobenzoate (**10e**)

^{1}H NMR (400 MHz, chloroform-*d*)

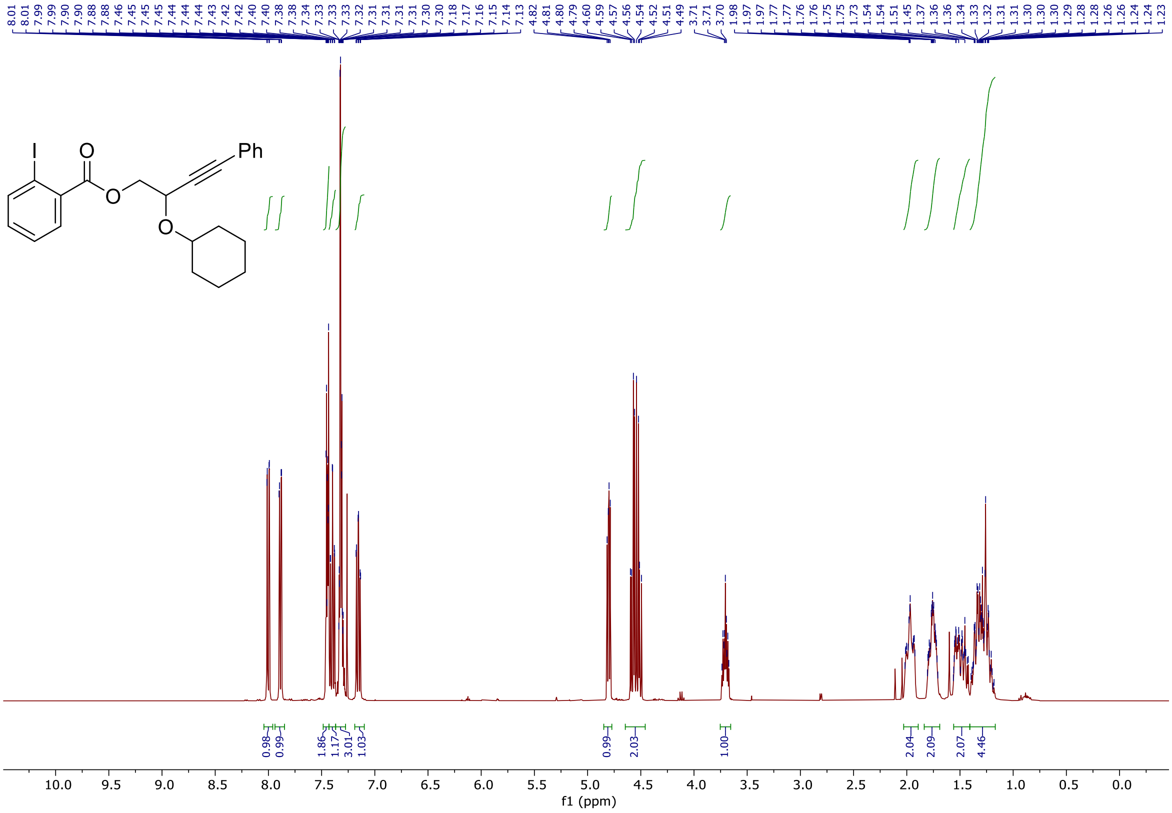

^{13}C NMR (400 MHz, chloroform-*d*)

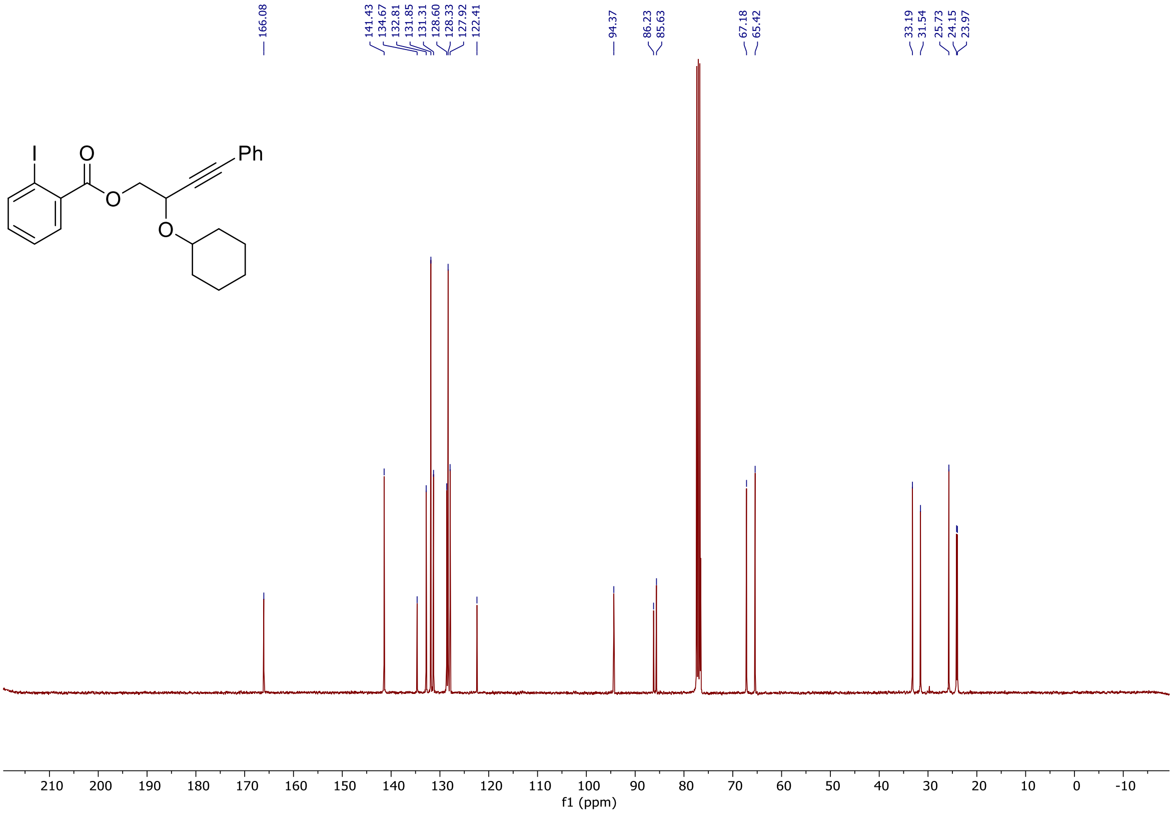

2-ethoxyoxy-2-methyl-4-phenylbut-1-yn-4-yl 2-iodobenzoate (**10f**)

^{1}H NMR (400 MHz, chloroform-*d*)

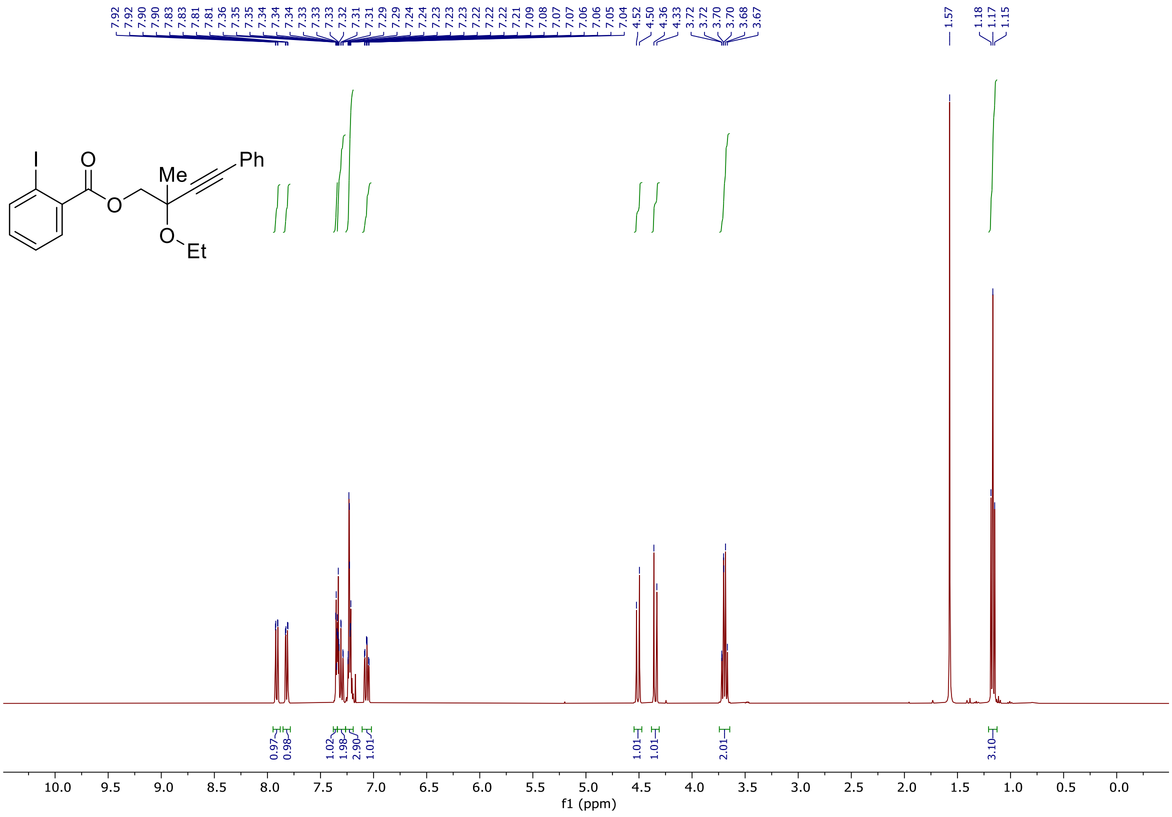

^{13}C NMR (400 MHz, chloroform-*d*)

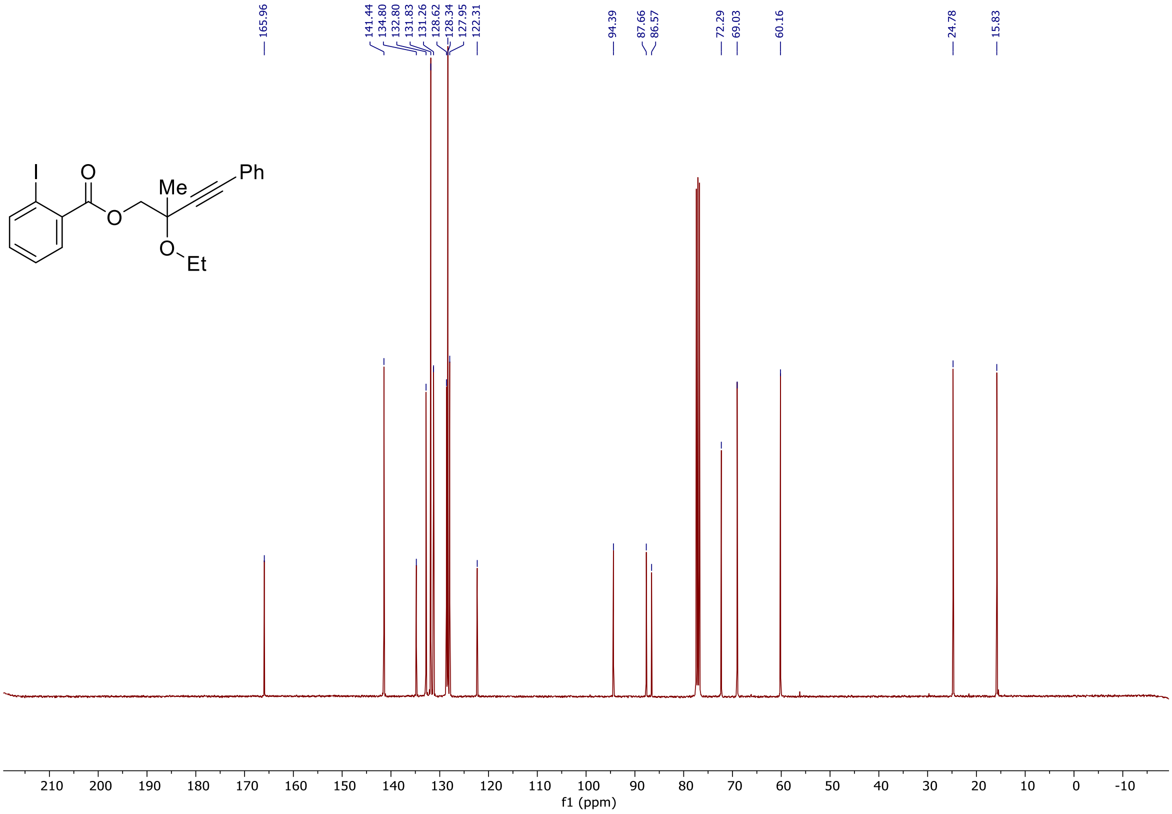

1-(benzyloxy)-2-(phenylethynyl)hexyl 2-iodobenzoate (**10ga**)

^{1}H NMR (400 MHz, chloroform-*d*)

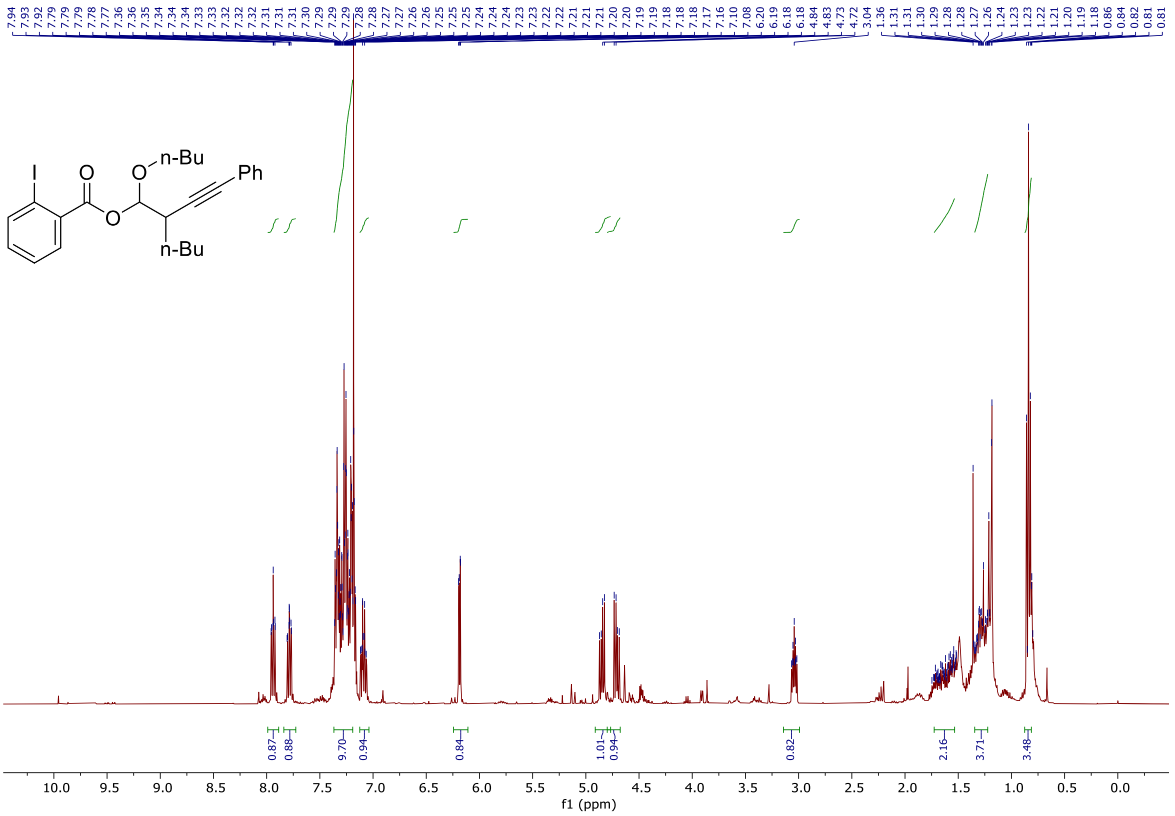

^{13}C NMR (400 MHz, chloroform-*d*)

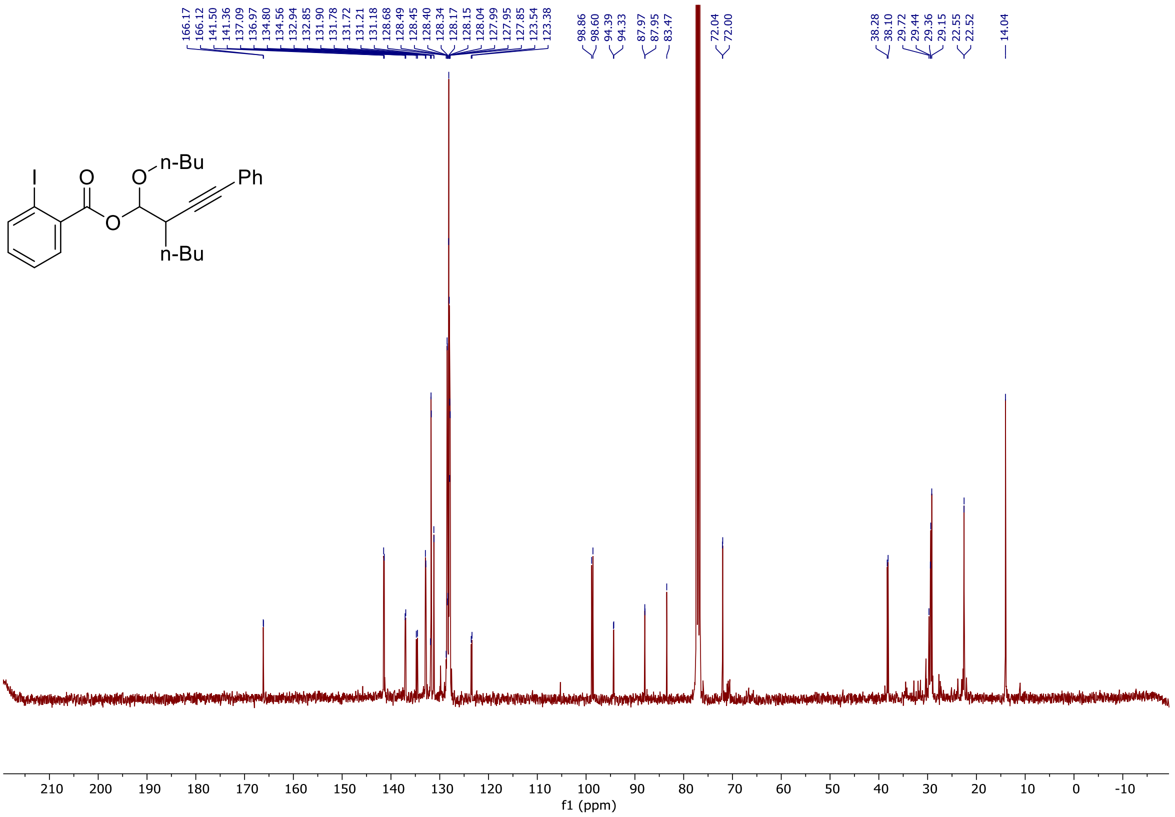

3-(benzyloxy)-1-phenyloct-1-yn-4-yl 2-iodobenzoate (**10gb**)

^{1}H NMR (400 MHz, chloroform-d)

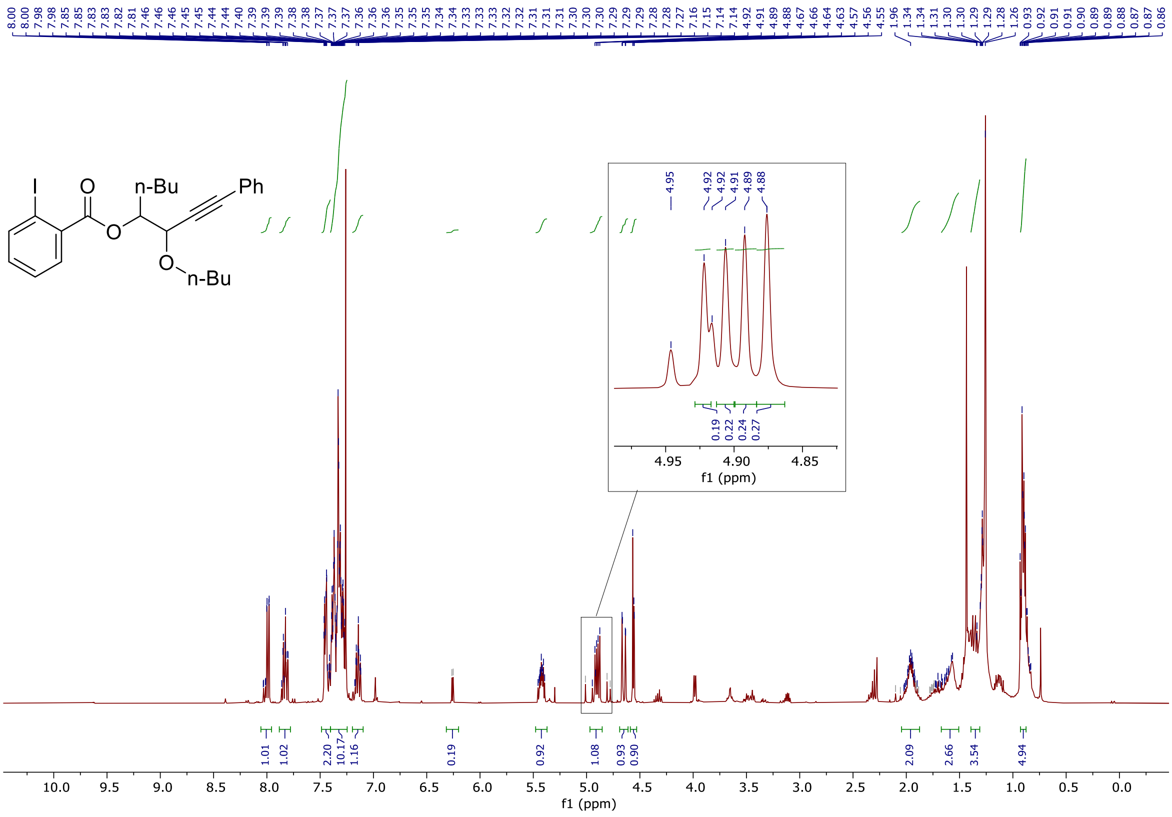

^{13}C NMR (400 MHz, chloroform-d)

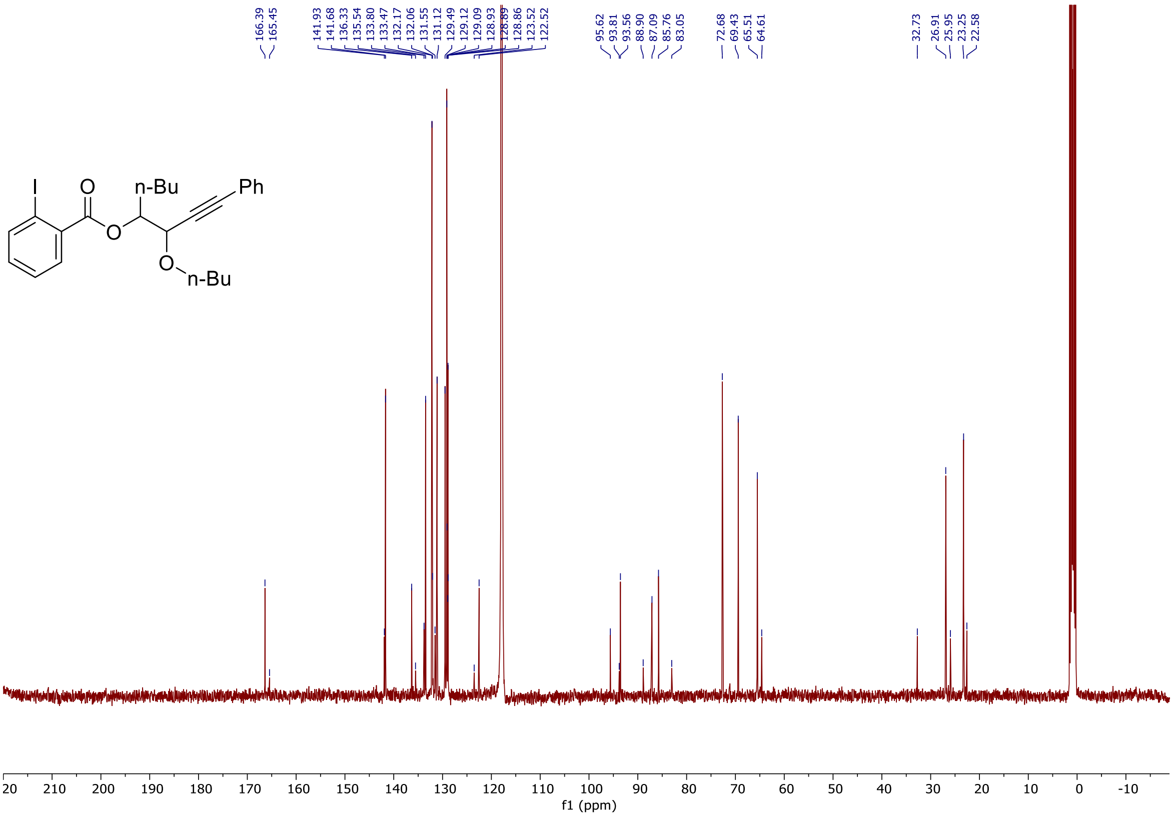

2-(phenylethynyl)tetrahydro-2H-pyran-3-yl 2-iodobenzoate (**10ha**) 3-(phenylethynyl)tetrahydro-2H-pyran-2-yl 2-iodobenzoate (**10hb**); 4:1 regioisomeric mixture

^{1}H NMR (400 MHz, Acetonitrile-d_3)

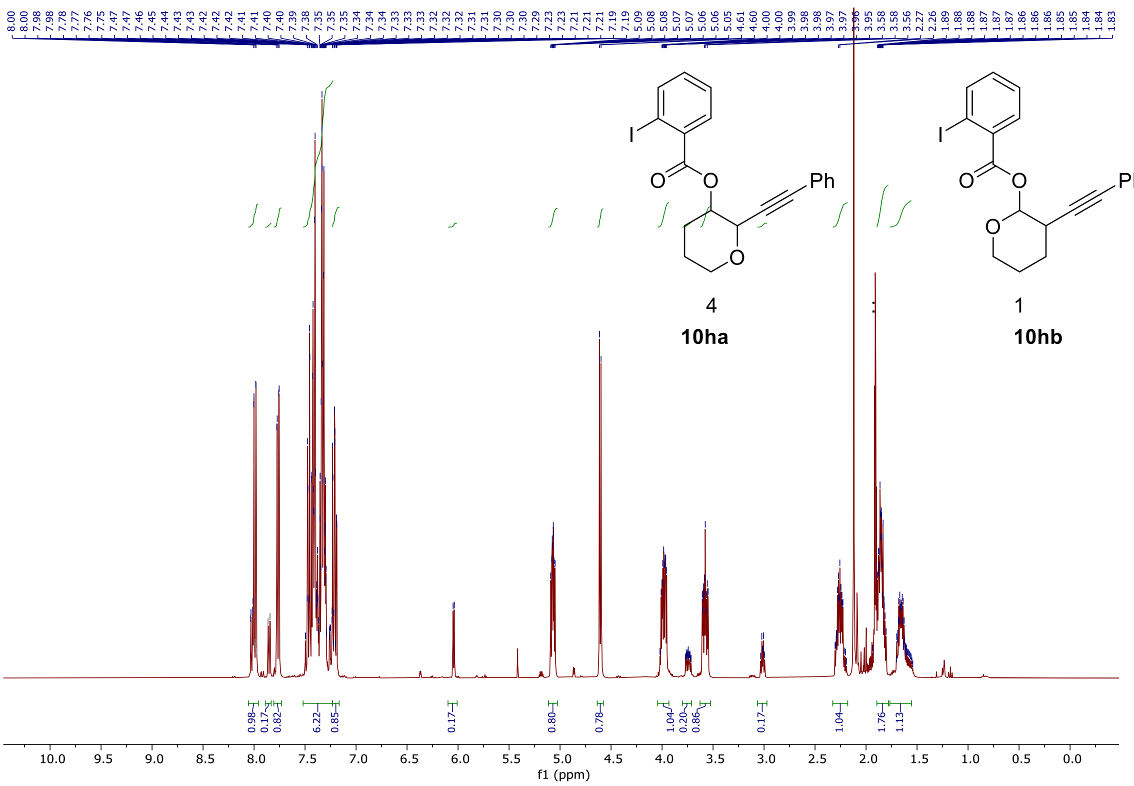

^{13}C NMR (400 MHz, Acetonitrile-d_3)

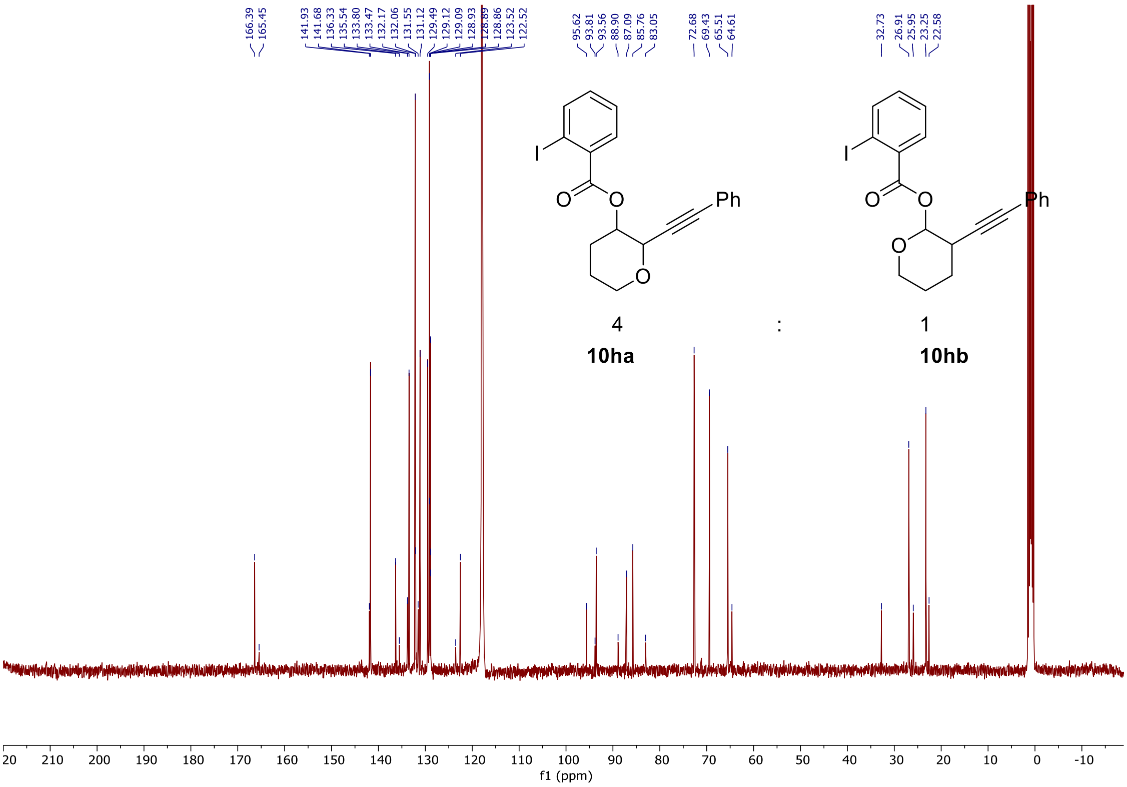

2-(((benzyloxy)carbonyl)(phenethyl)amino)-4-(4-(trifluoromethyl)phenyl)but-3-yn-1-yl 2-iodobenzoate (**12a**)

^{1}H NMR (400 MHz, Acetonitrile-d_3)

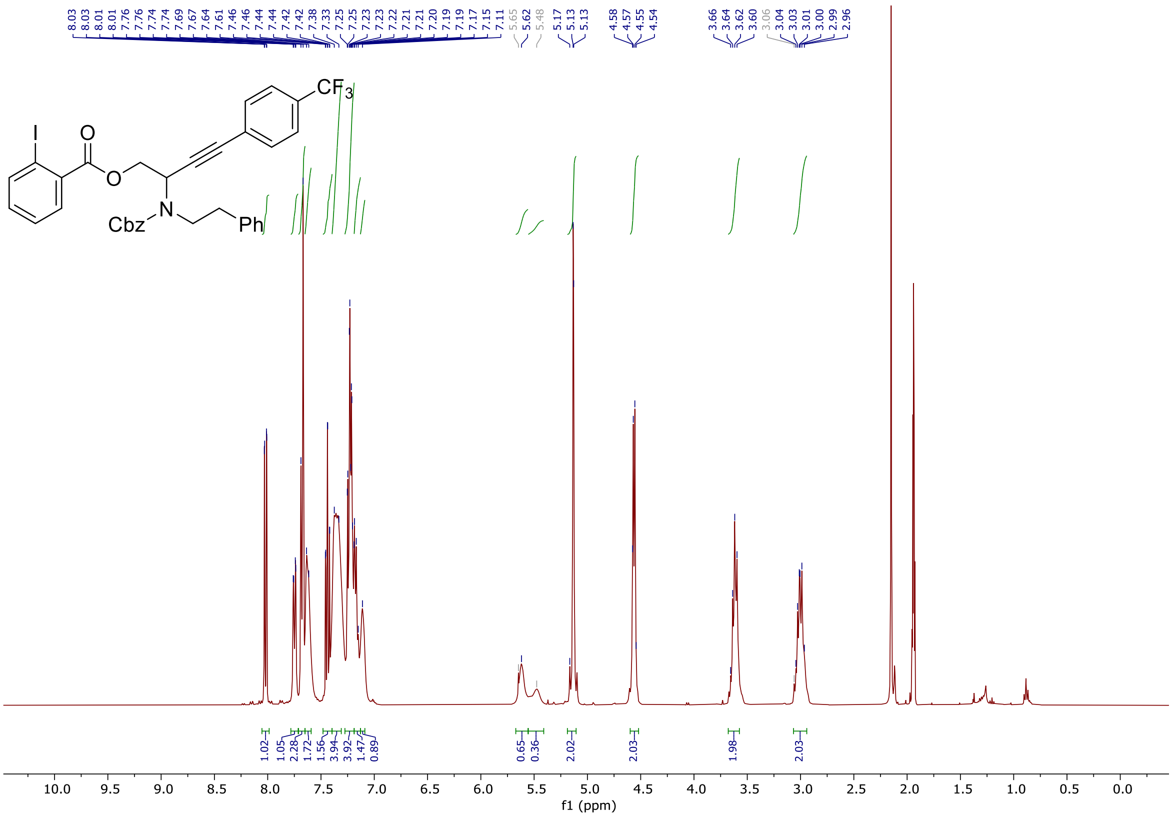

^{13}C NMR (400 MHz, Acetonitrile-d_3)

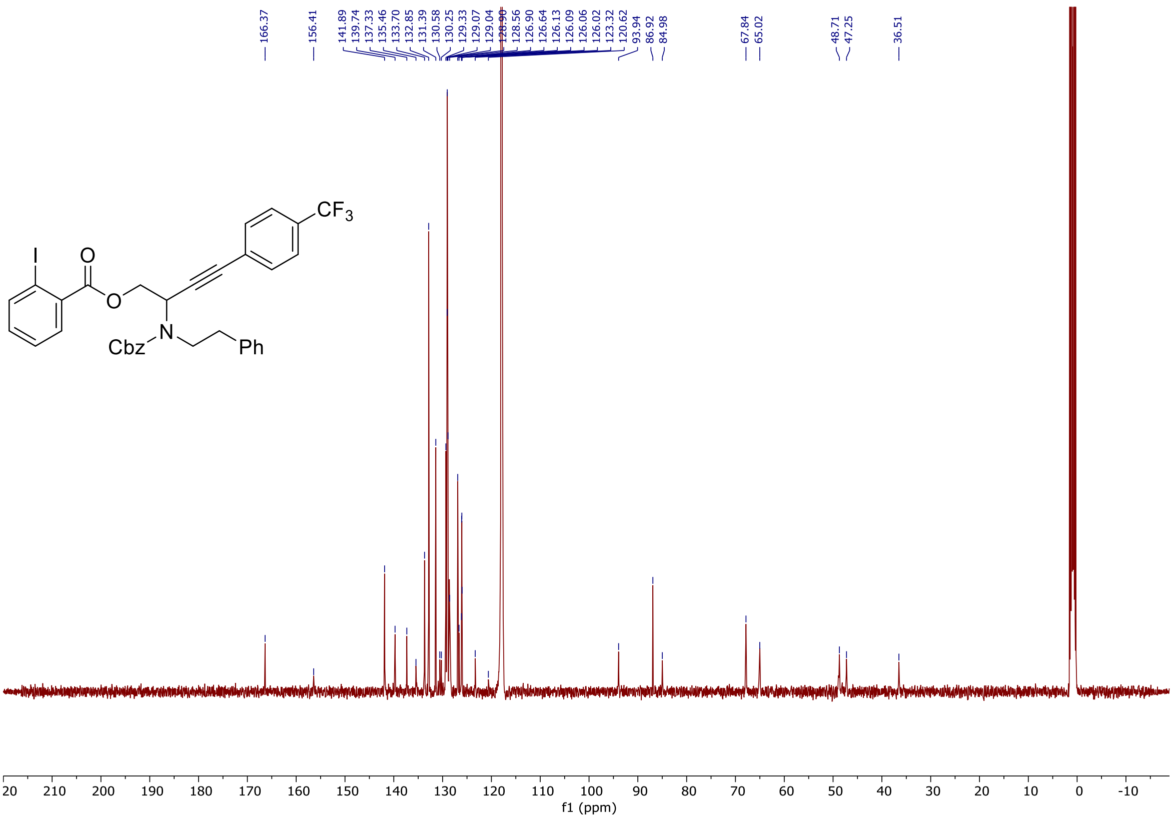

^{19}F NMR (376 MHz, Acetonitrile-d_3)

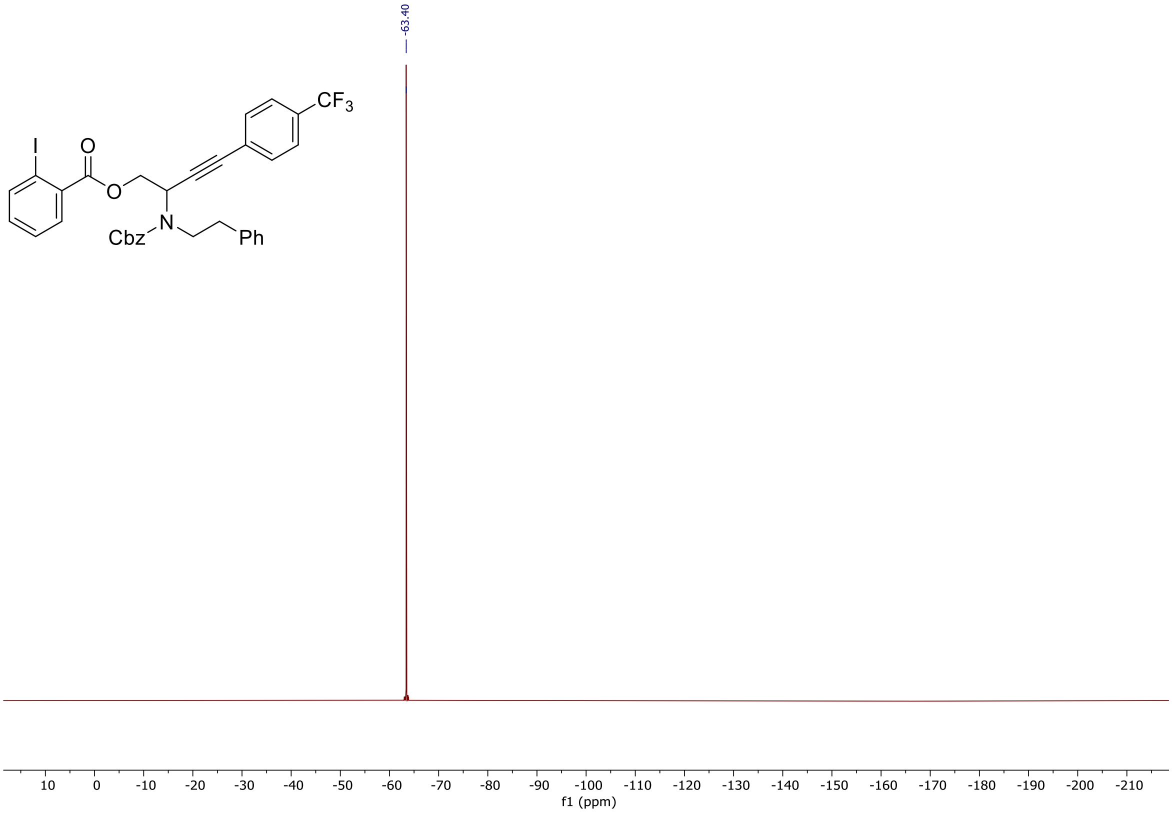

2-(((benzyloxy)carbonyl)(phenethyl)amino)-4-(4-bromophenyl)but-3-yn-1-yl 2-iodobenzoate (**12b**)

^{1}H NMR (400 MHz, Acetonitrile-d_3)

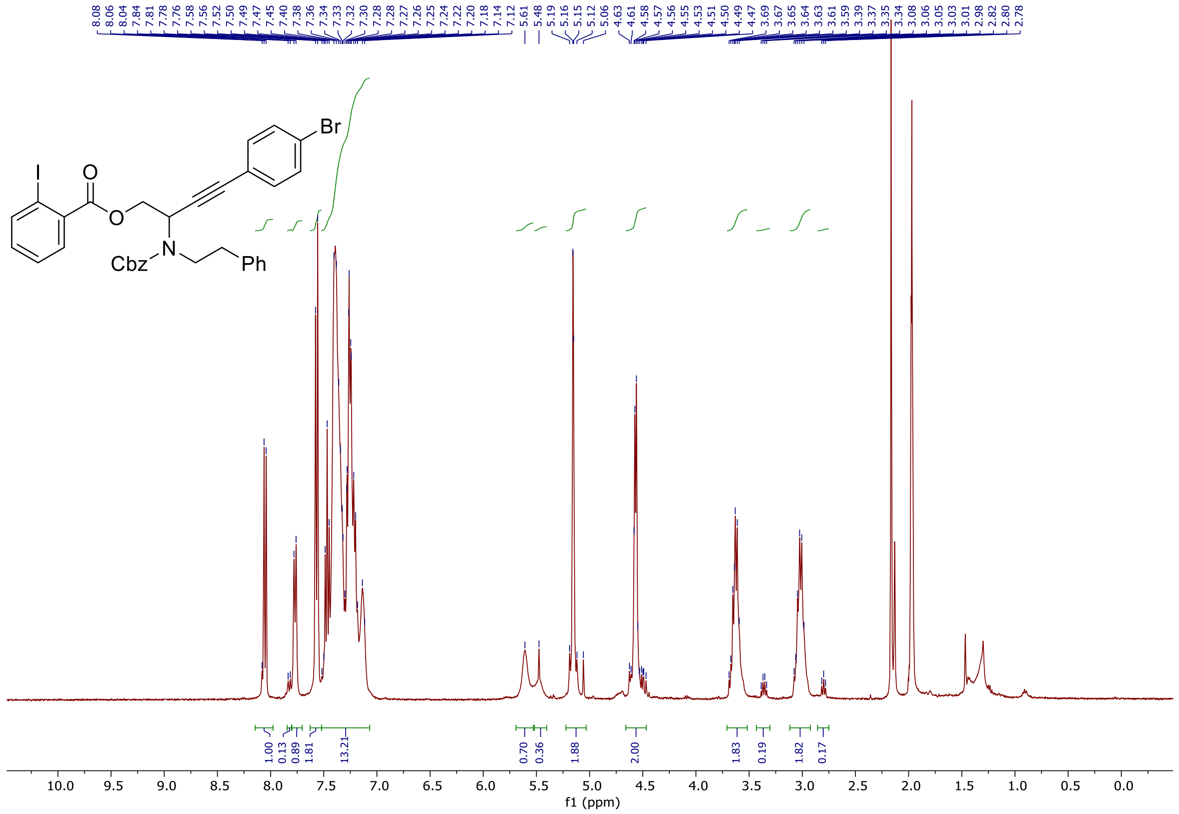

^{13}C NMR (400 MHz, Acetonitrile-d_3)

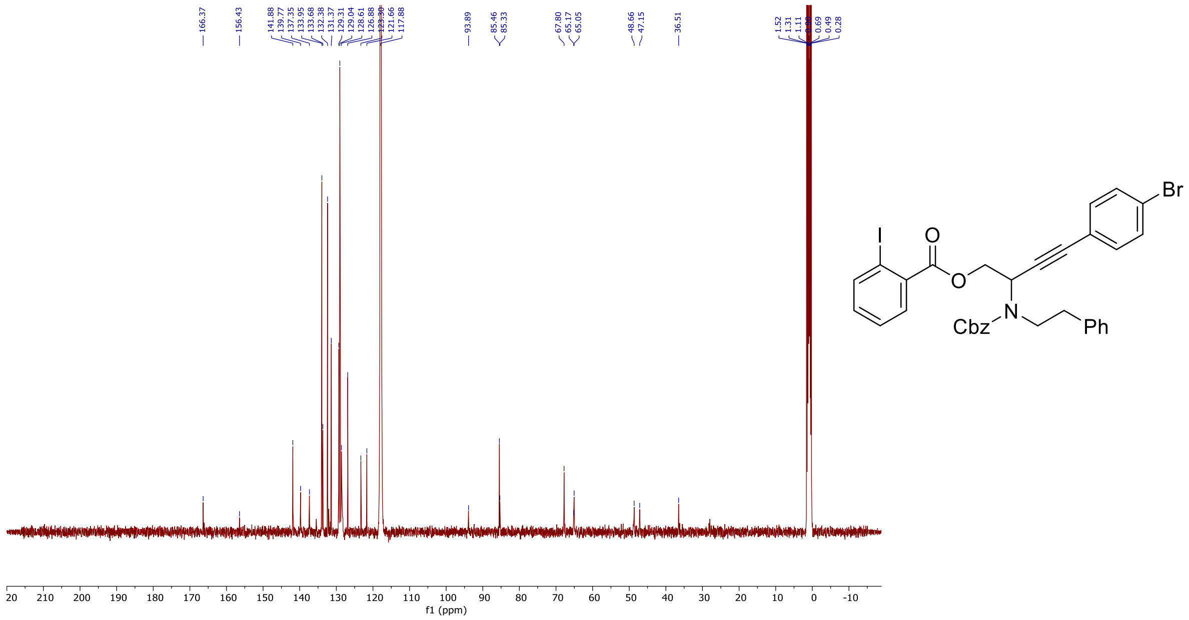

COSY NMR (400 MHz, Acetonitrile-d_3) Correlation between 1 and 2 and 1′ and 2′

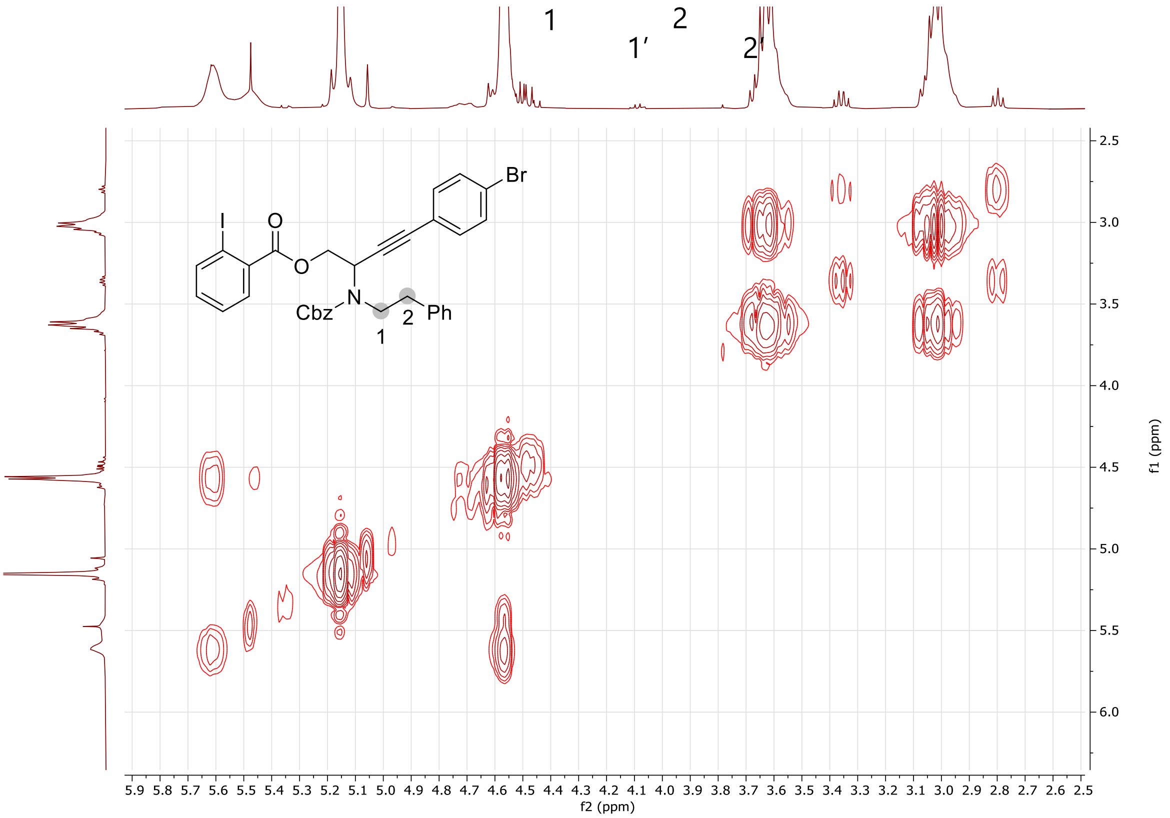

HMBC NMR (400 MHz, Acetonitrile-d_3) Correlation between 2 and 2′ to the same carbons

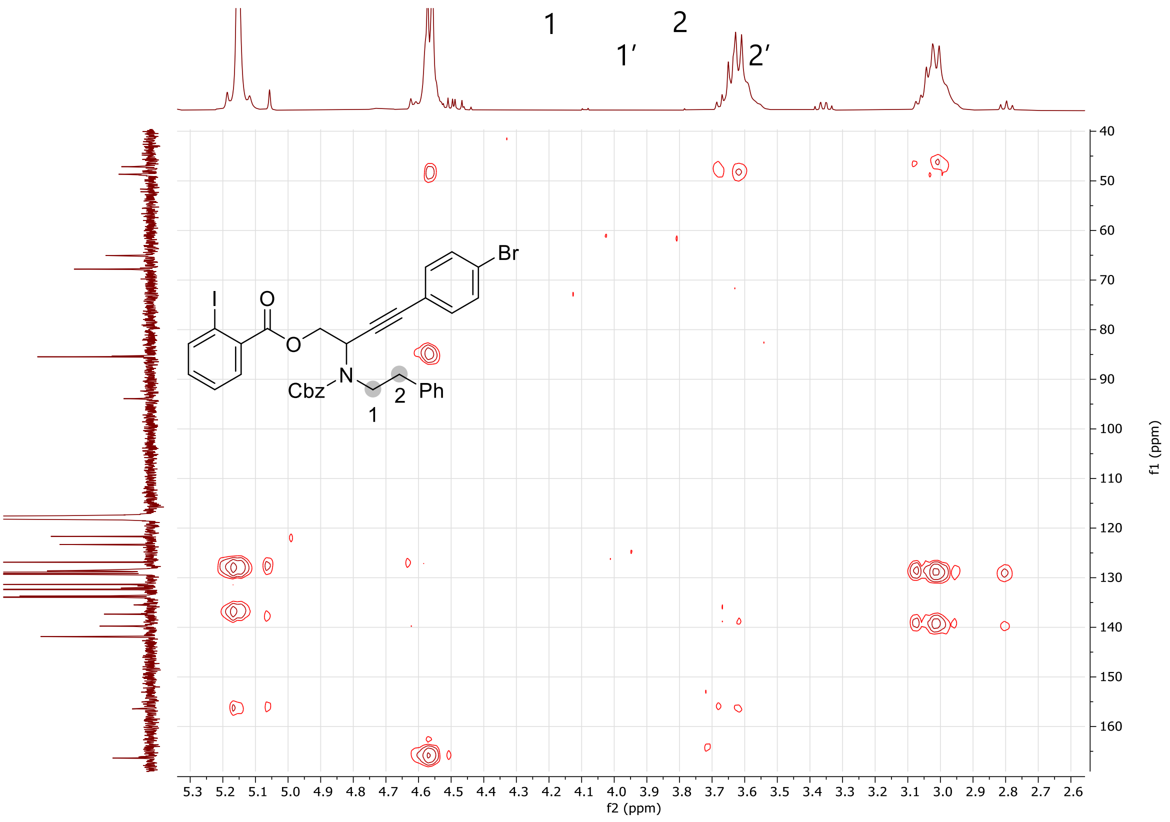

4-(2-bromophenyl)-2-(2-oxooxazolidin-3-yl)but-3-yn-1-yl 2-iodobenzoate (**12c**)

^{1}H NMR (400 MHz, Acetonitrile-d_3)

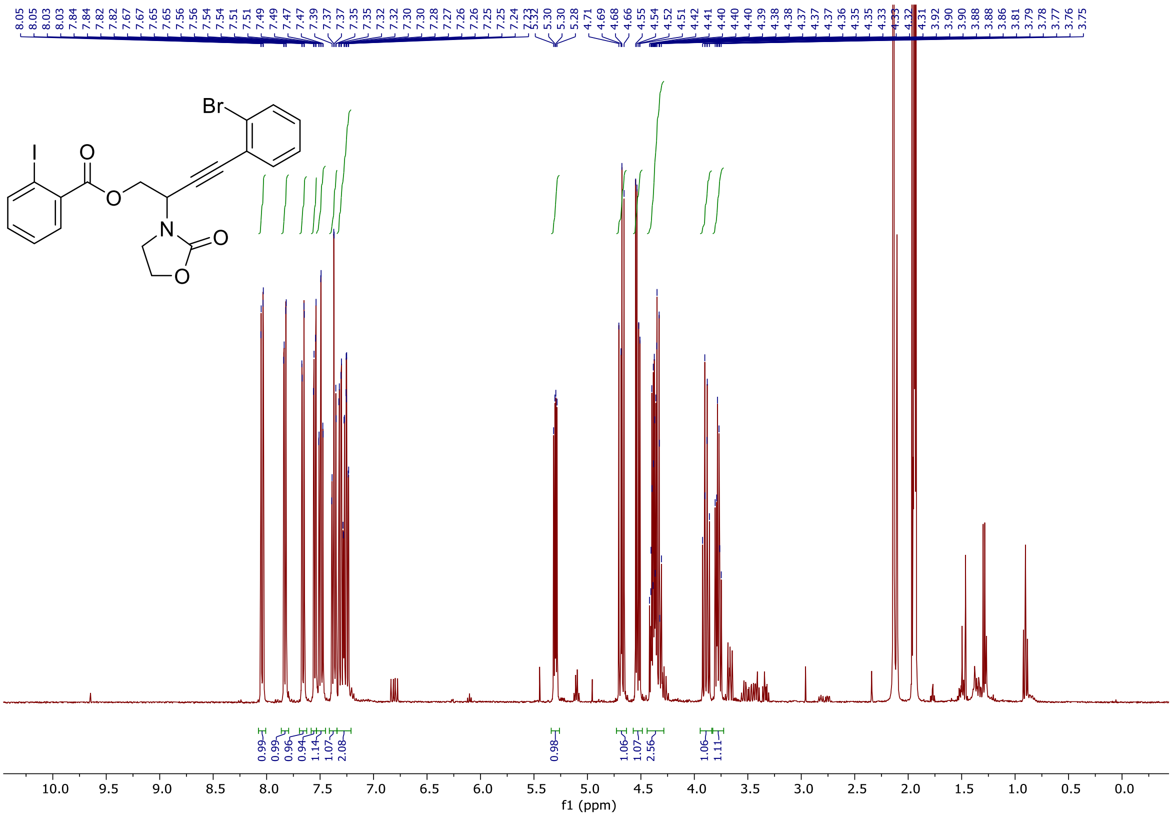

^{13}C NMR (400 MHz, Acetonitrile-d_3)

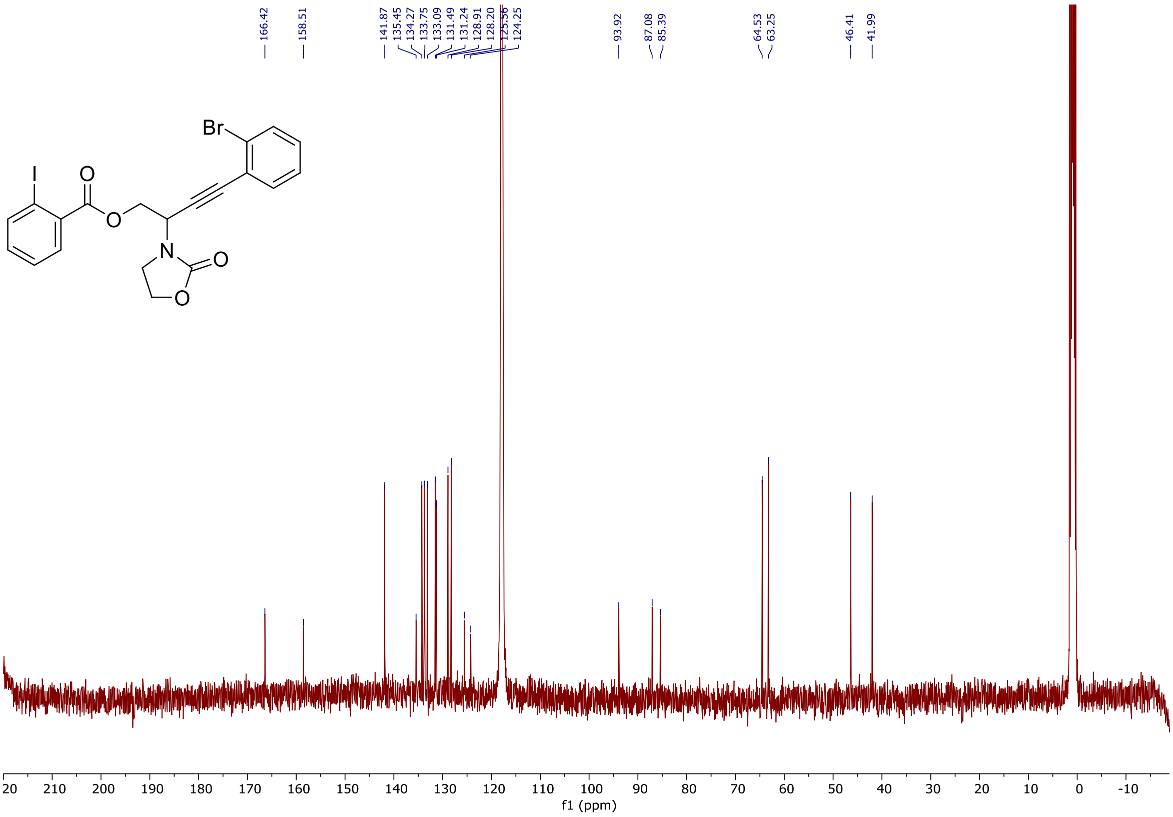

2-(((benzyloxy)carbonyl)(phenethyl)amino)-4-(3-fluorophenyl)but-3-yn-1-yl 2-iodobenzoate (**12d**)

^{1}H NMR (400 MHz, chloroform-*d*)

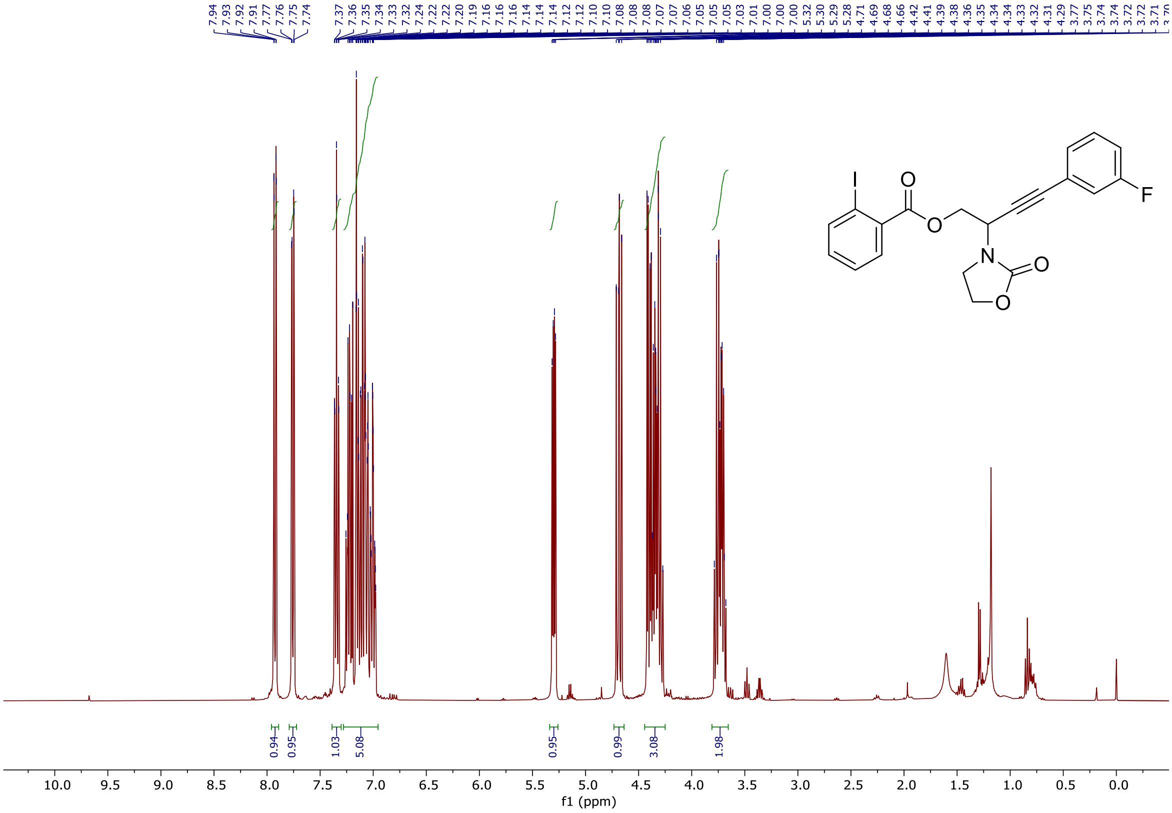

^{13}C NMR (400 MHz, chloroform-*d*)

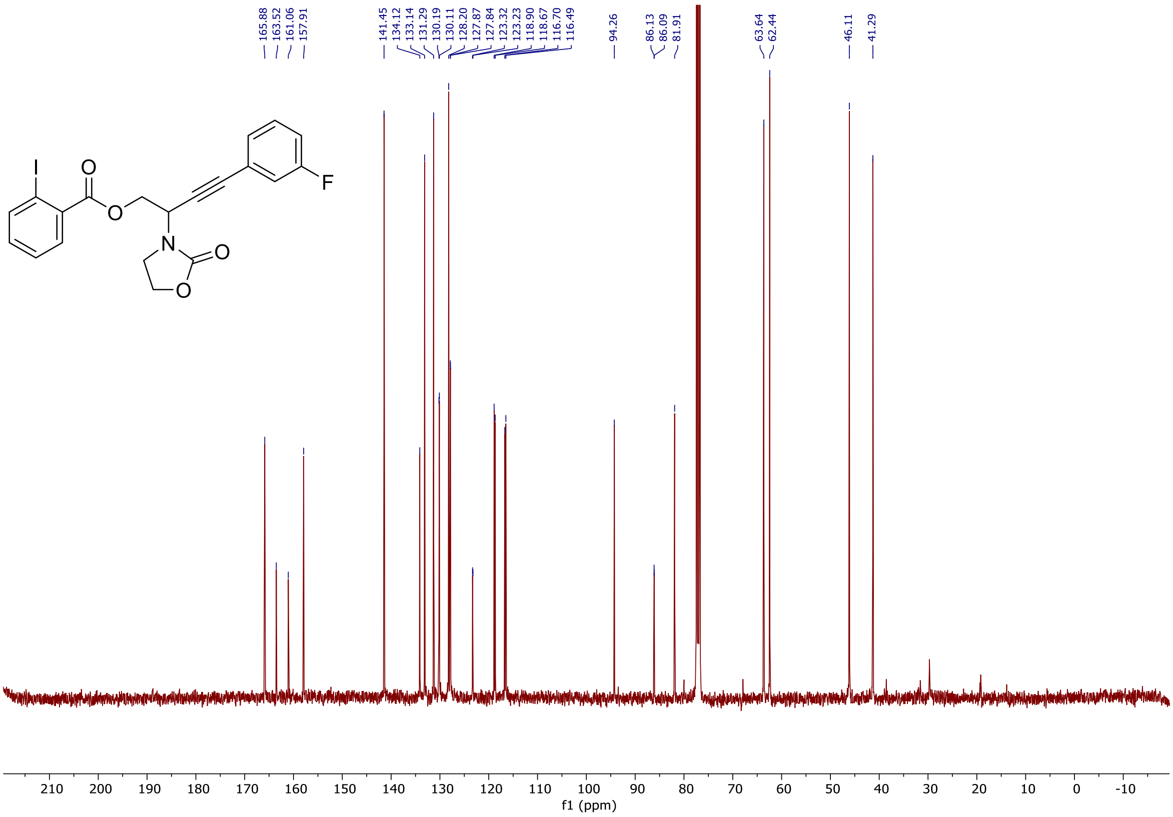

^{19}F NMR (376 MHz, Chloroform-d_3)

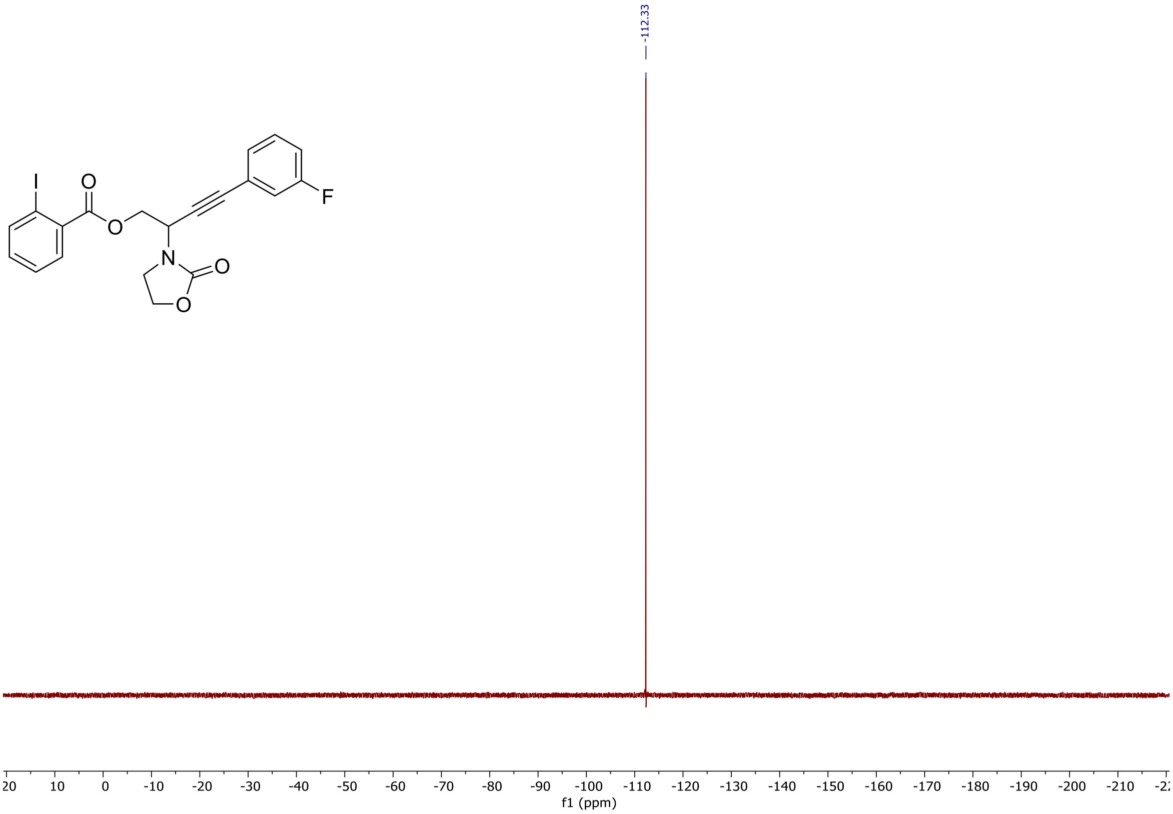

(4-methylphenyl)but-3-yn-1-yl 2-iodobenzoate (**12e**)

^{1}H NMR (400 MHz, Acetonitrile-d_3)

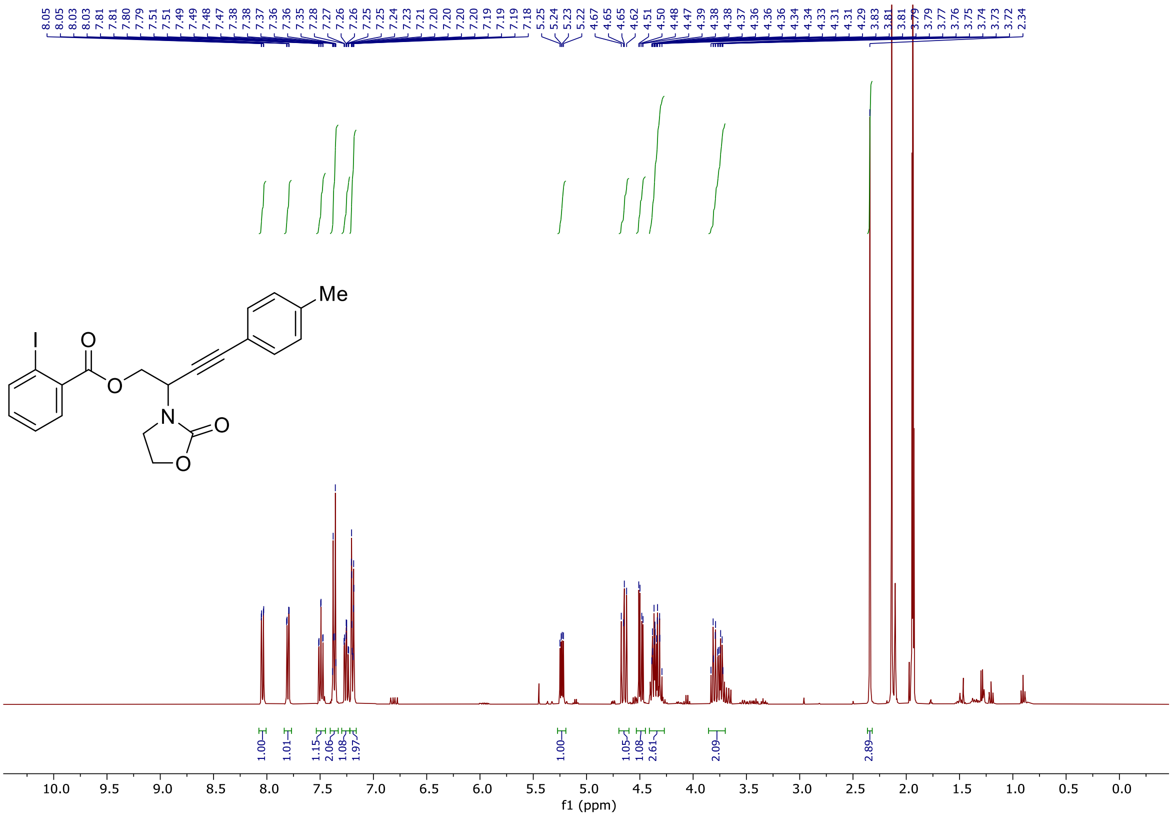

^{13}C NMR (400 MHz, Acetonitrile-d_3)

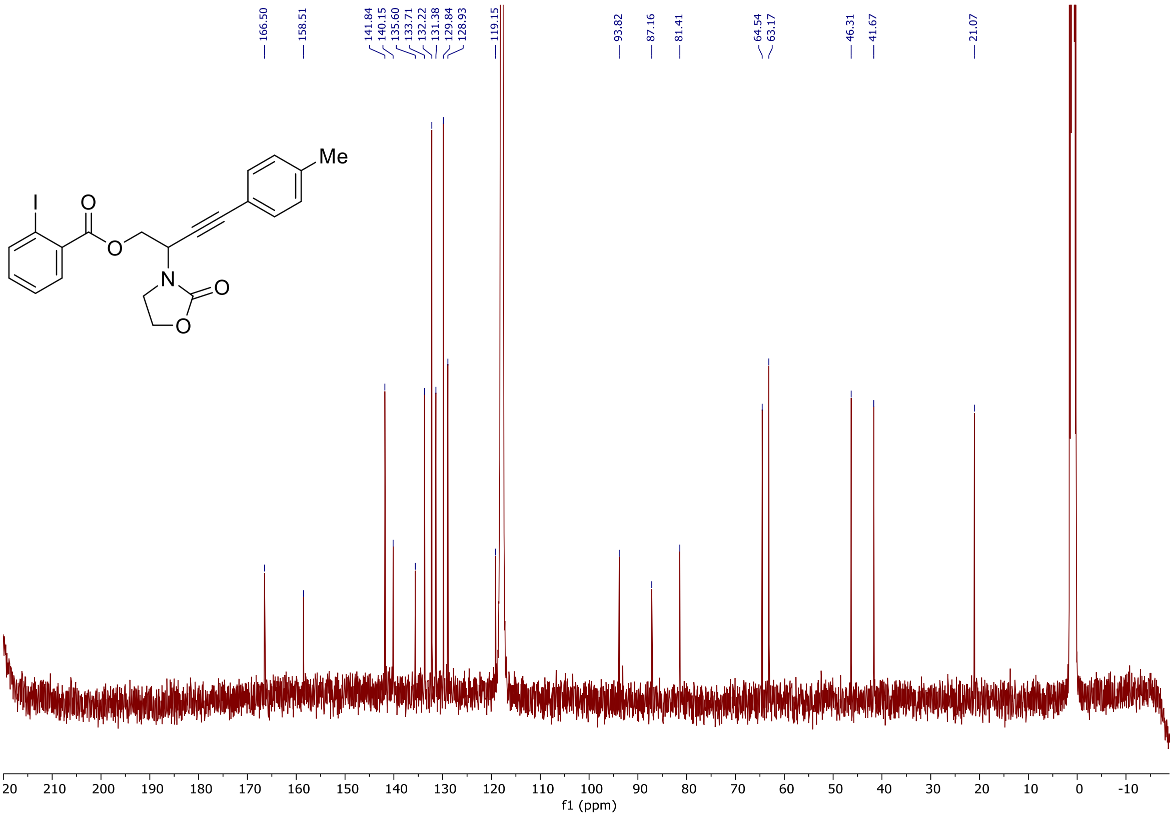

2-(((benzyloxy)carbonyl)(phenethyl)amino)-4-(triisopropylsilyl)but-3-yn-1-yl 2-iodobenzoate (**12f**)

^{1}H NMR (400 MHz, Acetonitrile-d_3)

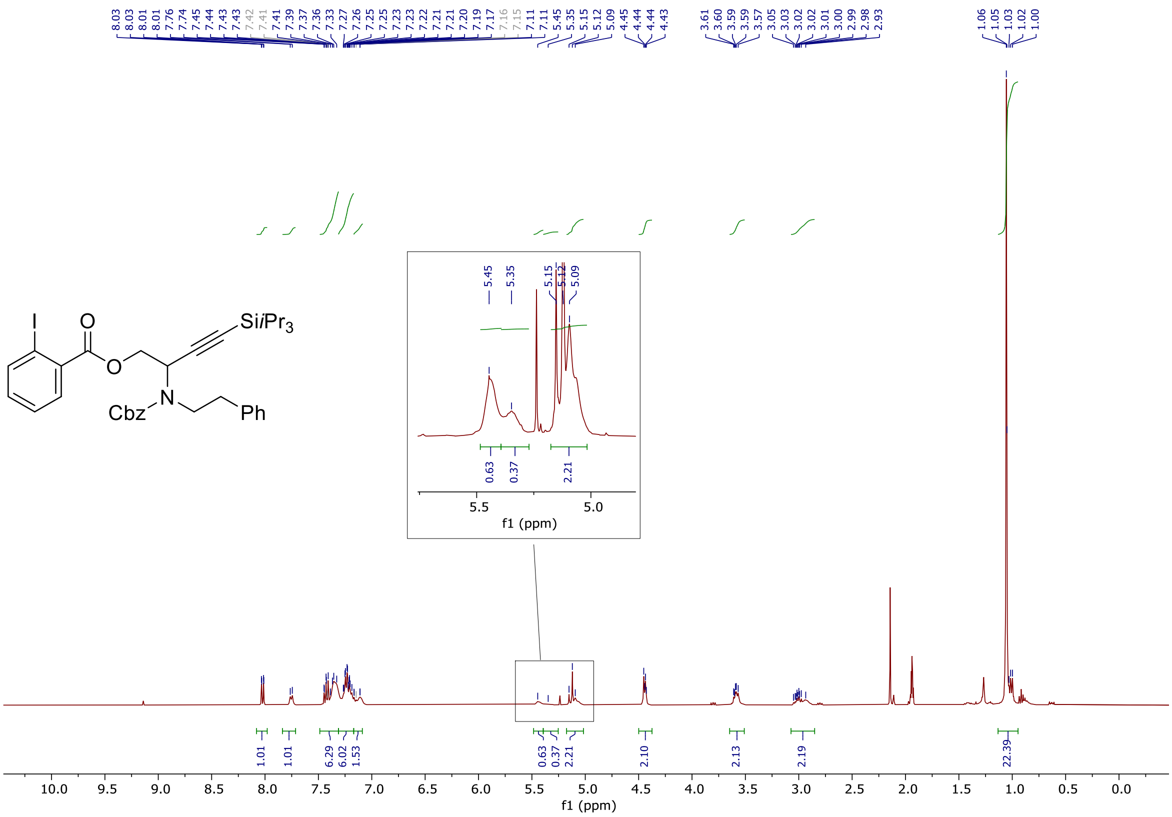

^{13}C NMR (101 MHz, Acetonitrile-d_3)

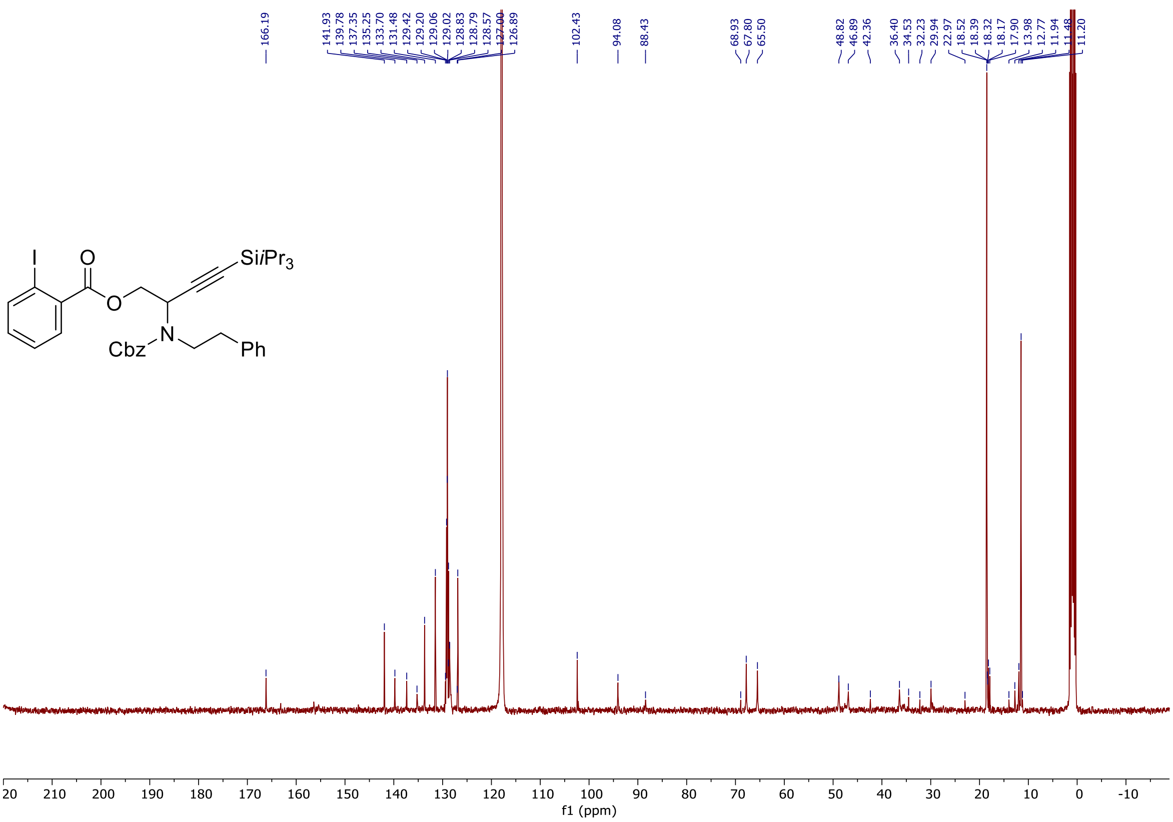

4-(4-((3-bromopropoxy)carbonyl)phenyl)-2-(2-oxooxazolidin-3-yl)but-3-yn-1-yl 2-iodobenzoate (**12g**)

^{1}H NMR (400 MHz, chloroform-*d*)

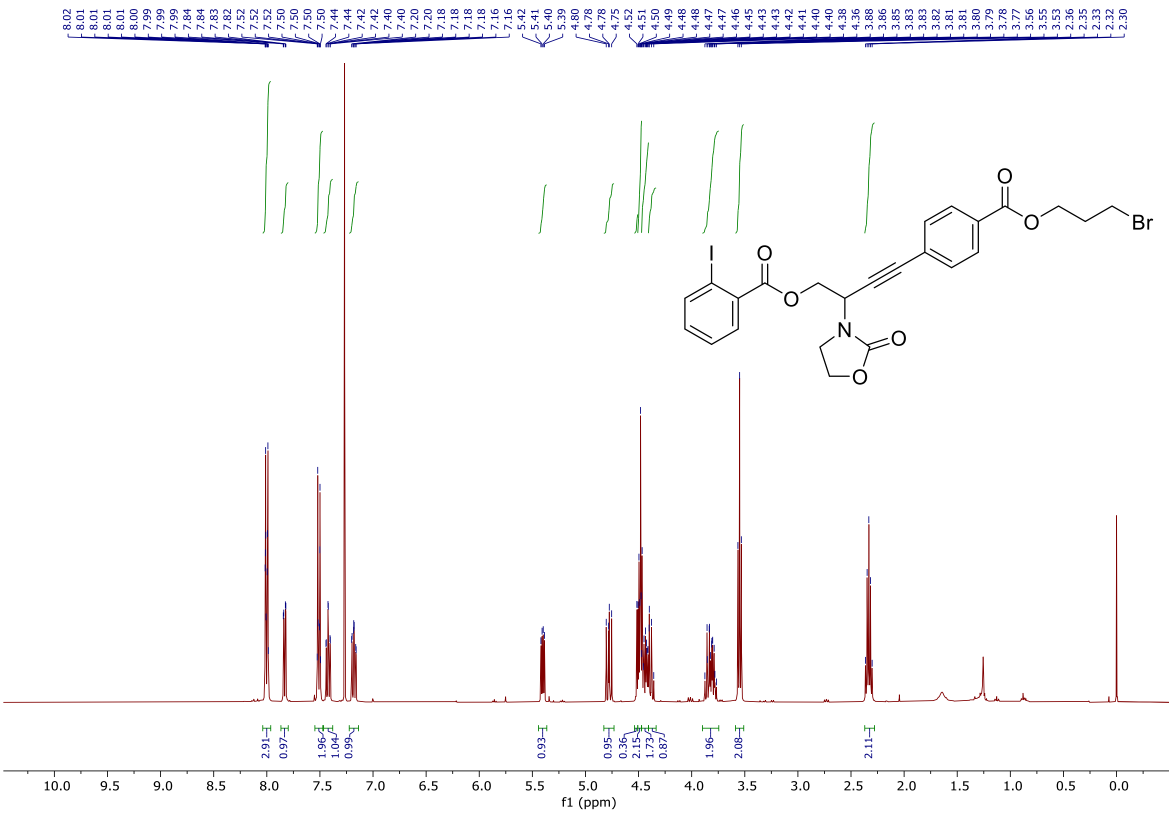

^{13}C NMR (400 MHz, chloroform-*d*)

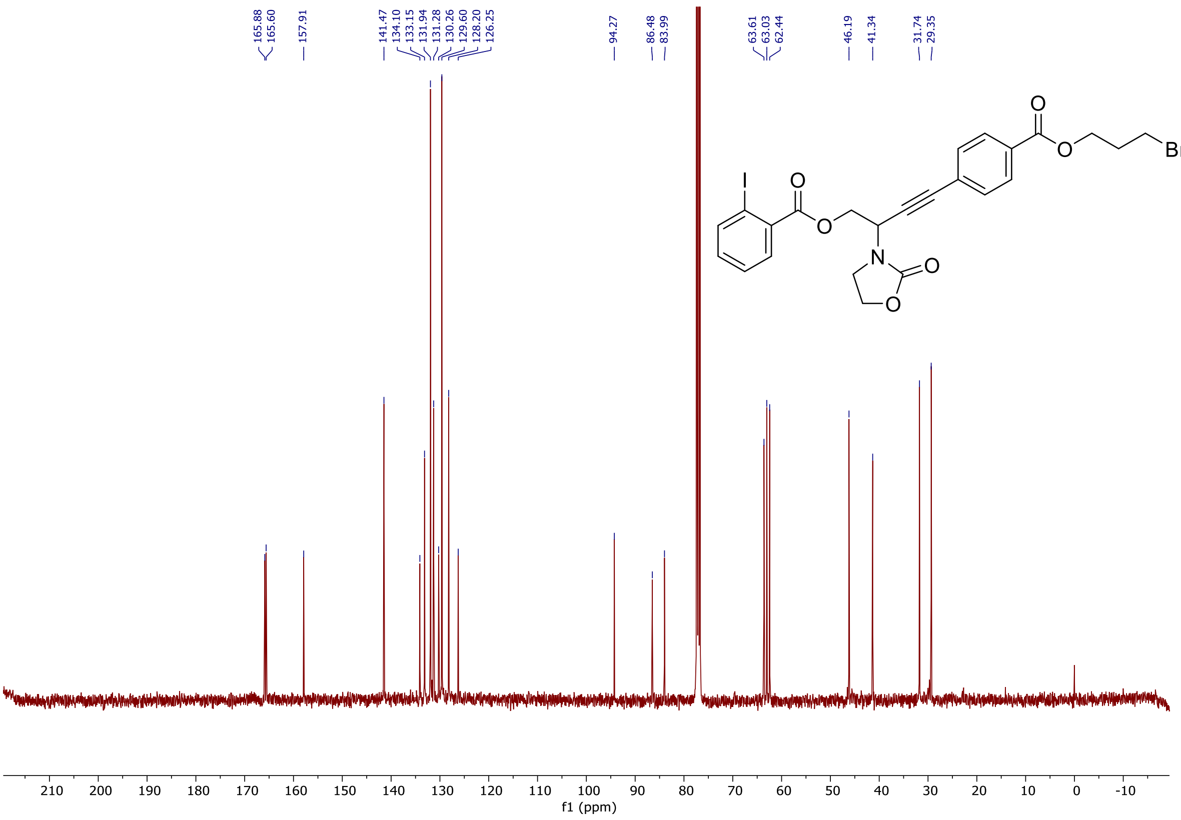

4-(4-((allyloxy)carbonyl)phenyl)-2-(2-oxooxazolidin-3-yl)but-3-yn-1-yl 2-iodobenzoate (**12h**)

^{1}H NMR (400 MHz, chloroform-*d*)

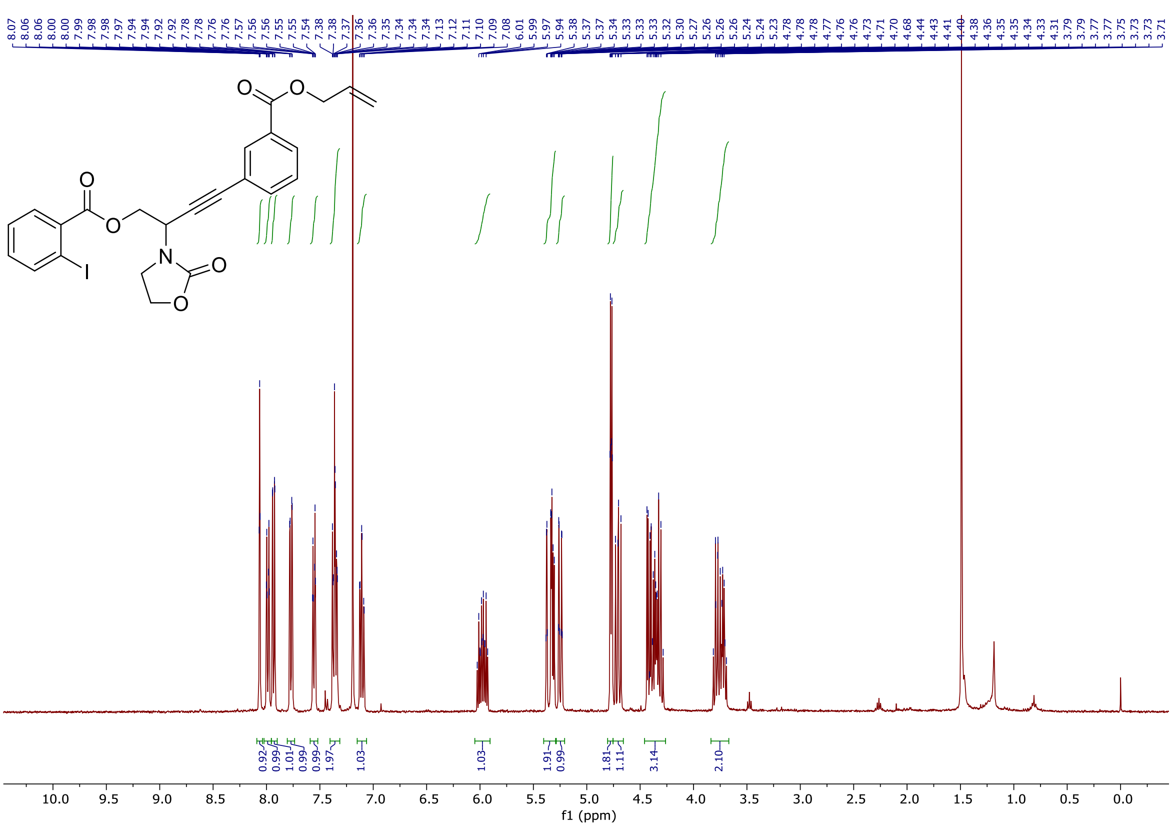

^{13}C NMR (400 MHz, chloroform-*d*)

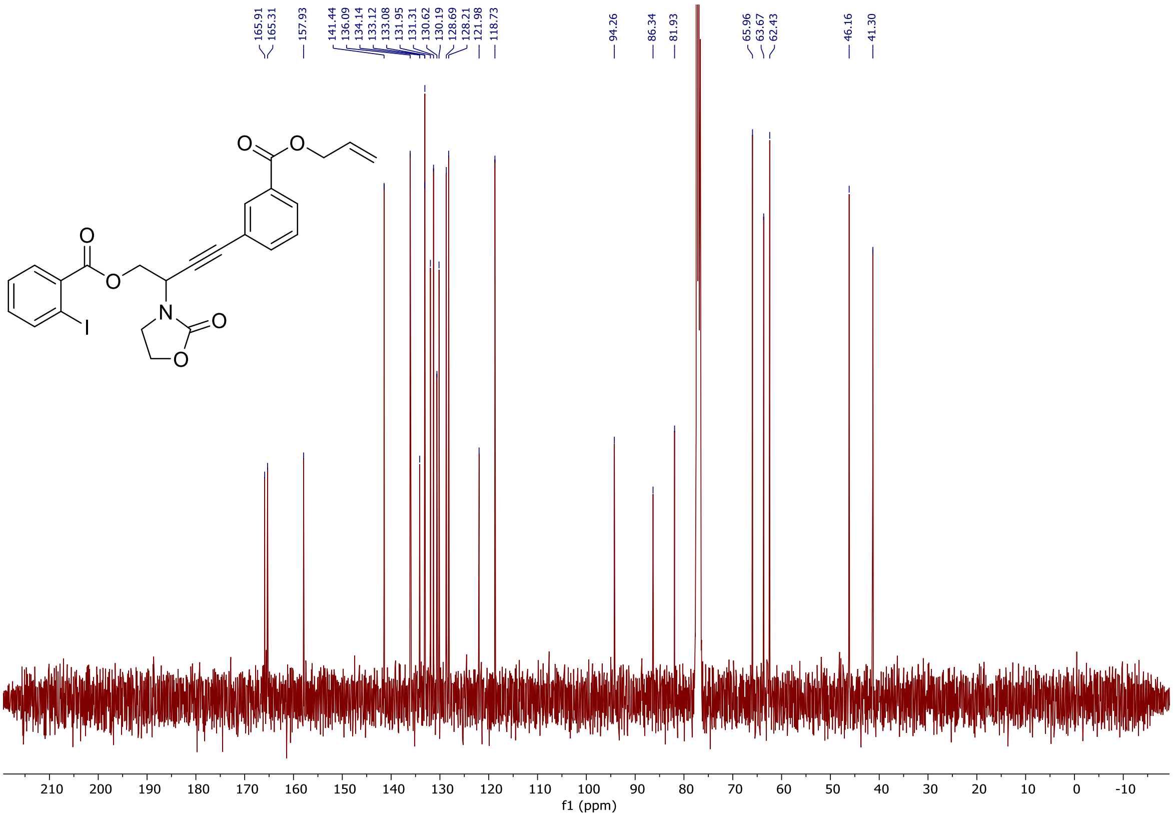

2-butoxy-4-(4-(trifluoromethyl)phenyl)but-3-yn-1-yl 2-iodobenzoate (**12i**)

^{1}H NMR (400 MHz, chloroform-*d*)

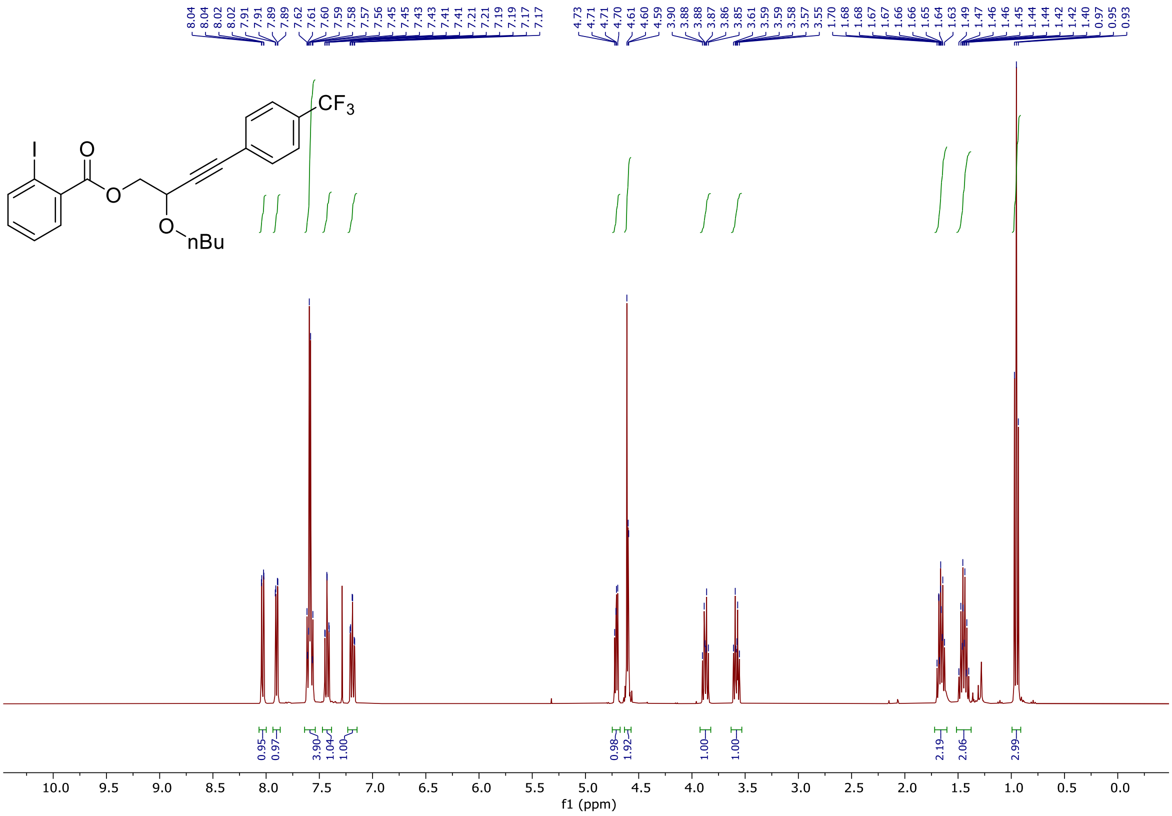

^{13}C NMR (400 MHz, chloroform-*d*)

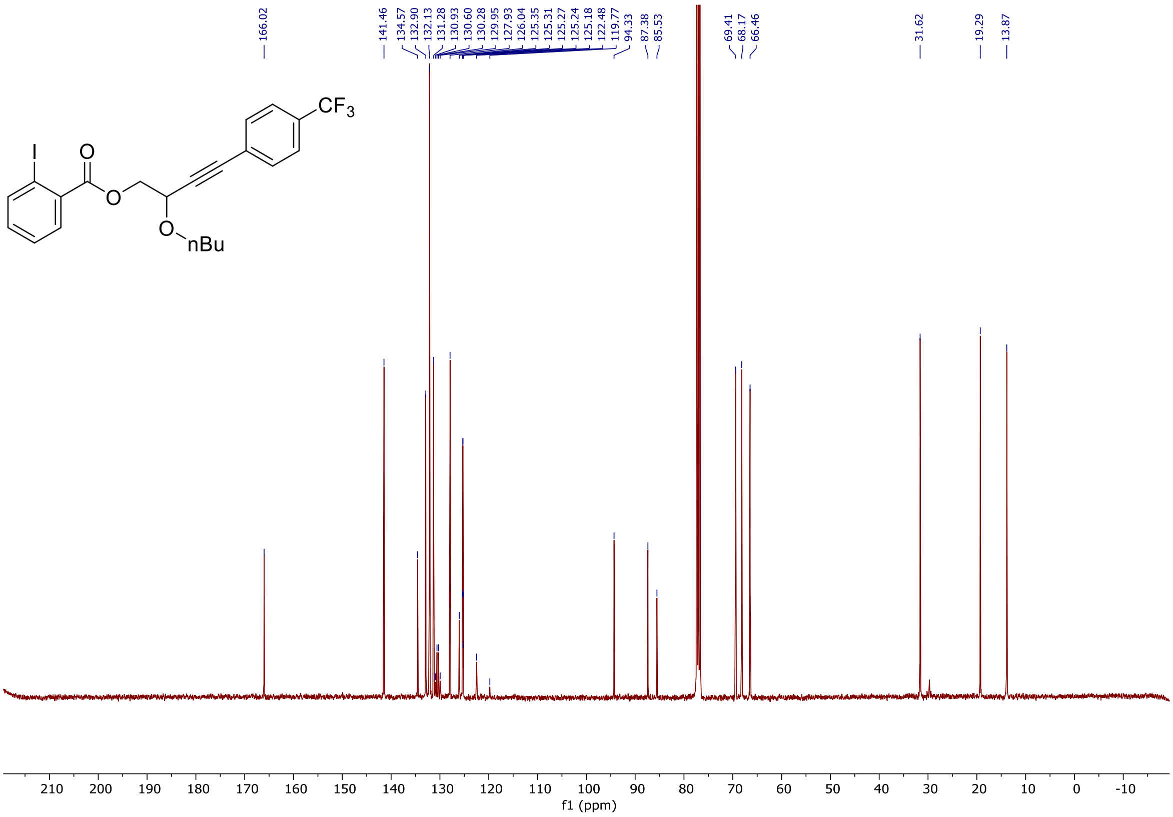

^{19}F NMR (376 MHz, Chloroform-*d*)

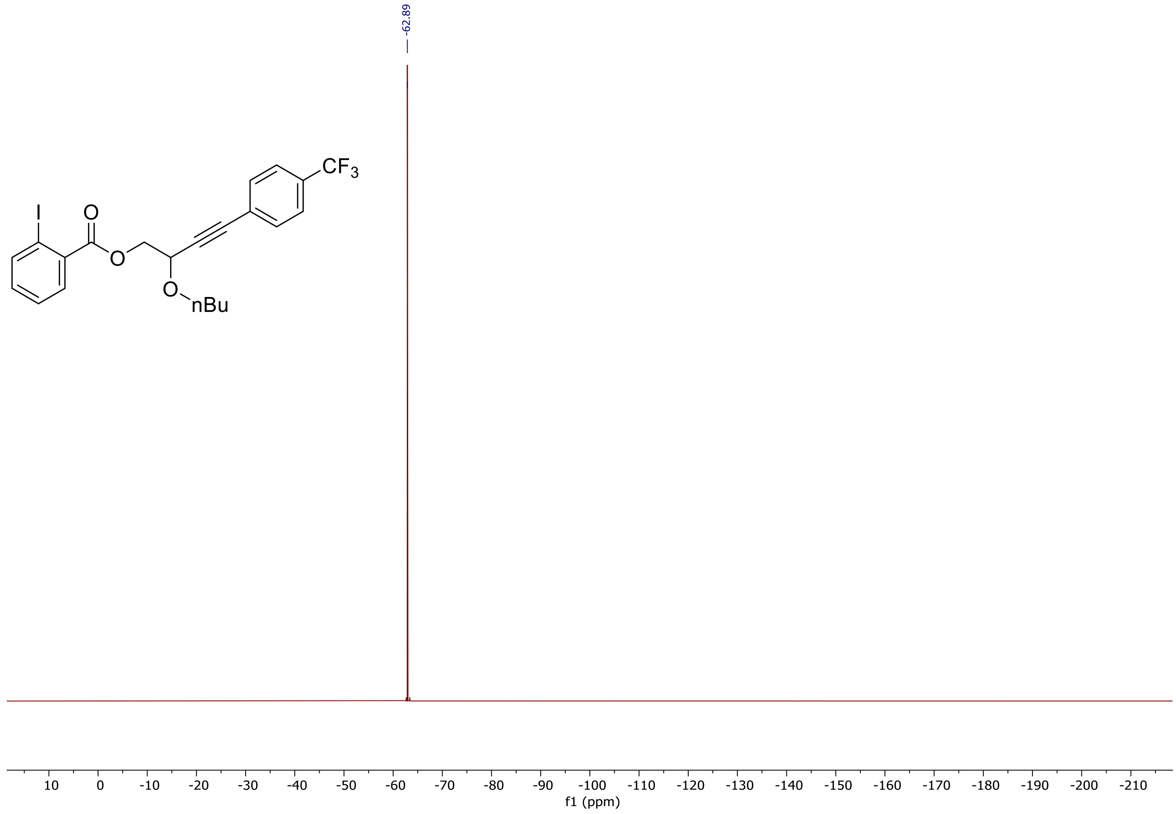

4-(4-((3-bromopropoxy)carbonyl)phenyl)-2-butoxybut-3-yn-1-yl 2-iodobenzoate (**12j**)

^{1}H NMR (400 MHz, chloroform-*d*)

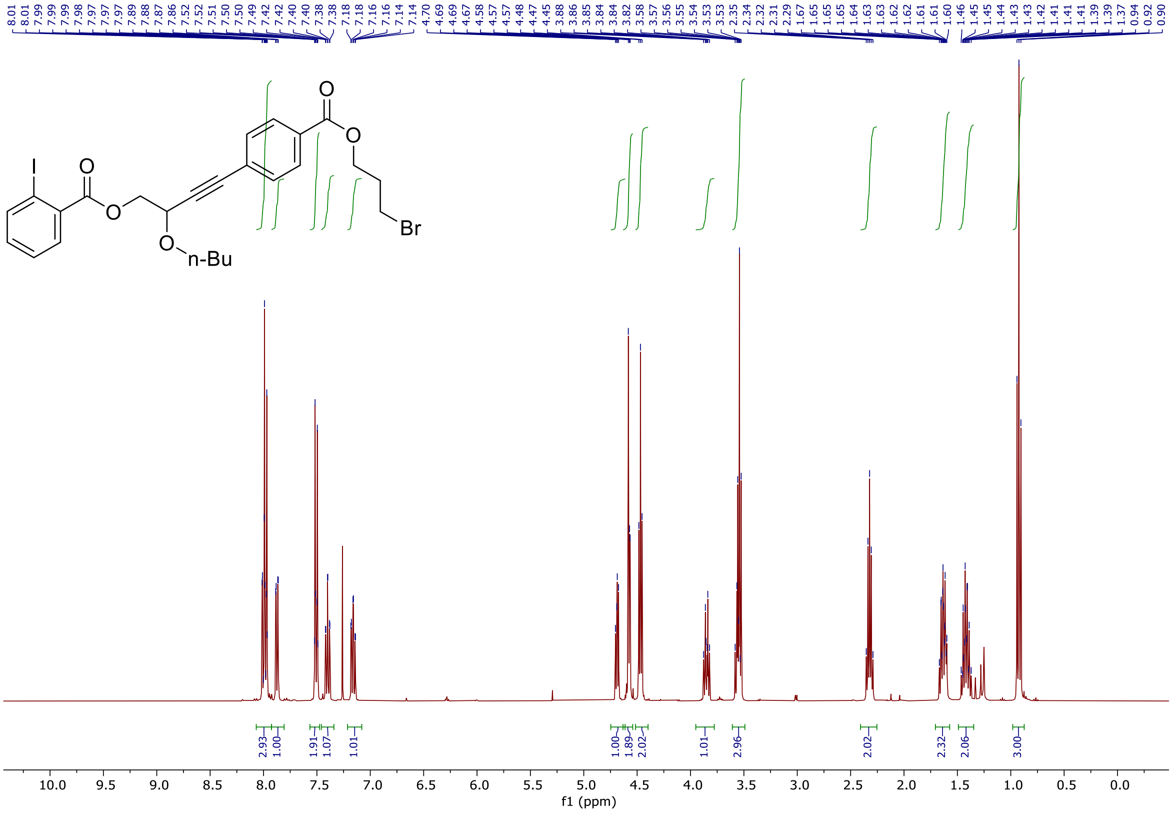

^{13}C NMR (400 MHz, chloroform-*d*)

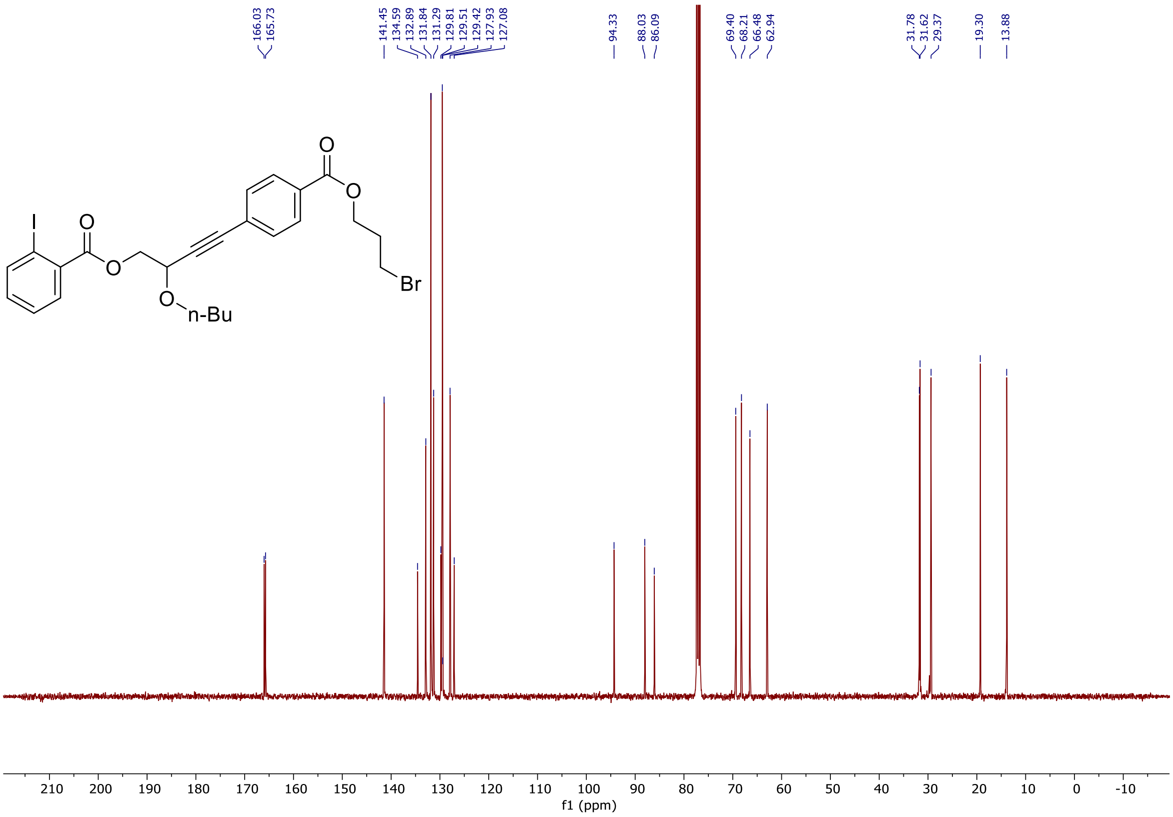

2-(2-oxopyrrolidin-1-yl)-4-phenylbut-3-yn-1-yl acetate (**13**)

^{1}H NMR (Chloroform-d, 400 MHz)

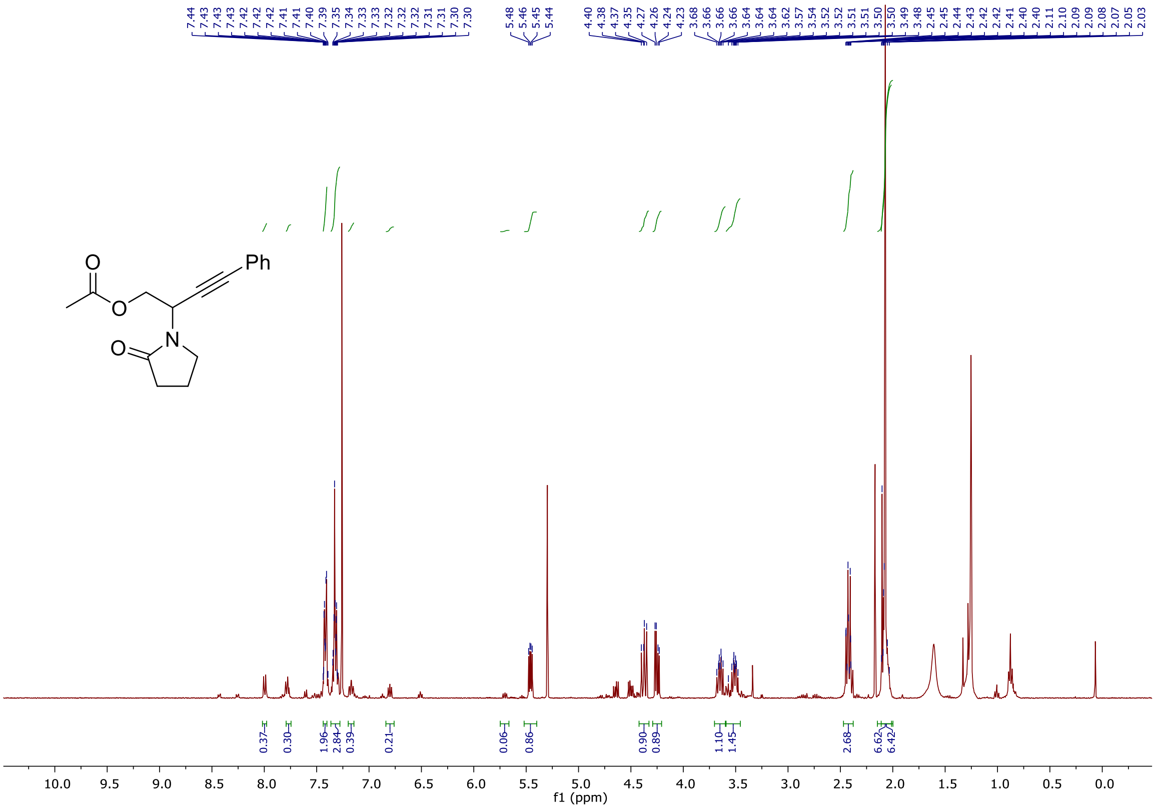

^{13}C NMR (Chloroform-d, 101 MHz)

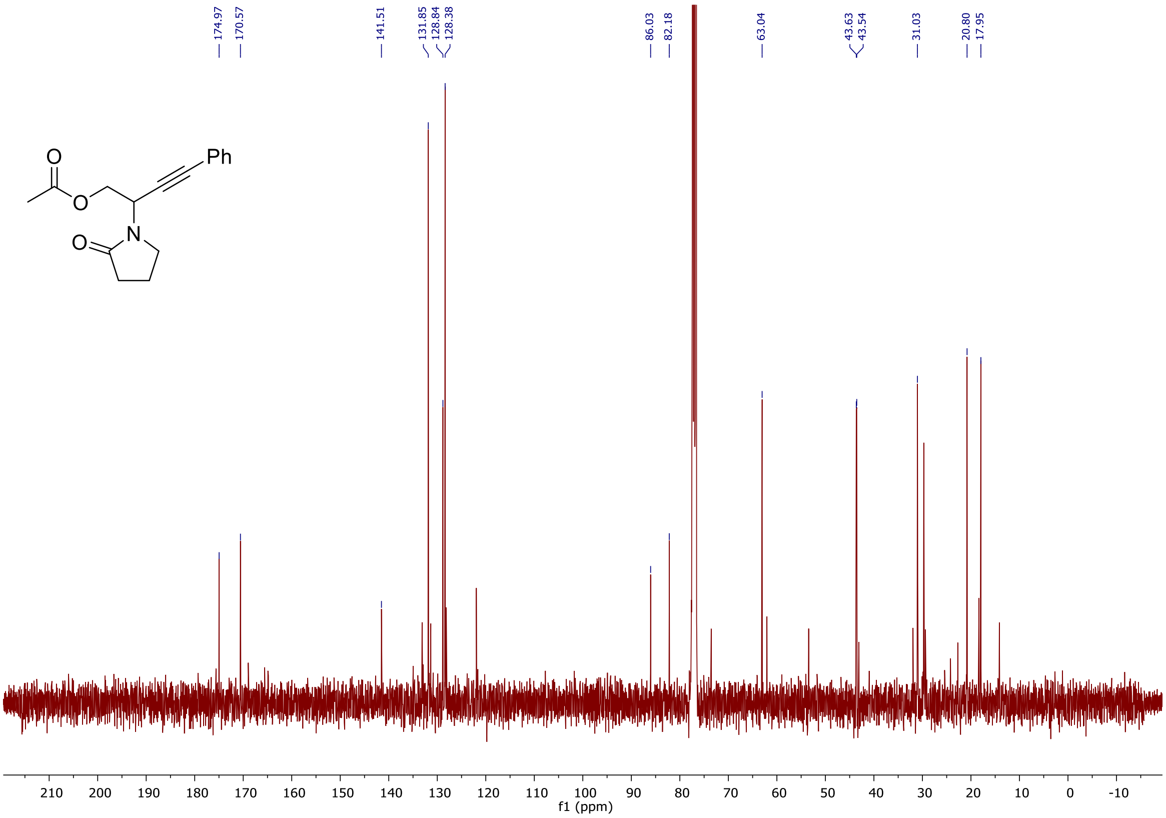

HMBC (Chloroform-*d*) zoom on aromatic/alkyne regions for attribution of the quaternary carbons

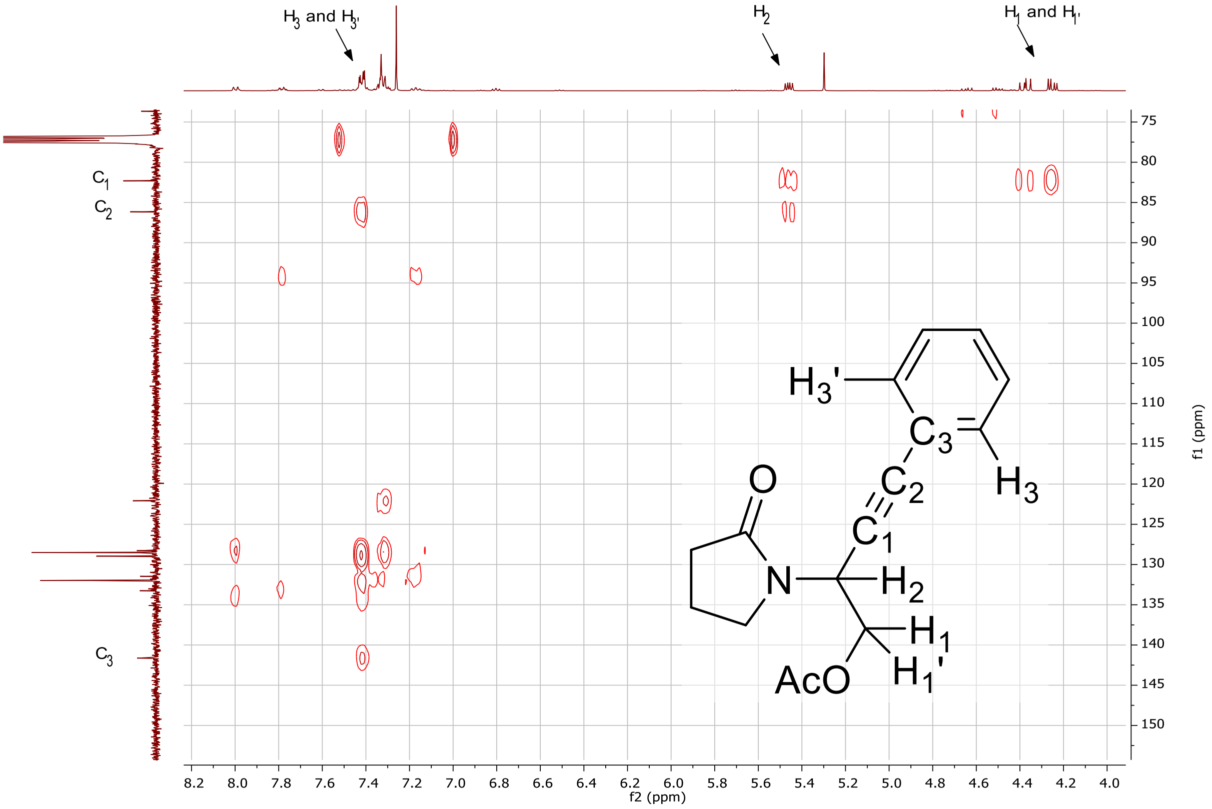

Product modification

1-(1-hydroxy-4-phenylbut-3-yn-2-yl)pyrrolidin-2-one (**14**)

^{1}H NMR (400 MHz, Chloroform-*d*)

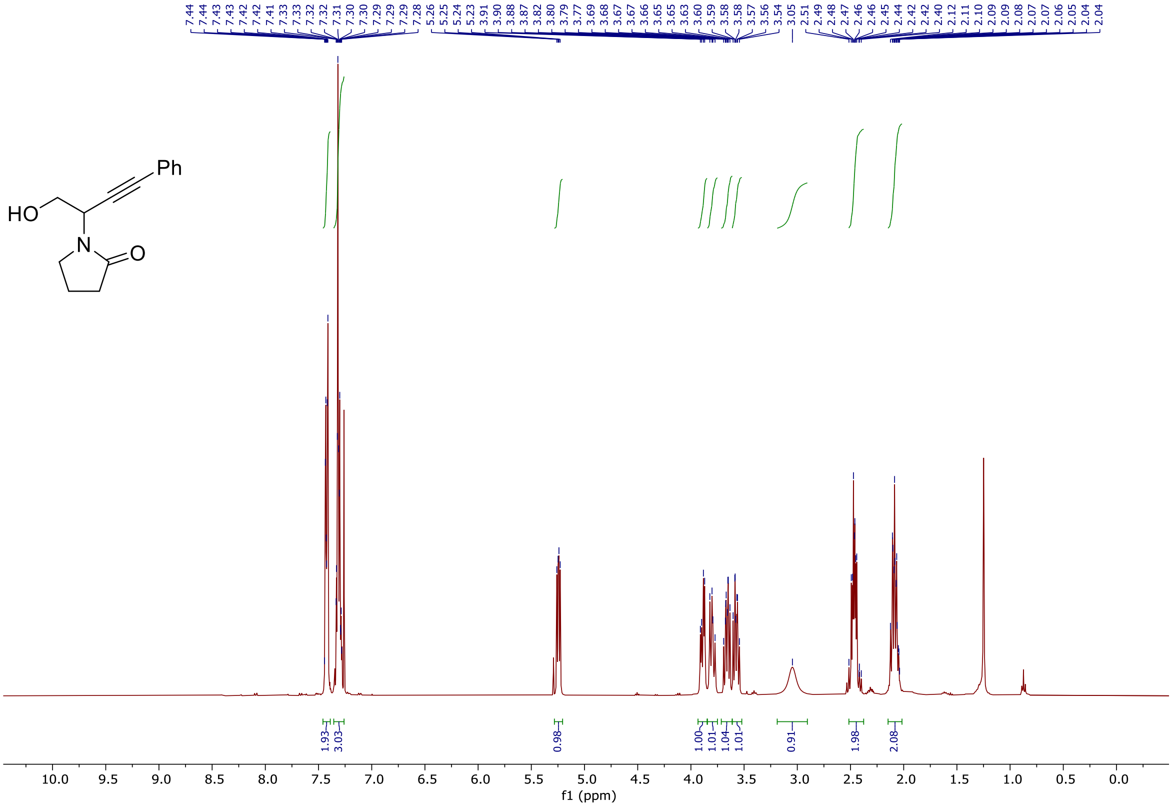

^{13}C NMR (101 MHz, Chloroform-*d*)

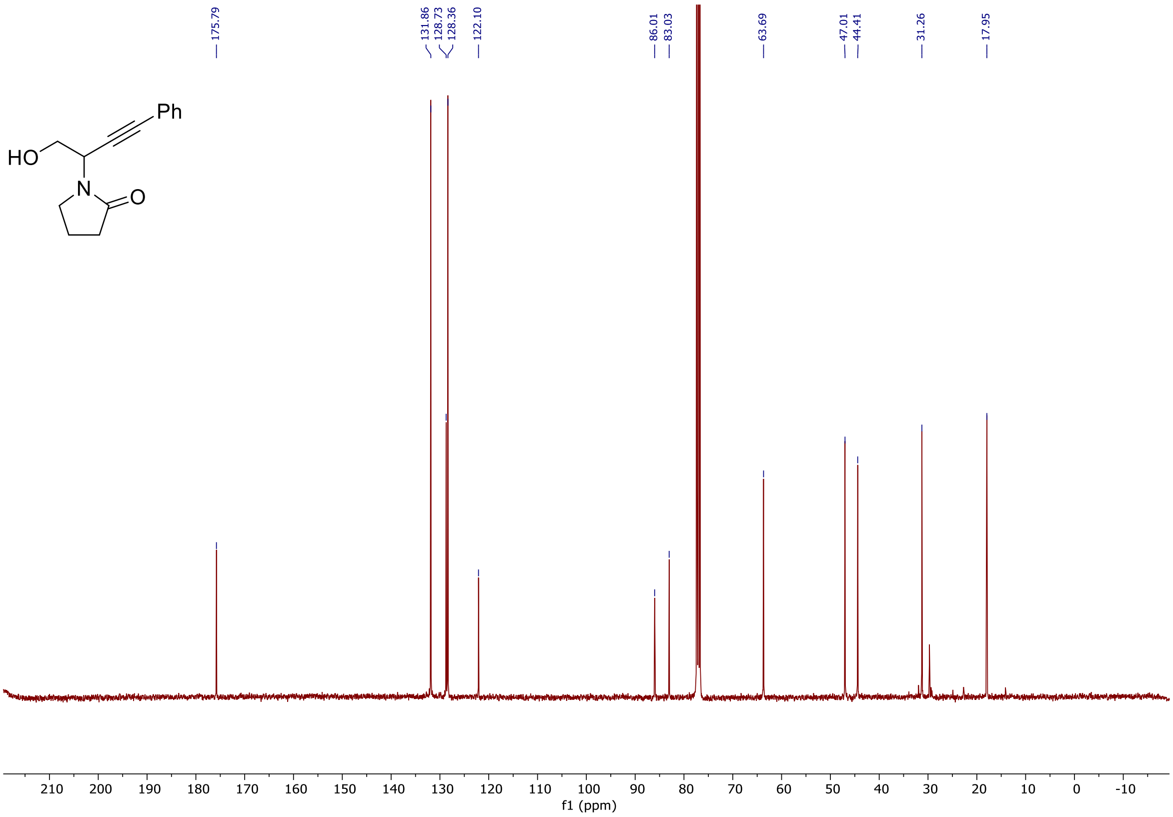

2-butoxy-4-phenylbut-3-yn-1-ol (**15**)

^{1}H NMR (400 MHz, chloroform-*d*)

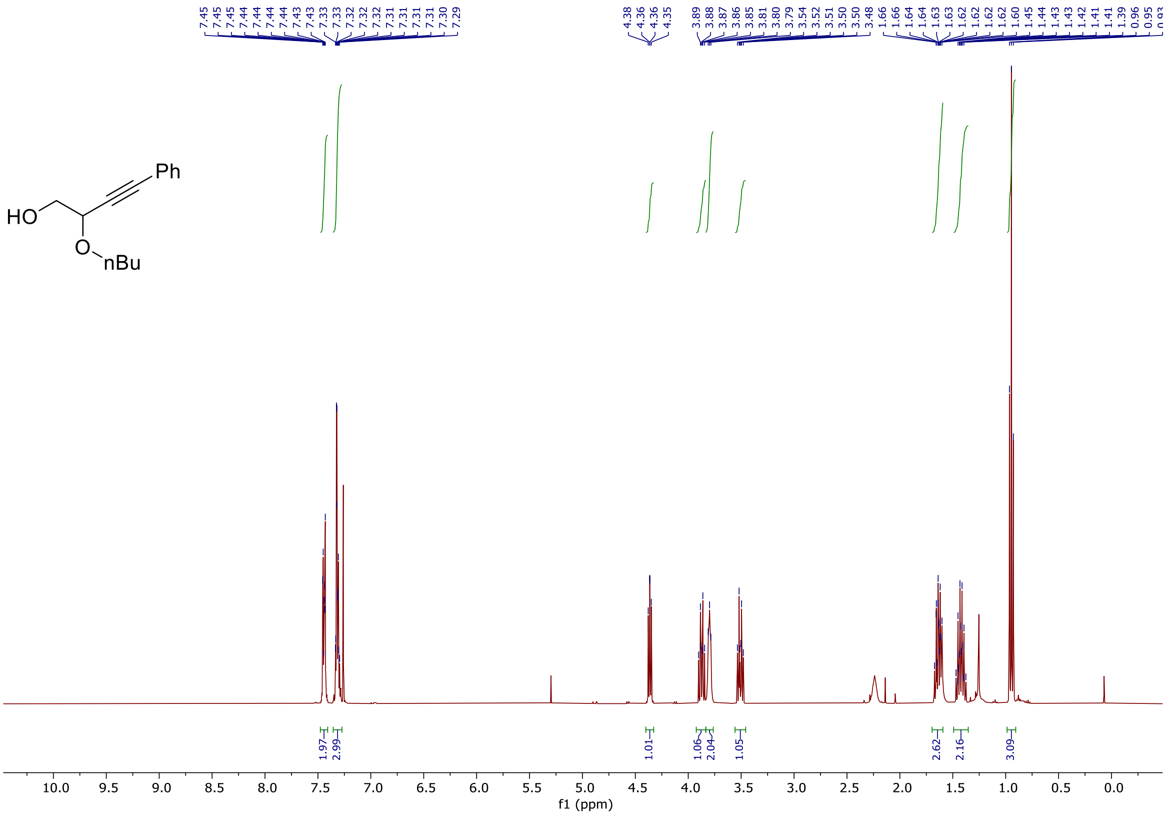

^{13}C NMR (400 MHz, chloroform-*d*)

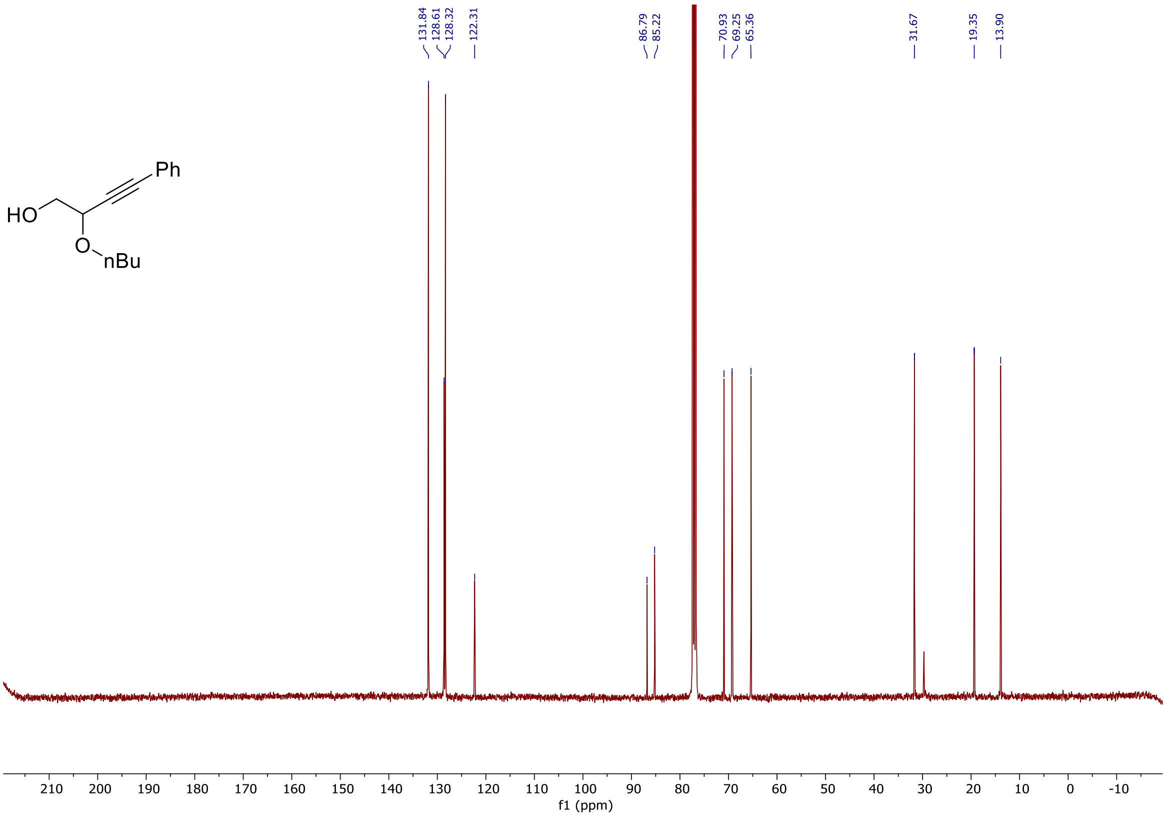

2-(phenethylamino)-4-phenylbut-3-yn-1-yl 2-iodobenzoate (**16**)

^{1}H NMR (400 MHz, Acetonitrile-*d*$_3$)

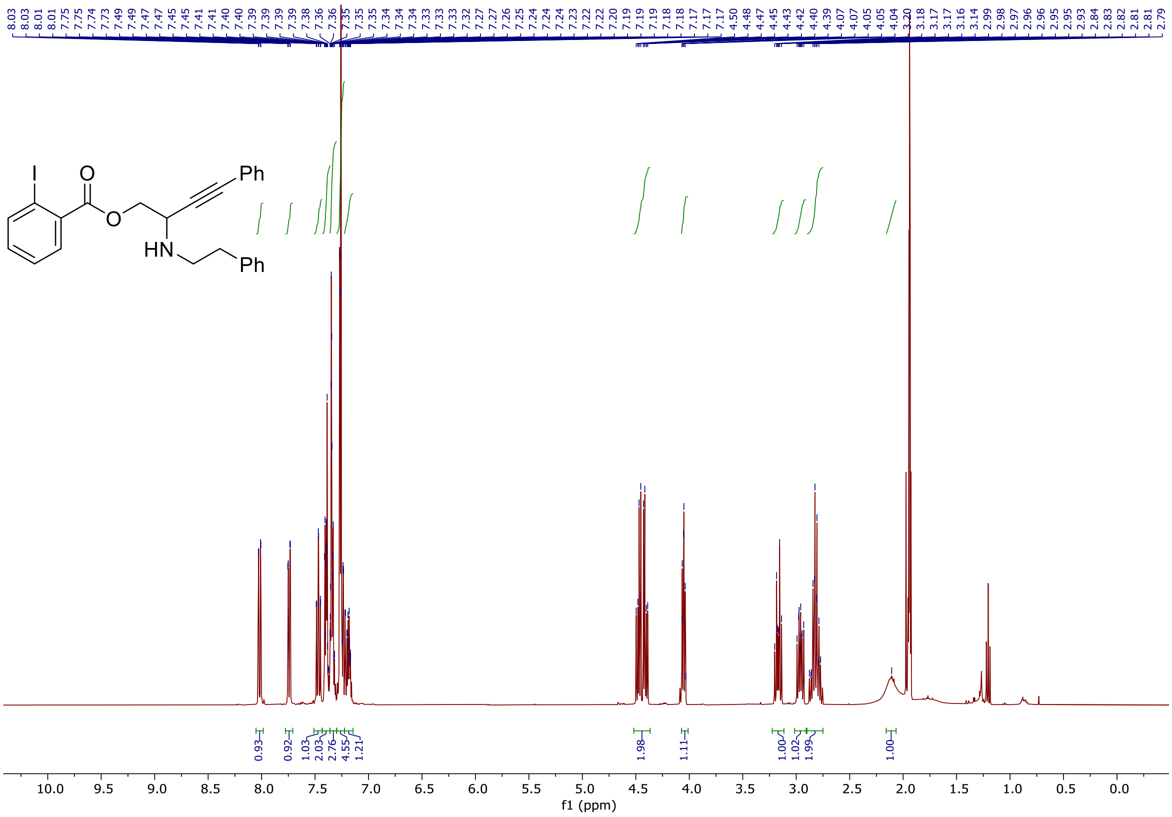

^{13}C NMR (101 MHz, Acetonitrile-*d*$_3$)

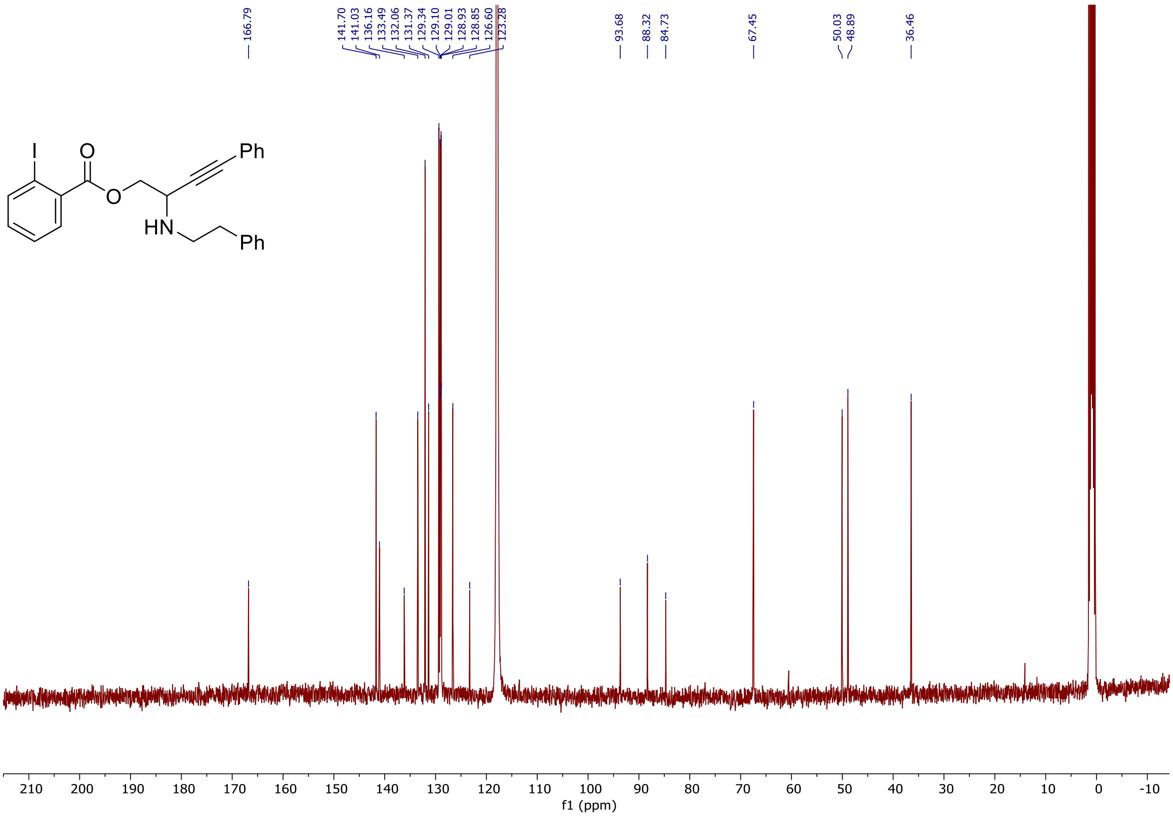

Supplement: SC-011-D0SC03655B-s001 [file SC-011-D0SC03655B-s001.pdf]
